# Inside or outside the box? Effect of substrate location on coordination-cage based catalysis†

**DOI:** 10.1039/d2dt01713j

**Published:** 2022-06-30

**Authors:** Atena B. Solea, Burin Sudittapong, Christopher G. P. Taylor, Michael D. Ward

**Affiliations:** Department of Chemistry, University of Warwick Coventry CV4 7AL UK m.d.ward@warwick.ac.uk

## Abstract

In this work we compare and contrast the hydrolysis of two different aromatic esters using an octanuclear cubic Co_8_ coordination cage host as the catalyst. Diacetyl fluorescein (DAF) is too large to bind inside the cage cavity, but in aqueous solution it interacts with the exterior surface of the cage *via* a hydrophobic interaction with *K* = 1.5(2) × 10^4^ M^−1^. This is sufficient to bring it into close proximity to the layer of hydroxide ions which also surrounds the 16+ cage surface even at modest pH values, accelerating the hydrolysis of DAF to fluorescein with *k*_cat_/*k*_uncat_ (the rate acceleration for that fraction of DAF in contact with the cage surface in the equilibrium) ≈50. This is far smaller than many known examples of catalysis inside a cage cavity, but at the exterior surface it is potentially general with no cavity-imposed size/shape limitations for guest binding. In contrast 4-nitrophenyl acetate (4NPA) binds inside the cage cavity with *K* = 3.5(3) × 10^3^ M^−1^ and as such is surrounded in solution by the hydroxide ions which accumulate around the cage surface. However its hydrolysis is actually inhibited: either because of a geometrically unfavourable geometry of the bound substrate which makes it inaccessible to surface-bound hydroxide, or because the necessary volume expansion/geometry change associated with formation of a tetrahedral intermediate cannot be accommodated inside the cavity. Any 4NPA that is free in solution as part of the equilibrium undergoes catalysed hydrolysis at the cage exterior surface in the same way as DAF, but the effect is limited by the low affinity of 4NPA for the exterior surface. We conclude that exterior-surface catalysis can be effective and potentially general; and that cavity-binding of guests can result in negative, rather than positive, catalysis.

## Abstract 2

The cavity is not everything! Catalysed hydrolysis of 4-nitrophenylacetate is inhibited *inside* a cage (left), but hydrolysis of diacetylfluorescein (right) is catalysed by the exterior surface.

## Graphical abstract

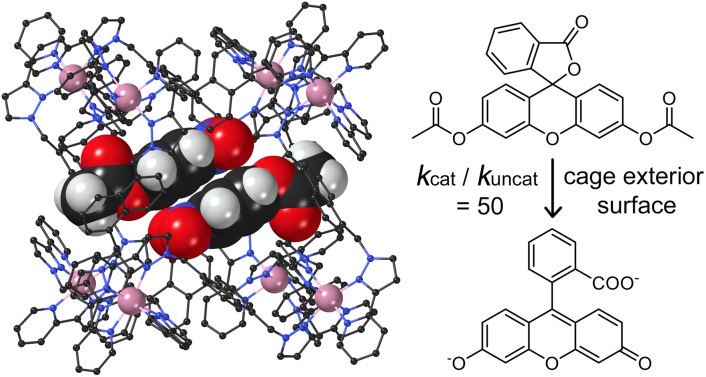

## Introduction

One of the most interesting and widely studied applications of coordination cages is their ability to catalyse reactions of guests that occupy the central cavity.^1^ The environment of a guest molecule encapsulated inside the cavity of a host is quite different from that in bulk solution, and can result in substantially changed reactivity for many distinct reasons. These may include geometric factors such as co-location of two species giving a high local concentration,^2,3^ or the ‘constrictive binding’ of flexible substrates which have to fold up to fit inside a host cage, resulting in adoption of a geometry close to a reaction transition state.^4^ An electronic effect has also been identified whereby the high charge on a coordination cage can substantially change the equilibria associated with protonation or deprotonation of bound guests,^5^ with a highly negatively charged cage, for example, making acid-based catalysis of bound guests much easier by stabilising their protonated forms.

**Fig. 1:**
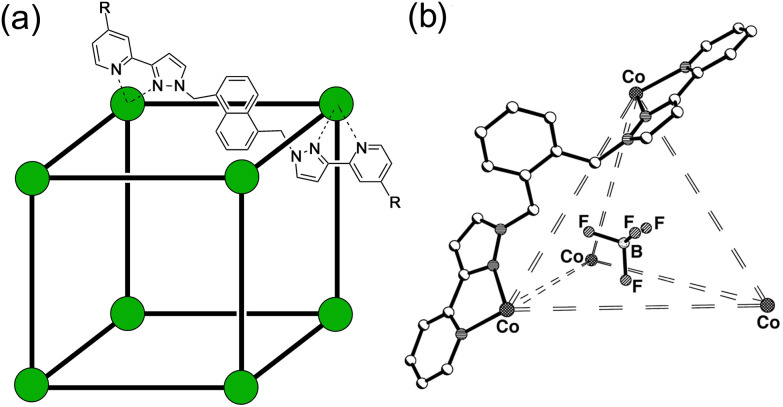
(a) The cages **H**^**w**^ [R = CH_2_OH, Co(ii) vertices] used for solution studies, and **H**^**Ni**^ [R = H, Ni(ii) vertices] used for the crystallography; and (b) **T** (used for control experiments).

Our own work on cage-based catalysis using the octanuclear cubic cage family [M_8_L_12_]^16+^ (Fig. 1) has demonstrated a catalytic mechanism that is based on a combination of two effects.^3,6^ Binding of an organic guest in the cage cavity in aqueous solution is driven predominantly by the hydrophobic effect.^3*a*^ Additionally the high positive charge of the cage (16+) attracts counter-ions which accumulate around the cage surface, occupying in particular portals in the cage faces where they can bind strongly.^3*a*,6^ The very high local concentration of surface-bound anions surrounding the cavity-bound substrate provides co-location of two different reaction partners which can result in effective catalysis.

Thus, the Kemp elimination reaction of cavity-bound benzisoxazole with surface-bound hydroxide was accelerated by a factor of >10^5^ fold,^3*c*^ because the accumulation of hydroxide ions around the 16+ cage surface resulted in a local concentration of hydroxide ions of the order of 0.1 M surrounding the benzisoxazole unit, even when [HO^−^] in the bulk solution pH is in the μM range.

It is significant that the two interactions that result in co-location of the reaction partners are essentially orthogonal:^6*a*^ guest binding in the cavity is based on the hydrophobic effect, whereas accumulation of desolvated anions around the cage surface is driven by electrostatics. In principle this can allow a wide range of cavity-bound substrate/surface-bound anions to be brought together as reaction partners. For example we have demonstrated that surface-bound phenolate ions can act as bases, instead of hydroxide ions, reacting with cavity-bound benzisoxazole in an autocatalytic process;^3*d*^ and likewise a cage-catalysed aldol condensation of indane-dione involves the co-location of a neutral molecule and its anionic enolate as the two reaction partners.^7^

Recent investigations into extending the scope of this type of cage-based catalysis showed, unexpectedly, that it can also occur at the exterior surface of the cage: binding of the substrate inside the cavity is not essential, although catalysis at the external surface is much slower. The hydrophobic effect that provides strong guest binding inside the cavity, when the guest is a good fit to the cage interior surface, operates also at the external surface, although cage/guest interactions will naturally be weaker due to lack of complete encapsulation and sub-optimal complementarity of the associated hydrophobic surfaces. Nonetheless we have demonstrated that reactions such as organophosphate ester hydrolysis,^8^ the Kemp elimination with 5-nitrobenzisoxazole,^6*a*^ and the aldol condensation mentioned above, can all be catalysed by the [M_8_L_12_]^16+^ cubic cage in water^7^ – even when the reaction cannot be occurring inside the cavity because either (i) the substrate is too large to bind in the cavity, or (ii) the cavity is blocked by a strongly-bonding inhibitor. The exterior surface of the cage therefore acts as the catalyst by accumulating both the organic substrate and the anionic reactions partners around the cage surface *via* the two independent interactions mentioned above: there is an obvious parallel with catalysis inside hydrophobic micelles which is based on the same combination of effects.^9^

Here we report further studies into the ability of the [M_8_L_12_]^16+^ cubic cage family to act as a catalyst for reactions between organic substrates and anions. To extend the scope of the reaction types that can be catalysed, we have investigated ester hydrolysis. Accordingly the substrates in both cases are both acetate esters: diacetyl fluorescein (DAF), and 4-nitrophenyl-acetate (4NPA). In one case, with DAF as substrate, we observed significant catalysis: given that DAF is too large for cavity binding,‡‡The volume of DAF (*ca.* 300 Å^3^) is smaller than the cage cavity volume of *ca.* 400 Å^3^, but the anisotropic shape of DAF means that it is too long to fit inside the cage, with a maximum length of >14 Å. The maximum length that can be accommodated in the cavity along the long diagonal of the cube is *ca.* 8 Å. this must be at the cage external surface, and the acceleration induced by the cage is *ca.* 50-fold. In the other case the substrate 4NPA does bind inside the cavity, as solution NMR and solid-state X-ray crystallography measurements confirmed. However in this case not only is there no catalysis of the ester hydrolysis but the hydrolysis of the ester is actually inhibited by the cage – so the cage actually protects the substrate from reaction, with only residual unbound 4NPA undergoing weak surface-based catalysis. Together these experiments provide substantial additional insights into the mechanisms of catalytic processes that this cage can promote.

## Results and discussion

### Diacetylfluorescein (DAF) as substrate

**Scheme 1:**
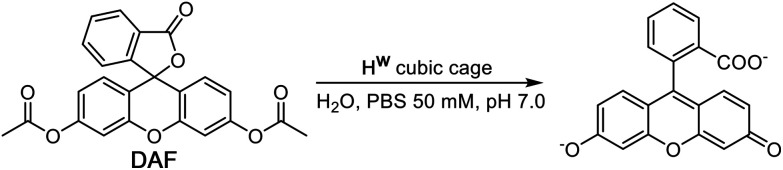
Hydrolysis of DAF to fluorescein.

**Fig. 2:**
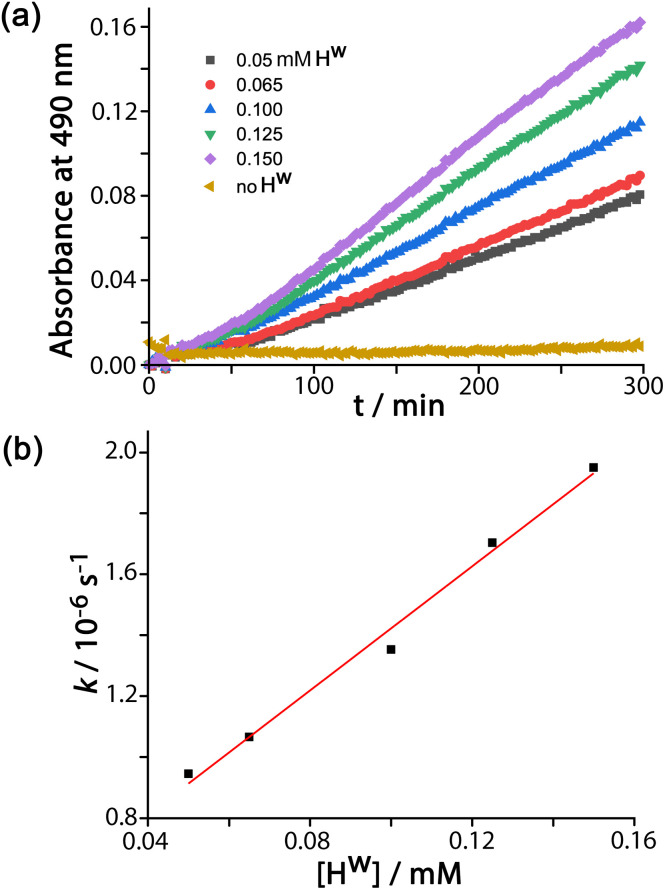
(a) Catalysed conversion of DAF to fluorescein at different concentrations of **H**^**w**^ as indicated and corrected for the background reaction: 65 μM DAF, 50 mM PBS, pH 7.0, 298 K. (b) Linear dependence of reaction rate constants on catalyst concentration from the data in (a) confirming first-order dependence on **H**^**w**^; the gradient gives a second-order rate constant *k*_2_ = 1.0 × 10^−2^ M^−1^ s^−1^.

**Fig. 3:**
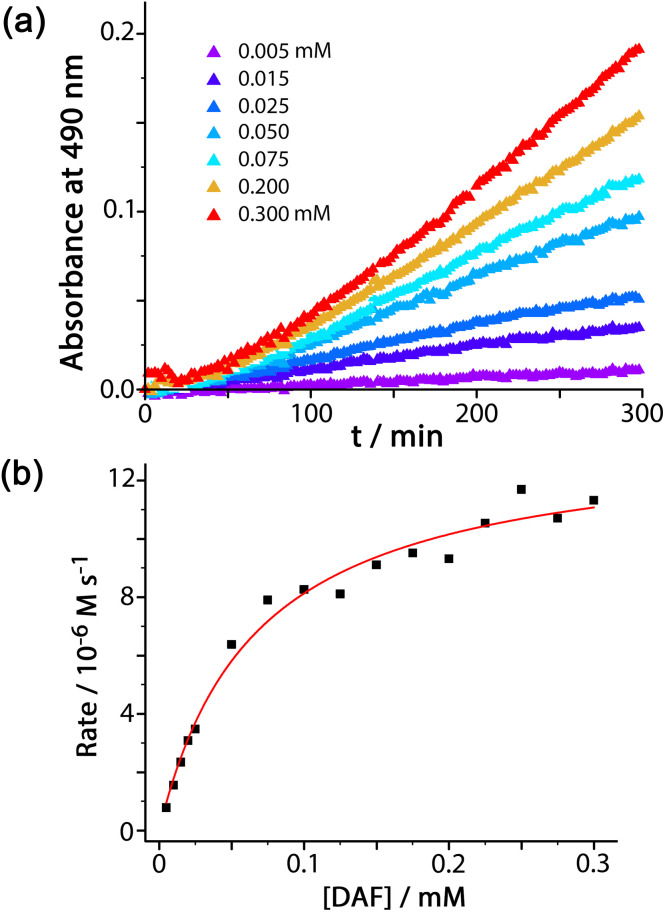
(a) Catalysed conversion of DAF to fluorescein at different concentrations of DAF, as indicated: 0.1 mM **H**^**w**^, 50 mM PBS, pH 7.0, 298 K. (b) Dependence of reaction rates on substrate concentration from the data in (a) fitted to a Michaelis–Menten reaction model (see main text).

DAF (see Scheme 1 for the hydrolysis reaction) is too large to bind in the cage cavity so any catalysed hydrolysis of the ester groups must necessary occur at the cage external surface. The hydrolysis of DAF to the fluorescein dianion (the dominant form at pH 7) is easy to follow by UV/Vis spectroscopy by monitoring the appearance of the strong fluorescein absorbance in the 400–500 nm range which extends to longer wavelengths than the absorbance of neutral DAF. In the presence of water-soluble cubic cage **H**^**w**^ (0.1 mM, as its chloride salt) the hydrolysis of DAF is clearly substantially accelerated (Fig. 2, 3) compared to the uncatalyzed background reaction (first-order rate constant 5.4 × 10^−8^ s^−1^ under the conditions used).

In all cases the reaction progress curves show some slight deviation from normal pseudo-first-order behaviour in the early stages. In particular the slight upwards curvature apparent in the early stages of the reaction are possibly indicative of some contribution from an autocatalytic pathway^3*d*,10^ during the initial phase when the relative increase in product concentration is most pronounced. However this becomes insignificant after about an hour under the conditions used and thereafter, in all cases, product formation follows standard pseudo-first-order behaviour as shown by linear ln[DAF] *vs.* time plots whose gradients gave the rate constants used in Fig. 2b. With a fixed concentration of DAF (65 μM) the use of different concentrations of **H**^**w**^ reveals a first-order dependence of reaction rate constant on catalyst concentration (Fig. 2b). This means that we can divide observed reaction rate constants by catalyst concentration (the gradient of the line in Fig. 2b) to obtain a second-order reaction rate constant of 1.0 × 10^−2^ M^−1^ s^−1^, which is comparable to what we observed for external-surface catalysed hydrolysis of some phospho-triester based insecticides under similar conditions.^8^

Control experiments with no **H**^**w**^ present, but using instead 0.1 mM CoCl_2_ or 0.1 mM L^peg^ (the PEG-ylated version of the ligand used in the cage, which is water soluble),^11^ showed no difference in the rate of DAF hydrolysis compared to the background reaction (the ‘no **H**^**w**^’ line in Fig. 2a), confirming that the assembled cage – with its combination of hydrophobic surface and high positive charge – is required to achieve the catalytic effect.

For experiments using increasing concentrations of DAF up to the solubility limit in water, as the substrate concentration increases the rise in reaction rate gets slower due to the expected effect of the catalyst surface sites becoming blocked (Fig. 3a). Fitting the rate *vs.* substrate concentration curve to a Michaelis–Menten model (Fig. 3b) yields a *K*_m_ value of 6.6(8) × 10^−5^ M, which implies – assuming a rapid equilibrium between ‘free’ and ‘bound’ states – a conventional 1 : 1 binding constant between **H**^**w**^ and DAF of 15 000 (±2000) M^−1^. This is in the range that we see for cavity-bound guests (10^3^–10^6^ M^−1^) where host interior surface and guest surface are well-matched.^3*a*^ Clearly the high hydrophobic surface area of DAF compensates for the lack of cavity binding and gives effective external-surface association with the cage. Actually this binding constant can be rationalised on the basis of the surface area of DAF, as the magnitude of the hydrophobic effect depends on the overlapping area of the hydrophobic surfaces that are in contact.^12,13^ We can identify from our previous work a cavity-bonding guest with a similar binding constant: cyclononanone binds with *K* = 11 000 (±3000) M^−1^ in water, and has a surface area of 183 Å^2^.^13^ As this is a cavity-binding guest we can assume that all of its surface is involved in making contact with the hydrophobic cavity interior, and this matching of hydrophobic surfaces substantially defines the binding constant. DAF has an essentially identical binding constant to **H**^**w**^ (15 000 ± 2000 M^−1^) but has a much larger surface area of 346 Å^2^. However as DAF is in contact with an *external* face of **H**^**w**^ we can estimate that only half of this surface area (173 Å^2^) contributes to the hydrophobic effect – which is very similar to the surface area of cyclononanone, hence a similar association constant.

The global rate increase under a particular set of conditions arises only from that fraction of DAF molecules that is in contact with the cage surface. Taking one set of conditions as an illustration (65 μM DAF, 0.1 mM **H**^**w**^, pH 7; data from Fig. 2) we can see that the first-order rate constant for product appearance in the presence of catalyst is 1.34 × 10^−6^ s^−1^ after correcting for the background reaction, whereas in the absence of catalyst it is 5.4 × 10^−8^ s^−1^. Hence, the rate due to **H**^**w**^ increases by a factor of 25 over the background reaction. From the concentrations of species in the reaction and the *K* value we find that the fraction of surface-bound guest in the equilibrium is 0.5. Accordingly the ratio *k*_cat_/*k*_uncat_, *i.e.* the extent to which the hydrolysis of a molecule of DAF in contact with the surface of **H**^**w**^ is accelerated compared to the uncatalysed reaction in bulk solution, is 25/0.5 ≈ 50. This may be compared with the *k*_cat_/*k*_uncat_ value of 2 × 10^5^ that we observed for the cage-catalysed Kemp elimination of benzisoxazole,^3*c*^ which benefited from a high binding constant for the guest inside the cavity where it was completely surrounded by surface-bound hydroxide ions. Raymond and co-workers have reported *k*_cat_/*k*_uncat_ values of millions for particularly effective catalysis inside cage cavities.^1*e*,*g*,4^

This large difference in the *k*_cat_/*k*_uncat_ ratios between cavity-bound benzisoxazole and surface-bound DAF is not, therefore, a consequence of the strength of binding of the substrate to the catalyst, but illustrates the importance of the *location* of substrate binding: binding at the exterior surface (partly in contact with the bulk solution) exposes the substrate to a lower local concentration of hydroxide ions around the substrate compared to cavity binding where the guest is completely surrounded. Accordingly there is a tradeoff between generality (external surface, as shown by DAF) and *k*_cat_/*k*_uncat_ ratio (cavity binding, as shown by benzisoxazole). Using the external surface for catalysis means that there are no binding limitations based on cavity shape and size constraints: but much broader generality for a range of substrates, albeit with less effectiveness, at the exterior surface.

### Effects of additional anion on cage-catalysed DAF hydrolysis

We were also interested to examine the effect of other anions on the progress of the reaction as these can compete with hydroxide for binding to the cage surface. Recent work on the Kemp elimination reaction with 5-nitrobenzisoxazole showed that the catalysed reaction occurred at the cage exterior surface, in contrast to unsubstituted benzisoxazole, which we ascribed to a less favourable orientation of the cavity-bound guest due to the steric effect of the nitro groups.^6*a*^ We also found in this work that the ability of different anions to inhibit the surface-catalysed reaction depended on their affinity for the cage surface, which in turn depended on a combination of their ease of desolvation (related to their position in the Hofmeister series) and their charge. Thus, the most weakly solvated anions such as nitrate and bromide showed the best inhibiting ability by binding most strongly to the cage surface and displacing the hydroxide that is necessary for the reaction. In contrast, weakly basic anions such as fluoride and hydrogencarbonate slightly *accelerated* the reaction, implying that the role of hydroxide in abstracting a proton from 5-nitrobenzisoxazole could also be filled by these anions if they preferentially accumulated around the cage.^6*a*^

**Fig. 4:**
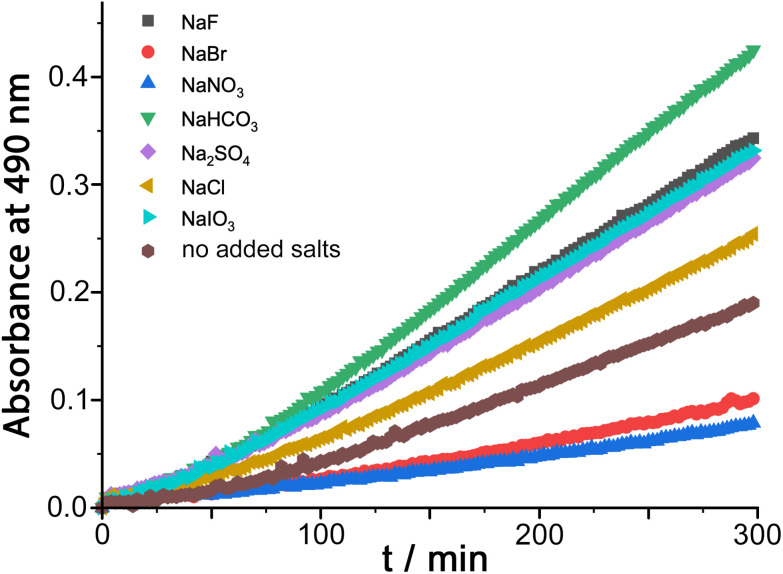
Effect of added ions (5 mM as sodium salts) on the progress of the **H**^**w**^-catalysed DAF hydrolysis (0.25 mM DAF, 0.1 mM **H**^**w**^, 50 mM PBS, pH 7.0, 298 K). These data are corrected for the uncatalyzed background reaction under the same conditions.

The results of similar experiments using DAF as substrate are shown in Fig. 4 (see also Table 1). Again we note the early-stage upwards curvature indicative of some autocatalysis^3*d*,10^ which becomes insignificant after about an hour, and the rate constants given in Table 1 are based on the later data collected after this period. The brown trace in Fig. 4 shows the reaction progress using **H**^**w**^ (fluoroborate salt) as catalyst with no added anions. On addition of 5 mM of other anions (as their sodium salts) the effect of the anions is, as before, to either accelerate or retard the reaction. Reassuringly, the order of reaction rate with added anion is precisely the same as previously observed using 5-nitrobenzisoxazole as substrate [HCO_3_^−^ > F^−^ > IO_3_^−^ > Cl^−^ > Br^−^ > NO_3_^−^], with bromide and nitrate having a particularly strong inhibitory effect because of the low cost of desolvating them such that they have a particularly high affinity for the cage surface.^6*a*^ Where these results differ from those previously observed, however, is that *only* bromide and nitrate act as inhibitors; all other anions accelerate the reaction (previously we observed that only fluoride and hydrogencarbonate accelerated the Kemp elimination due to their basicity). In contrast, for this ester hydrolysis reaction of DAF, chloride, sulfate and iodate anions also act to accelerate the reaction despite not being involved in performing nucleophilic attack on the ester group.

**Table 1:** Second-order rate constants for **H**^**w**^-catalysed hydrolysis of DAF in the presence of different concentrations of added sodium salts (0.25 mM DAF, 0.1 mM **H**^**w**^, 50 mM PBS, pH 7.0, 298 K) – derived from data plotted in Fig. 4

Entry	Added anion (as Na^+^ salt)	10^3^*k*_2_/M^−1^ s^−1^
a	None	6.1
b	F^−^	10.4
c	Cl^−^	7.9
d	Br^−^	3.0
e	NO_3_^−^	2.2
f	HCO_3_^−^	13.5
g	SO_4_^2−^	9.9
h	IO_3_^−^	10.1

We ascribe this to the fact that the fluorescein dianion, the reaction product, itself binds strongly to the cage surface as it is both dianionic and relatively hydrophobic.^6*b*^ We exploited this in previous work as the basis of a competitive displacement assay for evaluating binding of other anions according to how well they could displace fluorescein and restore its luminescence.^6*b*^ The effect of this will be to partly block the cage surface and retard the reaction by product inhibition. If binding of the fluorescein dianion to the cage has to compete with an added anion such as fluoride which occupies the windows in the cage surface, the ability of fluorescein to inhibit the reaction will be reduced and the reaction can accelerate.

This raises an obvious question: why is that effect not even stronger with bromide and nitrate? A solution to this is that if bromide and nitrate bind to the cage particularly strongly they will *also* displace hydroxide, at which point the catalysed hydrolysis stops. So we suggest that in the presence of anions such as fluoride and chloride the fluorescein dianion is preferentially displaced from the cage over hydroxide, preventing product inhibition; but more strongly binding anions such as bromide and nitrate *also* displace hydroxide and switch the catalysis off. We can easily deduce from this that hydroxide binds to the cage more strongly than the fluorescein dianion as it is more difficult to displace, which accounts for the remarkably effective reaction of cavity-bound benzisoxazole with the surrounding hydroxide ions.^3*c*^

### 4-Nitrophenyl-acetate (4NPA) as substrate

**Fig. 5:**
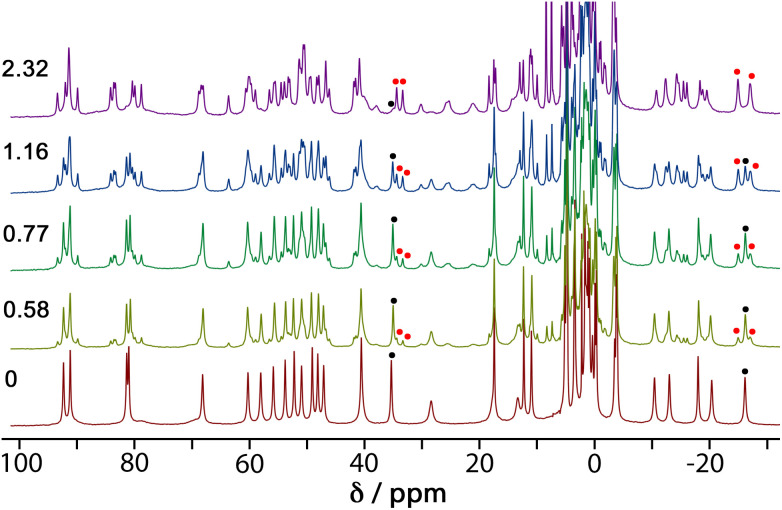
^1^H NMR spectra (300 MHz, D_2_O, 298 K) of mixtures of 1 mM **H**^**w**^ and 4NPA at varied concentrations (number of equivalents of added 4NPA shown on the left). For the signals around −27 and +35 ppm, the black dots indicate the original signal from empty **H**^**w**^; the red dots indicate the new signals from the **H**^**w**^/4NPA complex.

4NPA is a convenient substrate to test for cage-catalysed reactivity for the same reason as DAF, because the hydrolysis reaction liberates a coloured anion (4-nitrophenolate; *λ*_max_ ≈ 400 nm) whose accumulation can be followed colorimetically using UV/Vis spectroscopy. Binding of this substrate inside the cage cavity was demonstrated by a ^1^H NMR spectroscopic titration in water. A titration in which portions of the guest were added to a 1.0 mM solution of **H**^**w**^ resulted in the paramagnetically-shifted ^1^H NMR spectrum becoming more complex, with splitting of signals associated with the empty cage occurring due to guest binding in slow exchange (Fig. 5).

We see that one proton signal for the empty cage is replaced with multiple smaller signals on guest binding, which implies desymmetrisation of the *S*_6_-symmetric cage: *i.e.* the motion of the guest inside the cavity is also slow on the NMR timescale – in addition to the in/out exchange – and there is no averaging of the guest orientation from tumbling inside the cage. The signals corresponding to cage/guest complex and free cage are carefully integrated to obtain the [HG]/[H] ratio, and averaging many such measurements using different signals across spectra affords a binding constant of 3.5(3) × 10^3^ M^−1^. We note that this is comparable to the binding constant of guests such as cyclooctanone [2.1(5) × 10^3^ M^−1^] and bicyclo[3.3.1]nonan-3-one [*K* = 4.00(4) × 10^3^ M^−1^] which have similar hydrophobic surface areas.^13^ We assume that this binding constant of 3.5(3) × 10^3^ M^−1^ represents 1 : 1 cage guest binding: although in the solid state we can sometimes see (*vide infra*) two guest molecules stacked together inside the cavity of this type of cage, this is under non-equilibrium forcing conditions (*i.e.* a crystal soaked in a large excess of guest). In solution we expect that the second guest binding constant *K*_2_ is much less than the first one *K*_1_ such that the 1 : 1 cage : guest complex dominates the solution speciation.^14^

**Fig. 6:**
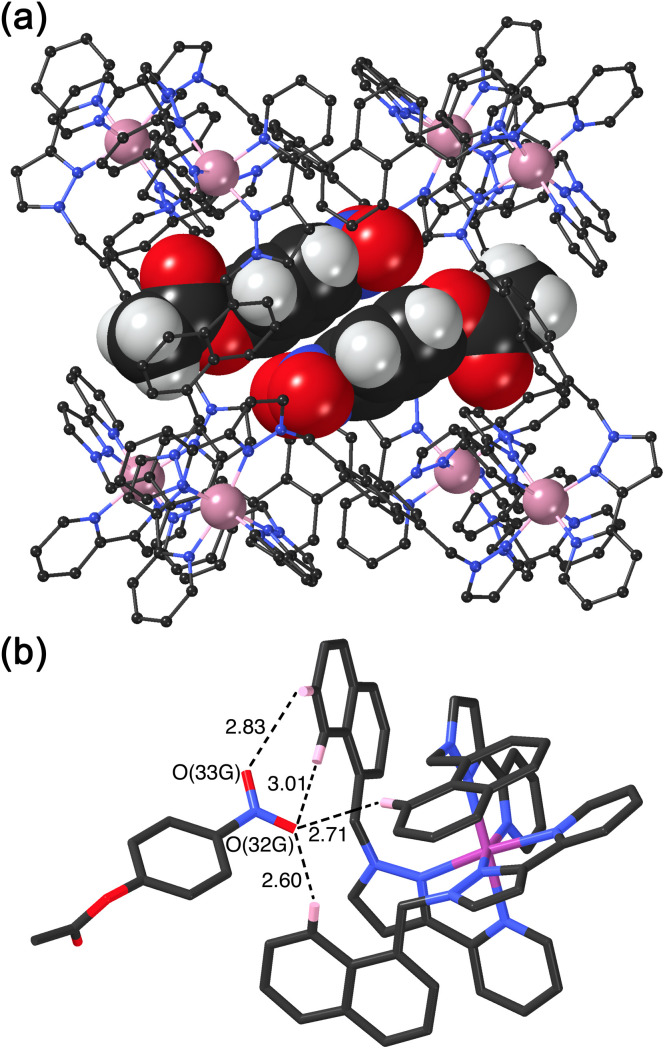
Crystal structure of the host/guest complex **H**^**Ni**^/NPA. (a) A view of the cage complex cation showing the stacked pair of crystallographically equivalent encapsulated NPA guests in the cavity which lie across an inversion centre, with unit site occupancy each; (b) a view of the hydrogen-bonding environment around each guest showing in particular the CH⋯N/CH⋯O contacts (distances shown in Å) between the nitro group of the NPA and the cage interior surface close to a Ni(ii) cation.

We obtained a crystal structure of the cage/guest complex using unsubstituted cage **H**^**Ni**^ as the host, containing Ni(ii) ions at the vertices rather than the more usual Co(ii) ions – we have found that these diffract better due to lower X-ray absorption issues, but the Ni(ii) and Co(ii) versions of **H** are isostructural. The sample was prepared using the crystalline sponge technique that we have reported before,^14^ involving immersing pre-formed crystals of **H**^**Ni**^ into a concentrated solution of 4NPA for several hours, resulting in guest molecules being taken up into the cage cavity with retention of crystallinity. We observe, as we have done with a range of small aromatic guests, the presence of a stacked pair of guests lying across the inversion centre which is at the centre of the cage (Fig. 6a).^14^ The two phenyl rings are parallel and offset with a separation of 3.60 Å between the two planes. Each guest is oriented such that its nitro substituent projects into the pocket close to a *fac* tris-chelate vertex where multiple C–H protons converge, resulting in a collection of CH⋯O hydrogen-bonding interactions that are assisted by the increased *δ*+ on the H atoms arising from positive charge of the nearby Ni(ii) centre (Fig. 6b).

This type of hydrogen-bonding between the array of CH donors at the internal surface around the two *fac* tris-chelate vertices, and the H-bond acceptor parts of polar organic guests, is a recurrent feature of the crystal structures of all cage/guest complexes that we have structurally characterised with this host family.^15^

**Fig. 7:**
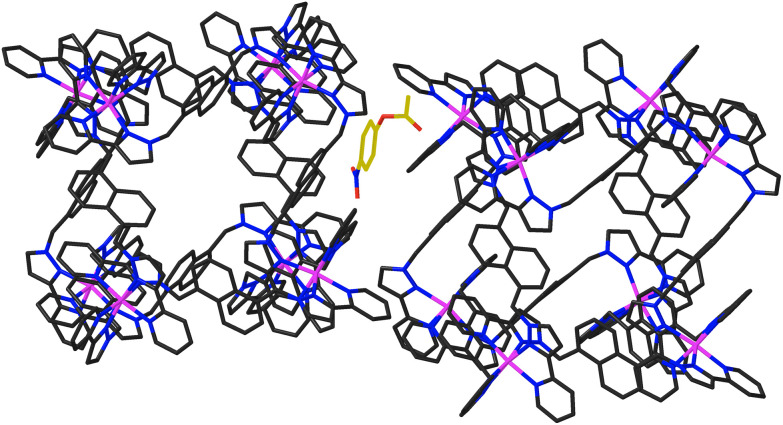
Location of an additional 4NPA guest molecule (0.3 site occupancy, highlighted using a gold colour for the C atoms) in the space between two adjacent **H**^**Ni**^ complex units, interacting with the cage exterior surfaces.

In addition to the two 4NPA guests that form a stacked pair in the cavity with 100% site occupancy, there is an additional exterior molecule of 4NPA (0.3 site occupancy per complete cage) which lies sandwiched in the space between two adjacent cages (Fig. 7). This is oriented such that the acetate group lies close to the cationic vertex of one cage and the nitro group lies over the face of the other. There is no point in over-analysing the large number of weak CH⋯O hydrogen-bonds that are involved, but observation of this ‘external’ guest is relevant to the discussion about external surface catalysis below, and we note that we have seen other examples of guests that lie in the space between cubic cage molecules in the crystal, interacting with the cage external surfaces.^16^

**Fig. 8:**
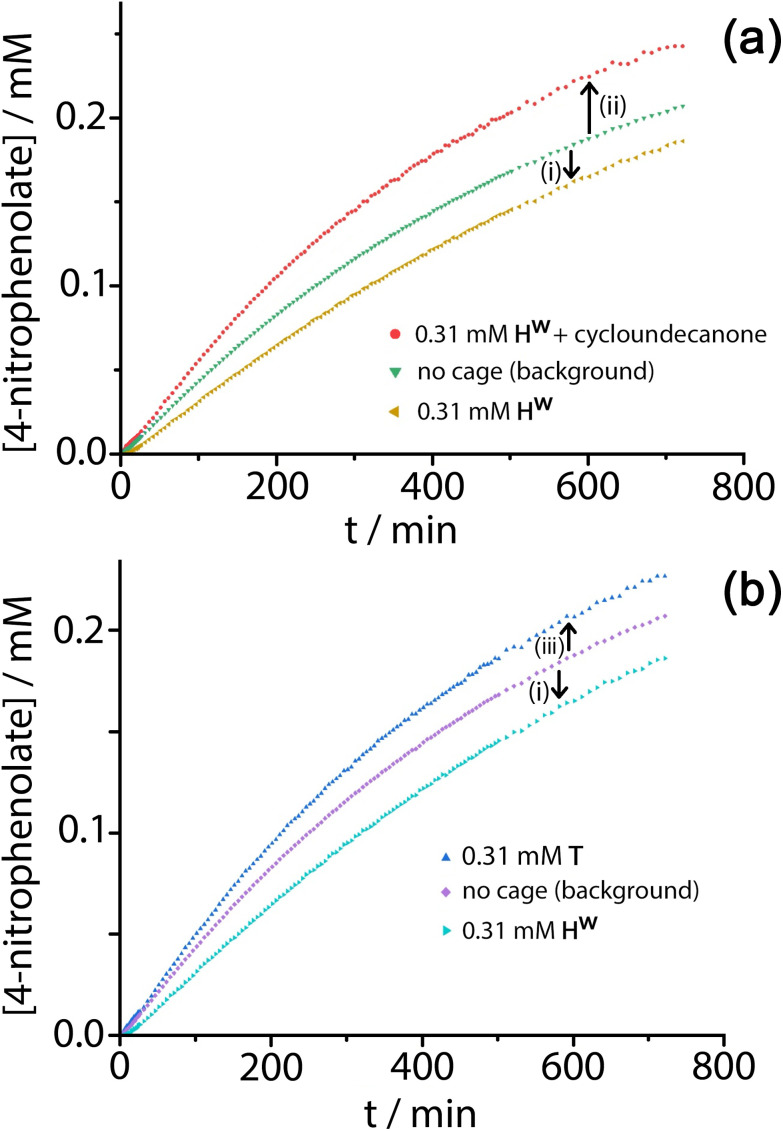
Contributions of **H**^**w**^ and **T** to positive and negative catalysis of 4NPA hydrolysis (0.3 mM 4NPA, pH 7.4, 303 K). Panel (a) shows the retardation effect (negative catalysis) of **H**^**w**^ on 4NPA hydrolysis [arrow (i)] associated with cavity binding, and the small acceleration [arrow (ii)] associated with external surface based catalysis when the cavity is blocked using cycloundecanone. Panel (b) shows the contrasting effects of **H**^**w**^ [arrow (i), as before] and **T** [arrow (iii)] in which no cavity binding of the 4NPA is possible and only external-surface catalysis can occur.

Given the obvious inclusion of the 4NPA in the cage cavity, and the ability of the cage to accumulate HO^−^ ions around the surface,^3*a*–*c*^ we were interested to examine this system for catalysed ester hydrolysis. The hydrolysis of 4NPA has a much higher background rate constant than the hydrolysis of DAF (first-order rate constant 2.5 × 10^−5^ s^−1^) under the conditions used, a consequence in part of the Hammett parameter associated with the *p*-nitro group. We found however that the hydrolysis of 4NPA was consistently slightly *retarded* in the presence of **H**^**w**^, *i.e.* negative catalysis is occurring. Fig. 8a shows how the reaction progress for hydrolysis of 4NPA at pH 7.4 is slightly slowed down (*ca.* 10%) in the presence of 0.3 mM **H**^**w**^. Addition of a strongly cavity-binding inhibitor (cyclo-undecanone, *K* ≈ 10^6^ M^−1^ for cavity binding),^12^ which blocks uptake of 4NPA, removes this inhibition and actually causes a small rate acceleration compared to the background reaction with catalyst absent.

This implies two things: firstly that cavity-bound 4NPA is not reacting with the surrounding shell of hydroxide ions, such that the surrounding cage is actually acting as a protecting group; and secondly that, when released from the cavity, 4NPA undergoes weak catalysis at the external surface. The lack of reactivity of cavity-bound 4NPA could be due either to an unfavourable orientation of 4NPA in the cavity which renders attack of hydroxides on the ester group impossible, or to the fact that the tetrahedral intermediate which would form following initial attack by hydroxide ions is destabilised in the confined space so its formation is prevented. Nitschke and co-workers famously demonstrated how stabilisation of P_4_ molecules in the cavity of a tetrahedral coordination cage, in air, arose not because molecules of O_2_ are excluded from the cavity: but because there is no room in the cavity for the necessary expansion in volume of the guest associated with formation of the reaction intermediates.^17^

Under the conditions of the reaction, and knowing the binding constant of 3500 M^−1^, we can calculate that for the experiment in Fig. 8, with 0.3 mM 4NPA and 0.31 mM **H**^**w**^, *ca.* 40% of the substrate is bound at any one time: yet the rate of appearance of product is slowed by only 10% [arrow labelled (i) in Fig. 8a and b]. If we assume that the cavity-bound guests are not significantly reactive, we conclude that the free 4NPA is undergoing some catalysed hydrolysis at the cage exterior surface, and this effect is contrary to the cavity-based inhibition. Further confirmation of this is provided by an additional control experiment using the tetrahedral Co_4_L_6_ cage **T** (see Fig. 1 for structure) as a catalyst. Cage **T** has a similar hydrophobic external surface to **H**^**w**^ but is about half the size and charge: the key point here is that its central cavity is far too small to accommodate 4NPA so inhibition by cavity binding is not possible.^8^ We see (Fig. 8b) that whereas 0.3 mM **H**^**w**^ overall slightly retards hydrolysis of 4NPA [arrow labelled (i)] because the deactivating cavity-binding effect is outweighs the effect of surface-based catalysis for the free 4NPA, the same concentration of **T** (no cavity binding) results in a slight increase in hydrolysis [arrow labelled (iii)]. The catalytic effect of **T** is clearly very small (<10% increase in overall conversion), likely because of a high background rate for hydrolysis of 4NPA as mentioned earlier: but the key point is that the two contrasting effects, in opposite directions, arising from cavity-binding and surface-binding are quite clear.

The rate increase due to external surface catalysis of the 4NPA hydrolysis by **H**^**w**^ is accordingly given by the arrow labelled (ii) in Fig. 8a, which is the difference between the green curve (background reaction for hydrolysis of 4NPA) and the red curve (catalysis in the presence of **H**^**w**^ but with the cavity blocked by cycloundecanone). The measured initial rates for product appearance increase from 7.5 × 10^−9^ M s^−1^ (background, Fig. 8a, green curve) to 9.6 × 10^−9^ M s^−1^ (catalyst with cavity blocked, Fig. 8a, red curve), which gives a second-order rate constant *k*_2_ of 0.023 M^−1^ s^−1^, of the same order as we observed for catalysed DAF hydrolysis (*k*_2_ ≈ 10^−2^ M^−1^ s^−1^).

This *k*_2_ value for catalysed 4NPA hydrolysis arises from a combination of (i) the inherent effect of the cage on reactivity of bound substrate, and (ii) the equilibrium constant for formation of the **H**^**w**^/4NPA complex, which is likely to be much smaller than observed for the **H**^**w**^/DAF complex because of the smaller hydrophobic surface area of 4NPA.^12,13^ If we make the crude approximation that the *k*_cat_/*k*_uncat_ ratio for hydrolysis of 4NPA (*i.e.* the effect of the cage on a surface-bound substrate) is comparable to that of DAF and set this (see discussion above) at 50, then the 1.3-fold rate acceleration that we observe for hydrolysis of 4NPA requires just 0.6% of it to be surface-bound – giving an equilibrium constant for **H**^**w**^/4NPA external surface association of the order of 10^1^ M^−1^, three orders of magnitude less than for DAF. This is to be expected on the basis of the lower hydrophobicity of 4NPA, and suggests that the similarity of the two *k*_2_ values (both ≈10^−2^ M^−1^ s^−1^) occurs because the much higher inherent reactivity of 4NPA is counterbalanced by its much weaker association with the catalyst surface. We note that a direct measure of *K* using the same type of analysis as shown in Fig. 3b is not possible given such weak binding, and the figures above are a crude estimate, but the general picture is clear.

## Conclusions

In conclusion, the similarities and differences between the behaviour of two different esters DAF and 4NPA as they undergo cage-catalysed hydrolysis with **H**^**w**^ provide useful insights into the different factors at play and how to extend and optimise catalytic behaviour in the future. DAF is too large to bind inside the cavity of **H**^**w**^ but its hydrolysis is effectively catalysed by the cage exterior surface, which acts to co-locate both the hydrophobic substrate and hydroxide ions in the same region. We can estimate a *k*_cat_/*k*_uncat_ ratio of *ca.* 50 for the effect of the cage, which is assisted by a strong **H**^**w**^/DAF interaction (15 000 M^−1^) – which is actually comparable to what we observe for many cavity-binding guests and is a consequence of the high hydrophobic surface area of DAF. Clearly, lack of cavity-based binding need not be a problem for achieving good binding of a substrate to the cage at the exterior surface, though *k*_cat_/*k*_uncat_ suffers because the substrate is not completely surrounded by the partly desolvated, surface-bound hydroxide ions.

In contrast, the substrate 4NPA is of a shape and size to bind strongly inside **H**^**w**^, and does so with *K* = 3500 M^−1^. However hydrolysis of 4NPA is actually inhibited by cavity binding. This could be either because it binds in a way that makes it inaccessible to surface-bound hydroxide ions, or because the cage cannot accommodate the tetrahedral intermediate following the first step in the usual hydrolysis mechanism. Any 4NPA that is unbound as part of the normal solution equilibrium still undergoes catalysed hydrolysis at the cage exterior surface, although the small hydrophobic surface area of 4NPA means that it binds to the **H**^**w**^ surface much more weakly than does DAF which limits the catalytic effect.

Overall: despite the outstanding results that we obtained earlier in one case (Kemp elimination of benzisoxazole),^3*c*^ cavity-based guest binding does not always lead to catalysis – substrate reactivity can actually be decreased, with the cage effectively acting as a protecting group, preventing the ester hydrolysis of 4NPA. In contrast catalysed reactions at the cage exterior surface can occur due to significant rate accelerations (*k*_cat_/*k*_uncat_ ratio of *ca.* 50 for DAF) and the substrate having a reasonable affinity for the cage surface because of its hydrophobicity.

The possible benefits of this for supramolecular catalysis of reactions between neutral hydrophobic substrates, and anions, will be the subject of further investigations with cages of different shapes and sizes, focussing as much on the cage external surface as the central cavity. We suggest that the use of the cage external surface in this way combines attributes of both heterogeneous and homogeneous catalysis, with the binding of the two reaction partners to the surface being reminiscent of surface-based heterogeneous catalysis, but with the benefits of solubility and solution processability associated with homogeneous catalysis.^18^

## Experimental

### General details

The cages **H**^**w**^ and **H**^**Ni**^ were prepared and crystallised as previously described,^19^ with the small difference that unsubstituted host cage **H** was prepared as the Ni(ii) complex rather than the original Co(ii) complex; in all other respects the synthesis is unchanged.^19*a*^ Tetrahedral Co_4_ cage **T** was likewise prepared as previously reported.^8^ Substrates 4NDP and DAF were purchased from Sigma-Aldrich and Merck, respectively, and used as received. ^1^H NMR spectra for the binding constant of 4NPA were recorded in D_2_O on a Bruker AV-300 instrument at 298 K. The catalysis studies (see below) were performed using a BMG Clariostar plate reader.

### Catalysis studies

The catalysis studies were carried out in water in total volumes of 200 μL in each well of a standard 96-well plate, by mixing the appropriate amounts of **H**^**w**^, substrate (25 mM DAF solution in acetone or 4NPA), and PBS (phosphate buffer) stock solutions to reach the required concentrations of cage and substrate. With DAF as substrate the conditions were 50 mM PBS, pH 7.0, 298 K. With 4NPA as substrate the conditions were 100 mM PBS, pH 7.4, 303 K. The reactions were monitored over the course of several hours, by following the UV/Vis absorption of the products [with DAF as substrate, the product is fluorescein with *λ*_max_ 490 nm (*ε* = 80 000 M^−1^ cm^−1^); with 4NPA as substrate, the product is 4-nitrophenolate with *λ*_max_ 400 nm (*ε* = 12 800 M^−1^ cm^−1^)]. Each dataset is the average of four measurements and is corrected for the uncatalyzed background reaction, unless specified otherwise. Key data associated with effects of added anions are summarised in Table 1; the ESI† contains all of the data used for the rate and rate constant calculations.

### X-ray crystallography

The X-ray crystallographic data collection for the **H**^**Ni**^·4NPA host/guest complex were collected on a Rigaku Supernova four-circle diffractometer with an Atlas-S2 CCD detector. Data collection, integration and absorption correction were performed using the CrysAlisPro software;^20^ structure solution and refinement used the OLEX2 software.^21^ Diffuse electron density associated with regions of disordered solvent was removed from the refinement using the ‘solvent mask’ function (SQUEEZE); full details are in the CIF (CCDC deposition number 2175256†). Information on the crystal properties, data collection and refinement parameters associated with this structure determination is collected in Table 2.

**Table 2:** Crystal parameters, data collection and refinement details for the structure of the HNi/NPA complex

Complex	H^Ni^·NPA_2.3_·13MeOH
Formula	C_367.4_H_332.1_B_16_F_64_N_74.3_Ni_8_O_22.2_
Molecular weight	8001.99
*T*/K	150(1)
Radiation wavelength/Å	Cu-Kα (1.54184)
Crystal system	Monoclinic
Space group	*P*2_1_/*c*
*a*/Å	31.4788(5)
*b*/Å	30.5879(5)
*c*/Å	39.9794(7)
*β*/°	100.927(2)
*V*/Å^3^	37 797.0(11)
*Z*	4
*ρ*/g cm^−3^	1.406
Crystal size/mm^3^	0.18 × 0.13 × 0.11
*μ*/mm^−1^	1.307
Data, restraints, parameters	63 718, 21 834, 4633
*R*_int_, *R*_sigma_	0.0496, 0.0693
Final *R*_1_, w*R*_2_^a^	0.1191, 0.4049
Largest diff. peak/hole/e Å^−3^	1.17/−0.43

a The value of *R*_1_ is based on ‘observed’ data with *I* > 2σ(*I*); the value of w*R*_2_ is based on all data.

## Author contributions

A. B. S.: all work using DAF as substrate. B. S.: all work using 4NPA as substrate. C. G. P. T.: crystallographic analysis of the **H**^**Ni**^/4NPA complex. M. D. W.: project conception, supervision, and manuscript preparation.

## Conflicts of interest

There are no conflicts to declare.

## Supplementary Material

DT-051-D2DT01713J-s001

DT-051-D2DT01713J-s002
