# Supplementary material for: Inside or outside the box? Effect of substrate location on coordination-cage based catalysis
Source: Dalton Trans. 2022 Jun 30;51(30):11277–85. doi: 10.1039/d2dt01713j (PMC9344580; doi:10.1039/d2dt01713j)

Electronic Supplementary Material (ESI) for Dalton Transactions.
This journal is © The Royal Society of Chemistry 2022

Inside or outside the box? Effect of substrate location on coordination-cage based catalysis

Atena B. Solea, Burin Sudittapong, Christopher G. P. Taylor and Michael D. Ward*

Supporting information: data for catalysis studies using diacetyl-fluorescein as substrate

Contents

Catalysis of **DAF** to **FLU** with 0.1 mM **CoH^{w}Cl** added (50 mM PBS pH 7) at various DAF concentrations (0.005-0.300 mM) 2

Catalysis of **DAF** to **FLU** with concentrations of **CoH^{w}Cl** between 0.05-0.150 mM ([**DAF**]=0.065 mM , 50 mM PBS buffer pH 7) 71

Catalysis of **DAF** to **FLU** in the presence of **CoHwBF$_4$** and various anions (0.25 mM **DAF**, 5 mM anion, 50 mM pH 7 **PBS** buffer, 0.1 mM **CoH^{w}BF$_4$**) 91

Catalysis of **DAF** to **FLU** with 0.1 mM **CoH^wCl** added (50 mM PBS pH 7) at various DAF concentrations (0.005-0.300 mM)

DAF varied for 0.1mM CoHwCl

0.005 mM **DAF**

Time (min)	A@490nm minus BK	set to 0	[FLU] (M)	[DAF] (M)	ln[DAF]
0	0.024	0	0	0.000005	-12.2061
2	0.022	-0.00225	-2.8E-08	5.03E-06	-12.2005
4	0.02225	-0.00175	-2.2E-08	5.02E-06	-12.2017
6	0.02275	-0.00375	-4.7E-08	5.05E-06	-12.1967
8	0.0235	-0.001	-1.3E-08	5.01E-06	-12.2036
10	0.023	-0.0015	-1.9E-08	5.02E-06	-12.2023
12	0.02225	-0.0015	-1.9E-08	5.02E-06	-12.2023
14	0.022	-0.00325	-4.1E-08	5.04E-06	-12.198
16	0.02275	-0.00125	-1.6E-08	5.02E-06	-12.203
18	0.0225	-0.00175	-2.2E-08	5.02E-06	-12.2017
20	0.02375	-7.50E-04	-9.4E-09	5.01E-06	-12.2042
22	0.0225	-0.00325	-4.1E-08	5.04E-06	-12.198
24	0.02275	-0.002	-2.5E-08	5.03E-06	-12.2011
26	0.02225	-0.00225	-2.8E-08	5.03E-06	-12.2005
28	0.02275	-0.0015	-1.9E-08	5.02E-06	-12.2023
30	0.02325	-2.50E-04	-3.1E-09	5E-06	-12.2054
32	0.02325	-7.50E-04	-9.4E-09	5.01E-06	-12.2042
34	0.02275	-0.00125	-1.6E-08	5.02E-06	-12.203
36	0.02275	-0.00175	-2.2E-08	5.02E-06	-12.2017
38	0.02325	-7.50E-04	-9.4E-09	5.01E-06	-12.2042
40	0.023	-0.00125	-1.6E-08	5.02E-06	-12.203
42	0.02375	-2.50E-04	-3.1E-09	5E-06	-12.2054
44	0.0235	-2.50E-04	-3.1E-09	5E-06	-12.2054
46	0.02325	-0.00175	-2.2E-08	5.02E-06	-12.2017
48	0.02425	2.50E-04	3.13E-09	5E-06	-12.2067
50	0.025	0.0015	1.88E-08	4.98E-06	-12.2098
52	0.023	0	0	0.000005	-12.2061
54	0.02425	5.00E-04	6.25E-09	4.99E-06	-12.2073
56	0.0245	0.00125	1.56E-08	4.98E-06	-12.2092
58	0.02425	-2.50E-04	-3.1E-09	5E-06	-12.2054
60	0.024	5.00E-04	6.25E-09	4.99E-06	-12.2073
62	0.0245	2.50E-04	3.13E-09	5E-06	-12.2067
64	0.0245	5.00E-04	6.25E-09	4.99E-06	-12.2073
66	0.02475	5.00E-04	6.25E-09	4.99E-06	-12.2073
68	0.02475	0	0	0.000005	-12.2061
70	0.02575	0.00175	2.19E-08	4.98E-06	-12.2105
72	0.0245	0	0	0.000005	-12.2061
74	0.0245	5.00E-04	6.25E-09	4.99E-06	-12.2073

76	0.025	5.00E-04	6.25E-09	4.99E-06	-12.2073
78	0.02525	0.00225	2.81E-08	4.97E-06	-12.2117
80	0.025	7.50E-04	9.38E-09	4.99E-06	-12.2079
82	0.02525	0.002	2.5E-08	4.98E-06	-12.2111
84	0.024	-7.50E-04	-9.4E-09	5.01E-06	-12.2042
86	0.02575	0.001	1.25E-08	4.99E-06	-12.2086
88	0.02575	0.00175	2.19E-08	4.98E-06	-12.2105
90	0.02575	0.002	2.5E-08	4.98E-06	-12.2111
92	0.0255	0.00125	1.56E-08	4.98E-06	-12.2092
94	0.0255	0.00175	2.19E-08	4.98E-06	-12.2105
96	0.0265	0.00275	3.44E-08	4.97E-06	-12.213
98	0.027	0.00225	2.81E-08	4.97E-06	-12.2117
100	0.026	0.00275	3.44E-08	4.97E-06	-12.213
102	0.02625	0.0015	1.88E-08	4.98E-06	-12.2098
104	0.02625	0.0025	3.13E-08	4.97E-06	-12.2123
106	0.02625	0.003	3.75E-08	4.96E-06	-12.2136
108	0.0265	0.003	3.75E-08	4.96E-06	-12.2136
110	0.0265	0.00175	2.19E-08	4.98E-06	-12.2105
112	0.02725	0.0035	4.38E-08	4.96E-06	-12.2149
114	0.0265	0.0025	3.13E-08	4.97E-06	-12.2123
116	0.027	0.00225	2.81E-08	4.97E-06	-12.2117
118	0.02675	0.0025	3.13E-08	4.97E-06	-12.2123
120	0.027	0.003	3.75E-08	4.96E-06	-12.2136
122	0.02725	0.00275	3.44E-08	4.97E-06	-12.213
124	0.02775	0.004	5E-08	4.95E-06	-12.2161
126	0.02875	0.00425	5.31E-08	4.95E-06	-12.2168
128	0.02775	0.0035	4.38E-08	4.96E-06	-12.2149
130	0.02875	0.004	5E-08	4.95E-06	-12.2161
132	0.028	0.0045	5.63E-08	4.94E-06	-12.2174
134	0.02875	0.00425	5.31E-08	4.95E-06	-12.2168
136	0.02775	0.003	3.75E-08	4.96E-06	-12.2136
138	0.02925	0.0089	1.11E-07	4.89E-06	-12.2286
140	0.02825	0.004	5E-08	4.95E-06	-12.2161
142	0.029	0.004	5E-08	4.95E-06	-12.2161
144	0.02925	0.0045	5.63E-08	4.94E-06	-12.2174
146	0.0285	0.00325	4.06E-08	4.96E-06	-12.2142
148	0.02875	0.00375	4.69E-08	4.95E-06	-12.2155
150	0.0285	0.00325	4.06E-08	4.96E-06	-12.2142
152	0.02975	0.005	6.25E-08	4.94E-06	-12.2187
154	0.02975	0.005	6.25E-08	4.94E-06	-12.2187
156	0.029	0.0045	5.63E-08	4.94E-06	-12.2174
158	0.02975	0.00475	5.94E-08	4.94E-06	-12.218
160	0.0295	0.00425	5.31E-08	4.95E-06	-12.2168
162	0.029	0.00375	4.69E-08	4.95E-06	-12.2155
164	0.03025	0.00525	6.56E-08	4.93E-06	-12.2193
166	0.03	0.0055	6.88E-08	4.93E-06	-12.2199

168	0.03075	0.00575	7.19E-08	4.93E-06	-12.2206
170	0.03025	0.00525	6.56E-08	4.93E-06	-12.2193
172	0.02975	0.005	6.25E-08	4.94E-06	-12.2187
174	0.03075	0.006	7.5E-08	4.93E-06	-12.2212
176	0.03075	0.00475	5.94E-08	4.94E-06	-12.218
178	0.031	0.00625	7.81E-08	4.92E-06	-12.2218
180	0.0285	0.00425	5.31E-08	4.95E-06	-12.2168
182	0.03075	0.0055	6.88E-08	4.93E-06	-12.2199
184	0.03075	0.00575	7.19E-08	4.93E-06	-12.2206
186	0.03025	0.005	6.25E-08	4.94E-06	-12.2187
188	0.031	0.006	7.5E-08	4.93E-06	-12.2212
190	0.0305	0.00625	7.81E-08	4.92E-06	-12.2218
192	0.0315	0.00625	7.81E-08	4.92E-06	-12.2218
194	0.03125	0.006	7.5E-08	4.93E-06	-12.2212
196	0.03125	0.00675	8.44E-08	4.92E-06	-12.2231
198	0.03225	0.00825	1.03E-07	4.9E-06	-12.2269
200	0.032	0.00675	8.44E-08	4.92E-06	-12.2231
202	0.032	0.0075	9.38E-08	4.91E-06	-12.225
204	0.03225	0.00775	9.69E-08	4.9E-06	-12.2256
206	0.03225	0.00775	9.69E-08	4.9E-06	-12.2256
208	0.0315	0.007	8.75E-08	4.91E-06	-12.2237
210	0.03225	0.0075	9.38E-08	4.91E-06	-12.225
212	0.032	0.00675	8.44E-08	4.92E-06	-12.2231
214	0.03275	0.0075	9.38E-08	4.91E-06	-12.225
216	0.0325	0.00625	7.81E-08	4.92E-06	-12.2218
218	0.03175	0.006	7.5E-08	4.93E-06	-12.2212
220	0.03325	0.00825	1.03E-07	4.9E-06	-12.2269
222	0.033	0.00775	9.69E-08	4.9E-06	-12.2256
224	0.03275	0.008	1E-07	4.9E-06	-12.2263
226	0.0335	0.00775	9.69E-08	4.9E-06	-12.2256
228	0.033	0.0075	9.38E-08	4.91E-06	-12.225
230	0.03375	0.00875	1.09E-07	4.89E-06	-12.2282
232	0.0335	0.008	1E-07	4.9E-06	-12.2263
234	0.03275	0.0065	8.13E-08	4.92E-06	-12.2225
236	0.033	0.0085	1.06E-07	4.89E-06	-12.2276
238	0.03275	0.00725	9.06E-08	4.91E-06	-12.2244
240	0.033	0.00725	9.06E-08	4.91E-06	-12.2244
242	0.03375	0.00825	1.03E-07	4.9E-06	-12.2269
244	0.03325	0.00775	9.69E-08	4.9E-06	-12.2256
246	0.03375	0.00825	1.03E-07	4.9E-06	-12.2269
248	0.03425	0.00875	1.09E-07	4.89E-06	-12.2282
250	0.03475	0.00975	1.22E-07	4.88E-06	-12.2307
252	0.035	0.00925	1.16E-07	4.88E-06	-12.2295
254	0.03425	0.0085	1.06E-07	4.89E-06	-12.2276
256	0.03475	0.0085	1.06E-07	4.89E-06	-12.2276
258	0.03475	0.0085	1.06E-07	4.89E-06	-12.2276

260	0.03375	0.00725	9.06E-08	4.91E-06	-12.2244
262	0.0345	0.00775	9.69E-08	4.9E-06	-12.2256
264	0.0355	0.00925	1.16E-07	4.88E-06	-12.2295
266	0.03375	0.00875	1.09E-07	4.89E-06	-12.2282
268	0.035	0.00875	1.09E-07	4.89E-06	-12.2282
270	0.03525	0.0095	1.19E-07	4.88E-06	-12.2301
272	0.035	0.00825	1.03E-07	4.9E-06	-12.2269
274	0.0355	0.0085	1.06E-07	4.89E-06	-12.2276
276	0.035	0.00825	1.03E-07	4.9E-06	-12.2269
278	0.03575	0.0095	1.19E-07	4.88E-06	-12.2301
280	0.03625	0.00975	1.22E-07	4.88E-06	-12.2307
282	0.03725	0.0115	1.44E-07	4.86E-06	-12.2352
284	0.0365	0.00975	1.22E-07	4.88E-06	-12.2307
286	0.03575	0.00975	1.22E-07	4.88E-06	-12.2307
288	0.03625	0.009	1.13E-07	4.89E-06	-12.2288
290	0.03725	0.0115	1.44E-07	4.86E-06	-12.2352
292	0.03625	0.0105	1.31E-07	4.87E-06	-12.2327
294	0.037	0.011	1.38E-07	4.86E-06	-12.234
296	0.03625	0.0105	1.31E-07	4.87E-06	-12.2327
298	0.03775	0.011	1.38E-07	4.86E-06	-12.234

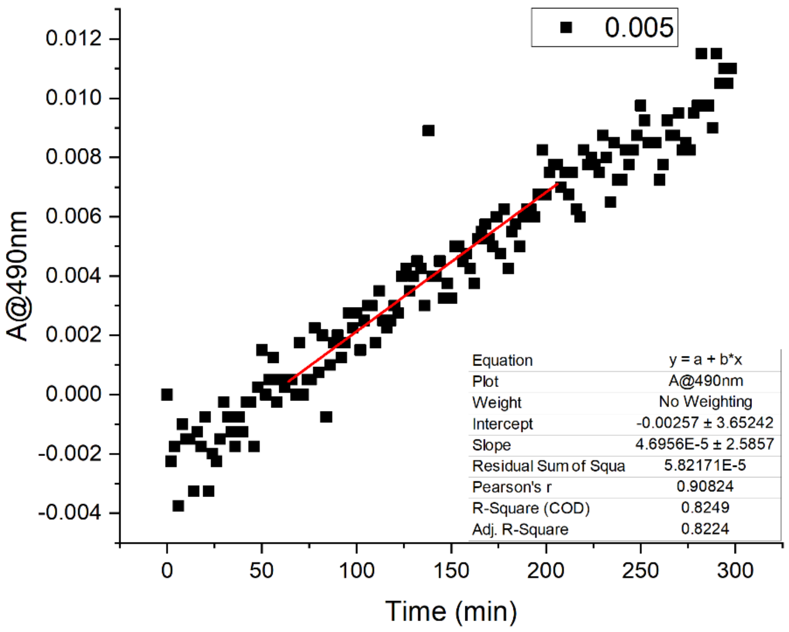

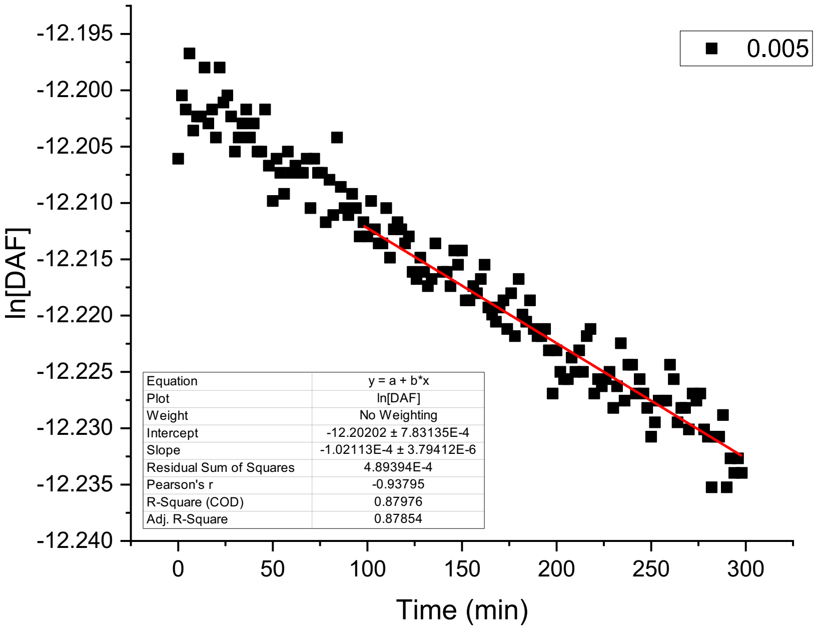

0.010 mM **DAF**

Time (min)	A@490nm minus BK	set to 0	[FLU] (M)	[DAF] (M)	ln[DAF]
0	0.0235	0	0	0.00001	-11.5129
2	0.02425	5.00E-04	6.25E-09	9.99E-06	-11.5136
4	0.02275	-0.002	-2.5E-08	1E-05	-11.5104
6	0.023	-0.00125	-1.6E-08	1E-05	-11.5114
8	0.0235	-0.0015	-1.9E-08	1E-05	-11.5111
10	0.0225	-0.002	-2.5E-08	1E-05	-11.5104
12	0.023	-0.001	-1.3E-08	1E-05	-11.5117
14	0.02275	-0.00125	-1.6E-08	1E-05	-11.5114
16	0.02375	-0.0015	-1.9E-08	1E-05	-11.5111
18	0.02275	-0.002	-2.5E-08	1E-05	-11.5104
20	0.0245	-5.00E-04	-6.3E-09	1E-05	-11.5123
22	0.0245	0.00125	1.56E-08	9.98E-06	-11.5145
24	0.02425	0.00125	1.56E-08	9.98E-06	-11.5145
26	0.0255	0.001	1.25E-08	9.99E-06	-11.5142
28	0.0245	5.00E-04	6.25E-09	9.99E-06	-11.5136
30	0.02425	7.50E-04	9.38E-09	9.99E-06	-11.5139
32	0.025	0.001	1.25E-08	9.99E-06	-11.5142
34	0.02475	0.00125	1.56E-08	9.98E-06	-11.5145
36	0.025	0.00175	2.19E-08	9.98E-06	-11.5151
38	0.02575	0.00225	2.81E-08	9.97E-06	-11.5157
40	0.02525	0.00175	2.19E-08	9.98E-06	-11.5151
42	0.02575	0.0015	1.88E-08	9.98E-06	-11.5148
44	0.026	0.0025	3.13E-08	9.97E-06	-11.5161
46	0.0255	7.50E-04	9.38E-09	9.99E-06	-11.5139
48	0.02675	0.00325	4.06E-08	9.96E-06	-11.517

50	0.02675	0.00325	4.06E-08	9.96E-06	-11.517
52	0.026	0.00375	4.69E-08	9.95E-06	-11.5176
54	0.0265	0.0035	4.38E-08	9.96E-06	-11.5173
56	0.0275	0.005	6.25E-08	9.94E-06	-11.5192
58	0.0275	0.0035	4.38E-08	9.96E-06	-11.5173
60	0.02675	0.00275	3.44E-08	9.97E-06	-11.5164
62	0.02875	0.0055	6.88E-08	9.93E-06	-11.5198
64	0.02875	0.005	6.25E-08	9.94E-06	-11.5192
66	0.0285	0.00525	6.56E-08	9.93E-06	-11.5195
68	0.02825	0.0035	4.38E-08	9.96E-06	-11.5173
70	0.029	0.005	6.25E-08	9.94E-06	-11.5192
72	0.0285	0.00425	5.31E-08	9.95E-06	-11.5183
74	0.029	0.00575	7.19E-08	9.93E-06	-11.5201
76	0.0295	0.00525	6.56E-08	9.93E-06	-11.5195
78	0.03	0.006	7.5E-08	9.93E-06	-11.5205
80	0.02925	0.00425	5.31E-08	9.95E-06	-11.5183
82	0.03025	0.00725	9.06E-08	9.91E-06	-11.522
84	0.02875	0.00425	5.31E-08	9.95E-06	-11.5183
86	0.03125	0.00675	8.44E-08	9.92E-06	-11.5214
88	0.03	0.00625	7.81E-08	9.92E-06	-11.5208
90	0.03125	0.0075	9.38E-08	9.91E-06	-11.5223
92	0.03175	0.0075	9.38E-08	9.91E-06	-11.5223
94	0.03075	0.007	8.75E-08	9.91E-06	-11.5217
96	0.032	0.0075	9.38E-08	9.91E-06	-11.5223
98	0.03225	0.00725	9.06E-08	9.91E-06	-11.522
100	0.0315	0.007	8.75E-08	9.91E-06	-11.5217
102	0.03225	0.0075	9.38E-08	9.91E-06	-11.5223
104	0.03175	0.0075	9.38E-08	9.91E-06	-11.5223
106	0.0325	0.008	1E-07	9.9E-06	-11.523
108	0.0325	0.00825	1.03E-07	9.9E-06	-11.5233
110	0.03275	0.00725	9.06E-08	9.91E-06	-11.522
112	0.0335	0.0095	1.19E-07	9.88E-06	-11.5249
114	0.034	0.009	1.13E-07	9.89E-06	-11.5242
116	0.0325	0.0075	9.38E-08	9.91E-06	-11.5223
118	0.0335	0.0085	1.06E-07	9.89E-06	-11.5236
120	0.03425	0.0075	9.38E-08	9.91E-06	-11.5223
122	0.0345	0.00975	1.22E-07	9.88E-06	-11.5252
124	0.035	0.01125	1.41E-07	9.86E-06	-11.5271
126	0.0355	0.00825	1.03E-07	9.9E-06	-11.5233
128	0.03525	0.01075	1.34E-07	9.87E-06	-11.5265
130	0.0365	0.0115	1.44E-07	9.86E-06	-11.5274
132	0.0365	0.01275	1.59E-07	9.84E-06	-11.529
134	0.03575	0.011	1.38E-07	9.86E-06	-11.5268
136	0.035	0.0095	1.19E-07	9.88E-06	-11.5249
138	0.0365	0.01523	1.9E-07	9.81E-06	-11.5321
140	0.0355	0.008	1E-07	9.9E-06	-11.523

142	0.037	0.0125	1.56E-07	9.84E-06	-11.5287
144	0.037	0.012	1.5E-07	9.85E-06	-11.528
146	0.03675	0.0115	1.44E-07	9.86E-06	-11.5274
148	0.03725	0.01225	1.53E-07	9.85E-06	-11.5284
150	0.03725	0.0125	1.56E-07	9.84E-06	-11.5287
152	0.038	0.01225	1.53E-07	9.85E-06	-11.5284
154	0.038	0.0115	1.44E-07	9.86E-06	-11.5274
156	0.0375	0.01225	1.53E-07	9.85E-06	-11.5284
158	0.03825	0.013	1.63E-07	9.84E-06	-11.5293
160	0.0385	0.013	1.63E-07	9.84E-06	-11.5293
162	0.03775	0.01225	1.53E-07	9.85E-06	-11.5284
164	0.039	0.01425	1.78E-07	9.82E-06	-11.5309
166	0.03825	0.0135	1.69E-07	9.83E-06	-11.5299
168	0.0395	0.0145	1.81E-07	9.82E-06	-11.5312
170	0.039	0.014	1.75E-07	9.83E-06	-11.5306
172	0.0395	0.015	1.88E-07	9.81E-06	-11.5319
174	0.04	0.01475	1.84E-07	9.82E-06	-11.5315
176	0.03975	0.01375	1.72E-07	9.83E-06	-11.5303
178	0.04025	0.016	2E-07	9.8E-06	-11.5331
180	0.03825	0.01325	1.66E-07	9.83E-06	-11.5296
182	0.04025	0.01475	1.84E-07	9.82E-06	-11.5315
184	0.0405	0.01525	1.91E-07	9.81E-06	-11.5322
186	0.04025	0.01475	1.84E-07	9.82E-06	-11.5315
188	0.04075	0.01525	1.91E-07	9.81E-06	-11.5322
190	0.04025	0.01575	1.97E-07	9.8E-06	-11.5328
192	0.04175	0.01625	2.03E-07	9.8E-06	-11.5334
194	0.0415	0.01575	1.97E-07	9.8E-06	-11.5328
196	0.0415	0.016	2E-07	9.8E-06	-11.5331
198	0.043	0.01825	2.28E-07	9.77E-06	-11.536
200	0.043	0.01875	2.34E-07	9.77E-06	-11.5366
202	0.04325	0.01775	2.22E-07	9.78E-06	-11.5354
204	0.0425	0.017	2.13E-07	9.79E-06	-11.5344
206	0.04225	0.0165	2.06E-07	9.79E-06	-11.5338
208	0.0425	0.0175	2.19E-07	9.78E-06	-11.535
210	0.0425	0.01725	2.16E-07	9.78E-06	-11.5347
212	0.043	0.01625	2.03E-07	9.8E-06	-11.5334
214	0.0435	0.018	2.25E-07	9.78E-06	-11.5357
216	0.04375	0.01775	2.22E-07	9.78E-06	-11.5354
218	0.0435	0.01725	2.16E-07	9.78E-06	-11.5347
220	0.0445	0.019	2.38E-07	9.76E-06	-11.537
222	0.045	0.019	2.38E-07	9.76E-06	-11.537
224	0.04425	0.019	2.38E-07	9.76E-06	-11.537
226	0.04525	0.01975	2.47E-07	9.75E-06	-11.5379
228	0.0445	0.0185	2.31E-07	9.77E-06	-11.5363
230	0.04475	0.0195	2.44E-07	9.76E-06	-11.5376
232	0.0455	0.019	2.38E-07	9.76E-06	-11.537

234	0.04525	0.01925	2.41E-07	9.76E-06	-11.5373
236	0.04575	0.02075	2.59E-07	9.74E-06	-11.5392
238	0.04425	0.01825	2.28E-07	9.77E-06	-11.536
240	0.04625	0.02	2.5E-07	9.75E-06	-11.5382
242	0.046	0.0195	2.44E-07	9.76E-06	-11.5376
244	0.0455	0.01975	2.47E-07	9.75E-06	-11.5379
246	0.04625	0.02075	2.59E-07	9.74E-06	-11.5392
248	0.04625	0.02	2.5E-07	9.75E-06	-11.5382
250	0.04625	0.021	2.63E-07	9.74E-06	-11.5395
252	0.04675	0.02075	2.59E-07	9.74E-06	-11.5392
254	0.0465	0.0205	2.56E-07	9.74E-06	-11.5389
256	0.0465	0.02	2.5E-07	9.75E-06	-11.5382
258	0.0475	0.021	2.63E-07	9.74E-06	-11.5395
260	0.047	0.02125	2.66E-07	9.73E-06	-11.5398
262	0.0465	0.01925	2.41E-07	9.76E-06	-11.5373
264	0.0485	0.0225	2.81E-07	9.72E-06	-11.5415
266	0.047	0.021	2.63E-07	9.74E-06	-11.5395
268	0.04775	0.02125	2.66E-07	9.73E-06	-11.5398
270	0.04775	0.0215	2.69E-07	9.73E-06	-11.5402
272	0.048	0.021	2.63E-07	9.74E-06	-11.5395
274	0.0475	0.02	2.5E-07	9.75E-06	-11.5382
276	0.04775	0.02125	2.66E-07	9.73E-06	-11.5398
278	0.04825	0.0215	2.69E-07	9.73E-06	-11.5402
280	0.04825	0.02175	2.72E-07	9.73E-06	-11.5405
282	0.0495	0.023	2.88E-07	9.71E-06	-11.5421
284	0.04875	0.02225	2.78E-07	9.72E-06	-11.5411
286	0.04875	0.02225	2.78E-07	9.72E-06	-11.5411
288	0.04875	0.02175	2.72E-07	9.73E-06	-11.5405
290	0.04925	0.023	2.88E-07	9.71E-06	-11.5421
292	0.04925	0.023	2.88E-07	9.71E-06	-11.5421
294	0.04975	0.0225	2.81E-07	9.72E-06	-11.5415
296	0.04925	0.0235	2.94E-07	9.71E-06	-11.5427
298	0.05	0.023	2.88E-07	9.71E-06	-11.5421

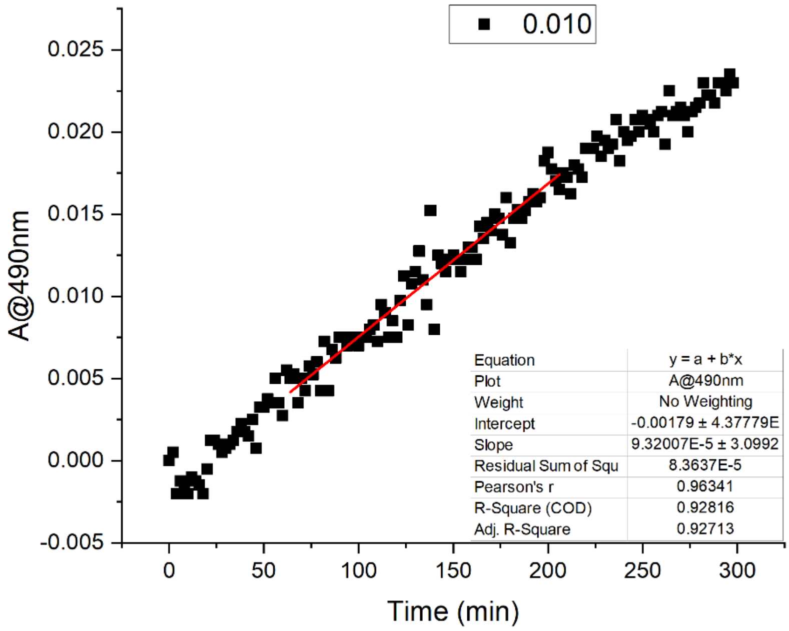

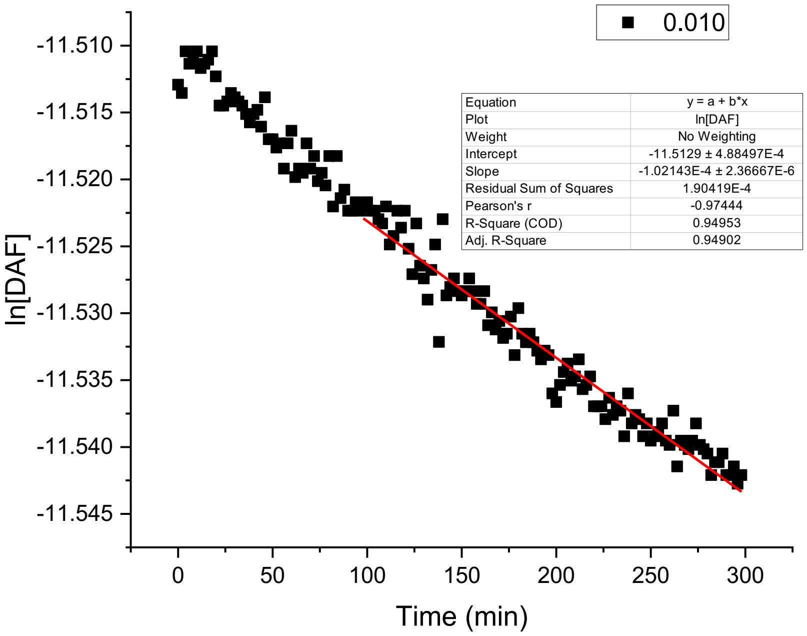

0.015 mM **DAF**

Time (min)	A@490nm minus BK	set to 0	[FLU] (M)	[DAF] (M)	ln[DAF]
0	0.02275	0	0	0.000015	-11.1075
2	0.0225	5.00E-04	6.25E-09	1.5E-05	-11.1079
4	0.0225	-2.50E-04	-3.1E-09	1.5E-05	-11.1073
6	0.023	0.001	1.25E-08	1.5E-05	-11.1083
8	0.023	-0.00175	-2.2E-08	1.5E-05	-11.106
10	0.02225	-5.00E-04	-6.3E-09	1.5E-05	-11.107

12	0.02275	0	0	0.000015	-11.1075
14	0.022	-0.001	-1.3E-08	1.5E-05	-11.1066
16	0.02425	0.00225	2.81E-08	1.5E-05	-11.1093
18	0.02275	-7.50E-04	-9.4E-09	1.5E-05	-11.1068
20	0.024	-5.00E-04	-6.3E-09	1.5E-05	-11.107
22	0.02375	0.0015	1.88E-08	1.5E-05	-11.1087
24	0.0235	0.00225	2.81E-08	1.5E-05	-11.1093
26	0.02475	0.00125	1.56E-08	1.5E-05	-11.1085
28	0.02425	0.0015	1.88E-08	1.5E-05	-11.1087
30	0.02425	0.0015	1.88E-08	1.5E-05	-11.1087
32	0.025	0.00225	2.81E-08	1.5E-05	-11.1093
34	0.02525	0.003	3.75E-08	1.5E-05	-11.11
36	0.0245	0.00125	1.56E-08	1.5E-05	-11.1085
38	0.0255	0.0025	3.13E-08	1.5E-05	-11.1095
40	0.0255	0.003	3.75E-08	1.5E-05	-11.11
42	0.026	0.003	3.75E-08	1.5E-05	-11.11
44	0.0265	0.0035	4.38E-08	1.5E-05	-11.1104
46	0.026	0.0025	3.13E-08	1.5E-05	-11.1095
48	0.027	0.00375	4.69E-08	1.5E-05	-11.1106
50	0.0285	0.00475	5.94E-08	1.49E-05	-11.1114
52	0.02625	0.00575	7.19E-08	1.49E-05	-11.1123
54	0.02775	0.00475	5.94E-08	1.49E-05	-11.1114
56	0.0285	0.0065	8.13E-08	1.49E-05	-11.1129
58	0.02875	0.0055	6.88E-08	1.49E-05	-11.1121
60	0.0285	0.0055	6.88E-08	1.49E-05	-11.1121
62	0.02875	0.006	7.5E-08	1.49E-05	-11.1125
64	0.02925	0.006	7.5E-08	1.49E-05	-11.1125
66	0.029	0.0055	6.88E-08	1.49E-05	-11.1121
68	0.02975	0.0065	8.13E-08	1.49E-05	-11.1129
70	0.0305	0.00775	9.69E-08	1.49E-05	-11.1139
72	0.03	0.0065	8.13E-08	1.49E-05	-11.1129
74	0.031	0.0085	1.06E-07	1.49E-05	-11.1146
76	0.03	0.007	8.75E-08	1.49E-05	-11.1133
78	0.031	0.0075	9.38E-08	1.49E-05	-11.1137
80	0.031	0.00625	7.81E-08	1.49E-05	-11.1127
82	0.03175	0.00975	1.22E-07	1.49E-05	-11.1156
84	0.0305	0.00725	9.06E-08	1.49E-05	-11.1135
86	0.033	0.009	1.13E-07	1.49E-05	-11.115
88	0.03325	0.01025	1.28E-07	1.49E-05	-11.116
90	0.0335	0.0115	1.44E-07	1.49E-05	-11.1171
92	0.0335	0.01	1.25E-07	1.49E-05	-11.1158
94	0.03325	0.0095	1.19E-07	1.49E-05	-11.1154
96	0.034	0.01125	1.41E-07	1.49E-05	-11.1169
98	0.03475	0.01025	1.28E-07	1.49E-05	-11.116
100	0.034	0.011	1.38E-07	1.49E-05	-11.1167
102	0.035	0.01175	1.47E-07	1.49E-05	-11.1173

104	0.035	0.011	1.38E-07	1.49E-05	-11.1167
106	0.03575	0.01275	1.59E-07	1.48E-05	-11.1181
108	0.03575	0.01175	1.47E-07	1.49E-05	-11.1173
110	0.03525	0.011	1.38E-07	1.49E-05	-11.1167
112	0.036	0.0125	1.56E-07	1.48E-05	-11.1179
114	0.03675	0.0135	1.69E-07	1.48E-05	-11.1188
116	0.03625	0.01275	1.59E-07	1.48E-05	-11.1181
118	0.03725	0.013	1.63E-07	1.48E-05	-11.1184
120	0.03775	0.01475	1.84E-07	1.48E-05	-11.1198
122	0.03825	0.01475	1.84E-07	1.48E-05	-11.1198
124	0.03875	0.016	2E-07	1.48E-05	-11.1209
126	0.03925	0.0155	1.94E-07	1.48E-05	-11.1205
128	0.0385	0.015	1.88E-07	1.48E-05	-11.12
130	0.04025	0.016	2E-07	1.48E-05	-11.1209
132	0.04025	0.0175	2.19E-07	1.48E-05	-11.1222
134	0.0405	0.017	2.13E-07	1.48E-05	-11.1217
136	0.0405	0.01575	1.97E-07	1.48E-05	-11.1207
138	0.0405	0.02025	2.53E-07	1.47E-05	-11.1245
140	0.04025	0.0165	2.06E-07	1.48E-05	-11.1213
142	0.04175	0.01775	2.22E-07	1.48E-05	-11.1224
144	0.04175	0.01775	2.22E-07	1.48E-05	-11.1224
146	0.04175	0.017	2.13E-07	1.48E-05	-11.1217
148	0.042	0.017	2.13E-07	1.48E-05	-11.1217
150	0.04225	0.01775	2.22E-07	1.48E-05	-11.1224
152	0.04275	0.019	2.38E-07	1.48E-05	-11.1234
154	0.04275	0.0175	2.19E-07	1.48E-05	-11.1222
156	0.04325	0.019	2.38E-07	1.48E-05	-11.1234
158	0.04375	0.0195	2.44E-07	1.48E-05	-11.1238
160	0.044	0.01925	2.41E-07	1.48E-05	-11.1236
162	0.04325	0.019	2.38E-07	1.48E-05	-11.1234
164	0.044	0.02025	2.53E-07	1.47E-05	-11.1245
166	0.04475	0.0205	2.56E-07	1.47E-05	-11.1247
168	0.04475	0.02125	2.66E-07	1.47E-05	-11.1253
170	0.04525	0.02125	2.66E-07	1.47E-05	-11.1253
172	0.04475	0.021	2.63E-07	1.47E-05	-11.1251
174	0.04575	0.0215	2.69E-07	1.47E-05	-11.1255
176	0.046	0.02075	2.59E-07	1.47E-05	-11.1249
178	0.04625	0.02225	2.78E-07	1.47E-05	-11.1262
180	0.0445	0.02075	2.59E-07	1.47E-05	-11.1249
182	0.04675	0.02225	2.78E-07	1.47E-05	-11.1262
184	0.04725	0.023	2.88E-07	1.47E-05	-11.1268
186	0.0475	0.023	2.88E-07	1.47E-05	-11.1268
188	0.047	0.022	2.75E-07	1.47E-05	-11.126
190	0.04825	0.024	3E-07	1.47E-05	-11.1277
192	0.0485	0.02375	2.97E-07	1.47E-05	-11.1275
194	0.049	0.02425	3.03E-07	1.47E-05	-11.1279

196	0.049	0.02475	3.09E-07	1.47E-05	-11.1283
198	0.0495	0.026	3.25E-07	1.47E-05	-11.1294
200	0.04925	0.02475	3.09E-07	1.47E-05	-11.1283
202	0.05	0.02575	3.22E-07	1.47E-05	-11.1292
204	0.05025	0.0265	3.31E-07	1.47E-05	-11.1298
206	0.05025	0.0255	3.19E-07	1.47E-05	-11.1289
208	0.05025	0.026	3.25E-07	1.47E-05	-11.1294
210	0.04975	0.02525	3.16E-07	1.47E-05	-11.1287
212	0.051	0.026	3.25E-07	1.47E-05	-11.1294
214	0.051	0.0265	3.31E-07	1.47E-05	-11.1298
216	0.052	0.027	3.38E-07	1.47E-05	-11.1302
218	0.05125	0.02525	3.16E-07	1.47E-05	-11.1287
220	0.0525	0.02825	3.53E-07	1.46E-05	-11.1313
222	0.053	0.02825	3.53E-07	1.46E-05	-11.1313
224	0.052	0.028	3.5E-07	1.47E-05	-11.1311
226	0.0535	0.0295	3.69E-07	1.46E-05	-11.1324
228	0.0525	0.028	3.5E-07	1.47E-05	-11.1311
230	0.0535	0.029	3.63E-07	1.46E-05	-11.1319
232	0.05375	0.029	3.63E-07	1.46E-05	-11.1319
234	0.05275	0.028	3.5E-07	1.47E-05	-11.1311
236	0.05375	0.0295	3.69E-07	1.46E-05	-11.1324
238	0.05325	0.028	3.5E-07	1.47E-05	-11.1311
240	0.05325	0.02775	3.47E-07	1.47E-05	-11.1309
242	0.05425	0.02925	3.66E-07	1.46E-05	-11.1321
244	0.0535	0.02825	3.53E-07	1.46E-05	-11.1313
246	0.05575	0.03175	3.97E-07	1.46E-05	-11.1343
248	0.05475	0.02975	3.72E-07	1.46E-05	-11.1326
250	0.0555	0.03025	3.78E-07	1.46E-05	-11.133
252	0.05575	0.03	3.75E-07	1.46E-05	-11.1328
254	0.0555	0.03125	3.91E-07	1.46E-05	-11.1338
256	0.05575	0.03025	3.78E-07	1.46E-05	-11.133
258	0.05525	0.02975	3.72E-07	1.46E-05	-11.1326
260	0.056	0.0305	3.81E-07	1.46E-05	-11.1332
262	0.05575	0.02975	3.72E-07	1.46E-05	-11.1326
264	0.05775	0.03225	4.03E-07	1.46E-05	-11.1347
266	0.05575	0.0305	3.81E-07	1.46E-05	-11.1332
268	0.058	0.0325	4.06E-07	1.46E-05	-11.1349
270	0.05775	0.03275	4.09E-07	1.46E-05	-11.1351
272	0.05775	0.032	4E-07	1.46E-05	-11.1345
274	0.05875	0.032	4E-07	1.46E-05	-11.1345
276	0.05825	0.0325	4.06E-07	1.46E-05	-11.1349
278	0.0585	0.03325	4.16E-07	1.46E-05	-11.1356
280	0.0595	0.0335	4.19E-07	1.46E-05	-11.1358
282	0.0605	0.03525	4.41E-07	1.46E-05	-11.1373
284	0.059	0.03275	4.09E-07	1.46E-05	-11.1351
286	0.0595	0.03375	4.22E-07	1.46E-05	-11.136

288	0.059	0.033	4.13E-07	1.46E-05	-11.1353
290	0.05975	0.034	4.25E-07	1.46E-05	-11.1362
292	0.0595	0.0335	4.19E-07	1.46E-05	-11.1358
294	0.06	0.034	4.25E-07	1.46E-05	-11.1362
296	0.06	0.0345	4.31E-07	1.46E-05	-11.1366
298	0.061	0.03475	4.34E-07	1.46E-05	-11.1368

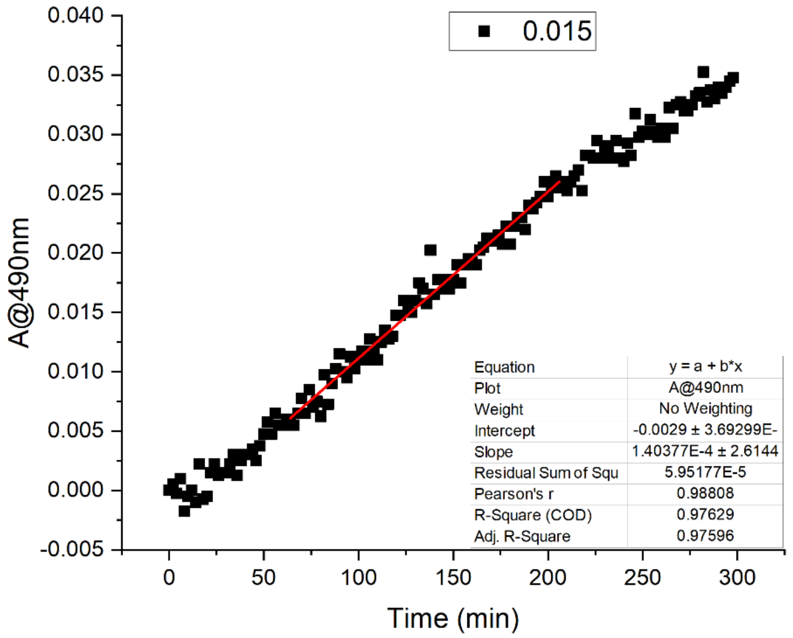

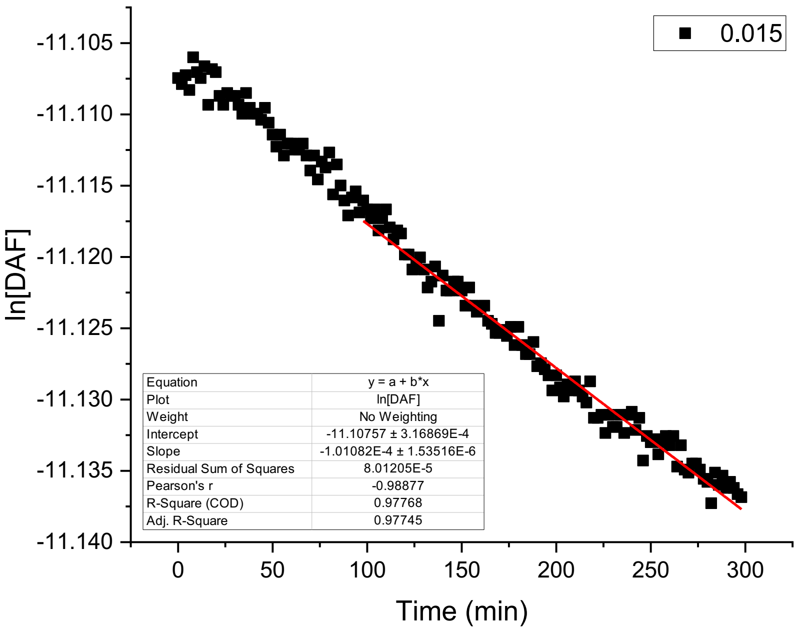

0.020 mM **DAF**

Time (min)	A@490nm minus BK	set to 0	[FLU] (M)	[DAF] (M)	ln[DAF]
0	0.022	0	0	0.00002	-10.8198
2	0.02275	7.50E-04	9.38E-09	2E-05	-10.8202
4	0.02225	0	0	0.00002	-10.8198
6	0.02325	0.0015	1.88E-08	2E-05	-10.8207
8	0.02225	0	0	0.00002	-10.8198
10	0.0225	0	0	0.00002	-10.8198
12	0.0225	2.50E-04	3.13E-09	2E-05	-10.8199
14	0.0225	-5.00E-04	-6.3E-09	2E-05	-10.8195
16	0.024	0.0015	1.88E-08	2E-05	-10.8207
18	0.02275	5.00E-04	6.25E-09	2E-05	-10.8201
20	0.024	7.50E-04	9.38E-09	2E-05	-10.8202
22	0.024	0.002	2.5E-08	2E-05	-10.821
24	0.024	0.00175	2.19E-08	2E-05	-10.8209
26	0.02525	0.00175	2.19E-08	2E-05	-10.8209
28	0.02525	0.00275	3.44E-08	2E-05	-10.8215
30	0.0245	0.002	2.5E-08	2E-05	-10.821
32	0.02475	0.00175	2.19E-08	2E-05	-10.8209
34	0.02525	0.004	5E-08	2E-05	-10.8223
36	0.02525	0.00225	2.81E-08	2E-05	-10.8212
38	0.0255	0.0035	4.38E-08	2E-05	-10.822
40	0.0255	0.00325	4.06E-08	2E-05	-10.8218
42	0.02675	0.00475	5.94E-08	1.99E-05	-10.8228
44	0.02625	0.0035	4.38E-08	2E-05	-10.822
46	0.02625	0.00325	4.06E-08	2E-05	-10.8218
48	0.02825	0.00625	7.81E-08	1.99E-05	-10.8237
50	0.02875	0.00675	8.44E-08	1.99E-05	-10.824
52	0.0275	0.00675	8.44E-08	1.99E-05	-10.824
54	0.0285	0.005	6.25E-08	1.99E-05	-10.8229
56	0.02925	0.007	8.75E-08	1.99E-05	-10.8242
58	0.029	0.0055	6.88E-08	1.99E-05	-10.8232
60	0.02925	0.0055	6.88E-08	1.99E-05	-10.8232
62	0.0295	0.0065	8.13E-08	1.99E-05	-10.8238
64	0.0305	0.0075	9.38E-08	1.99E-05	-10.8245
66	0.031	0.008	1E-07	1.99E-05	-10.8248
68	0.0315	0.00825	1.03E-07	1.99E-05	-10.8249
70	0.0315	0.009	1.13E-07	1.99E-05	-10.8254
72	0.03225	0.0095	1.19E-07	1.99E-05	-10.8257
74	0.03175	0.0095	1.19E-07	1.99E-05	-10.8257
76	0.03275	0.01	1.25E-07	1.99E-05	-10.826
78	0.0335	0.0105	1.31E-07	1.99E-05	-10.8264
80	0.0335	0.01125	1.41E-07	1.99E-05	-10.8268
82	0.03375	0.01225	1.53E-07	1.98E-05	-10.8275
84	0.032	0.00875	1.09E-07	1.99E-05	-10.8253
86	0.03525	0.01275	1.59E-07	1.98E-05	-10.8278

88	0.035	0.013	1.63E-07	1.98E-05	-10.8279
90	0.03575	0.01475	1.84E-07	1.98E-05	-10.829
92	0.0365	0.01325	1.66E-07	1.98E-05	-10.8281
94	0.03625	0.01375	1.72E-07	1.98E-05	-10.8284
96	0.03725	0.01475	1.84E-07	1.98E-05	-10.829
98	0.03775	0.0155	1.94E-07	1.98E-05	-10.8295
100	0.03675	0.014	1.75E-07	1.98E-05	-10.8286
102	0.038	0.015	1.88E-07	1.98E-05	-10.8292
104	0.03775	0.01425	1.78E-07	1.98E-05	-10.8287
106	0.03925	0.0165	2.06E-07	1.98E-05	-10.8301
108	0.0395	0.01675	2.09E-07	1.98E-05	-10.8303
110	0.039	0.01525	1.91E-07	1.98E-05	-10.8294
112	0.04025	0.01725	2.16E-07	1.98E-05	-10.8306
114	0.04075	0.0175	2.19E-07	1.98E-05	-10.8308
116	0.041	0.018	2.25E-07	1.98E-05	-10.8311
118	0.04125	0.018	2.25E-07	1.98E-05	-10.8311
120	0.04175	0.01825	2.28E-07	1.98E-05	-10.8313
122	0.043	0.0205	2.56E-07	1.97E-05	-10.8327
124	0.0435	0.021	2.63E-07	1.97E-05	-10.833
126	0.04375	0.0205	2.56E-07	1.97E-05	-10.8327
128	0.0435	0.0205	2.56E-07	1.97E-05	-10.8327
130	0.0455	0.0225	2.81E-07	1.97E-05	-10.8339
132	0.04475	0.022	2.75E-07	1.97E-05	-10.8336
134	0.04525	0.02175	2.72E-07	1.97E-05	-10.8335
136	0.04475	0.021	2.63E-07	1.97E-05	-10.833
138	0.04625	0.02625	3.28E-07	1.97E-05	-10.8363
140	0.04625	0.023	2.88E-07	1.97E-05	-10.8343
142	0.04625	0.02275	2.84E-07	1.97E-05	-10.8341
144	0.0475	0.02425	3.03E-07	1.97E-05	-10.8351
146	0.048	0.0245	3.06E-07	1.97E-05	-10.8352
148	0.04775	0.02375	2.97E-07	1.97E-05	-10.8347
150	0.0475	0.02325	2.91E-07	1.97E-05	-10.8344
152	0.0485	0.025	3.13E-07	1.97E-05	-10.8355
154	0.04925	0.0255	3.19E-07	1.97E-05	-10.8358
156	0.0495	0.0255	3.19E-07	1.97E-05	-10.8358
158	0.05	0.026	3.25E-07	1.97E-05	-10.8362
160	0.04975	0.026	3.25E-07	1.97E-05	-10.8362
162	0.04975	0.02625	3.28E-07	1.97E-05	-10.8363
164	0.05075	0.0265	3.31E-07	1.97E-05	-10.8365
166	0.05125	0.027	3.38E-07	1.97E-05	-10.8368
168	0.0515	0.028	3.5E-07	1.97E-05	-10.8374
170	0.05125	0.0265	3.31E-07	1.97E-05	-10.8365
172	0.05175	0.02825	3.53E-07	1.96E-05	-10.8376
174	0.05275	0.0285	3.56E-07	1.96E-05	-10.8378
176	0.053	0.0285	3.56E-07	1.96E-05	-10.8378
178	0.053	0.0295	3.69E-07	1.96E-05	-10.8384

180	0.052	0.02825	3.53E-07	1.96E-05	-10.8376
182	0.0535	0.0285	3.56E-07	1.96E-05	-10.8378
184	0.0545	0.03025	3.78E-07	1.96E-05	-10.8389
186	0.0545	0.03025	3.78E-07	1.96E-05	-10.8389
188	0.05475	0.0305	3.81E-07	1.96E-05	-10.839
190	0.05575	0.0315	3.94E-07	1.96E-05	-10.8397
192	0.056	0.03175	3.97E-07	1.96E-05	-10.8398
194	0.05675	0.03225	4.03E-07	1.96E-05	-10.8401
196	0.0565	0.03325	4.16E-07	1.96E-05	-10.8408
198	0.0575	0.03375	4.22E-07	1.96E-05	-10.8411
200	0.057	0.03275	4.09E-07	1.96E-05	-10.8405
202	0.05825	0.034	4.25E-07	1.96E-05	-10.8413
204	0.0575	0.03325	4.16E-07	1.96E-05	-10.8408
206	0.05825	0.03375	4.22E-07	1.96E-05	-10.8411
208	0.0575	0.03325	4.16E-07	1.96E-05	-10.8408
210	0.05925	0.03475	4.34E-07	1.96E-05	-10.8417
212	0.0595	0.0345	4.31E-07	1.96E-05	-10.8416
214	0.05975	0.035	4.38E-07	1.96E-05	-10.8419
216	0.05975	0.03475	4.34E-07	1.96E-05	-10.8417
218	0.0605	0.03425	4.28E-07	1.96E-05	-10.8414
220	0.06125	0.03675	4.59E-07	1.95E-05	-10.843
222	0.06125	0.0365	4.56E-07	1.95E-05	-10.8429
224	0.06175	0.037	4.63E-07	1.95E-05	-10.8432
226	0.063	0.0385	4.81E-07	1.95E-05	-10.8441
228	0.06275	0.03775	4.72E-07	1.95E-05	-10.8437
230	0.06275	0.038	4.75E-07	1.95E-05	-10.8438
232	0.0635	0.038	4.75E-07	1.95E-05	-10.8438
234	0.06225	0.036	4.5E-07	1.96E-05	-10.8425
236	0.06325	0.03875	4.84E-07	1.95E-05	-10.8443
238	0.063	0.038	4.75E-07	1.95E-05	-10.8438
240	0.06425	0.03875	4.84E-07	1.95E-05	-10.8443
242	0.06425	0.0385	4.81E-07	1.95E-05	-10.8441
244	0.06375	0.038	4.75E-07	1.95E-05	-10.8438
246	0.065	0.04	5E-07	1.95E-05	-10.8451
248	0.0655	0.03975	4.97E-07	1.95E-05	-10.8449
250	0.0655	0.0405	5.06E-07	1.95E-05	-10.8454
252	0.06675	0.04075	5.09E-07	1.95E-05	-10.8456
254	0.0665	0.04075	5.09E-07	1.95E-05	-10.8456
256	0.0675	0.0415	5.19E-07	1.95E-05	-10.8461
258	0.06675	0.04	5E-07	1.95E-05	-10.8451
260	0.067	0.0405	5.06E-07	1.95E-05	-10.8454
262	0.067	0.04025	5.03E-07	1.95E-05	-10.8453
264	0.0685	0.04225	5.28E-07	1.95E-05	-10.8465
266	0.06775	0.04225	5.28E-07	1.95E-05	-10.8465
268	0.068	0.042	5.25E-07	1.95E-05	-10.8464
270	0.06925	0.04375	5.47E-07	1.95E-05	-10.8475

272	0.06975	0.043	5.38E-07	1.95E-05	-10.847
274	0.07025	0.043	5.38E-07	1.95E-05	-10.847
276	0.06975	0.0435	5.44E-07	1.95E-05	-10.8473
278	0.07025	0.04425	5.53E-07	1.94E-05	-10.8478
280	0.071	0.04475	5.59E-07	1.94E-05	-10.8481
282	0.07175	0.04575	5.72E-07	1.94E-05	-10.8488
284	0.0715	0.04525	5.66E-07	1.94E-05	-10.8485
286	0.07125	0.045	5.63E-07	1.94E-05	-10.8483
288	0.0715	0.044	5.5E-07	1.95E-05	-10.8477
290	0.0715	0.0455	5.69E-07	1.94E-05	-10.8486
292	0.0725	0.0455	5.69E-07	1.94E-05	-10.8486
294	0.07175	0.0455	5.69E-07	1.94E-05	-10.8486
296	0.0725	0.04625	5.78E-07	1.94E-05	-10.8491
298	0.07325	0.04525	5.66E-07	1.94E-05	-10.8485

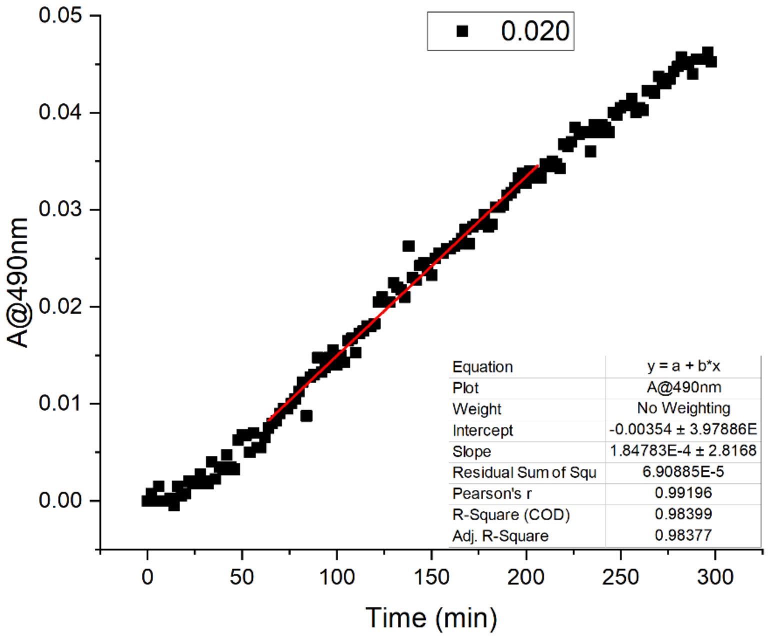

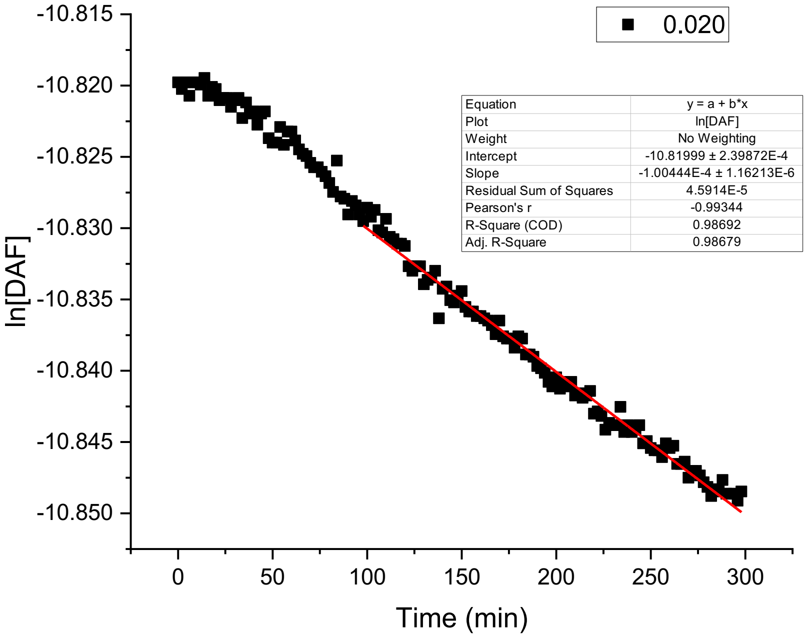

0.025 mM **DAF**

Time (min)	A@490nm minus BK	set to 0	[FLU] (M)	[DAF] (M)	ln[DAF]
0	0.02325	0	0	0.000025	-10.5966
2	0.02325	0	0	0.000025	-10.5966
4	0.02325	-5.00E-04	-6.3E-09	2.5E-05	-10.5964
6	0.02325	0	0	0.000025	-10.5966
8	0.0235	5.00E-04	6.25E-09	2.5E-05	-10.5969
10	0.02375	2.50E-04	3.13E-09	2.5E-05	-10.5968
12	0.0245	0.001	1.25E-08	2.5E-05	-10.5971
14	0.024	-0.001	-1.3E-08	2.5E-05	-10.5961
16	0.025	0.00175	2.19E-08	2.5E-05	-10.5975
18	0.025	0.00125	1.56E-08	2.5E-05	-10.5973
20	0.02575	0.001	1.25E-08	2.5E-05	-10.5971
22	0.0245	7.50E-04	9.38E-09	2.5E-05	-10.597
24	0.026	0.00225	2.81E-08	2.5E-05	-10.5978
26	0.02675	0.003	3.75E-08	2.5E-05	-10.5981
28	0.0265	0.002	2.5E-08	2.5E-05	-10.5976
30	0.0265	0.002	2.5E-08	2.5E-05	-10.5976
32	0.02725	0.003	3.75E-08	2.5E-05	-10.5981
34	0.02675	0.00325	4.06E-08	2.5E-05	-10.5983
36	0.02775	0.00375	4.69E-08	2.5E-05	-10.5985
38	0.02825	0.0045	5.63E-08	2.49E-05	-10.5989
40	0.0285	0.005	6.25E-08	2.49E-05	-10.5991
42	0.02875	0.00425	5.31E-08	2.49E-05	-10.5988
44	0.02925	0.0045	5.63E-08	2.49E-05	-10.5989
46	0.02875	0.00425	5.31E-08	2.49E-05	-10.5988
48	0.031	0.00675	8.44E-08	2.49E-05	-10.6

50	0.03075	0.00675	8.44E-08	2.49E-05	-10.6
52	0.0305	0.008	1E-07	2.49E-05	-10.6006
54	0.03125	0.00725	9.06E-08	2.49E-05	-10.6003
56	0.0315	0.008	1E-07	2.49E-05	-10.6006
58	0.03175	0.007	8.75E-08	2.49E-05	-10.6001
60	0.032	0.00675	8.44E-08	2.49E-05	-10.6
62	0.03375	0.00925	1.16E-07	2.49E-05	-10.6013
64	0.034	0.01	1.25E-07	2.49E-05	-10.6016
66	0.034	0.00875	1.09E-07	2.49E-05	-10.601
68	0.0345	0.00925	1.16E-07	2.49E-05	-10.6013
70	0.03425	0.00975	1.22E-07	2.49E-05	-10.6015
72	0.0355	0.01025	1.28E-07	2.49E-05	-10.6018
74	0.036	0.01175	1.47E-07	2.49E-05	-10.6025
76	0.03525	0.00975	1.22E-07	2.49E-05	-10.6015
78	0.0365	0.01225	1.53E-07	2.48E-05	-10.6028
80	0.037	0.01175	1.47E-07	2.49E-05	-10.6025
82	0.0365	0.0125	1.56E-07	2.48E-05	-10.6029
84	0.03675	0.01175	1.47E-07	2.49E-05	-10.6025
86	0.03825	0.01325	1.66E-07	2.48E-05	-10.6033
88	0.039	0.015	1.88E-07	2.48E-05	-10.6042
90	0.0395	0.0155	1.94E-07	2.48E-05	-10.6044
92	0.03975	0.015	1.88E-07	2.48E-05	-10.6042
94	0.04025	0.015	1.88E-07	2.48E-05	-10.6042
96	0.041	0.01625	2.03E-07	2.48E-05	-10.6048
98	0.04125	0.01675	2.09E-07	2.48E-05	-10.605
100	0.04125	0.016	2E-07	2.48E-05	-10.6047
102	0.04175	0.01725	2.16E-07	2.48E-05	-10.6053
104	0.04175	0.017	2.13E-07	2.48E-05	-10.6052
106	0.04275	0.018	2.25E-07	2.48E-05	-10.6057
108	0.04325	0.018	2.25E-07	2.48E-05	-10.6057
110	0.04325	0.01725	2.16E-07	2.48E-05	-10.6053
112	0.0435	0.0185	2.31E-07	2.48E-05	-10.6059
114	0.0455	0.02025	2.53E-07	2.47E-05	-10.6068
116	0.04575	0.0205	2.56E-07	2.47E-05	-10.6069
118	0.04575	0.0195	2.44E-07	2.48E-05	-10.6064
120	0.04625	0.021	2.63E-07	2.47E-05	-10.6072
122	0.04725	0.0205	2.56E-07	2.47E-05	-10.6069
124	0.04675	0.022	2.75E-07	2.47E-05	-10.6077
126	0.048	0.0225	2.81E-07	2.47E-05	-10.6079
128	0.0475	0.0225	2.81E-07	2.47E-05	-10.6079
130	0.04925	0.0235	2.94E-07	2.47E-05	-10.6085
132	0.04875	0.02375	2.97E-07	2.47E-05	-10.6086
134	0.05	0.024	3E-07	2.47E-05	-10.6087
136	0.04975	0.02425	3.03E-07	2.47E-05	-10.6088
138	0.051	0.03	3.75E-07	2.46E-05	-10.6117
140	0.05125	0.026	3.25E-07	2.47E-05	-10.6097

142	0.051	0.02525	3.16E-07	2.47E-05	-10.6093
144	0.05225	0.027	3.38E-07	2.47E-05	-10.6102
146	0.05225	0.026	3.25E-07	2.47E-05	-10.6097
148	0.05375	0.02675	3.34E-07	2.47E-05	-10.6101
150	0.05275	0.0265	3.31E-07	2.47E-05	-10.61
152	0.0535	0.028	3.5E-07	2.47E-05	-10.6107
154	0.0545	0.02775	3.47E-07	2.47E-05	-10.6106
156	0.05475	0.029	3.63E-07	2.46E-05	-10.6112
158	0.05525	0.028	3.5E-07	2.47E-05	-10.6107
160	0.0555	0.0295	3.69E-07	2.46E-05	-10.6115
162	0.05575	0.029	3.63E-07	2.46E-05	-10.6112
164	0.0575	0.0305	3.81E-07	2.46E-05	-10.612
166	0.0565	0.03025	3.78E-07	2.46E-05	-10.6119
168	0.05775	0.03075	3.84E-07	2.46E-05	-10.6121
170	0.058	0.03075	3.84E-07	2.46E-05	-10.6121
172	0.05875	0.03225	4.03E-07	2.46E-05	-10.6129
174	0.05925	0.0325	4.06E-07	2.46E-05	-10.613
176	0.059	0.03125	3.91E-07	2.46E-05	-10.6124
178	0.05925	0.03325	4.16E-07	2.46E-05	-10.6134
180	0.059	0.032	4E-07	2.46E-05	-10.6128
182	0.0595	0.0315	3.94E-07	2.46E-05	-10.6125
184	0.06075	0.0335	4.19E-07	2.46E-05	-10.6135
186	0.06125	0.0345	4.31E-07	2.46E-05	-10.614
188	0.062	0.035	4.38E-07	2.46E-05	-10.6143
190	0.0615	0.03475	4.34E-07	2.46E-05	-10.6142
192	0.06225	0.03575	4.47E-07	2.46E-05	-10.6147
194	0.06275	0.0355	4.44E-07	2.46E-05	-10.6145
196	0.0635	0.036	4.5E-07	2.46E-05	-10.6148
198	0.06425	0.0375	4.69E-07	2.45E-05	-10.6156
200	0.065	0.03825	4.78E-07	2.45E-05	-10.6159
202	0.065	0.03825	4.78E-07	2.45E-05	-10.6159
204	0.06475	0.03725	4.66E-07	2.45E-05	-10.6154
206	0.06575	0.0385	4.81E-07	2.45E-05	-10.6161
208	0.06575	0.0385	4.81E-07	2.45E-05	-10.6161
210	0.066	0.039	4.88E-07	2.45E-05	-10.6163
212	0.06675	0.03875	4.84E-07	2.45E-05	-10.6162
214	0.06825	0.04075	5.09E-07	2.45E-05	-10.6172
216	0.06775	0.04	5E-07	2.45E-05	-10.6168
218	0.06875	0.04025	5.03E-07	2.45E-05	-10.617
220	0.069	0.041	5.13E-07	2.45E-05	-10.6173
222	0.06925	0.041	5.13E-07	2.45E-05	-10.6173
224	0.06925	0.04225	5.28E-07	2.45E-05	-10.618
226	0.0705	0.043	5.38E-07	2.45E-05	-10.6184
228	0.07	0.04275	5.34E-07	2.45E-05	-10.6182
230	0.07025	0.04275	5.34E-07	2.45E-05	-10.6182
232	0.071	0.04275	5.34E-07	2.45E-05	-10.6182

234	0.0705	0.04275	5.34E-07	2.45E-05	-10.6182
236	0.0715	0.0445	5.56E-07	2.44E-05	-10.6191
238	0.071	0.04325	5.41E-07	2.45E-05	-10.6185
240	0.07125	0.04275	5.34E-07	2.45E-05	-10.6182
242	0.07325	0.045	5.63E-07	2.44E-05	-10.6194
244	0.072	0.0435	5.44E-07	2.45E-05	-10.6186
246	0.073	0.0445	5.56E-07	2.44E-05	-10.6191
248	0.0735	0.0445	5.56E-07	2.44E-05	-10.6191
250	0.07425	0.046	5.75E-07	2.44E-05	-10.6199
252	0.07425	0.04575	5.72E-07	2.44E-05	-10.6198
254	0.07475	0.046	5.75E-07	2.44E-05	-10.6199
256	0.075	0.04575	5.72E-07	2.44E-05	-10.6198
258	0.07525	0.0465	5.81E-07	2.44E-05	-10.6202
260	0.0755	0.047	5.88E-07	2.44E-05	-10.6204
262	0.076	0.0465	5.81E-07	2.44E-05	-10.6202
264	0.0765	0.0475	5.94E-07	2.44E-05	-10.6207
266	0.07625	0.0485	6.06E-07	2.44E-05	-10.6212
268	0.07675	0.048	6E-07	2.44E-05	-10.6209
270	0.0775	0.04925	6.16E-07	2.44E-05	-10.6216
272	0.078	0.04775	5.97E-07	2.44E-05	-10.6208
274	0.078	0.04725	5.91E-07	2.44E-05	-10.6205
276	0.078	0.04825	6.03E-07	2.44E-05	-10.6211
278	0.0795	0.05	6.25E-07	2.44E-05	-10.622
280	0.079	0.04925	6.16E-07	2.44E-05	-10.6216
282	0.0805	0.05125	6.41E-07	2.44E-05	-10.6226
284	0.0805	0.051	6.38E-07	2.44E-05	-10.6225
286	0.07925	0.04925	6.16E-07	2.44E-05	-10.6216
288	0.0805	0.0505	6.31E-07	2.44E-05	-10.6222
290	0.081	0.0515	6.44E-07	2.44E-05	-10.6227
292	0.08175	0.05175	6.47E-07	2.44E-05	-10.6229
294	0.0815	0.052	6.5E-07	2.44E-05	-10.623
296	0.0815	0.052	6.5E-07	2.44E-05	-10.623
298	0.08125	0.05075	6.34E-07	2.44E-05	-10.6223

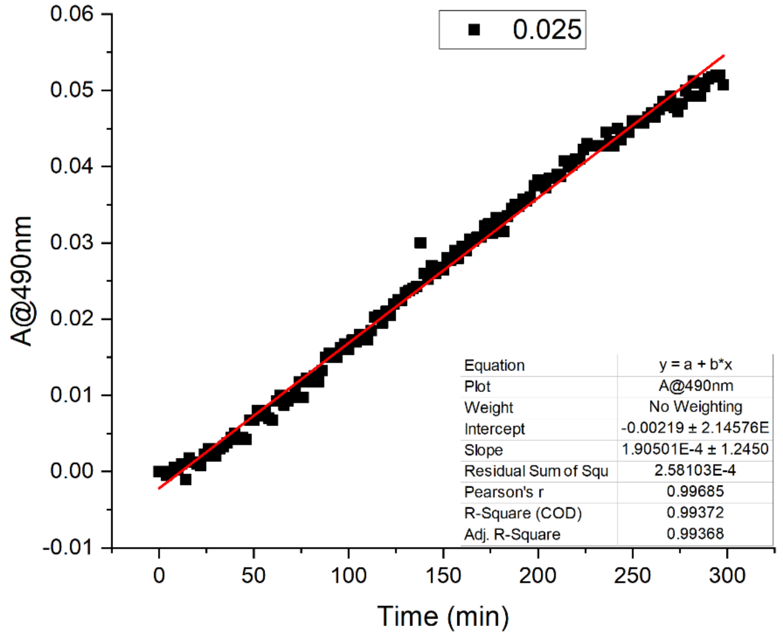

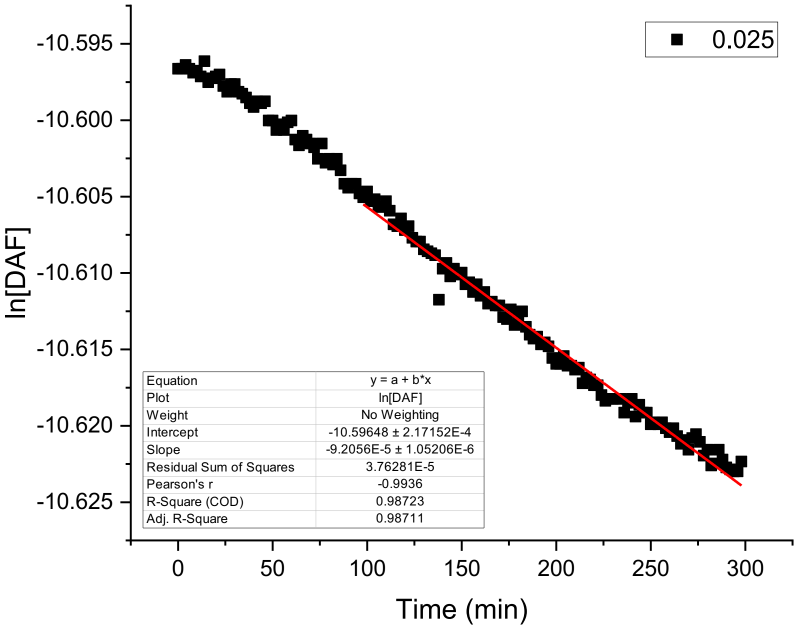

0.050 mM **DAF**

Time (min)	A@490nm minus BK	set to 0	[FLU] (M)	[DAF] (M)	ln[DAF]
0	0.0255	0	0	0.00005	-9.90349
2	0.02675	0	0	0.00005	-9.90349
4	0.02425	-0.003	-3.8E-08	5E-05	-9.90274
6	0.02475	-0.0025	-3.1E-08	5E-05	-9.90286
8	0.02575	-0.0025	-3.1E-08	5E-05	-9.90286
10	0.0255	-0.001	-1.3E-08	5E-05	-9.90324

12	0.026	-7.50E-04	-9.4E-09	5E-05	-9.9033
14	0.02625	-7.50E-04	-9.4E-09	5E-05	-9.9033
16	0.02775	5.00E-04	6.25E-09	5E-05	-9.90361
18	0.02725	2.50E-04	3.13E-09	5E-05	-9.90355
20	0.0285	5.00E-04	6.25E-09	5E-05	-9.90361
22	0.02775	0.001	1.25E-08	5E-05	-9.90374
24	0.028	7.50E-04	9.38E-09	5E-05	-9.90368
26	0.03	0.0015	1.88E-08	5E-05	-9.90386
28	0.03025	0.0025	3.13E-08	5E-05	-9.90411
30	0.031	0.0035	4.38E-08	5E-05	-9.90436
32	0.031	0.00375	4.69E-08	5E-05	-9.90443
34	0.032	0.00425	5.31E-08	4.99E-05	-9.90455
36	0.032	0.00425	5.31E-08	4.99E-05	-9.90455
38	0.033	0.00525	6.56E-08	4.99E-05	-9.9048
40	0.033	0.00475	5.94E-08	4.99E-05	-9.90468
42	0.03375	0.00425	5.31E-08	4.99E-05	-9.90455
44	0.0345	0.00525	6.56E-08	4.99E-05	-9.9048
46	0.0345	0.005	6.25E-08	4.99E-05	-9.90474
48	0.03725	0.0085	1.06E-07	4.99E-05	-9.90561
50	0.03775	0.00975	1.22E-07	4.99E-05	-9.90593
52	0.037	0.01	1.25E-07	4.99E-05	-9.90599
54	0.0385	0.01	1.25E-07	4.99E-05	-9.90599
56	0.03975	0.01175	1.47E-07	4.99E-05	-9.90643
58	0.03975	0.01075	1.34E-07	4.99E-05	-9.90618
60	0.03925	0.011	1.38E-07	4.99E-05	-9.90624
62	0.0405	0.0115	1.44E-07	4.99E-05	-9.90637
64	0.0415	0.013	1.63E-07	4.98E-05	-9.90674
66	0.0425	0.013	1.63E-07	4.98E-05	-9.90674
68	0.04325	0.0135	1.69E-07	4.98E-05	-9.90687
70	0.04425	0.01325	1.66E-07	4.98E-05	-9.90681
72	0.0455	0.016	2E-07	4.98E-05	-9.9075
74	0.0465	0.01675	2.09E-07	4.98E-05	-9.90768
76	0.0465	0.0155	1.94E-07	4.98E-05	-9.90737
78	0.048	0.0175	2.19E-07	4.98E-05	-9.90787
80	0.04775	0.015	1.88E-07	4.98E-05	-9.90724
82	0.04875	0.01725	2.16E-07	4.98E-05	-9.90781
84	0.04725	0.01425	1.78E-07	4.98E-05	-9.90706
86	0.05	0.019	2.38E-07	4.98E-05	-9.90825
88	0.05175	0.02025	2.53E-07	4.97E-05	-9.90856
90	0.0525	0.0235	2.94E-07	4.97E-05	-9.90938
92	0.05275	0.0225	2.81E-07	4.97E-05	-9.90913
94	0.053	0.02275	2.84E-07	4.97E-05	-9.90919
96	0.0545	0.02425	3.03E-07	4.97E-05	-9.90957
98	0.055	0.02425	3.03E-07	4.97E-05	-9.90957
100	0.056	0.025	3.13E-07	4.97E-05	-9.90976
102	0.0575	0.0265	3.31E-07	4.97E-05	-9.91013

104	0.05725	0.02625	3.28E-07	4.97E-05	-9.91007
106	0.05825	0.02775	3.47E-07	4.97E-05	-9.91045
108	0.06	0.02925	3.66E-07	4.96E-05	-9.91083
110	0.0595	0.028	3.5E-07	4.97E-05	-9.91051
112	0.0615	0.03125	3.91E-07	4.96E-05	-9.91133
114	0.06175	0.02975	3.72E-07	4.96E-05	-9.91095
116	0.06275	0.0315	3.94E-07	4.96E-05	-9.91139
118	0.06375	0.0325	4.06E-07	4.96E-05	-9.91165
120	0.0645	0.03275	4.09E-07	4.96E-05	-9.91171
122	0.06625	0.0335	4.19E-07	4.96E-05	-9.9119
124	0.0665	0.035	4.38E-07	4.96E-05	-9.91228
126	0.06775	0.03525	4.41E-07	4.96E-05	-9.91234
128	0.0685	0.03625	4.53E-07	4.95E-05	-9.91259
130	0.0705	0.0375	4.69E-07	4.95E-05	-9.91291
132	0.0705	0.03775	4.72E-07	4.95E-05	-9.91297
134	0.072	0.0395	4.94E-07	4.95E-05	-9.91341
136	0.07125	0.038	4.75E-07	4.95E-05	-9.91303
138	0.07325	0.045	5.63E-07	4.94E-05	-9.9148
140	0.07375	0.04	5E-07	4.95E-05	-9.91354
142	0.075	0.0425	5.31E-07	4.95E-05	-9.91417
144	0.0765	0.042	5.25E-07	4.95E-05	-9.91404
146	0.077	0.0435	5.44E-07	4.95E-05	-9.91442
148	0.0775	0.044	5.5E-07	4.95E-05	-9.91455
150	0.0785	0.04575	5.72E-07	4.94E-05	-9.91499
152	0.07925	0.0465	5.81E-07	4.94E-05	-9.91518
154	0.08025	0.0465	5.81E-07	4.94E-05	-9.91518
156	0.081	0.0465	5.81E-07	4.94E-05	-9.91518
158	0.08225	0.04675	5.84E-07	4.94E-05	-9.91524
160	0.08275	0.04675	5.84E-07	4.94E-05	-9.91524
162	0.08325	0.048	6E-07	4.94E-05	-9.91556
164	0.0855	0.0505	6.31E-07	4.94E-05	-9.91619
166	0.0855	0.05075	6.34E-07	4.94E-05	-9.91626
168	0.0875	0.05275	6.59E-07	4.93E-05	-9.91676
170	0.0885	0.05375	6.72E-07	4.93E-05	-9.91702
172	0.08725	0.05025	6.28E-07	4.94E-05	-9.91613
174	0.09025	0.0545	6.81E-07	4.93E-05	-9.91721
176	0.0905	0.05375	6.72E-07	4.93E-05	-9.91702
178	0.0905	0.0535	6.69E-07	4.93E-05	-9.91695
180	0.0895	0.05275	6.59E-07	4.93E-05	-9.91676
182	0.09225	0.05425	6.78E-07	4.93E-05	-9.91714
184	0.0945	0.05725	7.16E-07	4.93E-05	-9.9179
186	0.09525	0.05775	7.22E-07	4.93E-05	-9.91803
188	0.09575	0.0585	7.31E-07	4.93E-05	-9.91822
190	0.09625	0.05975	7.47E-07	4.93E-05	-9.91854
192	0.09725	0.06025	7.53E-07	4.92E-05	-9.91866
194	0.09875	0.0605	7.56E-07	4.92E-05	-9.91873

196	0.09925	0.061	7.63E-07	4.92E-05	-9.91886
198	0.10125	0.06525	8.16E-07	4.92E-05	-9.91993
200	0.10175	0.06425	8.03E-07	4.92E-05	-9.91968
202	0.1025	0.065	8.13E-07	4.92E-05	-9.91987
204	0.10275	0.0655	8.19E-07	4.92E-05	-9.92
206	0.10475	0.06725	8.41E-07	4.92E-05	-9.92044
208	0.10475	0.06725	8.41E-07	4.92E-05	-9.92044
210	0.1065	0.06825	8.53E-07	4.91E-05	-9.9207
212	0.10625	0.068	8.5E-07	4.92E-05	-9.92063
214	0.10825	0.06975	8.72E-07	4.91E-05	-9.92108
216	0.1085	0.06975	8.72E-07	4.91E-05	-9.92108
218	0.109	0.06925	8.66E-07	4.91E-05	-9.92095
220	0.1115	0.07225	9.03E-07	4.91E-05	-9.92172
222	0.112	0.07075	8.84E-07	4.91E-05	-9.92133
224	0.113	0.073	9.13E-07	4.91E-05	-9.92191
226	0.1135	0.0725	9.06E-07	4.91E-05	-9.92178
228	0.114	0.07275	9.09E-07	4.91E-05	-9.92184
230	0.115	0.07425	9.28E-07	4.91E-05	-9.92222
232	0.116	0.07375	9.22E-07	4.91E-05	-9.9221
234	0.11575	0.073	9.13E-07	4.91E-05	-9.92191
236	0.11775	0.0775	9.69E-07	4.9E-05	-9.92305
238	0.1175	0.07725	9.66E-07	4.9E-05	-9.92299
240	0.1185	0.076	9.5E-07	4.91E-05	-9.92267
242	0.1195	0.07875	9.84E-07	4.9E-05	-9.92337
244	0.1205	0.08025	1E-06	4.9E-05	-9.92375
246	0.122	0.0815	1.02E-06	4.9E-05	-9.92407
248	0.123	0.08175	1.02E-06	4.9E-05	-9.92414
250	0.1235	0.08225	1.03E-06	4.9E-05	-9.92426
252	0.12525	0.08425	1.05E-06	4.89E-05	-9.92478
254	0.1255	0.08425	1.05E-06	4.89E-05	-9.92478
256	0.12625	0.083	1.04E-06	4.9E-05	-9.92446
258	0.127	0.08425	1.05E-06	4.89E-05	-9.92478
260	0.127	0.084	1.05E-06	4.9E-05	-9.92471
262	0.12825	0.083	1.04E-06	4.9E-05	-9.92446
264	0.12975	0.08625	1.08E-06	4.89E-05	-9.92529
266	0.12975	0.08775	1.1E-06	4.89E-05	-9.92567
268	0.131	0.0865	1.08E-06	4.89E-05	-9.92535
270	0.1325	0.08825	1.1E-06	4.89E-05	-9.9258
272	0.133	0.089	1.11E-06	4.89E-05	-9.92599
274	0.13375	0.0895	1.12E-06	4.89E-05	-9.92612
276	0.13425	0.09025	1.13E-06	4.89E-05	-9.92631
278	0.13575	0.09125	1.14E-06	4.89E-05	-9.92656
280	0.138	0.0935	1.17E-06	4.88E-05	-9.92714
282	0.138	0.09375	1.17E-06	4.88E-05	-9.9272
284	0.1385	0.0925	1.16E-06	4.88E-05	-9.92688
286	0.1385	0.09275	1.16E-06	4.88E-05	-9.92695

288	0.13925	0.092	1.15E-06	4.89E-05	-9.92676
290	0.14025	0.0955	1.19E-06	4.88E-05	-9.92765
292	0.142	0.09475	1.18E-06	4.88E-05	-9.92746
294	0.142	0.0955	1.19E-06	4.88E-05	-9.92765
296	0.142	0.095	1.19E-06	4.88E-05	-9.92752
298	0.144	0.09725	1.22E-06	4.88E-05	-9.9281

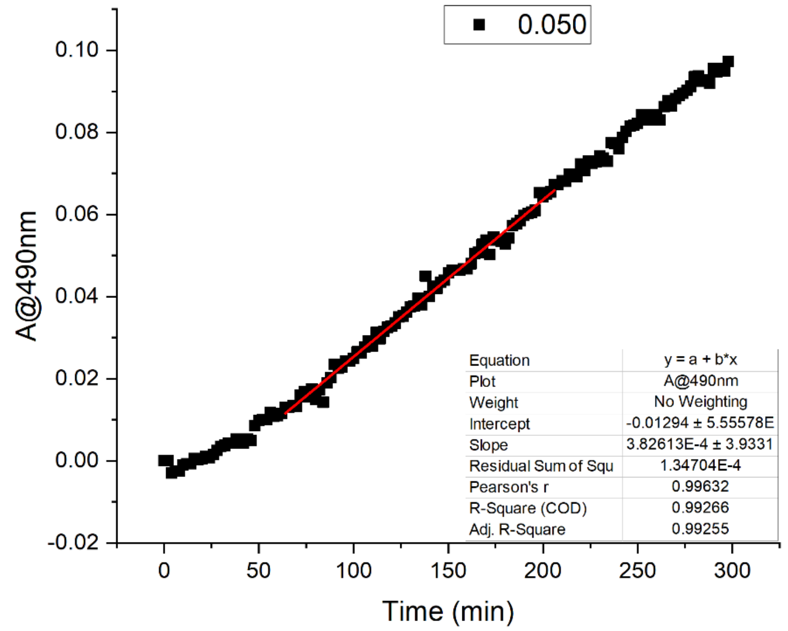

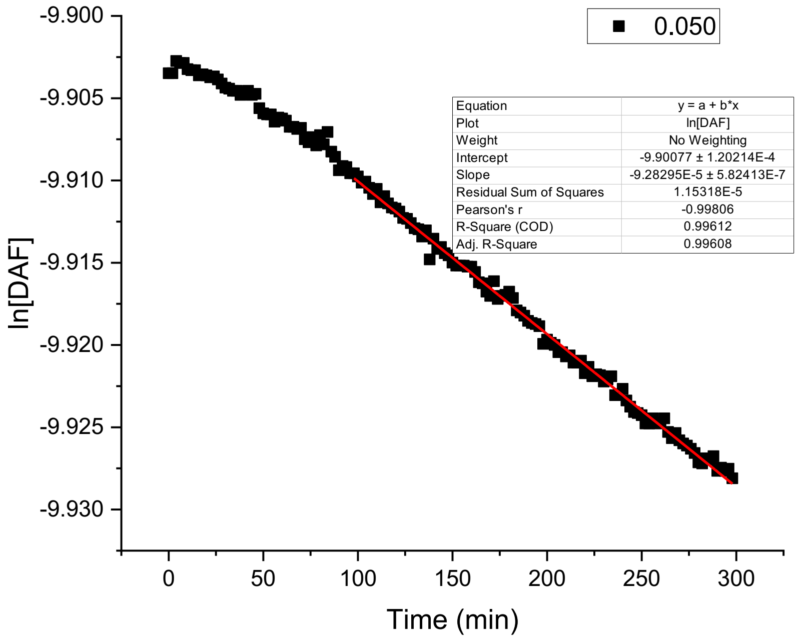

0.075 mM **DAF**

Time (min)	A@490nm minus BK	set to 0	[FLU] (M)	[DAF] (M)	ln[DAF]
0	0.026	0	0	0.000075	-9.49802
2	0.02525	0	0	0.000075	-9.49802
4	0.02575	0.00125	1.56E-08	7.5E-05	-9.49823
6	0.0255	0.001	1.25E-08	7.5E-05	-9.49819
8	0.02525	0.001	1.25E-08	7.5E-05	-9.49819
10	0.025	-0.0015	-1.9E-08	7.5E-05	-9.49777
12	0.0255	7.50E-04	9.38E-09	7.5E-05	-9.49815
14	0.02575	2.50E-04	3.13E-09	7.5E-05	-9.49806
16	0.02675	0.00225	2.81E-08	7.5E-05	-9.4984
18	0.0255	0	0	0.000075	-9.49802
20	0.02725	0.0025	3.13E-08	7.5E-05	-9.49844
22	0.02675	0.002	2.5E-08	7.5E-05	-9.49836
24	0.02775	0.0035	4.38E-08	7.5E-05	-9.49861
26	0.02925	0.0045	5.63E-08	7.49E-05	-9.49877
28	0.02925	0.003	3.75E-08	7.5E-05	-9.49852
30	0.03	0.004	5E-08	7.5E-05	-9.49869
32	0.0295	0.00175	2.19E-08	7.5E-05	-9.49831
34	0.0315	0.004	5E-08	7.5E-05	-9.49869
36	0.03125	0.0035	4.38E-08	7.5E-05	-9.49861
38	0.03225	0.00525	6.56E-08	7.49E-05	-9.4989
40	0.032	0.006	7.5E-08	7.49E-05	-9.49902
42	0.03375	0.00675	8.44E-08	7.49E-05	-9.49915
44	0.0345	0.00775	9.69E-08	7.49E-05	-9.49931
46	0.0345	0.0065	8.13E-08	7.49E-05	-9.49911
48	0.0365	0.009	1.13E-07	7.49E-05	-9.49952
50	0.0375	0.00975	1.22E-07	7.49E-05	-9.49965
52	0.03675	0.0105	1.31E-07	7.49E-05	-9.49977
54	0.0395	0.01075	1.34E-07	7.49E-05	-9.49982
56	0.03875	0.011	1.38E-07	7.49E-05	-9.49986
58	0.04	0.01125	1.41E-07	7.49E-05	-9.4999
60	0.04025	0.01125	1.41E-07	7.49E-05	-9.4999
62	0.042	0.01375	1.72E-07	7.48E-05	-9.50032
64	0.04225	0.0145	1.81E-07	7.48E-05	-9.50044
66	0.043	0.01525	1.91E-07	7.48E-05	-9.50057
68	0.044	0.016	2E-07	7.48E-05	-9.50069
70	0.04425	0.0155	1.94E-07	7.48E-05	-9.50061
72	0.04575	0.01625	2.03E-07	7.48E-05	-9.50073
74	0.04625	0.0175	2.19E-07	7.48E-05	-9.50094
76	0.04775	0.01875	2.34E-07	7.48E-05	-9.50115
78	0.04925	0.01975	2.47E-07	7.48E-05	-9.50132
80	0.049	0.019	2.38E-07	7.48E-05	-9.50119
82	0.04975	0.02025	2.53E-07	7.47E-05	-9.5014
84	0.04975	0.0195	2.44E-07	7.48E-05	-9.50128
86	0.05275	0.02225	2.78E-07	7.47E-05	-9.50174

88	0.05325	0.023	2.88E-07	7.47E-05	-9.50186
90	0.05475	0.02525	3.16E-07	7.47E-05	-9.50224
92	0.05525	0.02475	3.09E-07	7.47E-05	-9.50216
94	0.05625	0.0255	3.19E-07	7.47E-05	-9.50228
96	0.0575	0.02725	3.41E-07	7.47E-05	-9.50257
98	0.05825	0.028	3.5E-07	7.47E-05	-9.5027
100	0.061	0.02975	3.72E-07	7.46E-05	-9.50299
102	0.06025	0.029	3.63E-07	7.46E-05	-9.50287
104	0.06125	0.0295	3.69E-07	7.46E-05	-9.50295
106	0.062	0.0315	3.94E-07	7.46E-05	-9.50329
108	0.063	0.03125	3.91E-07	7.46E-05	-9.50324
110	0.06325	0.03	3.75E-07	7.46E-05	-9.50303
112	0.06475	0.033	4.13E-07	7.46E-05	-9.50354
114	0.06625	0.0345	4.31E-07	7.46E-05	-9.50379
116	0.06725	0.03525	4.41E-07	7.46E-05	-9.50391
118	0.068	0.0355	4.44E-07	7.46E-05	-9.50396
120	0.07	0.038	4.75E-07	7.45E-05	-9.50438
122	0.07125	0.0385	4.81E-07	7.45E-05	-9.50446
124	0.07175	0.03975	4.97E-07	7.45E-05	-9.50467
126	0.073	0.04025	5.03E-07	7.45E-05	-9.50475
128	0.074	0.0415	5.19E-07	7.45E-05	-9.50496
130	0.0755	0.04325	5.41E-07	7.45E-05	-9.50526
132	0.07575	0.043	5.38E-07	7.45E-05	-9.50521
134	0.07775	0.044	5.5E-07	7.45E-05	-9.50538
136	0.07825	0.0455	5.69E-07	7.44E-05	-9.50563
138	0.08	0.0515	6.44E-07	7.44E-05	-9.50664
140	0.08025	0.049	6.13E-07	7.44E-05	-9.50622
142	0.08175	0.05	6.25E-07	7.44E-05	-9.50639
144	0.082	0.0505	6.31E-07	7.44E-05	-9.50647
146	0.08325	0.0515	6.44E-07	7.44E-05	-9.50664
148	0.08475	0.052	6.5E-07	7.44E-05	-9.50673
150	0.08575	0.0535	6.69E-07	7.43E-05	-9.50698
152	0.08625	0.0545	6.81E-07	7.43E-05	-9.50715
154	0.08775	0.05475	6.84E-07	7.43E-05	-9.50719
156	0.08875	0.05575	6.97E-07	7.43E-05	-9.50736
158	0.0905	0.05625	7.03E-07	7.43E-05	-9.50744
160	0.091	0.057	7.13E-07	7.43E-05	-9.50757
162	0.0915	0.05725	7.16E-07	7.43E-05	-9.50761
164	0.09325	0.05875	7.34E-07	7.43E-05	-9.50786
166	0.09425	0.06075	7.59E-07	7.42E-05	-9.5082
168	0.0955	0.06125	7.66E-07	7.42E-05	-9.50828
170	0.097	0.0625	7.81E-07	7.42E-05	-9.50849
172	0.097	0.06325	7.91E-07	7.42E-05	-9.50862
174	0.09925	0.06375	7.97E-07	7.42E-05	-9.5087
176	0.09975	0.06425	8.03E-07	7.42E-05	-9.50879
178	0.1005	0.06675	8.34E-07	7.42E-05	-9.50921

180	0.1	0.06475	8.09E-07	7.42E-05	-9.50887
182	0.102	0.0665	8.31E-07	7.42E-05	-9.50917
184	0.10375	0.06825	8.53E-07	7.41E-05	-9.50946
186	0.105	0.07	8.75E-07	7.41E-05	-9.50976
188	0.1065	0.071	8.88E-07	7.41E-05	-9.50993
190	0.107	0.072	9E-07	7.41E-05	-9.5101
192	0.10875	0.0725	9.06E-07	7.41E-05	-9.51018
194	0.10975	0.07475	9.34E-07	7.41E-05	-9.51056
196	0.11075	0.075	9.38E-07	7.41E-05	-9.5106
198	0.1125	0.07525	9.41E-07	7.41E-05	-9.51064
200	0.113	0.07725	9.66E-07	7.4E-05	-9.51098
202	0.1145	0.079	9.88E-07	7.4E-05	-9.51128
204	0.11525	0.07925	9.91E-07	7.4E-05	-9.51132
206	0.1165	0.07975	9.97E-07	7.4E-05	-9.5114
208	0.1165	0.07875	9.84E-07	7.4E-05	-9.51123
210	0.118	0.0805	1.01E-06	7.4E-05	-9.51153
212	0.11975	0.08225	1.03E-06	7.4E-05	-9.51183
214	0.121	0.08425	1.05E-06	7.39E-05	-9.51216
216	0.12125	0.08425	1.05E-06	7.39E-05	-9.51216
218	0.12275	0.08525	1.07E-06	7.39E-05	-9.51233
220	0.12425	0.087	1.09E-06	7.39E-05	-9.51263
222	0.1255	0.0875	1.09E-06	7.39E-05	-9.51271
224	0.126	0.08825	1.1E-06	7.39E-05	-9.51284
226	0.128	0.09	1.13E-06	7.39E-05	-9.51314
228	0.1285	0.09	1.13E-06	7.39E-05	-9.51314
230	0.12875	0.0905	1.13E-06	7.39E-05	-9.51322
232	0.13075	0.09175	1.15E-06	7.39E-05	-9.51343
234	0.13075	0.091	1.14E-06	7.39E-05	-9.51331
236	0.1325	0.094	1.18E-06	7.38E-05	-9.51381
238	0.133	0.09275	1.16E-06	7.38E-05	-9.5136
240	0.1345	0.09475	1.18E-06	7.38E-05	-9.51394
242	0.13525	0.0945	1.18E-06	7.38E-05	-9.5139
244	0.13575	0.096	1.2E-06	7.38E-05	-9.51415
246	0.13825	0.09875	1.23E-06	7.38E-05	-9.51462
248	0.139	0.09825	1.23E-06	7.38E-05	-9.51453
250	0.14	0.1	1.25E-06	7.38E-05	-9.51483
252	0.142	0.1015	1.27E-06	7.37E-05	-9.51508
254	0.14225	0.10175	1.27E-06	7.37E-05	-9.51513
256	0.144	0.10175	1.27E-06	7.37E-05	-9.51513
258	0.14375	0.101	1.26E-06	7.37E-05	-9.515
260	0.145	0.102	1.28E-06	7.37E-05	-9.51517
262	0.14575	0.10175	1.27E-06	7.37E-05	-9.51513
264	0.14825	0.104	1.3E-06	7.37E-05	-9.51551
266	0.148	0.106	1.33E-06	7.37E-05	-9.51585
268	0.14975	0.10575	1.32E-06	7.37E-05	-9.5158
270	0.15075	0.107	1.34E-06	7.37E-05	-9.51602

272	0.155	0.11	1.38E-06	7.36E-05	-9.51653
274	0.15275	0.108	1.35E-06	7.37E-05	-9.51619
276	0.154	0.11125	1.39E-06	7.36E-05	-9.51674
278	0.1545	0.1095	1.37E-06	7.36E-05	-9.51644
280	0.16	0.11475	1.43E-06	7.36E-05	-9.51733
282	0.15925	0.1145	1.43E-06	7.36E-05	-9.51729
284	0.1585	0.114	1.43E-06	7.36E-05	-9.51721
286	0.15925	0.11425	1.43E-06	7.36E-05	-9.51725
288	0.16075	0.114	1.43E-06	7.36E-05	-9.51721
290	0.162	0.11675	1.46E-06	7.35E-05	-9.51767
292	0.16225	0.1155	1.44E-06	7.36E-05	-9.51746
294	0.1645	0.11775	1.47E-06	7.35E-05	-9.51784
296	0.1645	0.11725	1.47E-06	7.35E-05	-9.51776
298	0.16625	0.11825	1.48E-06	7.35E-05	-9.51793

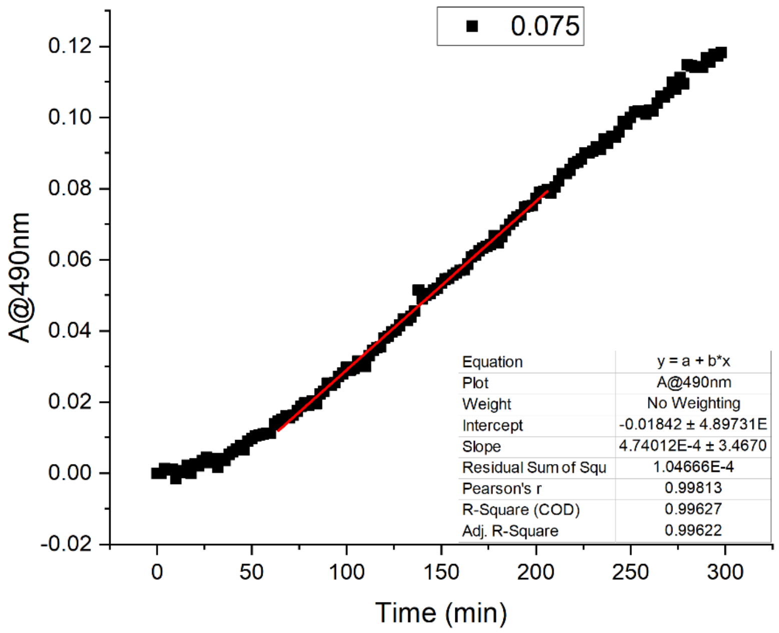

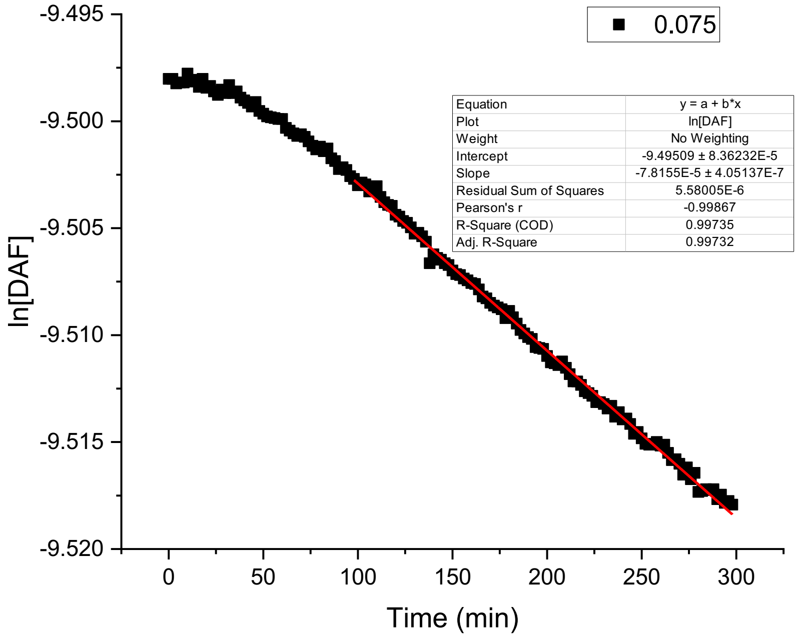

0.100 mM **DAF**

Time (min)	A@490nm minus BK	set to 0	[FLU] (M)	[DAF] (M)	ln[DAF]
0	0.01	0	0	0.0001	-9.21034
2	0.009	-0.001	-1.3E-08	0.0001	-9.21022
4	0.00875	-0.00125	-1.6E-08	0.0001	-9.21018
6	0.00875	-0.00125	-1.6E-08	0.0001	-9.21018
8	0.01025	0.00025	3.12E-09	1E-04	-9.21037
10	0.0095	-0.0005	-6.2E-09	0.0001	-9.21028
12	0.0095	-0.0005	-6.3E-09	0.0001	-9.21028
14	0.0085	-0.0015	-1.9E-08	0.0001	-9.21015
16	0.0105	0.0005	6.25E-09	1E-04	-9.2104
18	0.00975	-0.00025	-3.1E-09	0.0001	-9.21031
20	0.0105	0.0005	6.25E-09	1E-04	-9.2104
22	0.0105	0.0005	6.25E-09	1E-04	-9.2104
24	0.011	0.001	1.25E-08	1E-04	-9.21047
26	0.0115	0.0015	1.88E-08	1E-04	-9.21053
28	0.01125	0.00125	1.56E-08	1E-04	-9.2105
30	0.01175	0.00175	2.19E-08	1E-04	-9.21056
32	0.011	0.001	1.25E-08	1E-04	-9.21047
34	0.013	0.003	3.75E-08	1E-04	-9.21072
36	0.0125	0.0025	3.13E-08	1E-04	-9.21065
38	0.0135	0.0035	4.38E-08	1E-04	-9.21078
40	0.01175	0.00175	2.19E-08	1E-04	-9.21056
42	0.00125	-0.00875	-1.1E-07	0.0001	-9.20925
44	0.01375	0.00375	4.69E-08	1E-04	-9.21081
46	0.013	0.003	3.75E-08	1E-04	-9.21072
48	0.017	0.007	8.75E-08	9.99E-05	-9.21122

50	0.01675	0.00675	8.44E-08	9.99E-05	-9.21118
52	0.018	0.008	1E-07	9.99E-05	-9.21134
54	0.0185	0.0085	1.06E-07	9.99E-05	-9.2114
56	0.019	0.009	1.13E-07	9.99E-05	-9.21147
58	0.019	0.009	1.13E-07	9.99E-05	-9.21147
60	0.018	0.008	1E-07	9.99E-05	-9.21134
62	0.02075	0.01075	1.34E-07	9.99E-05	-9.21169
64	0.02	0.01	1.25E-07	9.99E-05	-9.21159
66	0.022	0.012	1.5E-07	9.99E-05	-9.21184
68	0.0225	0.0125	1.56E-07	9.98E-05	-9.2119
70	0.02325	0.01325	1.66E-07	9.98E-05	-9.212
72	0.024	0.014	1.75E-07	9.98E-05	-9.21209
74	0.0255	0.0155	1.94E-07	9.98E-05	-9.21228
76	0.0265	0.0165	2.06E-07	9.98E-05	-9.21241
78	0.02175	0.01175	1.47E-07	9.99E-05	-9.21181
80	0.0255	0.0155	1.94E-07	9.98E-05	-9.21228
82	0.027	0.017	2.13E-07	9.98E-05	-9.21247
84	0.02625	0.01625	2.03E-07	9.98E-05	-9.21237
86	0.0285	0.0185	2.31E-07	9.98E-05	-9.21266
88	0.0305	0.0205	2.56E-07	9.97E-05	-9.21291
90	0.03375	0.02375	2.97E-07	9.97E-05	-9.21331
92	0.03375	0.02375	2.97E-07	9.97E-05	-9.21331
94	0.0345	0.0245	3.06E-07	9.97E-05	-9.21341
96	0.0365	0.0265	3.31E-07	9.97E-05	-9.21366
98	0.0365	0.0265	3.31E-07	9.97E-05	-9.21366
100	0.037	0.027	3.38E-07	9.97E-05	-9.21372
102	0.0385	0.0285	3.56E-07	9.96E-05	-9.21391
104	0.0385	0.0285	3.56E-07	9.96E-05	-9.21391
106	0.04025	0.03025	3.78E-07	9.96E-05	-9.21413
108	0.04075	0.03075	3.84E-07	9.96E-05	-9.21419
110	0.04075	0.03075	3.84E-07	9.96E-05	-9.21419
112	0.04175	0.03175	3.97E-07	9.96E-05	-9.21432
114	0.044	0.034	4.25E-07	9.96E-05	-9.2146
116	0.0435	0.0335	4.19E-07	9.96E-05	-9.21454
118	0.04375	0.03375	4.22E-07	9.96E-05	-9.21457
120	0.0465	0.0365	4.56E-07	9.95E-05	-9.21491
122	0.047	0.037	4.63E-07	9.95E-05	-9.21498
124	0.0485	0.0385	4.81E-07	9.95E-05	-9.21516
126	0.0495	0.0395	4.94E-07	9.95E-05	-9.21529
128	0.0505	0.0405	5.06E-07	9.95E-05	-9.21542
130	0.051	0.041	5.13E-07	9.95E-05	-9.21548
132	0.053	0.043	5.38E-07	9.95E-05	-9.21573
134	0.053	0.043	5.38E-07	9.95E-05	-9.21573
136	0.054	0.044	5.5E-07	9.95E-05	-9.21586
138	0.06	0.05	6.25E-07	9.94E-05	-9.21661
140	0.05675	0.04675	5.84E-07	9.94E-05	-9.2162

142	0.057	0.047	5.88E-07	9.94E-05	-9.21623
144	0.058	0.048	6E-07	9.94E-05	-9.21636
146	0.0595	0.0495	6.19E-07	9.94E-05	-9.21655
148	0.06025	0.05025	6.28E-07	9.94E-05	-9.21664
150	0.04625	0.03625	4.53E-07	9.95E-05	-9.21488
152	0.0625	0.0525	6.56E-07	9.93E-05	-9.21692
154	0.063	0.053	6.63E-07	9.93E-05	-9.21699
156	0.0645	0.0545	6.81E-07	9.93E-05	-9.21718
158	0.06675	0.05675	7.09E-07	9.93E-05	-9.21746
160	0.06525	0.05525	6.91E-07	9.93E-05	-9.21727
162	0.0665	0.0565	7.06E-07	9.93E-05	-9.21743
164	0.06825	0.05825	7.28E-07	9.93E-05	-9.21765
166	0.06975	0.05975	7.47E-07	9.93E-05	-9.21784
168	0.07025	0.06025	7.53E-07	9.92E-05	-9.2179
170	0.072	0.062	7.75E-07	9.92E-05	-9.21812
172	0.07175	0.06175	7.72E-07	9.92E-05	-9.21809
174	0.07425	0.06425	8.03E-07	9.92E-05	-9.2184
176	0.0745	0.0645	8.06E-07	9.92E-05	-9.21844
178	0.0765	0.0665	8.31E-07	9.92E-05	-9.21869
180	0.07525	0.06525	8.16E-07	9.92E-05	-9.21853
182	0.077	0.067	8.38E-07	9.92E-05	-9.21875
184	0.07975	0.06975	8.72E-07	9.91E-05	-9.2191
186	0.08025	0.07025	8.78E-07	9.91E-05	-9.21916
188	0.0815	0.0715	8.94E-07	9.91E-05	-9.21932
190	0.082	0.072	9E-07	9.91E-05	-9.21938
192	0.08325	0.07325	9.16E-07	9.91E-05	-9.21954
194	0.08325	0.07325	9.16E-07	9.91E-05	-9.21954
196	0.084	0.074	9.25E-07	9.91E-05	-9.21963
198	0.08675	0.07675	9.59E-07	9.9E-05	-9.21998
200	0.0865	0.0765	9.56E-07	9.9E-05	-9.21995
202	0.089	0.079	9.88E-07	9.9E-05	-9.22026
204	0.0895	0.0795	9.94E-07	9.9E-05	-9.22033
206	0.09025	0.08025	1E-06	9.9E-05	-9.22042
208	0.09125	0.08125	1.02E-06	9.9E-05	-9.22055
210	0.0925	0.0825	1.03E-06	9.9E-05	-9.22071
212	0.09225	0.08225	1.03E-06	9.9E-05	-9.22067
214	0.0945	0.0845	1.06E-06	9.89E-05	-9.22096
216	0.096	0.086	1.08E-06	9.89E-05	-9.22115
218	0.09625	0.08625	1.08E-06	9.89E-05	-9.22118
220	0.09875	0.08875	1.11E-06	9.89E-05	-9.2215
222	0.09975	0.08975	1.12E-06	9.89E-05	-9.22162
224	0.101	0.091	1.14E-06	9.89E-05	-9.22178
226	0.10175	0.09175	1.15E-06	9.89E-05	-9.22188
228	0.102	0.092	1.15E-06	9.89E-05	-9.22191
230	0.104	0.094	1.18E-06	9.88E-05	-9.22216
232	0.1035	0.0935	1.17E-06	9.88E-05	-9.2221

234	0.1045	0.0945	1.18E-06	9.88E-05	-9.22222
236	0.10775	0.09775	1.22E-06	9.88E-05	-9.22263
238	0.10625	0.09625	1.2E-06	9.88E-05	-9.22244
240	0.1075	0.0975	1.22E-06	9.88E-05	-9.2226
242	0.10875	0.09875	1.23E-06	9.88E-05	-9.22276
244	0.109	0.099	1.24E-06	9.88E-05	-9.22279
246	0.11175	0.10175	1.27E-06	9.87E-05	-9.22314
248	0.113	0.103	1.29E-06	9.87E-05	-9.2233
250	0.11325	0.10325	1.29E-06	9.87E-05	-9.22333
252	0.11475	0.10475	1.31E-06	9.87E-05	-9.22352
254	0.11625	0.10625	1.33E-06	9.87E-05	-9.22371
256	0.11625	0.10625	1.33E-06	9.87E-05	-9.22371
258	0.11675	0.10675	1.33E-06	9.87E-05	-9.22377
260	0.11775	0.10775	1.35E-06	9.87E-05	-9.2239
262	0.11875	0.10875	1.36E-06	9.86E-05	-9.22403
264	0.11925	0.10925	1.37E-06	9.86E-05	-9.22409
266	0.1215	0.1115	1.39E-06	9.86E-05	-9.22438
268	0.12175	0.11175	1.4E-06	9.86E-05	-9.22441
270	0.12175	0.11175	1.4E-06	9.86E-05	-9.22441
272	0.12225	0.11225	1.4E-06	9.86E-05	-9.22447
274	0.122	0.112	1.4E-06	9.86E-05	-9.22444
276	0.12375	0.11375	1.42E-06	9.86E-05	-9.22466
278	0.125	0.115	1.44E-06	9.86E-05	-9.22482
280	0.126	0.116	1.45E-06	9.86E-05	-9.22495
282	0.12825	0.11825	1.48E-06	9.85E-05	-9.22523
284	0.12775	0.11775	1.47E-06	9.85E-05	-9.22517
286	0.12875	0.11875	1.48E-06	9.85E-05	-9.2253
288	0.12925	0.11925	1.49E-06	9.85E-05	-9.22536
290	0.13075	0.12075	1.51E-06	9.85E-05	-9.22555
292	0.1315	0.1215	1.52E-06	9.85E-05	-9.22564
294	0.13225	0.12225	1.53E-06	9.85E-05	-9.22574
296	0.13275	0.12275	1.53E-06	9.85E-05	-9.2258
298	0.13225	0.12225	1.53E-06	9.85E-05	-9.22574

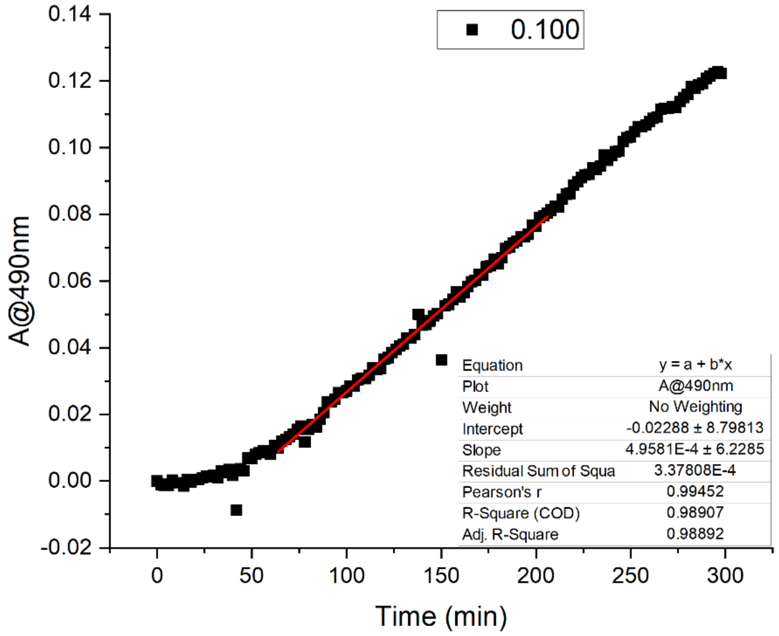

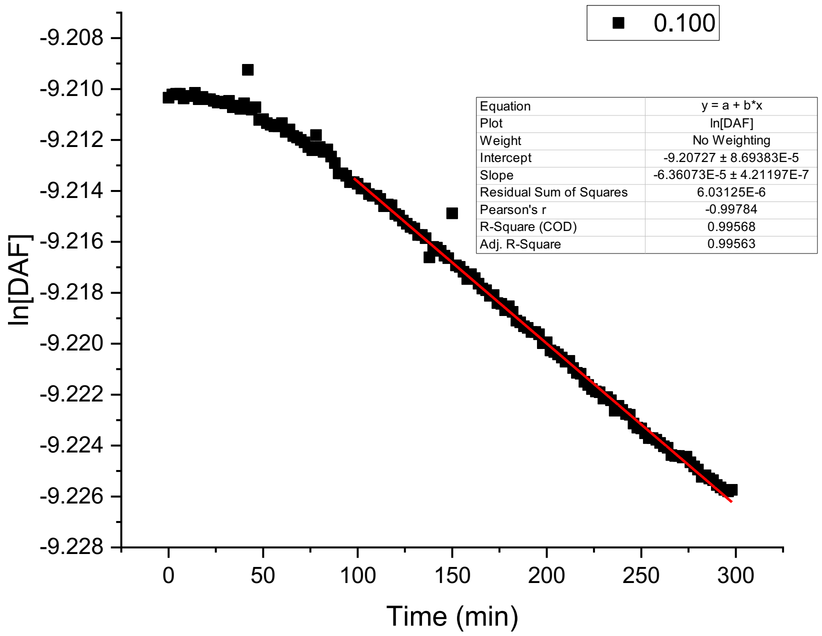

0.125 mM **DAF**

Time (min)	A@490nm minus BK	set to 0	[FLU] (M)	[DAF] (M)	ln[DAF]
0	0.041	0	0	0.000125	-8.9872
2	0.04125	0.0015	1.88E-08	0.000125	-8.98735
4	0.0395	0.00275	3.44E-08	0.000125	-8.98747
6	0.04025	0.004	5E-08	0.000125	-8.9876
8	0.04075	0.00725	9.06E-08	0.000125	-8.98792
10	0.04025	0.008	1E-07	0.000125	-8.988

12	0.04175	0.009	1.13E-07	0.000125	-8.9881
14	0.04025	0.00725	9.06E-08	0.000125	-8.98792
16	0.041	0.0055	6.88E-08	0.000125	-8.98775
18	0.0415	0.0075	9.38E-08	0.000125	-8.98795
20	0.0415	0.01225	1.53E-07	0.000125	-8.98842
22	0.0405	0.00975	1.22E-07	0.000125	-8.98817
24	0.041	0.0085	1.06E-07	0.000125	-8.98805
26	0.04225	0.00975	1.22E-07	0.000125	-8.98817
28	0.042	0.01125	1.41E-07	0.000125	-8.98832
30	0.04375	0.0115	1.44E-07	0.000125	-8.98835
32	0.04375	0.01175	1.47E-07	0.000125	-8.98837
34	0.04475	0.014	1.75E-07	0.000125	-8.9886
36	0.04475	0.0125	1.56E-07	0.000125	-8.98845
38	0.0455	0.01475	1.84E-07	0.000125	-8.98867
40	0.04625	0.01575	1.97E-07	0.000125	-8.98877
42	0.048	0.01725	2.16E-07	0.000125	-8.98892
44	0.049	0.01775	2.22E-07	0.000125	-8.98897
46	0.0485	0.0175	2.19E-07	0.000125	-8.98895
48	0.05025	0.0195	2.44E-07	0.000125	-8.98915
50	0.0505	0.0195	2.44E-07	0.000125	-8.98915
52	0.051	0.0205	2.56E-07	0.000125	-8.98925
54	0.05325	0.021	2.63E-07	0.000125	-8.9893
56	0.05375	0.022	2.75E-07	0.000125	-8.9894
58	0.05475	0.022	2.75E-07	0.000125	-8.9894
60	0.0555	0.02475	3.09E-07	0.000125	-8.98967
62	0.05625	0.0245	3.06E-07	0.000125	-8.98965
64	0.0575	0.0255	3.19E-07	0.000125	-8.98975
66	0.0575	0.025	3.13E-07	0.000125	-8.9897
68	0.058	0.026	3.25E-07	0.000125	-8.9898
70	0.059	0.02625	3.28E-07	0.000125	-8.98983
72	0.05975	0.027	3.38E-07	0.000125	-8.9899
74	0.06175	0.02875	3.59E-07	0.000125	-8.99008
76	0.06325	0.02975	3.72E-07	0.000125	-8.99018
78	0.064	0.0315	3.94E-07	0.000125	-8.99035
80	0.0645	0.031	3.88E-07	0.000125	-8.9903
82	0.066	0.03275	4.09E-07	0.000125	-8.99048
84	0.06525	0.03075	3.84E-07	0.000125	-8.99028
86	0.06875	0.03525	4.41E-07	0.000125	-8.99073
88	0.06975	0.035	4.38E-07	0.000125	-8.9907
90	0.07175	0.036	4.5E-07	0.000125	-8.9908
92	0.07125	0.036	4.5E-07	0.000125	-8.9908
94	0.0725	0.0385	4.81E-07	0.000125	-8.99105
96	0.073	0.0375	4.69E-07	0.000125	-8.99095
98	0.07525	0.0405	5.06E-07	0.000124	-8.99126
100	0.07525	0.041	5.13E-07	0.000124	-8.99131
102	0.07625	0.0415	5.19E-07	0.000124	-8.99136

104	0.078	0.04325	5.41E-07	0.000124	-8.99153
106	0.0785	0.0425	5.31E-07	0.000124	-8.99146
108	0.08	0.04325	5.41E-07	0.000124	-8.99153
110	0.0815	0.039	4.88E-07	0.000125	-8.9911
112	0.0825	0.04475	5.59E-07	0.000124	-8.99168
114	0.08475	0.0465	5.81E-07	0.000124	-8.99186
116	0.08425	0.047	5.88E-07	0.000124	-8.99191
118	0.08575	0.04825	6.03E-07	0.000124	-8.99203
120	0.087	0.04875	6.09E-07	0.000124	-8.99208
122	0.089	0.052	6.5E-07	0.000124	-8.99241
124	0.09	0.052	6.5E-07	0.000124	-8.99241
126	0.091	0.054	6.75E-07	0.000124	-8.99261
128	0.09275	0.05425	6.78E-07	0.000124	-8.99264
130	0.09525	0.05575	6.97E-07	0.000124	-8.99279
132	0.09425	0.05525	6.91E-07	0.000124	-8.99274
134	0.097	0.05825	7.28E-07	0.000124	-8.99304
136	0.096	0.0555	6.94E-07	0.000124	-8.99276
138	0.099	0.0605	7.56E-07	0.000124	-8.99327
140	0.09975	0.061	7.63E-07	0.000124	-8.99332
142	0.10125	0.0615	7.69E-07	0.000124	-8.99337
144	0.1025	0.06175	7.72E-07	0.000124	-8.99339
146	0.103	0.06225	7.78E-07	0.000124	-8.99344
148	0.10525	0.065	8.13E-07	0.000124	-8.99372
150	0.1055	0.06525	8.16E-07	0.000124	-8.99374
152	0.10725	0.06575	8.22E-07	0.000124	-8.99379
154	0.1085	0.0675	8.44E-07	0.000124	-8.99397
156	0.1095	0.06875	8.59E-07	0.000124	-8.9941
158	0.1115	0.07	8.75E-07	0.000124	-8.99422
160	0.11175	0.071	8.88E-07	0.000124	-8.99432
162	0.1135	0.071	8.88E-07	0.000124	-8.99432
164	0.115	0.07275	9.09E-07	0.000124	-8.9945
166	0.116	0.073	9.13E-07	0.000124	-8.99452
168	0.1175	0.075	9.38E-07	0.000124	-8.99473
170	0.1195	0.0765	9.56E-07	0.000124	-8.99488
172	0.11925	0.07675	9.59E-07	0.000124	-8.9949
174	0.121	0.0775	9.69E-07	0.000124	-8.99498
176	0.1225	0.07975	9.97E-07	0.000124	-8.9952
178	0.1235	0.08025	1E-06	0.000124	-8.99525
180	0.1235	0.081	1.01E-06	0.000124	-8.99533
182	0.12625	0.08275	1.03E-06	0.000124	-8.99551
184	0.12825	0.0845	1.06E-06	0.000124	-8.99568
186	0.129	0.08575	1.07E-06	0.000124	-8.99581
188	0.1305	0.087	1.09E-06	0.000124	-8.99593
190	0.13325	0.0885	1.11E-06	0.000124	-8.99609
192	0.1335	0.09025	1.13E-06	0.000124	-8.99626
194	0.135	0.09175	1.15E-06	0.000124	-8.99641

196	0.13575	0.091	1.14E-06	0.000124	-8.99634
198	0.13725	0.063	7.88E-07	0.000124	-8.99352
200	0.13875	0.094	1.18E-06	0.000124	-8.99664
202	0.1395	0.06925	8.66E-07	0.000124	-8.99415
204	0.13975	0.09525	1.19E-06	0.000124	-8.99677
206	0.14275	0.09825	1.23E-06	0.000124	-8.99707
208	0.14375	0.0985	1.23E-06	0.000124	-8.9971
210	0.14425	0.09925	1.24E-06	0.000124	-8.99717
212	0.14575	0.1	1.25E-06	0.000124	-8.99725
214	0.148	0.104	1.3E-06	0.000124	-8.99765
216	0.1485	0.09675	1.21E-06	0.000124	-8.99692
218	0.1495	0.10425	1.3E-06	0.000124	-8.99768
220	0.15175	0.092	1.15E-06	0.000124	-8.99644
222	0.15175	0.10625	1.33E-06	0.000124	-8.99788
224	0.15475	0.10875	1.36E-06	0.000124	-8.99813
226	0.15575	0.099	1.24E-06	0.000124	-8.99715
228	0.15675	0.11125	1.39E-06	0.000124	-8.99838
230	0.1575	0.1115	1.39E-06	0.000124	-8.99841
232	0.15925	0.11375	1.42E-06	0.000124	-8.99864
234	0.15925	0.11375	1.42E-06	0.000124	-8.99864
236	0.16225	0.11525	1.44E-06	0.000124	-8.99879
238	0.16275	0.1165	1.46E-06	0.000124	-8.99892
240	0.1645	0.11825	1.48E-06	0.000124	-8.99909
242	0.16625	0.1195	1.49E-06	0.000124	-8.99922
244	0.16675	0.12075	1.51E-06	0.000123	-8.99935
246	0.16925	0.12275	1.53E-06	0.000123	-8.99955
248	0.16975	0.1235	1.54E-06	0.000123	-8.99962
250	0.1715	0.12475	1.56E-06	0.000123	-8.99975
252	0.17325	0.12675	1.58E-06	0.000123	-8.99995
254	0.17425	0.12775	1.6E-06	0.000123	-9.00005
256	0.17575	0.12825	1.6E-06	0.000123	-9.0001
258	0.1765	0.129	1.61E-06	0.000123	-9.00018
260	0.17725	0.13075	1.63E-06	0.000123	-9.00036
262	0.17925	0.131	1.64E-06	0.000123	-9.00038
264	0.182	0.134	1.68E-06	0.000123	-9.00069
266	0.18175	0.1345	1.68E-06	0.000123	-9.00074
268	0.184	0.13575	1.7E-06	0.000123	-9.00086
270	0.1855	0.1375	1.72E-06	0.000123	-9.00104
272	0.18625	0.13775	1.72E-06	0.000123	-9.00107
274	0.18825	0.1395	1.74E-06	0.000123	-9.00125
276	0.18825	0.1395	1.74E-06	0.000123	-9.00125
278	0.1905	0.14125	1.77E-06	0.000123	-9.00142
280	0.1925	0.144	1.8E-06	0.000123	-9.0017
282	0.19425	0.14475	1.81E-06	0.000123	-9.00178
284	0.19425	0.145	1.81E-06	0.000123	-9.0018
286	0.195	0.14625	1.83E-06	0.000123	-9.00193

288	0.197	0.1475	1.84E-06	0.000123	-9.00206
290	0.19925	0.149	1.86E-06	0.000123	-9.00221
292	0.201	0.15275	1.91E-06	0.000123	-9.00259
294	0.20175	0.152	1.9E-06	0.000123	-9.00251
296	0.20325	0.1535	1.92E-06	0.000123	-9.00267
298	0.2045	0.154	1.93E-06	0.000123	-9.00272

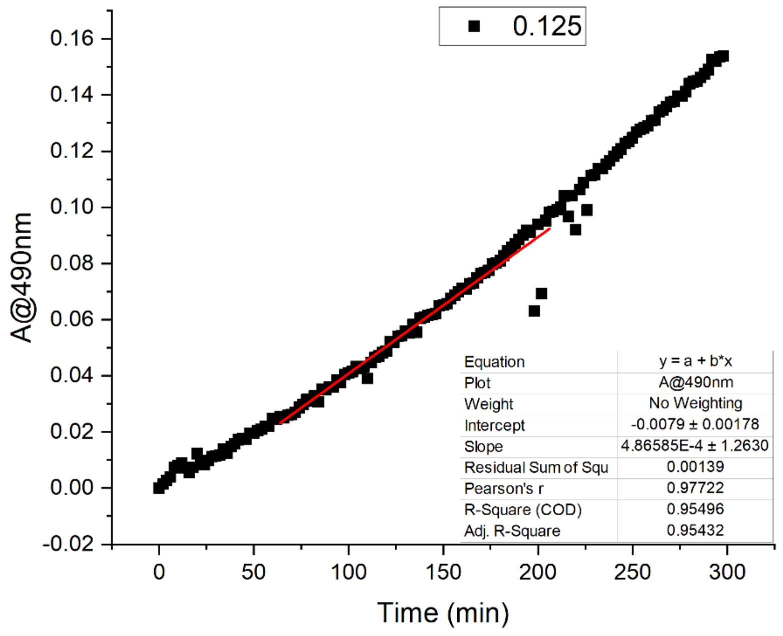

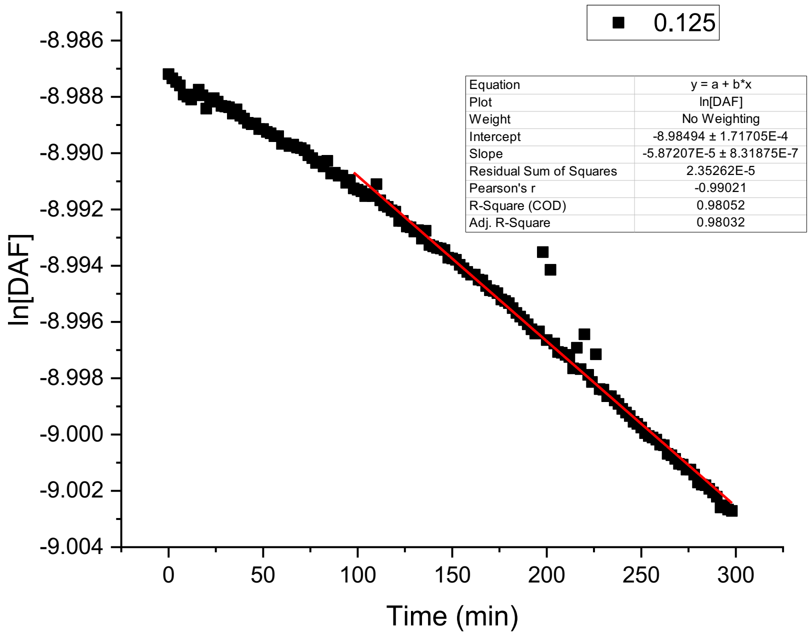

0.150 mM **DAF**

Time (min)	A@490nm minus BK	set to 0	[FLU] (M)	[DAF] (M)	ln[DAF]
0	0.04075	0	0	0.00015	-8.80488
2	0.0405	0.002	2.5E-08	0.00015	-8.80504
4	0.0405	0	0	0.00015	-8.80488
6	0.04025	0.0015	1.88E-08	0.00015	-8.805
8	0.041	0.00125	1.56E-08	0.00015	-8.80498
10	0.044	0.0045	5.63E-08	0.00015	-8.80525
12	0.041	0.00225	2.81E-08	0.00015	-8.80506
14	0.04	0.00175	2.19E-08	0.00015	-8.80502
16	0.041	0.0035	4.38E-08	0.00015	-8.80517
18	0.04075	7.50E-04	9.38E-09	0.00015	-8.80494
20	0.04225	0.001	1.25E-08	0.00015	-8.80496
22	0.0405	0.0015	1.88E-08	0.00015	-8.805
24	0.04075	0	0	0.00015	-8.80488
26	0.04275	0.0045	5.63E-08	0.00015	-8.80525
28	0.043	0.005	6.25E-08	0.00015	-8.80529
30	0.043	0.006	7.5E-08	0.00015	-8.80538
32	0.0435	0.006	7.5E-08	0.00015	-8.80538
34	0.04425	0.00675	8.44E-08	0.00015	-8.80544
36	0.04525	0.0075	9.38E-08	0.00015	-8.8055
38	0.04525	0.0075	9.38E-08	0.00015	-8.8055
40	0.0455	0.008	1E-07	0.00015	-8.80554
42	0.04775	0.01075	1.34E-07	0.00015	-8.80577
44	0.048	0.01025	1.28E-07	0.00015	-8.80573
46	0.0485	0.01	1.25E-07	0.00015	-8.80571
48	0.0505	0.01225	1.53E-07	0.00015	-8.8059
50	0.05125	0.0135	1.69E-07	0.00015	-8.806
52	0.0505	0.01425	1.78E-07	0.00015	-8.80606
54	0.0515	0.01175	1.47E-07	0.00015	-8.80585
56	0.0535	0.014	1.75E-07	0.00015	-8.80604
58	0.05375	0.01475	1.84E-07	0.00015	-8.80611
60	0.05525	0.015	1.88E-07	0.00015	-8.80613
62	0.05525	0.01625	2.03E-07	0.00015	-8.80623
64	0.057	0.01725	2.16E-07	0.00015	-8.80631
66	0.0565	0.01625	2.03E-07	0.00015	-8.80623
68	0.0585	0.01775	2.22E-07	0.00015	-8.80636
70	0.06025	0.02075	2.59E-07	0.00015	-8.80661
72	0.0615	0.02125	2.66E-07	0.00015	-8.80665
74	0.06175	0.0225	2.81E-07	0.00015	-8.80675
76	0.0625	0.022	2.75E-07	0.00015	-8.80671
78	0.06375	0.02525	3.16E-07	0.00015	-8.80698
80	0.0645	0.0245	3.06E-07	0.00015	-8.80692
82	0.067	0.027	3.38E-07	0.00015	-8.80713
84	0.064	0.0235	2.94E-07	0.00015	-8.80684
86	0.06925	0.0285	3.56E-07	0.00015	-8.80725

88	0.0685	0.02875	3.59E-07	0.00015	-8.80727
90	0.07025	0.02975	3.72E-07	0.00015	-8.80736
92	0.0715	0.0315	3.94E-07	0.00015	-8.8075
94	0.07175	0.03075	3.84E-07	0.00015	-8.80744
96	0.074	0.03525	4.41E-07	0.00015	-8.80782
98	0.07575	0.03525	4.41E-07	0.00015	-8.80782
100	0.075	0.0345	4.31E-07	0.00015	-8.80775
102	0.0765	0.03525	4.41E-07	0.00015	-8.80782
104	0.07775	0.03625	4.53E-07	0.00015	-8.8079
106	0.07875	0.0385	4.81E-07	0.00015	-8.80809
108	0.08	0.039	4.88E-07	0.00015	-8.80813
110	0.081	0.03925	4.91E-07	0.00015	-8.80815
112	0.082	0.0415	5.19E-07	0.000149	-8.80834
114	0.08425	0.043	5.38E-07	0.000149	-8.80847
116	0.08525	0.04325	5.41E-07	0.000149	-8.80849
118	0.087	0.0455	5.69E-07	0.000149	-8.80867
120	0.08925	0.048	6E-07	0.000149	-8.80888
122	0.08925	0.04725	5.91E-07	0.000149	-8.80882
124	0.09075	0.049	6.13E-07	0.000149	-8.80897
126	0.0915	0.05025	6.28E-07	0.000149	-8.80907
128	0.09225	0.0505	6.31E-07	0.000149	-8.80909
130	0.09425	0.0515	6.44E-07	0.000149	-8.80918
132	0.09625	0.054	6.75E-07	0.000149	-8.80939
134	0.097	0.054	6.75E-07	0.000149	-8.80939
136	0.0985	0.055	6.88E-07	0.000149	-8.80947
138	0.09925	0.06075	7.59E-07	0.000149	-8.80995
140	0.09975	0.057	7.13E-07	0.000149	-8.80964
142	0.1025	0.0585	7.31E-07	0.000149	-8.80976
144	0.103	0.05975	7.47E-07	0.000149	-8.80987
146	0.1035	0.0595	7.44E-07	0.000149	-8.80985
148	0.1055	0.06	7.5E-07	0.000149	-8.80989
150	0.10625	0.06125	7.66E-07	0.000149	-8.80999
152	0.1075	0.06325	7.91E-07	0.000149	-8.81016
154	0.10925	0.06375	7.97E-07	0.000149	-8.8102
156	0.1105	0.0655	8.19E-07	0.000149	-8.81035
158	0.1115	0.066	8.25E-07	0.000149	-8.81039
160	0.112	0.06575	8.22E-07	0.000149	-8.81037
162	0.11575	0.06975	8.72E-07	0.000149	-8.8107
164	0.1155	0.065	8.13E-07	0.000149	-8.81031
166	0.11625	0.0705	8.81E-07	0.000149	-8.81077
168	0.119	0.073	9.13E-07	0.000149	-8.81098
170	0.1195	0.0735	9.19E-07	0.000149	-8.81102
172	0.12	0.07325	9.16E-07	0.000149	-8.811
174	0.1225	0.076	9.5E-07	0.000149	-8.81123
176	0.124	0.07725	9.66E-07	0.000149	-8.81133
178	0.12475	0.0775	9.69E-07	0.000149	-8.81135

180	0.1245	0.0775	9.69E-07	0.000149	-8.81135
182	0.1275	0.07975	9.97E-07	0.000149	-8.81154
184	0.1295	0.082	1.03E-06	0.000149	-8.81173
186	0.1295	0.082	1.03E-06	0.000149	-8.81173
188	0.13125	0.082	1.03E-06	0.000149	-8.81173
190	0.1325	0.08425	1.05E-06	0.000149	-8.81192
192	0.13425	0.086	1.08E-06	0.000149	-8.81207
194	0.13675	0.0885	1.11E-06	0.000149	-8.81228
196	0.1375	0.08925	1.12E-06	0.000149	-8.81234
198	0.139	0.09075	1.13E-06	0.000149	-8.81247
200	0.14025	0.09125	1.14E-06	0.000149	-8.81251
202	0.142	0.09325	1.17E-06	0.000149	-8.81268
204	0.143	0.09375	1.17E-06	0.000149	-8.81272
206	0.1455	0.09575	1.2E-06	0.000149	-8.81289
208	0.14525	0.096	1.2E-06	0.000149	-8.81291
210	0.14675	0.098	1.23E-06	0.000149	-8.81308
212	0.149	0.099	1.24E-06	0.000149	-8.81316
214	0.15	0.0995	1.24E-06	0.000149	-8.8132
216	0.15175	0.10075	1.26E-06	0.000149	-8.81331
218	0.15325	0.1015	1.27E-06	0.000149	-8.81337
220	0.1555	0.1045	1.31E-06	0.000149	-8.81362
222	0.15575	0.105	1.31E-06	0.000149	-8.81366
224	0.15775	0.10725	1.34E-06	0.000149	-8.81385
226	0.15975	0.108	1.35E-06	0.000149	-8.81392
228	0.16	0.10875	1.36E-06	0.000149	-8.81398
230	0.16175	0.1105	1.38E-06	0.000149	-8.81413
232	0.1635	0.1115	1.39E-06	0.000149	-8.81421
234	0.16425	0.11175	1.4E-06	0.000149	-8.81423
236	0.16775	0.116	1.45E-06	0.000149	-8.81459
238	0.16625	0.114	1.43E-06	0.000149	-8.81442
240	0.17	0.117	1.46E-06	0.000149	-8.81467
242	0.17025	0.11725	1.47E-06	0.000149	-8.81469
244	0.17125	0.1175	1.47E-06	0.000149	-8.81472
246	0.174	0.121	1.51E-06	0.000148	-8.81501
248	0.1755	0.1225	1.53E-06	0.000148	-8.81514
250	0.176	0.12225	1.53E-06	0.000148	-8.81512
252	0.17975	0.12525	1.57E-06	0.000148	-8.81537
254	0.18	0.12625	1.58E-06	0.000148	-8.81545
256	0.18	0.12475	1.56E-06	0.000148	-8.81533
258	0.18125	0.12575	1.57E-06	0.000148	-8.81541
260	0.1825	0.127	1.59E-06	0.000148	-8.81551
262	0.1835	0.1265	1.58E-06	0.000148	-8.81547
264	0.18675	0.1305	1.63E-06	0.000148	-8.81581
266	0.18625	0.12975	1.62E-06	0.000148	-8.81575
268	0.1895	0.13325	1.67E-06	0.000148	-8.81604
270	0.19225	0.13575	1.7E-06	0.000148	-8.81625

272	0.1915	0.134	1.68E-06	0.000148	-8.8161
274	0.19375	0.13575	1.7E-06	0.000148	-8.81625
276	0.194	0.13275	1.66E-06	0.000148	-8.816
278	0.19575	0.13775	1.72E-06	0.000148	-8.81642
280	0.1985	0.13975	1.75E-06	0.000148	-8.81659
282	0.19925	0.1415	1.77E-06	0.000148	-8.81674
284	0.20125	0.1415	1.77E-06	0.000148	-8.81674
286	0.20225	0.14325	1.79E-06	0.000148	-8.81688
288	0.20325	0.143	1.79E-06	0.000148	-8.81686
290	0.205	0.1465	1.83E-06	0.000148	-8.81716
292	0.2065	0.1465	1.83E-06	0.000148	-8.81716
294	0.208	0.14475	1.81E-06	0.000148	-8.81701
296	0.209	0.148	1.85E-06	0.000148	-8.81729
298	0.212	0.15075	1.88E-06	0.000148	-8.81752

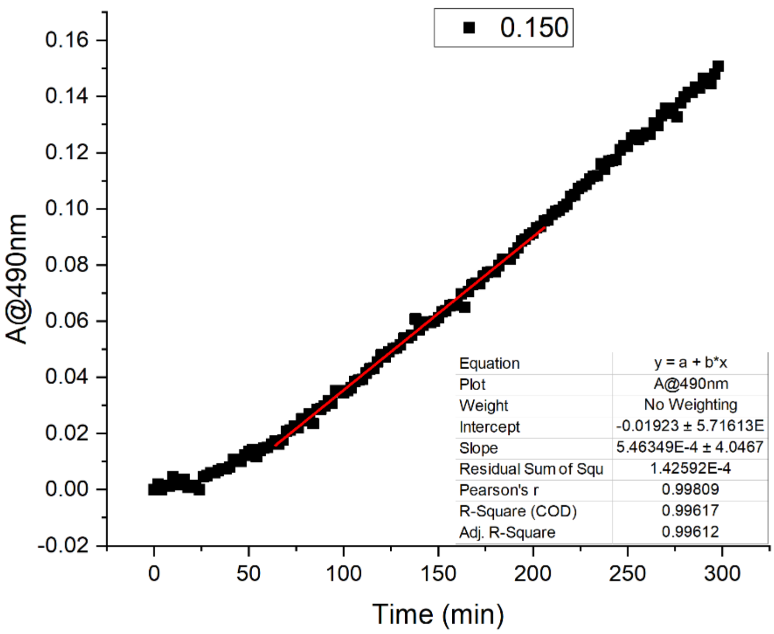

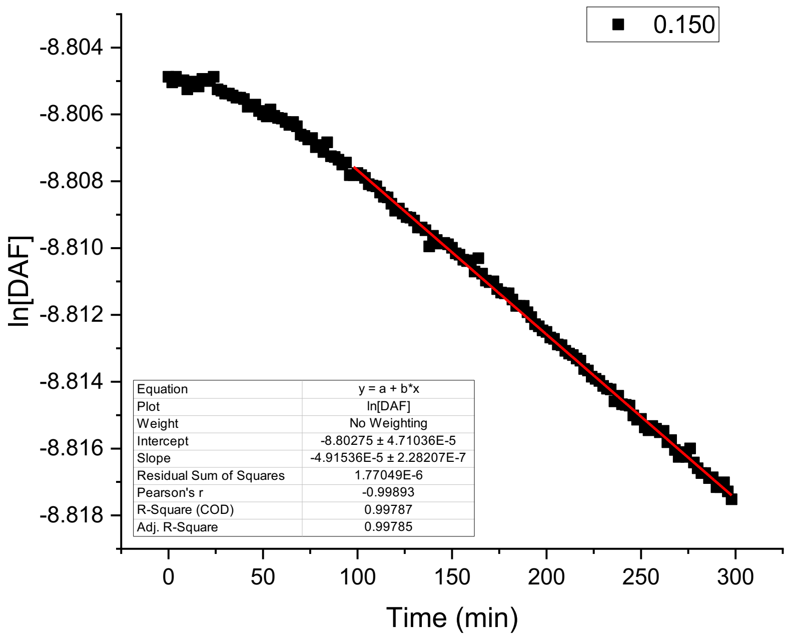

0.175 mM **DAF**

Time (min)	A@490nm minus BK	set to 0	[FLU] (M)	[DAF] (M)	ln[DAF]
0	0.05325	0	0	0.000175	-8.65072
2	0.04725	-0.002	-2.5E-08	0.000175	-8.65058
4	0.047	-0.00275	-3.4E-08	0.000175	-8.65053
6	0.0485	-0.00175	-2.2E-08	0.000175	-8.6506
8	0.0475	-0.00125	-1.6E-08	0.000175	-8.65064
10	0.049	-0.001	-1.3E-08	0.000175	-8.65065
12	0.0475	-0.0015	-1.9E-08	0.000175	-8.65062
14	0.0475	-0.0035	-4.4E-08	0.000175	-8.65047
16	0.04725	-0.00275	-3.4E-08	0.000175	-8.65053
18	0.047	-0.00275	-3.4E-08	0.000175	-8.65053
20	0.0475	-0.00375	-4.7E-08	0.000175	-8.65046
22	0.04625	-0.00325	-4.1E-08	0.000175	-8.65049
24	0.0475	-0.0025	-3.1E-08	0.000175	-8.65055
26	0.048	-0.00475	-5.9E-08	0.000175	-8.65039
28	0.049	-0.00325	-4.1E-08	0.000175	-8.65049
30	0.05	-5.00E-04	-6.3E-09	0.000175	-8.65069
32	0.04875	-0.003	-3.8E-08	0.000175	-8.65051
34	0.05	-0.00125	-1.6E-08	0.000175	-8.65064
36	0.05075	-5.00E-04	-6.3E-09	0.000175	-8.65069
38	0.0515	2.50E-04	3.13E-09	0.000175	-8.65074
40	0.0515	2.50E-04	3.13E-09	0.000175	-8.65074
42	0.05275	0.00125	1.56E-08	0.000175	-8.65081
44	0.05275	0.001	1.25E-08	0.000175	-8.6508
46	0.053	0.00125	1.56E-08	0.000175	-8.65081
48	0.05525	0.0045	5.63E-08	0.000175	-8.65105

50	0.05625	0.00475	5.94E-08	0.000175	-8.65106
52	0.05675	0.00625	7.81E-08	0.000175	-8.65117
54	0.05725	0.005	6.25E-08	0.000175	-8.65108
56	0.05975	0.00775	9.69E-08	0.000175	-8.65128
58	0.059	0.007	8.75E-08	0.000175	-8.65122
60	0.06	0.008	1E-07	0.000175	-8.6513
62	0.06225	0.00975	1.22E-07	0.000175	-8.65142
64	0.06325	0.01025	1.28E-07	0.000175	-8.65146
66	0.064	0.012	1.5E-07	0.000175	-8.65158
68	0.06425	0.01225	1.53E-07	0.000175	-8.6516
70	0.065	0.013	1.63E-07	0.000175	-8.65165
72	0.06675	0.01475	1.84E-07	0.000175	-8.65178
74	0.06825	0.0165	2.06E-07	0.000175	-8.6519
76	0.07025	0.019	2.38E-07	0.000175	-8.65208
78	0.07025	0.0165	2.06E-07	0.000175	-8.6519
80	0.072	0.01825	2.28E-07	0.000175	-8.65203
82	0.07225	0.01975	2.47E-07	0.000175	-8.65214
84	0.07075	0.01675	2.09E-07	0.000175	-8.65192
86	0.07575	0.022	2.75E-07	0.000175	-8.6523
88	0.07625	0.023	2.88E-07	0.000175	-8.65237
90	0.0765	0.02425	3.03E-07	0.000175	-8.65246
92	0.0775	0.02375	2.97E-07	0.000175	-8.65242
94	0.0785	0.025	3.13E-07	0.000175	-8.65251
96	0.08025	0.02725	3.41E-07	0.000175	-8.65267
98	0.08175	0.028	3.5E-07	0.000175	-8.65273
100	0.08225	0.02775	3.47E-07	0.000175	-8.65271
102	0.082	0.027	3.38E-07	0.000175	-8.65266
104	0.0845	0.02975	3.72E-07	0.000175	-8.65285
106	0.08475	0.0305	3.81E-07	0.000175	-8.65291
108	0.08775	0.03325	4.16E-07	0.000175	-8.6531
110	0.0875	0.03225	4.03E-07	0.000175	-8.65303
112	0.0885	0.0345	4.31E-07	0.000175	-8.65319
114	0.09025	0.036	4.5E-07	0.000175	-8.6533
116	0.09225	0.0365	4.56E-07	0.000175	-8.65334
118	0.09275	0.03725	4.66E-07	0.000175	-8.65339
120	0.0945	0.039	4.88E-07	0.000175	-8.65351
122	0.09675	0.04075	5.09E-07	0.000174	-8.65364
124	0.0975	0.0425	5.31E-07	0.000174	-8.65376
126	0.09975	0.04375	5.47E-07	0.000174	-8.65385
128	0.1	0.04475	5.59E-07	0.000174	-8.65393
130	0.10225	0.04525	5.66E-07	0.000174	-8.65396
132	0.104	0.04775	5.97E-07	0.000174	-8.65414
134	0.10375	0.0475	5.94E-07	0.000174	-8.65412
136	0.10525	0.04925	6.16E-07	0.000174	-8.65425
138	0.107	0.056	7E-07	0.000174	-8.65473
140	0.10725	0.05025	6.28E-07	0.000174	-8.65432

142	0.1105	0.05425	6.78E-07	0.000174	-8.65461
144	0.11	0.05375	6.72E-07	0.000174	-8.65457
146	0.11075	0.05325	6.66E-07	0.000174	-8.65454
148	0.11375	0.0555	6.94E-07	0.000174	-8.6547
150	0.114	0.0555	6.94E-07	0.000174	-8.6547
152	0.11475	0.05725	7.16E-07	0.000174	-8.65482
154	0.118	0.05975	7.47E-07	0.000174	-8.655
156	0.11825	0.05975	7.47E-07	0.000174	-8.655
158	0.1195	0.06025	7.53E-07	0.000174	-8.65504
160	0.12125	0.063	7.88E-07	0.000174	-8.65523
162	0.12225	0.0635	7.94E-07	0.000174	-8.65527
164	0.12375	0.065	8.13E-07	0.000174	-8.65538
166	0.12375	0.06525	8.16E-07	0.000174	-8.6554
168	0.1265	0.06775	8.47E-07	0.000174	-8.65558
170	0.12725	0.0675	8.44E-07	0.000174	-8.65556
172	0.128	0.069	8.63E-07	0.000174	-8.65567
174	0.1305	0.07325	9.16E-07	0.000174	-8.65597
176	0.1325	0.074	9.25E-07	0.000174	-8.65602
178	0.13275	0.074	9.25E-07	0.000174	-8.65602
180	0.13375	0.0745	9.31E-07	0.000174	-8.65606
182	0.1355	0.07625	9.53E-07	0.000174	-8.65619
184	0.1365	0.07675	9.59E-07	0.000174	-8.65622
186	0.13825	0.078	9.75E-07	0.000174	-8.65631
188	0.13925	0.079	9.88E-07	0.000174	-8.65638
190	0.14	0.07925	9.91E-07	0.000174	-8.6564
192	0.1425	0.08225	1.03E-06	0.000174	-8.65662
194	0.14375	0.08325	1.04E-06	0.000174	-8.65669
196	0.14475	0.08475	1.06E-06	0.000174	-8.6568
198	0.14675	0.08725	1.09E-06	0.000174	-8.65698
200	0.1475	0.08725	1.09E-06	0.000174	-8.65698
202	0.149	0.08875	1.11E-06	0.000174	-8.65708
204	0.15075	0.09025	1.13E-06	0.000174	-8.65719
206	0.15275	0.0915	1.14E-06	0.000174	-8.65728
208	0.15275	0.0915	1.14E-06	0.000174	-8.65728
210	0.15375	0.09275	1.16E-06	0.000174	-8.65737
212	0.1555	0.09375	1.17E-06	0.000174	-8.65744
214	0.158	0.0965	1.21E-06	0.000174	-8.65764
216	0.158	0.09625	1.2E-06	0.000174	-8.65762
218	0.15975	0.0965	1.21E-06	0.000174	-8.65764
220	0.162	0.09925	1.24E-06	0.000174	-8.65784
222	0.16325	0.101	1.26E-06	0.000174	-8.65797
224	0.16525	0.1025	1.28E-06	0.000174	-8.65807
226	0.167	0.10475	1.31E-06	0.000174	-8.65823
228	0.167	0.10425	1.3E-06	0.000174	-8.6582
230	0.16925	0.10675	1.33E-06	0.000174	-8.65838
232	0.17	0.1065	1.33E-06	0.000174	-8.65836

234	0.173	0.1095	1.37E-06	0.000174	-8.65858
236	0.17275	0.111	1.39E-06	0.000174	-8.65868
238	0.17375	0.1105	1.38E-06	0.000174	-8.65865
240	0.1755	0.11175	1.4E-06	0.000174	-8.65874
242	0.178	0.114	1.43E-06	0.000174	-8.6589
244	0.17875	0.11375	1.42E-06	0.000174	-8.65888
246	0.18025	0.117	1.46E-06	0.000174	-8.65912
248	0.18375	0.1205	1.51E-06	0.000173	-8.65937
250	0.18375	0.12025	1.5E-06	0.000173	-8.65935
252	0.18625	0.1225	1.53E-06	0.000173	-8.65951
254	0.18725	0.1235	1.54E-06	0.000173	-8.65959
256	0.1885	0.12325	1.54E-06	0.000173	-8.65957
258	0.1895	0.12475	1.56E-06	0.000173	-8.65968
260	0.19125	0.12575	1.57E-06	0.000173	-8.65975
262	0.1925	0.12725	1.59E-06	0.000173	-8.65986
264	0.1955	0.1295	1.62E-06	0.000173	-8.66002
266	0.1945	0.13	1.63E-06	0.000173	-8.66005
268	0.19675	0.131	1.64E-06	0.000173	-8.66013
270	0.2005	0.1355	1.69E-06	0.000173	-8.66045
272	0.20125	0.13425	1.68E-06	0.000173	-8.66036
274	0.2025	0.1365	1.71E-06	0.000173	-8.66052
276	0.20225	0.13575	1.7E-06	0.000173	-8.66047
278	0.205	0.13775	1.72E-06	0.000173	-8.66061
280	0.20675	0.14025	1.75E-06	0.000173	-8.66079
282	0.21025	0.14325	1.79E-06	0.000173	-8.66101
284	0.21	0.14275	1.78E-06	0.000173	-8.66097
286	0.21125	0.14375	1.8E-06	0.000173	-8.66105
288	0.21325	0.14525	1.82E-06	0.000173	-8.66115
290	0.2145	0.1475	1.84E-06	0.000173	-8.66132
292	0.21625	0.149	1.86E-06	0.000173	-8.66142
294	0.218	0.15025	1.88E-06	0.000173	-8.66151
296	0.21775	0.1505	1.88E-06	0.000173	-8.66153
298	0.22075	0.15175	1.9E-06	0.000173	-8.66162

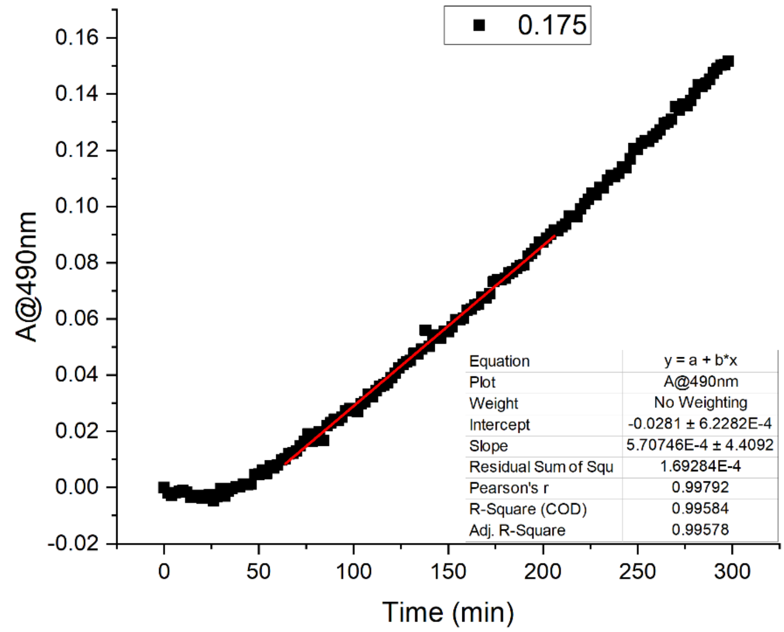

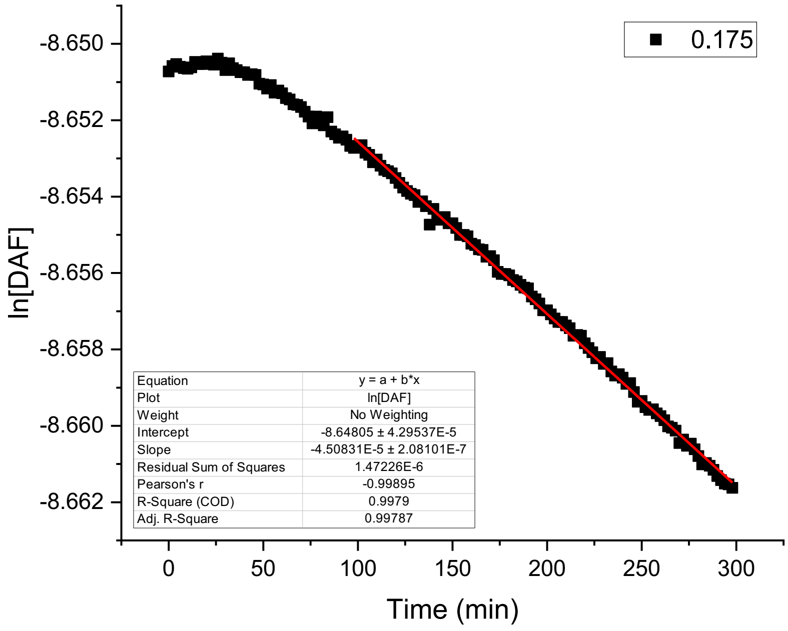

0.200 mM **DAF**

Time (min)	A@490nm minus BK	set to 0	[FLU] (M)	[DAF] (M)	ln[DAF]
0	0.04825	0	0	0.0002	-8.51719
2	0.0475	5.00E-04	6.25E-09	0.0002	-8.51722
4	0.04775	-0.00125	-1.6E-08	0.0002	-8.51712
6	0.04875	0.0025	3.13E-08	0.0002	-8.51735
8	0.05025	0.005	6.25E-08	0.0002	-8.51751
10	0.05075	0.005	6.25E-08	0.0002	-8.51751

12	0.05125	0.00475	5.94E-08	0.0002	-8.51749
14	0.04925	0.00375	4.69E-08	0.0002	-8.51743
16	0.05075	0.00475	5.94E-08	0.0002	-8.51749
18	0.049	0.00325	4.06E-08	0.0002	-8.5174
20	0.05	0.00375	4.69E-08	0.0002	-8.51743
22	0.05	0.00425	5.31E-08	0.0002	-8.51746
24	0.05025	0.0045	5.63E-08	0.0002	-8.51747
26	0.0515	0.006	7.5E-08	0.0002	-8.51757
28	0.053	0.00525	6.56E-08	0.0002	-8.51752
30	0.05375	0.00875	1.09E-07	0.0002	-8.51774
32	0.05275	0.007	8.75E-08	0.0002	-8.51763
34	0.05425	0.0085	1.06E-07	0.0002	-8.51772
36	0.0545	0.0075	9.38E-08	0.0002	-8.51766
38	0.0545	0.0075	9.38E-08	0.0002	-8.51766
40	0.05525	0.0085	1.06E-07	0.0002	-8.51772
42	0.056	0.00875	1.09E-07	0.0002	-8.51774
44	0.057	0.00725	9.06E-08	0.0002	-8.51765
46	0.0575	0.01025	1.28E-07	0.0002	-8.51783
48	0.058	0.0105	1.31E-07	0.0002	-8.51785
50	0.06125	0.01175	1.47E-07	0.0002	-8.51793
52	0.0605	0.0145	1.81E-07	0.0002	-8.5181
54	0.062	0.0145	1.81E-07	0.0002	-8.5181
56	0.063	0.015	1.88E-07	0.0002	-8.51813
58	0.063	0.01425	1.78E-07	0.0002	-8.51808
60	0.0645	0.01575	1.97E-07	0.0002	-8.51818
62	0.06625	0.017	2.13E-07	0.0002	-8.51826
64	0.06725	0.01825	2.28E-07	0.0002	-8.51833
66	0.06775	0.01975	2.47E-07	0.0002	-8.51843
68	0.0685	0.019	2.38E-07	0.0002	-8.51838
70	0.06925	0.02	2.5E-07	0.0002	-8.51844
72	0.07125	0.02225	2.78E-07	0.0002	-8.51858
74	0.072	0.02325	2.91E-07	0.0002	-8.51865
76	0.073	0.024	3E-07	0.0002	-8.51869
78	0.07425	0.0245	3.06E-07	0.0002	-8.51873
80	0.075	0.02575	3.22E-07	0.0002	-8.5188
82	0.07625	0.028	3.5E-07	0.0002	-8.51894
84	0.0755	0.025	3.13E-07	0.0002	-8.51876
86	0.07825	0.0255	3.19E-07	0.0002	-8.51879
88	0.07975	0.03	3.75E-07	0.0002	-8.51907
90	0.0815	0.032	4E-07	0.0002	-8.5192
92	0.0815	0.0315	3.94E-07	0.0002	-8.51916
94	0.083	0.033	4.13E-07	0.0002	-8.51926
96	0.084	0.0335	4.19E-07	0.0002	-8.51929
98	0.086	0.03475	4.34E-07	0.0002	-8.51937
100	0.0855	0.035	4.38E-07	0.0002	-8.51938
102	0.0865	0.03575	4.47E-07	0.0002	-8.51943

104	0.09	0.03775	4.72E-07	0.0002	-8.51956
106	0.089	0.039	4.88E-07	0.0002	-8.51963
108	0.0915	0.0405	5.06E-07	0.000199	-8.51973
110	0.09175	0.04025	5.03E-07	0.000199	-8.51971
112	0.0925	0.042	5.25E-07	0.000199	-8.51982
114	0.09525	0.04375	5.47E-07	0.000199	-8.51993
116	0.09525	0.0445	5.56E-07	0.000199	-8.51998
118	0.09675	0.046	5.75E-07	0.000199	-8.52007
120	0.0985	0.04725	5.91E-07	0.000199	-8.52015
122	0.10025	0.047	5.88E-07	0.000199	-8.52014
124	0.10075	0.049	6.13E-07	0.000199	-8.52026
126	0.103	0.05025	6.28E-07	0.000199	-8.52034
128	0.1035	0.05125	6.41E-07	0.000199	-8.5204
130	0.10525	0.052	6.5E-07	0.000199	-8.52045
132	0.10575	0.05325	6.66E-07	0.000199	-8.52053
134	0.10775	0.055	6.88E-07	0.000199	-8.52064
136	0.10875	0.05625	7.03E-07	0.000199	-8.52072
138	0.11025	0.061	7.63E-07	0.000199	-8.52101
140	0.1105	0.0575	7.19E-07	0.000199	-8.52079
142	0.11225	0.0585	7.31E-07	0.000199	-8.52086
144	0.11425	0.06075	7.59E-07	0.000199	-8.521
146	0.11525	0.06075	7.59E-07	0.000199	-8.521
148	0.11675	0.062	7.75E-07	0.000199	-8.52108
150	0.11875	0.064	8E-07	0.000199	-8.5212
152	0.11875	0.06425	8.03E-07	0.000199	-8.52122
154	0.12025	0.06525	8.16E-07	0.000199	-8.52128
156	0.12175	0.06625	8.28E-07	0.000199	-8.52134
158	0.1235	0.0675	8.44E-07	0.000199	-8.52142
160	0.124	0.0685	8.56E-07	0.000199	-8.52148
162	0.1255	0.07	8.75E-07	0.000199	-8.52158
164	0.12675	0.07075	8.84E-07	0.000199	-8.52162
166	0.1285	0.07325	9.16E-07	0.000199	-8.52178
168	0.13	0.074	9.25E-07	0.000199	-8.52183
170	0.13225	0.076	9.5E-07	0.000199	-8.52195
172	0.13225	0.0765	9.56E-07	0.000199	-8.52199
174	0.13425	0.0775	9.69E-07	0.000199	-8.52205
176	0.13525	0.078	9.75E-07	0.000199	-8.52208
178	0.13775	0.0815	1.02E-06	0.000199	-8.5223
180	0.13675	0.0795	9.94E-07	0.000199	-8.52217
182	0.1395	0.0815	1.02E-06	0.000199	-8.5223
184	0.14125	0.083	1.04E-06	0.000199	-8.52239
186	0.143	0.08475	1.06E-06	0.000199	-8.5225
188	0.14325	0.084	1.05E-06	0.000199	-8.52246
190	0.14525	0.087	1.09E-06	0.000199	-8.52265
192	0.14725	0.089	1.11E-06	0.000199	-8.52277
194	0.148	0.08875	1.11E-06	0.000199	-8.52276

196	0.1495	0.0905	1.13E-06	0.000199	-8.52287
198	0.151	0.092	1.15E-06	0.000199	-8.52296
200	0.15275	0.0935	1.17E-06	0.000199	-8.52305
202	0.15375	0.09425	1.18E-06	0.000199	-8.5231
204	0.156	0.0965	1.21E-06	0.000199	-8.52324
206	0.15775	0.0975	1.22E-06	0.000199	-8.52331
208	0.15825	0.098	1.23E-06	0.000199	-8.52334
210	0.15975	0.09975	1.25E-06	0.000199	-8.52345
212	0.16125	0.1005	1.26E-06	0.000199	-8.52349
214	0.16325	0.103	1.29E-06	0.000199	-8.52365
216	0.16425	0.10225	1.28E-06	0.000199	-8.5236
218	0.16625	0.105	1.31E-06	0.000199	-8.52378
220	0.1675	0.1055	1.32E-06	0.000199	-8.52381
222	0.16825	0.10625	1.33E-06	0.000199	-8.52386
224	0.1695	0.1085	1.36E-06	0.000199	-8.524
226	0.17275	0.11125	1.39E-06	0.000199	-8.52417
228	0.1735	0.111	1.39E-06	0.000199	-8.52415
230	0.174	0.11225	1.4E-06	0.000199	-8.52423
232	0.17625	0.113	1.41E-06	0.000199	-8.52428
234	0.176	0.112	1.4E-06	0.000199	-8.52422
236	0.179	0.1165	1.46E-06	0.000199	-8.5245
238	0.18	0.11625	1.45E-06	0.000199	-8.52449
240	0.18075	0.11625	1.45E-06	0.000199	-8.52449
242	0.1825	0.118	1.48E-06	0.000199	-8.5246
244	0.1845	0.12025	1.5E-06	0.000198	-8.52474
246	0.18675	0.12225	1.53E-06	0.000198	-8.52486
248	0.188	0.123	1.54E-06	0.000198	-8.52491
250	0.1885	0.12225	1.53E-06	0.000198	-8.52486
252	0.1905	0.125	1.56E-06	0.000198	-8.52504
254	0.19225	0.1265	1.58E-06	0.000198	-8.52513
256	0.1945	0.12725	1.59E-06	0.000198	-8.52518
258	0.195	0.12775	1.6E-06	0.000198	-8.52521
260	0.19575	0.1285	1.61E-06	0.000198	-8.52526
262	0.19775	0.1305	1.63E-06	0.000198	-8.52538
264	0.2005	0.133	1.66E-06	0.000198	-8.52554
266	0.2005	0.13275	1.66E-06	0.000198	-8.52552
268	0.203	0.13425	1.68E-06	0.000198	-8.52562
270	0.2045	0.1365	1.71E-06	0.000198	-8.52576
272	0.206	0.13625	1.7E-06	0.000198	-8.52575
274	0.2075	0.13825	1.73E-06	0.000198	-8.52587
276	0.20825	0.139	1.74E-06	0.000198	-8.52592
278	0.2105	0.14	1.75E-06	0.000198	-8.52598
280	0.21325	0.14125	1.77E-06	0.000198	-8.52606
282	0.21475	0.14525	1.82E-06	0.000198	-8.52631
284	0.215	0.14425	1.8E-06	0.000198	-8.52625
286	0.217	0.146	1.83E-06	0.000198	-8.52636

288	0.2185	0.14725	1.84E-06	0.000198	-8.52644
290	0.22	0.14925	1.87E-06	0.000198	-8.52657
292	0.2205	0.1495	1.87E-06	0.000198	-8.52658
294	0.222	0.1505	1.88E-06	0.000198	-8.52664
296	0.224	0.15225	1.9E-06	0.000198	-8.52675
298	0.2265	0.15375	1.92E-06	0.000198	-8.52685

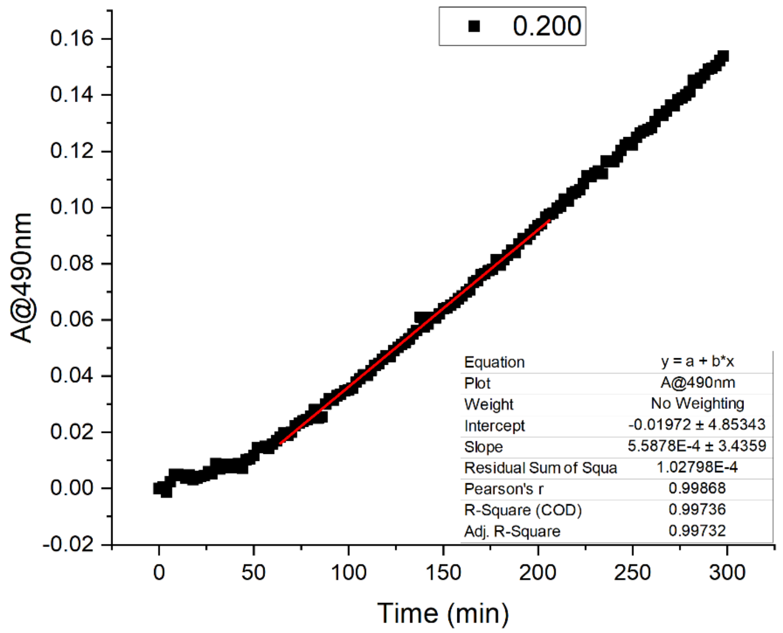

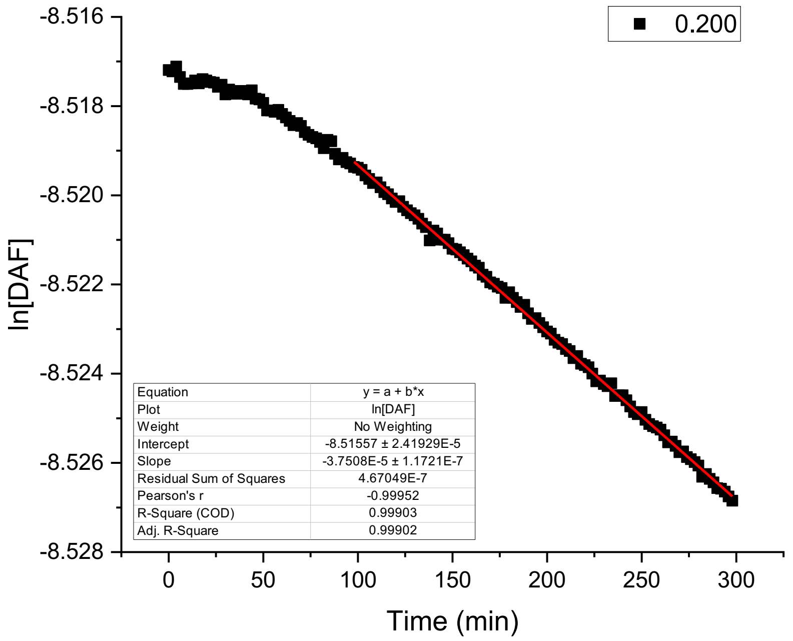

0.225 mM **DAF**

Time (min)	A@490nm minus BK	set to 0	[FLU] (M)	[DAF] (M)	ln[DAF]
0	0.05075	0	0	0.000225	-8.39941
2	0.05075	0.008	1E-07	0.000225	-8.39985
4	0.05175	0.01525	1.91E-07	0.000225	-8.40026
6	0.052	0.015	1.88E-07	0.000225	-8.40024
8	0.0535	0.017	2.13E-07	0.000225	-8.40036
10	0.052	0.0135	1.69E-07	0.000225	-8.40016
12	0.054	0.01825	2.28E-07	0.000225	-8.40042
14	0.0545	0.01925	2.41E-07	0.000225	-8.40048
16	0.055	0.0205	2.56E-07	0.000225	-8.40055
18	0.05525	0.0195	2.44E-07	0.000225	-8.40049
20	0.05625	0.02025	2.53E-07	0.000225	-8.40054
22	0.05625	0.0195	2.44E-07	0.000225	-8.40049
24	0.0565	0.0215	2.69E-07	0.000225	-8.40061
26	0.057	0.0215	2.69E-07	0.000225	-8.40061
28	0.05725	0.02075	2.59E-07	0.000225	-8.40056
30	0.058	0.02075	2.59E-07	0.000225	-8.40056
32	0.05825	0.02275	2.84E-07	0.000225	-8.40067
34	0.059	0.0235	2.94E-07	0.000225	-8.40072
36	0.06025	0.02375	2.97E-07	0.000225	-8.40073
38	0.06125	0.02275	2.84E-07	0.000225	-8.40067
40	0.06225	0.026	3.25E-07	0.000225	-8.40086
42	0.06275	0.026	3.25E-07	0.000225	-8.40086
44	0.0635	0.02875	3.59E-07	0.000225	-8.40101
46	0.064	0.02675	3.34E-07	0.000225	-8.4009
48	0.06575	0.03225	4.03E-07	0.000225	-8.4012
50	0.06675	0.03225	4.03E-07	0.000225	-8.4012
52	0.067	0.033	4.13E-07	0.000225	-8.40125
54	0.0675	0.0335	4.19E-07	0.000225	-8.40127
56	0.0695	0.03625	4.53E-07	0.000225	-8.40143
58	0.06975	0.0335	4.19E-07	0.000225	-8.40127
60	0.071	0.0365	4.56E-07	0.000225	-8.40144
62	0.0725	0.038	4.75E-07	0.000225	-8.40152
64	0.07325	0.0385	4.81E-07	0.000225	-8.40155
66	0.07425	0.03825	4.78E-07	0.000225	-8.40154
68	0.07475	0.03925	4.91E-07	0.000225	-8.40159
70	0.07675	0.043	5.38E-07	0.000224	-8.4018
72	0.07775	0.04275	5.34E-07	0.000224	-8.40179
74	0.07875	0.045	5.63E-07	0.000224	-8.40191
76	0.079	0.044	5.5E-07	0.000224	-8.40186
78	0.08025	0.0455	5.69E-07	0.000224	-8.40194
80	0.082	0.04725	5.91E-07	0.000224	-8.40204
82	0.08325	0.04925	6.16E-07	0.000224	-8.40215
84	0.08325	0.0485	6.06E-07	0.000224	-8.40211
86	0.08475	0.0485	6.06E-07	0.000224	-8.40211

88	0.08625	0.05175	6.47E-07	0.000224	-8.40229
90	0.0875	0.0535	6.69E-07	0.000224	-8.40239
92	0.089	0.05525	6.91E-07	0.000224	-8.40248
94	0.09	0.0545	6.81E-07	0.000224	-8.40244
96	0.0915	0.05675	7.09E-07	0.000224	-8.40257
98	0.0935	0.05825	7.28E-07	0.000224	-8.40265
100	0.093	0.0575	7.19E-07	0.000224	-8.40261
102	0.09475	0.05825	7.28E-07	0.000224	-8.40265
104	0.096	0.0595	7.44E-07	0.000224	-8.40272
106	0.097	0.06225	7.78E-07	0.000224	-8.40287
108	0.0995	0.0635	7.94E-07	0.000224	-8.40294
110	0.1005	0.06425	8.03E-07	0.000224	-8.40299
112	0.1015	0.0655	8.19E-07	0.000224	-8.40306
114	0.10225	0.06725	8.41E-07	0.000224	-8.40315
116	0.104	0.0675	8.44E-07	0.000224	-8.40317
118	0.10525	0.06925	8.66E-07	0.000224	-8.40326
120	0.106	0.07	8.75E-07	0.000224	-8.40331
122	0.10825	0.0715	8.94E-07	0.000224	-8.40339
124	0.11025	0.07425	9.28E-07	0.000224	-8.40354
126	0.1125	0.0755	9.44E-07	0.000224	-8.40361
128	0.1125	0.076	9.5E-07	0.000224	-8.40364
130	0.11525	0.0775	9.69E-07	0.000224	-8.40373
132	0.11475	0.077	9.63E-07	0.000224	-8.4037
134	0.1155	0.078	9.75E-07	0.000224	-8.40375
136	0.118	0.0785	9.81E-07	0.000224	-8.40378
138	0.11975	0.0855	1.07E-06	0.000224	-8.40417
140	0.12075	0.083	1.04E-06	0.000224	-8.40403
142	0.1225	0.08425	1.05E-06	0.000224	-8.4041
144	0.124	0.0855	1.07E-06	0.000224	-8.40417
146	0.124	0.0855	1.07E-06	0.000224	-8.40417
148	0.12625	0.08675	1.08E-06	0.000224	-8.40424
150	0.12725	0.08825	1.1E-06	0.000224	-8.40432
152	0.12925	0.09125	1.14E-06	0.000224	-8.40449
154	0.13025	0.09175	1.15E-06	0.000224	-8.40452
156	0.1315	0.0925	1.16E-06	0.000224	-8.40456
158	0.13425	0.095	1.19E-06	0.000224	-8.4047
160	0.13425	0.09475	1.18E-06	0.000224	-8.40469
162	0.1355	0.09625	1.2E-06	0.000224	-8.40477
164	0.1375	0.0985	1.23E-06	0.000224	-8.4049
166	0.139	0.10025	1.25E-06	0.000224	-8.405
168	0.14025	0.1015	1.27E-06	0.000224	-8.40507
170	0.142	0.10225	1.28E-06	0.000224	-8.40511
172	0.14275	0.10375	1.3E-06	0.000224	-8.40519
174	0.1445	0.1045	1.31E-06	0.000224	-8.40523
176	0.1455	0.105	1.31E-06	0.000224	-8.40526
178	0.148	0.1095	1.37E-06	0.000224	-8.40551

180	0.14725	0.10925	1.37E-06	0.000224	-8.4055
182	0.14925	0.1105	1.38E-06	0.000224	-8.40557
184	0.15225	0.11275	1.41E-06	0.000224	-8.40569
186	0.15275	0.11225	1.4E-06	0.000224	-8.40567
188	0.156	0.11625	1.45E-06	0.000224	-8.40589
190	0.156	0.1155	1.44E-06	0.000224	-8.40585
192	0.15775	0.11775	1.47E-06	0.000224	-8.40597
194	0.15925	0.11875	1.48E-06	0.000224	-8.40603
196	0.161	0.12075	1.51E-06	0.000223	-8.40614
198	0.163	0.12375	1.55E-06	0.000223	-8.40631
200	0.1645	0.12525	1.57E-06	0.000223	-8.40639
202	0.1655	0.1255	1.57E-06	0.000223	-8.40641
204	0.1665	0.127	1.59E-06	0.000223	-8.40649
206	0.16925	0.12875	1.61E-06	0.000223	-8.40659
208	0.16925	0.12825	1.6E-06	0.000223	-8.40656
210	0.17125	0.12975	1.62E-06	0.000223	-8.40664
212	0.1725	0.131	1.64E-06	0.000223	-8.40671
214	0.17475	0.1335	1.67E-06	0.000223	-8.40685
216	0.17625	0.13525	1.69E-06	0.000223	-8.40695
218	0.17775	0.13525	1.69E-06	0.000223	-8.40695
220	0.18	0.138	1.73E-06	0.000223	-8.40711
222	0.18075	0.14	1.75E-06	0.000223	-8.40722
224	0.18225	0.14175	1.77E-06	0.000223	-8.40732
226	0.18425	0.14275	1.78E-06	0.000223	-8.40737
228	0.18675	0.1455	1.82E-06	0.000223	-8.40753
230	0.18725	0.145	1.81E-06	0.000223	-8.4075
232	0.1885	0.1465	1.83E-06	0.000223	-8.40758
234	0.18975	0.14725	1.84E-06	0.000223	-8.40762
236	0.192	0.1505	1.88E-06	0.000223	-8.40781
238	0.19175	0.14975	1.87E-06	0.000223	-8.40776
240	0.195	0.15225	1.9E-06	0.000223	-8.4079
242	0.1955	0.15275	1.91E-06	0.000223	-8.40793
244	0.197	0.15425	1.93E-06	0.000223	-8.40802
246	0.20025	0.15775	1.97E-06	0.000223	-8.40821
248	0.2005	0.1585	1.98E-06	0.000223	-8.40825
250	0.203	0.16075	2.01E-06	0.000223	-8.40838
252	0.20425	0.16175	2.02E-06	0.000223	-8.40844
254	0.206	0.1635	2.04E-06	0.000223	-8.40853
256	0.2075	0.16375	2.05E-06	0.000223	-8.40855
258	0.2085	0.16425	2.05E-06	0.000223	-8.40858
260	0.21025	0.167	2.09E-06	0.000223	-8.40873
262	0.2125	0.168	2.1E-06	0.000223	-8.40879
264	0.21375	0.17075	2.13E-06	0.000223	-8.40894
266	0.214	0.1705	2.13E-06	0.000223	-8.40893
268	0.21625	0.17225	2.15E-06	0.000223	-8.40903
270	0.219	0.17575	2.2E-06	0.000223	-8.40922

272	0.2195	0.17575	2.2E-06	0.000223	-8.40922
274	0.22175	0.176	2.2E-06	0.000223	-8.40924
276	0.22325	0.179	2.24E-06	0.000223	-8.4094
278	0.225	0.1805	2.26E-06	0.000223	-8.40949
280	0.22725	0.18325	2.29E-06	0.000223	-8.40964
282	0.229	0.18525	2.32E-06	0.000223	-8.40976
284	0.23025	0.18625	2.33E-06	0.000223	-8.40981
286	0.233	0.18775	2.35E-06	0.000223	-8.4099
288	0.23175	0.18675	2.33E-06	0.000223	-8.40984
290	0.23375	0.1895	2.37E-06	0.000223	-8.40999
292	0.237	0.19275	2.41E-06	0.000223	-8.41018
294	0.238	0.193	2.41E-06	0.000223	-8.41019
296	0.2385	0.19425	2.43E-06	0.000223	-8.41026
298	0.24125	0.196	2.45E-06	0.000223	-8.41036

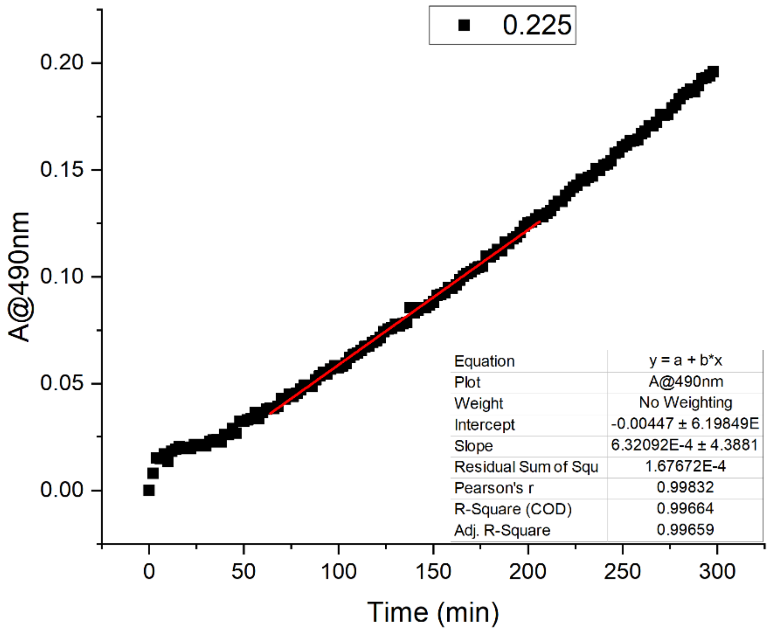

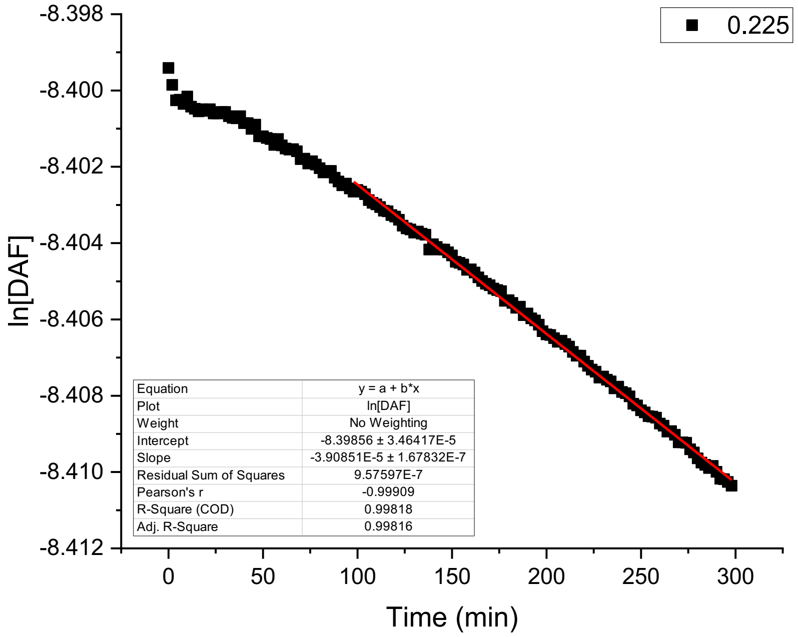

0.250 mM **DAF**

Time (min)	A@490nm minus BK	set to 0	[FLU] (M)	[DAF] (M)	ln[DAF]
0	0.05775	0	0	0.00025	-8.29405
2	0.0595	-0.00225	-2.8E-08	0.00025	-8.29394
4	0.06075	0.00225	2.81E-08	0.00025	-8.29416
6	0.0605	0.00475	5.94E-08	0.00025	-8.29429
8	0.0615	0.0055	6.88E-08	0.00025	-8.29432
10	0.06225	0.00975	1.22E-07	0.00025	-8.29454
12	0.06175	0.00875	1.09E-07	0.00025	-8.29449
14	0.06125	0.00825	1.03E-07	0.00025	-8.29446
16	0.065	0.01275	1.59E-07	0.00025	-8.29469
18	0.0625	0.01225	1.53E-07	0.00025	-8.29466
20	0.06425	0.01075	1.34E-07	0.00025	-8.29459
22	0.06475	0.011	1.38E-07	0.00025	-8.2946
24	0.06425	0.01275	1.59E-07	0.00025	-8.29469
26	0.065	0.0125	1.56E-07	0.00025	-8.29467
28	0.0655	0.00525	6.56E-08	0.00025	-8.29431
30	0.067	0.01475	1.84E-07	0.00025	-8.29479
32	0.0665	-0.00225	-2.8E-08	0.00025	-8.29394
34	0.06875	0.0105	1.31E-07	0.00025	-8.29457
36	0.0685	0.01575	1.97E-07	0.00025	-8.29484
38	0.0695	2.50E-04	3.13E-09	0.00025	-8.29406
40	0.06975	0.01075	1.34E-07	0.00025	-8.29459
42	0.07075	0.021	2.63E-07	0.00025	-8.2951
44	0.07225	0.00275	3.44E-08	0.00025	-8.29419
46	0.0715	-0.00325	-4.1E-08	0.00025	-8.29389
48	0.07275	-0.01725	-2.2E-07	0.00025	-8.29319

50	0.075	0.01525	1.91E-07	0.00025	-8.29481
52	0.07475	0.02525	3.16E-07	0.00025	-8.29531
54	0.07475	0.0245	3.06E-07	0.00025	-8.29528
56	0.076	0.0125	1.56E-07	0.00025	-8.29467
58	0.0765	0.0255	3.19E-07	0.00025	-8.29533
60	0.078	0.02725	3.41E-07	0.00025	-8.29541
62	0.08025	0.03075	3.84E-07	0.00025	-8.29559
64	0.0795	0.00325	4.06E-08	0.00025	-8.29421
66	0.0805	-7.50E-04	-9.4E-09	0.00025	-8.29401
68	0.081	0.0295	3.69E-07	0.00025	-8.29553
70	0.0815	0.02825	3.53E-07	0.00025	-8.29546
72	0.0835	0.0325	4.06E-07	0.00025	-8.29568
74	0.08525	0.035	4.38E-07	0.00025	-8.2958
76	0.08575	0.0355	4.44E-07	0.00025	-8.29583
78	0.08725	0.03475	4.34E-07	0.00025	-8.29579
80	0.08875	0.0235	2.94E-07	0.00025	-8.29523
82	0.08925	0.0235	2.94E-07	0.00025	-8.29523
84	0.0895	0.019	2.38E-07	0.00025	-8.295
86	0.09175	0.04075	5.09E-07	0.000249	-8.29609
88	0.09225	0.042	5.25E-07	0.000249	-8.29615
90	0.0945	0.04175	5.22E-07	0.000249	-8.29614
92	0.095	0.0445	5.56E-07	0.000249	-8.29628
94	0.09675	0.0455	5.69E-07	0.000249	-8.29633
96	0.097	0.045	5.63E-07	0.000249	-8.2963
98	0.099	0.02325	2.91E-07	0.00025	-8.29521
100	0.099	0.02425	3.03E-07	0.00025	-8.29526
102	0.09975	0.03075	3.84E-07	0.00025	-8.29559
104	0.10125	0.0425	5.31E-07	0.000249	-8.29618
106	0.10275	0.05275	6.59E-07	0.000249	-8.29669
108	0.105	0.05325	6.66E-07	0.000249	-8.29672
110	0.106	0.03325	4.16E-07	0.00025	-8.29571
112	0.10725	0.0535	6.69E-07	0.000249	-8.29673
114	0.10825	0.04075	5.09E-07	0.000249	-8.29609
116	0.10975	0.043	5.38E-07	0.000249	-8.2962
118	0.11175	0.04475	5.59E-07	0.000249	-8.29629
120	0.11225	0.04025	5.03E-07	0.000249	-8.29606
122	0.1135	0.04775	5.97E-07	0.000249	-8.29644
124	0.115	0.06475	8.09E-07	0.000249	-8.29729
126	0.1175	0.035	4.38E-07	0.00025	-8.2958
128	0.11975	0.0565	7.06E-07	0.000249	-8.29688
130	0.1205	0.059	7.38E-07	0.000249	-8.297
132	0.12325	0.07175	8.97E-07	0.000249	-8.29764
134	0.12275	0.0705	8.81E-07	0.000249	-8.29758
136	0.12325	0.0415	5.19E-07	0.000249	-8.29613
138	0.1265	0.03825	4.78E-07	0.00025	-8.29596
140	0.12575	0.074	9.25E-07	0.000249	-8.29776

142	0.12725	0.07525	9.41E-07	0.000249	-8.29782
144	0.12975	0.074	9.25E-07	0.000249	-8.29776
146	0.13	0.064	8E-07	0.000249	-8.29725
148	0.13325	0.079	9.88E-07	0.000249	-8.29801
150	0.134	0.0745	9.31E-07	0.000249	-8.29778
152	0.136	0.08425	1.05E-06	0.000249	-8.29827
154	0.138	0.0795	9.94E-07	0.000249	-8.29803
156	0.1395	0.06	7.5E-07	0.000249	-8.29705
158	0.14125	0.088	1.1E-06	0.000249	-8.29846
160	0.141	0.05225	6.53E-07	0.000249	-8.29667
162	0.142	0.0625	7.81E-07	0.000249	-8.29718
164	0.14425	0.09075	1.13E-06	0.000249	-8.2986
166	0.146	0.08875	1.11E-06	0.000249	-8.2985
168	0.14675	0.069	8.63E-07	0.000249	-8.29751
170	0.14875	0.07975	9.97E-07	0.000249	-8.29805
172	0.148	0.0955	1.19E-06	0.000249	-8.29884
174	0.151	0.09825	1.23E-06	0.000249	-8.29897
176	0.15375	0.0985	1.23E-06	0.000249	-8.29899
178	0.15425	0.10125	1.27E-06	0.000249	-8.29912
180	0.15475	0.08675	1.08E-06	0.000249	-8.2984
182	0.15775	0.0975	1.22E-06	0.000249	-8.29894
184	0.158	0.1045	1.31E-06	0.000249	-8.29929
186	0.1605	0.10575	1.32E-06	0.000249	-8.29935
188	0.16175	0.10775	1.35E-06	0.000249	-8.29945
190	0.16275	0.10975	1.37E-06	0.000249	-8.29955
192	0.16525	0.11175	1.4E-06	0.000249	-8.29965
194	0.16675	0.11325	1.42E-06	0.000249	-8.29973
196	0.16775	0.1145	1.43E-06	0.000249	-8.29979
198	0.171	0.11825	1.48E-06	0.000249	-8.29998
200	0.1715	0.11075	1.38E-06	0.000249	-8.2996
202	0.17625	0.10225	1.28E-06	0.000249	-8.29918
204	0.17375	0.1195	1.49E-06	0.000249	-8.30004
206	0.17475	0.1215	1.52E-06	0.000248	-8.30014
208	0.17675	0.1195	1.49E-06	0.000249	-8.30004
210	0.179	0.1055	1.32E-06	0.000249	-8.29934
212	0.17975	0.111	1.39E-06	0.000249	-8.29962
214	0.1825	0.0925	1.16E-06	0.000249	-8.29869
216	0.18325	0.1035	1.29E-06	0.000249	-8.29924
218	0.18625	0.13075	1.63E-06	0.000248	-8.30061
220	0.18675	0.13075	1.63E-06	0.000248	-8.30061
222	0.18875	0.1245	1.56E-06	0.000248	-8.30029
224	0.18925	0.13025	1.63E-06	0.000248	-8.30058
226	0.193	0.11225	1.4E-06	0.000249	-8.29968
228	0.193	0.10225	1.28E-06	0.000249	-8.29918
230	0.195	0.13925	1.74E-06	0.000248	-8.30104
232	0.1955	0.1395	1.74E-06	0.000248	-8.30105

234	0.1975	0.128	1.6E-06	0.000248	-8.30047
236	0.199	0.1435	1.79E-06	0.000248	-8.30125
238	0.19975	0.14475	1.81E-06	0.000248	-8.30131
240	0.20375	0.117	1.46E-06	0.000249	-8.29992
242	0.2055	0.1455	1.82E-06	0.000248	-8.30135
244	0.20425	0.14875	1.86E-06	0.000248	-8.30151
246	0.20825	0.15325	1.92E-06	0.000248	-8.30174
248	0.20875	0.153	1.91E-06	0.000248	-8.30173
250	0.20925	0.154	1.93E-06	0.000248	-8.30178
252	0.21175	0.15525	1.94E-06	0.000248	-8.30184
254	0.2145	0.1555	1.94E-06	0.000248	-8.30186
256	0.21525	0.15	1.88E-06	0.000248	-8.30158
258	0.21675	0.15925	1.99E-06	0.000248	-8.30204
260	0.21875	0.162	2.03E-06	0.000248	-8.30218
262	0.21975	0.163	2.04E-06	0.000248	-8.30223
264	0.222	0.1655	2.07E-06	0.000248	-8.30236
266	0.223	0.167	2.09E-06	0.000248	-8.30243
268	0.22525	0.16825	2.1E-06	0.000248	-8.3025
270	0.228	0.17	2.13E-06	0.000248	-8.30259
272	0.2275	0.17	2.13E-06	0.000248	-8.30259
274	0.23025	0.139	1.74E-06	0.000248	-8.30102
276	0.2315	0.16825	2.1E-06	0.000248	-8.3025
278	0.233	0.17525	2.19E-06	0.000248	-8.30285
280	0.23575	0.1775	2.22E-06	0.000248	-8.30296
282	0.23875	0.182	2.28E-06	0.000248	-8.30319
284	0.23925	0.18175	2.27E-06	0.000248	-8.30318
286	0.23975	0.1815	2.27E-06	0.000248	-8.30317
288	0.24125	0.1775	2.22E-06	0.000248	-8.30296
290	0.24425	0.18725	2.34E-06	0.000248	-8.30346
292	0.2455	0.18775	2.35E-06	0.000248	-8.30348
294	0.246	0.189	2.36E-06	0.000248	-8.30354
296	0.24875	0.1675	2.09E-06	0.000248	-8.30246
298	0.24975	0.19125	2.39E-06	0.000248	-8.30366

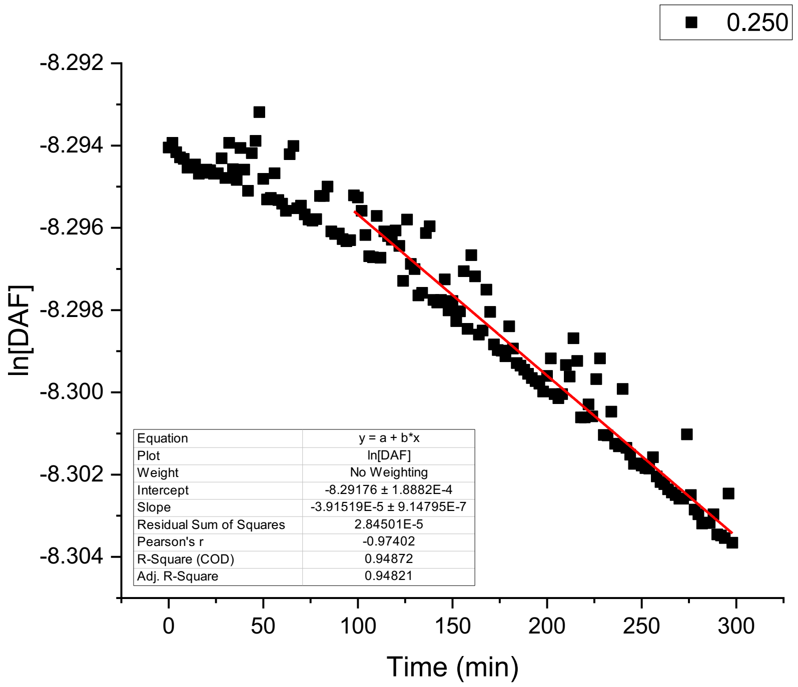

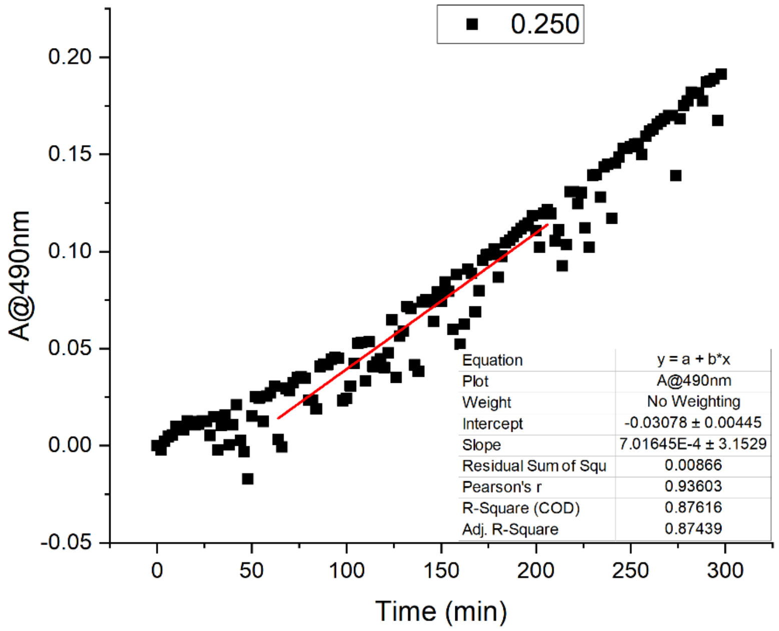

0.275 mM **DAF**

Time (min)	A@490nm minus BK	set to 0	[FLU] (M)	[DAF] (M)	ln[DAF]
0	0.06125	0	0	0.000275	-8.19874
2	0.06275	0.0045	5.63E-08	0.000275	-8.19894
4	0.06275	0.00375	4.69E-08	0.000275	-8.19891
6	0.06425	-0.00875	-1.1E-07	0.000275	-8.19834
8	0.06425	-0.0045	-5.6E-08	0.000275	-8.19853
10	0.06475	0.00625	7.81E-08	0.000275	-8.19902

12	0.06575	-0.00875	-1.1E-07	0.000275	-8.19834
14	0.065	0	0	0.000275	-8.19874
16	0.0665	-0.009	-1.1E-07	0.000275	-8.19833
18	0.06525	0.0065	8.13E-08	0.000275	-8.19903
20	0.06525	0.0035	4.38E-08	0.000275	-8.1989
22	0.0665	0.0025	3.13E-08	0.000275	-8.19885
24	0.0665	2.50E-04	3.13E-09	0.000275	-8.19875
26	0.0675	0.00325	4.06E-08	0.000275	-8.19889
28	0.06875	0.009	1.13E-07	0.000275	-8.19915
30	0.07	0.00425	5.31E-08	0.000275	-8.19893
32	0.0695	0.00675	8.44E-08	0.000275	-8.19905
34	0.07	0.0035	4.38E-08	0.000275	-8.1989
36	0.071	0.00725	9.06E-08	0.000275	-8.19907
38	0.0735	0.00425	5.31E-08	0.000275	-8.19893
40	0.0735	0.00275	3.44E-08	0.000275	-8.19886
42	0.0765	0.00525	6.56E-08	0.000275	-8.19898
44	0.0745	0.0125	1.56E-07	0.000275	-8.19931
46	0.07425	0.007	8.75E-08	0.000275	-8.19906
48	0.07625	0.00175	2.19E-08	0.000275	-8.19882
50	0.07725	-5.00E-04	-6.3E-09	0.000275	-8.19872
52	0.0765	0.00525	6.56E-08	0.000275	-8.19898
54	0.078	0.00975	1.22E-07	0.000275	-8.19918
56	0.08025	0.00625	7.81E-08	0.000275	-8.19902
58	0.08025	0.017	2.13E-07	0.000275	-8.19951
60	0.08125	0.01975	2.47E-07	0.000275	-8.19964
62	0.0825	0.005	6.25E-08	0.000275	-8.19897
64	0.0835	0.02125	2.66E-07	0.000275	-8.19971
66	0.085	0.02175	2.72E-07	0.000275	-8.19973
68	0.084	0.007	8.75E-08	0.000275	-8.19906
70	0.0855	0.00725	9.06E-08	0.000275	-8.19907
72	0.0865	0.0245	3.06E-07	0.000275	-8.19985
74	0.08725	0.02175	2.72E-07	0.000275	-8.19973
76	0.08875	0.016	2E-07	0.000275	-8.19947
78	0.09025	0.02375	2.97E-07	0.000275	-8.19982
80	0.091	0.01725	2.16E-07	0.000275	-8.19952
82	0.0915	0.02575	3.22E-07	0.000275	-8.19991
84	0.091	0.01625	2.03E-07	0.000275	-8.19948
86	0.0955	0.0225	2.81E-07	0.000275	-8.19976
88	0.095	0.0305	3.81E-07	0.000275	-8.20013
90	0.09625	0.019	2.38E-07	0.000275	-8.1996
92	0.09725	0.026	3.25E-07	0.000275	-8.19992
94	0.09925	0.02	2.5E-07	0.000275	-8.19965
96	0.1	0.033	4.13E-07	0.000275	-8.20024
98	0.1025	0.0265	3.31E-07	0.000275	-8.19994
100	0.10275	0.0285	3.56E-07	0.000275	-8.20004
102	0.104	0.03475	4.34E-07	0.000275	-8.20032

104	0.1045	0.037	4.63E-07	0.000275	-8.20042
106	0.107	0.04025	5.03E-07	0.000274	-8.20057
108	0.109	0.04075	5.09E-07	0.000274	-8.20059
110	0.1085	0.0295	3.69E-07	0.000275	-8.20008
112	0.10975	0.03175	3.97E-07	0.000275	-8.20018
114	0.1105	0.04575	5.72E-07	0.000274	-8.20082
116	0.1125	0.04675	5.84E-07	0.000274	-8.20087
118	0.1145	0.03025	3.78E-07	0.000275	-8.20012
120	0.11575	0.03575	4.47E-07	0.000275	-8.20037
122	0.117	0.044	5.5E-07	0.000274	-8.20074
124	0.1175	0.0395	4.94E-07	0.000275	-8.20054
126	0.11975	0.0455	5.69E-07	0.000274	-8.20081
128	0.1215	0.05225	6.53E-07	0.000274	-8.20112
130	0.124	0.0485	6.06E-07	0.000274	-8.20095
132	0.12375	0.05	6.25E-07	0.000274	-8.20101
134	0.12575	0.04425	5.53E-07	0.000274	-8.20075
136	0.12625	0.05425	6.78E-07	0.000274	-8.20121
138	0.129	0.0495	6.19E-07	0.000274	-8.20099
140	0.12875	0.0625	7.81E-07	0.000274	-8.20158
142	0.131	0.06425	8.03E-07	0.000274	-8.20166
144	0.13125	0.05025	6.28E-07	0.000274	-8.20103
146	0.13275	0.06025	7.53E-07	0.000274	-8.20148
148	0.1355	0.06275	7.84E-07	0.000274	-8.2016
150	0.13625	0.0575	7.19E-07	0.000274	-8.20136
152	0.137	0.05725	7.16E-07	0.000274	-8.20135
154	0.13975	0.06975	8.72E-07	0.000274	-8.20191
156	0.14075	0.067	8.38E-07	0.000274	-8.20179
158	0.1425	0.07525	9.41E-07	0.000274	-8.20217
160	0.144	0.07625	9.53E-07	0.000274	-8.20221
162	0.14525	0.0685	8.56E-07	0.000274	-8.20186
164	0.1465	0.06825	8.53E-07	0.000274	-8.20185
166	0.1475	0.079	9.88E-07	0.000274	-8.20234
168	0.1495	0.08225	1.03E-06	0.000274	-8.20249
170	0.151	0.08225	1.03E-06	0.000274	-8.20249
172	0.1515	0.082	1.03E-06	0.000274	-8.20247
174	0.15475	0.0875	1.09E-06	0.000274	-8.20272
176	0.156	0.075	9.38E-07	0.000274	-8.20215
178	0.15725	0.082	1.03E-06	0.000274	-8.20247
180	0.157	0.08825	1.1E-06	0.000274	-8.20276
182	0.1595	0.0785	9.81E-07	0.000274	-8.20231
184	0.16175	0.09075	1.13E-06	0.000274	-8.20287
186	0.1625	0.08425	1.05E-06	0.000274	-8.20258
188	0.1655	0.08725	1.09E-06	0.000274	-8.20271
190	0.168	0.098	1.23E-06	0.000274	-8.2032
192	0.16875	0.093	1.16E-06	0.000274	-8.20298
194	0.17	0.088	1.1E-06	0.000274	-8.20275

196	0.171	0.09275	1.16E-06	0.000274	-8.20296
198	0.17425	0.10625	1.33E-06	0.000274	-8.20358
200	0.1745	0.091	1.14E-06	0.000274	-8.20288
202	0.176	0.098	1.23E-06	0.000274	-8.2032
204	0.17725	0.10275	1.28E-06	0.000274	-8.20342
206	0.17925	0.099	1.24E-06	0.000274	-8.20325
208	0.18	0.09725	1.22E-06	0.000274	-8.20317
210	0.1815	0.10325	1.29E-06	0.000274	-8.20344
212	0.1835	0.10575	1.32E-06	0.000274	-8.20356
214	0.18525	0.111	1.39E-06	0.000274	-8.2038
216	0.187	0.11375	1.42E-06	0.000274	-8.20392
218	0.18725	0.10825	1.35E-06	0.000274	-8.20367
220	0.1905	0.118	1.48E-06	0.000274	-8.20412
222	0.19125	0.109	1.36E-06	0.000274	-8.20371
224	0.19275	0.10925	1.37E-06	0.000274	-8.20372
226	0.19475	0.1135	1.42E-06	0.000274	-8.20391
228	0.19575	0.11275	1.41E-06	0.000274	-8.20388
230	0.19625	0.114	1.43E-06	0.000274	-8.20393
232	0.19975	0.11875	1.48E-06	0.000274	-8.20415
234	0.20075	0.119	1.49E-06	0.000274	-8.20416
236	0.2025	0.11775	1.47E-06	0.000274	-8.20411
238	0.203	0.1315	1.64E-06	0.000273	-8.20473
240	0.205	0.12825	1.6E-06	0.000273	-8.20459
242	0.20725	0.126	1.58E-06	0.000273	-8.20448
244	0.209	0.1375	1.72E-06	0.000273	-8.20501
246	0.2105	0.125	1.56E-06	0.000273	-8.20444
248	0.21225	0.132	1.65E-06	0.000273	-8.20476
250	0.214	0.13225	1.65E-06	0.000273	-8.20477
252	0.21625	0.13475	1.68E-06	0.000273	-8.20488
254	0.21675	0.13775	1.72E-06	0.000273	-8.20502
256	0.21925	0.14825	1.85E-06	0.000273	-8.2055
258	0.22	0.14875	1.86E-06	0.000273	-8.20552
260	0.2225	0.143	1.79E-06	0.000273	-8.20526
262	0.22425	0.14275	1.78E-06	0.000273	-8.20525
264	0.2265	0.13925	1.74E-06	0.000273	-8.20509
266	0.22575	0.141	1.76E-06	0.000273	-8.20517
268	0.22825	0.14	1.75E-06	0.000273	-8.20512
270	0.23025	0.149	1.86E-06	0.000273	-8.20554
272	0.2325	0.1445	1.81E-06	0.000273	-8.20533
274	0.2335	0.15625	1.95E-06	0.000273	-8.20587
276	0.2355	0.14775	1.85E-06	0.000273	-8.20548
278	0.237	0.15825	1.98E-06	0.000273	-8.20596
280	0.23925	0.156	1.95E-06	0.000273	-8.20586
282	0.242	0.161	2.01E-06	0.000273	-8.20608
284	0.24175	0.16975	2.12E-06	0.000273	-8.20649
286	0.24275	0.1615	2.02E-06	0.000273	-8.20611

288	0.2455	0.16175	2.02E-06	0.000273	-8.20612
290	0.24725	0.174	2.18E-06	0.000273	-8.20668
292	0.24875	0.165	2.06E-06	0.000273	-8.20627
294	0.249	0.17	2.13E-06	0.000273	-8.2065
296	0.25275	0.164	2.05E-06	0.000273	-8.20622
298	0.2545	0.17925	2.24E-06	0.000273	-8.20692

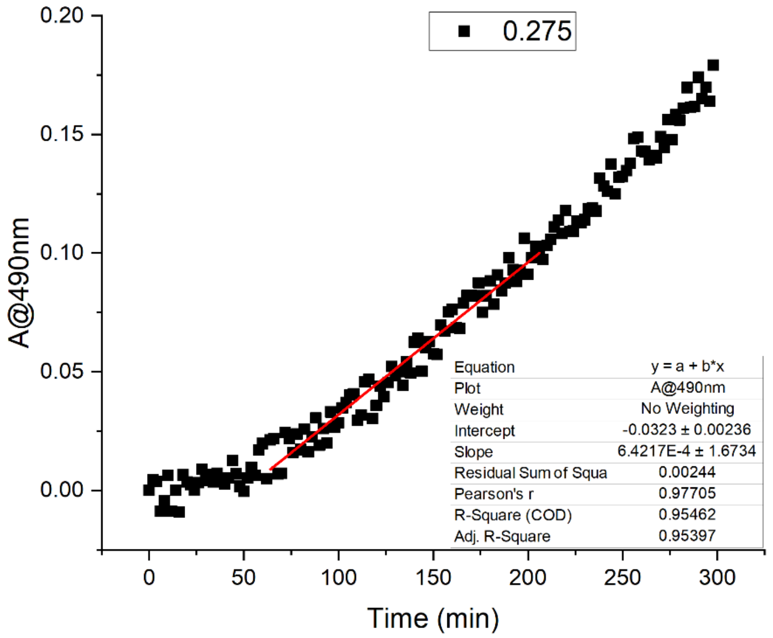

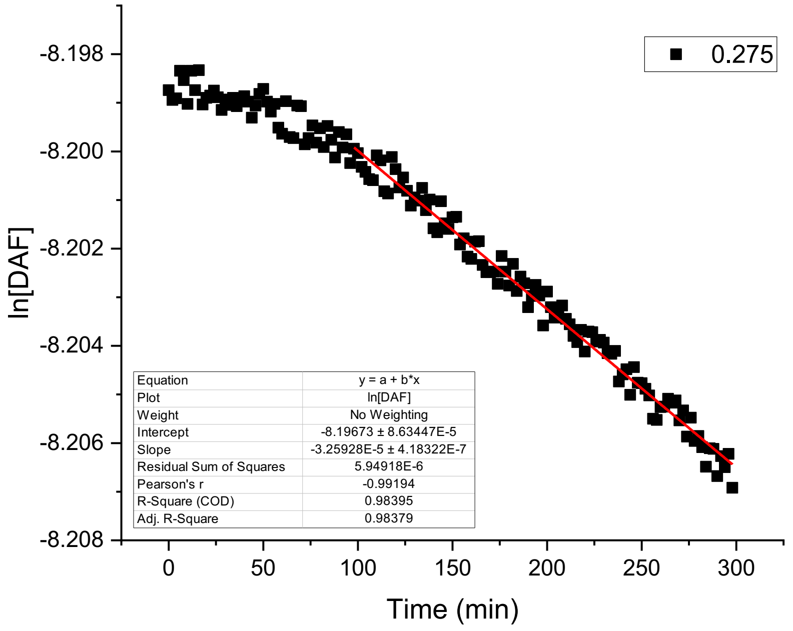

0.300 mM **DAF**

Time (min)	A@490nm minus BK	set to 0	[FLU] (M)	[DAF] (M)	ln[DAF]
0	0.0585	0	0	0.0003	-8.11173
2	0.06225	0.00925	1.16E-07	0.0003	-8.11211
4	0.0635	0.00875	1.09E-07	0.0003	-8.11209
6	0.062	0.0095	1.19E-07	0.0003	-8.11212
8	0.062	0.00875	1.09E-07	0.0003	-8.11209
10	0.06275	0.00625	7.81E-08	0.0003	-8.11199
12	0.06575	0.01175	1.47E-07	0.0003	-8.11222
14	0.065	0.0105	1.31E-07	0.0003	-8.11217
16	0.06525	0.008	1E-07	0.0003	-8.11206
18	0.0665	0.0055	6.88E-08	0.0003	-8.11196
20	0.066	0.00325	4.06E-08	0.0003	-8.11186
22	0.06725	0.0055	6.88E-08	0.0003	-8.11196
24	0.0675	0.00475	5.94E-08	0.0003	-8.11193
26	0.06975	0.005	6.25E-08	0.0003	-8.11194
28	0.07125	0.00875	1.09E-07	0.0003	-8.11209
30	0.0715	0.00875	1.09E-07	0.0003	-8.11209
32	0.07075	0.00625	7.81E-08	0.0003	-8.11199
34	0.072	0.00975	1.22E-07	0.0003	-8.11213
36	0.07225	0.01025	1.28E-07	0.0003	-8.11216
38	0.073	0.0105	1.31E-07	0.0003	-8.11217
40	0.07375	0.0115	1.44E-07	0.0003	-8.11221
42	0.07625	0.0115	1.44E-07	0.0003	-8.11221
44	0.0765	0.0135	1.69E-07	0.0003	-8.11229
46	0.077	0.012	1.5E-07	0.0003	-8.11223
48	0.07825	0.016	2E-07	0.0003	-8.11239
50	0.07825	0.01075	1.34E-07	0.0003	-8.11218
52	0.078	0.01775	2.22E-07	0.0003	-8.11247
54	0.08025	0.01475	1.84E-07	0.0003	-8.11234
56	0.08075	0.019	2.38E-07	0.0003	-8.11252
58	0.08325	0.0205	2.56E-07	0.0003	-8.11258
60	0.082	0.01925	2.41E-07	0.0003	-8.11253
62	0.085	0.02325	2.91E-07	0.0003	-8.1127
64	0.0865	0.02125	2.66E-07	0.0003	-8.11261
66	0.08725	0.02275	2.84E-07	0.0003	-8.11268
68	0.08775	0.02275	2.84E-07	0.0003	-8.11268
70	0.08875	0.0265	3.31E-07	0.0003	-8.11283
72	0.09	0.0265	3.31E-07	0.0003	-8.11283
74	0.09175	0.02925	3.66E-07	0.0003	-8.11295
76	0.0915	0.0255	3.19E-07	0.0003	-8.11279
78	0.093	0.03075	3.84E-07	0.0003	-8.11301
80	0.094	0.0305	3.81E-07	0.0003	-8.113
82	0.097	0.03425	4.28E-07	0.0003	-8.11316
84	0.09475	0.02875	3.59E-07	0.0003	-8.11293
86	0.09575	0.03175	3.97E-07	0.0003	-8.11305

88	0.098	0.03475	4.34E-07	0.0003	-8.11318
90	0.09875	0.0345	4.31E-07	0.0003	-8.11317
92	0.101	0.03725	4.66E-07	0.0003	-8.11328
94	0.10225	0.0375	4.69E-07	0.0003	-8.11329
96	0.10425	0.041	5.13E-07	0.000299	-8.11344
98	0.10375	0.03975	4.97E-07	0.0003	-8.11339
100	0.1045	0.04125	5.16E-07	0.000299	-8.11345
102	0.1075	0.04325	5.41E-07	0.000299	-8.11353
104	0.10925	0.04575	5.72E-07	0.000299	-8.11364
106	0.10925	0.046	5.75E-07	0.000299	-8.11365
108	0.1115	0.0485	6.06E-07	0.000299	-8.11375
110	0.11175	0.0465	5.81E-07	0.000299	-8.11367
112	0.114	0.0505	6.31E-07	0.000299	-8.11383
114	0.1155	0.051	6.38E-07	0.000299	-8.11386
116	0.116	0.0525	6.56E-07	0.000299	-8.11392
118	0.1175	0.05325	6.66E-07	0.000299	-8.11395
120	0.1195	0.0555	6.94E-07	0.000299	-8.11404
122	0.121	0.0565	7.06E-07	0.000299	-8.11409
124	0.12075	0.05775	7.22E-07	0.000299	-8.11414
126	0.12325	0.0585	7.31E-07	0.000299	-8.11417
128	0.12475	0.06075	7.59E-07	0.000299	-8.11426
130	0.12675	0.0615	7.69E-07	0.000299	-8.11429
132	0.1275	0.063	7.88E-07	0.000299	-8.11436
134	0.1295	0.06525	8.16E-07	0.000299	-8.11445
136	0.13	0.0655	8.19E-07	0.000299	-8.11446
138	0.132	0.072	9E-07	0.000299	-8.11473
140	0.13225	0.0615	7.69E-07	0.000299	-8.11429
142	0.13475	0.07075	8.84E-07	0.000299	-8.11468
144	0.1365	0.07125	8.91E-07	0.000299	-8.1147
146	0.13675	0.0715	8.94E-07	0.000299	-8.11471
148	0.1395	0.0735	9.19E-07	0.000299	-8.1148
150	0.1395	0.0755	9.44E-07	0.000299	-8.11488
152	0.1425	0.07825	9.78E-07	0.000299	-8.11499
154	0.1435	0.0785	9.81E-07	0.000299	-8.115
156	0.14475	0.08075	1.01E-06	0.000299	-8.1151
158	0.14625	0.0815	1.02E-06	0.000299	-8.11513
160	0.14725	0.082	1.03E-06	0.000299	-8.11515
162	0.15025	0.084	1.05E-06	0.000299	-8.11523
164	0.15275	0.0855	1.07E-06	0.000299	-8.1153
166	0.15225	0.087	1.09E-06	0.000299	-8.11536
168	0.15425	0.089	1.11E-06	0.000299	-8.11544
170	0.15575	0.0915	1.14E-06	0.000299	-8.11555
172	0.15675	0.091	1.14E-06	0.000299	-8.11553
174	0.15825	0.093	1.16E-06	0.000299	-8.11561
176	0.15925	0.09275	1.16E-06	0.000299	-8.1156
178	0.16075	0.09325	1.17E-06	0.000299	-8.11562

180	0.161	0.096	1.2E-06	0.000299	-8.11574
182	0.163	0.097	1.21E-06	0.000299	-8.11578
184	0.1665	0.1005	1.26E-06	0.000299	-8.11592
186	0.1685	0.10375	1.3E-06	0.000299	-8.11606
188	0.1705	0.1055	1.32E-06	0.000299	-8.11613
190	0.1705	0.10625	1.33E-06	0.000299	-8.11616
192	0.17325	0.10825	1.35E-06	0.000299	-8.11625
194	0.174	0.1085	1.36E-06	0.000299	-8.11626
196	0.176	0.108	1.35E-06	0.000299	-8.11624
198	0.1775	0.1025	1.28E-06	0.000299	-8.11601
200	0.17975	0.11425	1.43E-06	0.000299	-8.1165
202	0.1815	0.11675	1.46E-06	0.000299	-8.1166
204	0.1835	0.1175	1.47E-06	0.000299	-8.11664
206	0.18425	0.119	1.49E-06	0.000299	-8.1167
208	0.1855	0.1205	1.51E-06	0.000298	-8.11676
210	0.18725	0.12275	1.53E-06	0.000298	-8.11686
212	0.188	0.122	1.53E-06	0.000298	-8.11682
214	0.19	0.1245	1.56E-06	0.000298	-8.11693
216	0.19125	0.1255	1.57E-06	0.000298	-8.11697
218	0.19325	0.1245	1.56E-06	0.000298	-8.11693
220	0.19725	0.13	1.63E-06	0.000298	-8.11716
222	0.19625	0.12875	1.61E-06	0.000298	-8.11711
224	0.19825	0.133	1.66E-06	0.000298	-8.11729
226	0.201	0.13375	1.67E-06	0.000298	-8.11732
228	0.2025	0.1375	1.72E-06	0.000298	-8.11747
230	0.2025	0.13675	1.71E-06	0.000298	-8.11744
232	0.20475	0.13825	1.73E-06	0.000298	-8.11751
234	0.20475	0.1385	1.73E-06	0.000298	-8.11752
236	0.20875	0.1435	1.79E-06	0.000298	-8.11773
238	0.2095	0.14225	1.78E-06	0.000298	-8.11767
240	0.21125	0.1435	1.79E-06	0.000298	-8.11773
242	0.2125	0.14625	1.83E-06	0.000298	-8.11784
244	0.216	0.14925	1.87E-06	0.000298	-8.11797
246	0.217	0.15125	1.89E-06	0.000298	-8.11805
248	0.21925	0.15275	1.91E-06	0.000298	-8.11811
250	0.22125	0.1545	1.93E-06	0.000298	-8.11819
252	0.22225	0.1555	1.94E-06	0.000298	-8.11823
254	0.224	0.157	1.96E-06	0.000298	-8.11829
256	0.226	0.15775	1.97E-06	0.000298	-8.11832
258	0.22725	0.1595	1.99E-06	0.000298	-8.1184
260	0.22825	0.16075	2.01E-06	0.000298	-8.11845
262	0.2295	0.161	2.01E-06	0.000298	-8.11846
264	0.233	0.16475	2.06E-06	0.000298	-8.11862
266	0.23325	0.16625	2.08E-06	0.000298	-8.11868
268	0.2345	0.16675	2.08E-06	0.000298	-8.1187
270	0.23675	0.1685	2.11E-06	0.000298	-8.11877

272	0.2395	0.17025	2.13E-06	0.000298	-8.11885
274	0.24125	0.17275	2.16E-06	0.000298	-8.11895
276	0.24375	0.17525	2.19E-06	0.000298	-8.11906
278	0.2445	0.1765	2.21E-06	0.000298	-8.11911
280	0.2465	0.17775	2.22E-06	0.000298	-8.11916
282	0.2485	0.1795	2.24E-06	0.000298	-8.11924
284	0.249	0.18125	2.27E-06	0.000298	-8.11931
286	0.251	0.18025	2.25E-06	0.000298	-8.11927
288	0.25375	0.18425	2.3E-06	0.000298	-8.11943
290	0.25475	0.186	2.33E-06	0.000298	-8.11951
292	0.257	0.1885	2.36E-06	0.000298	-8.11961
294	0.258	0.189	2.36E-06	0.000298	-8.11963
296	0.26025	0.19075	2.38E-06	0.000298	-8.11971
298	0.26125	0.19125	2.39E-06	0.000298	-8.11973

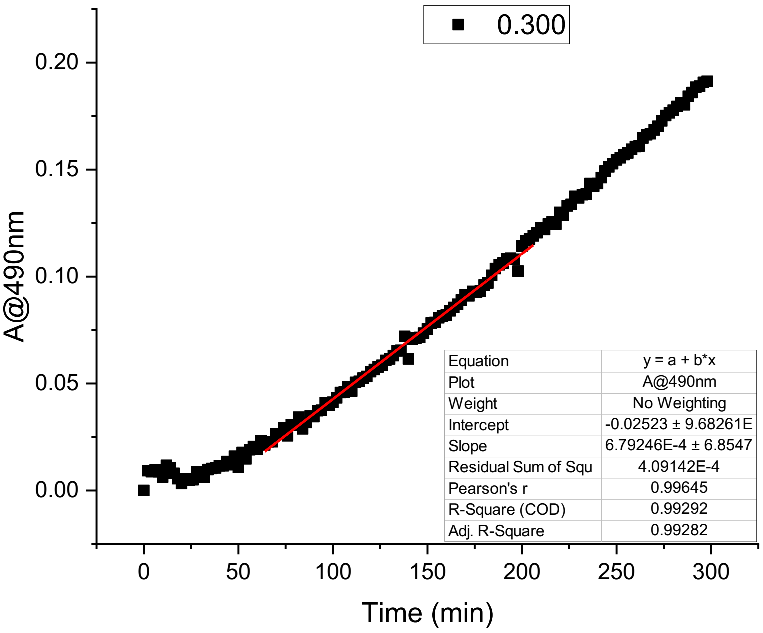

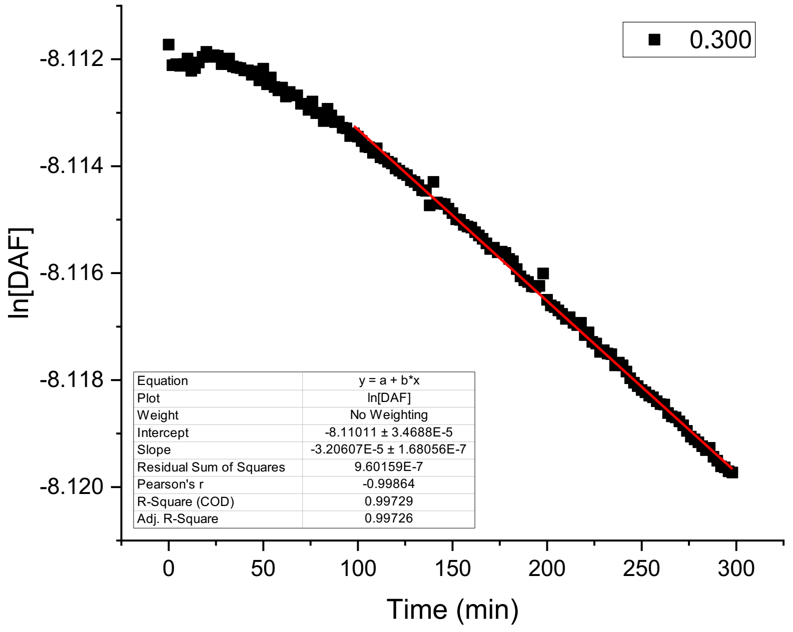

Catalysis of **DAF** to **FLU** with concentrations of **CoH^{w}Cl** between 0.05-0.150 mM ([**DAF**]=0.065 mM , 50 mM PBS buffer pH 7)

0.05 mM CoHwCl, 50 mM PBS pH 7, [DAF]=0.065 mM

Time (min)	A@490nm	A@490nm minus BK	set to 0	[FLU] (M)	[DAF] (M)	ln[DAF]
0	0.021	0.01025	0	0	0.000065	-9.64112
2	0.01875	0.0095	-0.00075	-6.3E-09	6.5E-05	-9.64103
4	0.019	0.011	0.00075	1.25E-08	6.5E-05	-9.64132
6	0.02	0.01225	0.002	3.13E-08	6.5E-05	-9.6416
8	0.0195	0.01175	0.0015	3.44E-08	6.5E-05	-9.64165
10	0.02	0.00825	-0.002	-2.5E-08	6.5E-05	-9.64074
12	0.0195	0.01475	0.0045	5.94E-08	6.49E-05	-9.64204
14	0.02075	0.015763	0.005513	6.89E-08	6.49E-05	-9.64218
16	0.01925	0.01375	0.0035	4.69E-08	6.5E-05	-9.64184
18	0.02025	0.01575	0.0055	6.88E-08	6.49E-05	-9.64218
20	0.0195	0.016	0.00575	8.44E-08	6.49E-05	-9.64242
22	0.02025	0.01525	0.005	6.56E-08	6.49E-05	-9.64213
24	0.02	0.015	0.00475	6.56E-08	6.49E-05	-9.64213
26	0.02125	0.01625	0.006	7.19E-08	6.49E-05	-9.64223
28	0.02225	0.01675	0.0065	8.13E-08	6.49E-05	-9.64237
30	0.02125	0.01575	0.0055	8.13E-08	6.49E-05	-9.64237
32	0.02175	0.0165	0.00625	8.75E-08	6.49E-05	-9.64247
34	0.02225	0.01725	0.007	9.69E-08	6.49E-05	-9.64261
36	0.023	0.01725	0.007	8.75E-08	6.49E-05	-9.64247
38	0.0225	0.01725	0.007	9.69E-08	6.49E-05	-9.64261

40	0.024	0.018	0.00775	9.06E-08	6.49E-05	-9.64252
42	0.024	0.018	0.00775	1E-07	6.49E-05	-9.64266
44	0.024	0.0185	0.00825	1.13E-07	6.49E-05	-9.64286
46	0.02475	0.0195	0.00925	1.16E-07	6.49E-05	-9.6429
48	0.02575	0.01975	0.0095	1.25E-07	6.49E-05	-9.64305
50	0.0255	0.0205	0.01025	1.31E-07	6.49E-05	-9.64314
52	0.0265	0.0205	0.01025	1.34E-07	6.49E-05	-9.64319
54	0.026	0.02025	0.01	1.34E-07	6.49E-05	-9.64319
56	0.026	0.02	0.00975	1.38E-07	6.49E-05	-9.64324
58	0.02675	0.01925	0.009	1.28E-07	6.49E-05	-9.6431
60	0.027	0.021	0.01075	1.53E-07	6.48E-05	-9.64348
62	0.02675	0.02075	0.0105	1.5E-07	6.49E-05	-9.64343
64	0.02725	0.0215	0.01125	1.63E-07	6.48E-05	-9.64363
66	0.0285	0.023	0.01275	1.66E-07	6.48E-05	-9.64367
68	0.028	0.02225	0.012	1.75E-07	6.48E-05	-9.64382
70	0.0295	0.02325	0.013	1.84E-07	6.48E-05	-9.64396
72	0.029	0.0235	0.01325	1.94E-07	6.48E-05	-9.64411
74	0.03	0.0235	0.01325	1.97E-07	6.48E-05	-9.64416
76	0.0325	0.0265	0.01625	2E-07	6.48E-05	-9.6442
78	0.03225	0.026	0.01575	2.09E-07	6.48E-05	-9.64435
80	0.03175	0.026	0.01575	2.09E-07	6.48E-05	-9.64435
82	0.03275	0.0265	0.01625	2.22E-07	6.48E-05	-9.64454
84	0.03325	0.02725	0.017	2.31E-07	6.48E-05	-9.64469
86	0.0335	0.02775	0.0175	2.34E-07	6.48E-05	-9.64474
88	0.03325	0.0285	0.01825	2.5E-07	6.48E-05	-9.64498
90	0.03475	0.02925	0.019	2.53E-07	6.47E-05	-9.64503
92	0.0355	0.0295	0.01925	2.56E-07	6.47E-05	-9.64507
94	0.0355	0.02975	0.0195	2.75E-07	6.47E-05	-9.64536
96	0.036	0.0305	0.02025	2.78E-07	6.47E-05	-9.64541
98	0.03725	0.03175	0.0215	2.91E-07	6.47E-05	-9.6456
100	0.0375	0.03175	0.0215	2.91E-07	6.47E-05	-9.6456
102	0.0375	0.03175	0.0215	3E-07	6.47E-05	-9.64575
104	0.03825	0.03175	0.0215	3.06E-07	6.47E-05	-9.64585
106	0.04175	0.037	0.02675	3.25E-07	6.47E-05	-9.64614
108	0.03975	0.03425	0.024	3.31E-07	6.47E-05	-9.64623
110	0.0425	0.03725	0.027	3.38E-07	6.47E-05	-9.64633
112	0.0415	0.03525	0.025	3.41E-07	6.47E-05	-9.64638
114	0.04075	0.035	0.02475	3.41E-07	6.47E-05	-9.64638
116	0.0415	0.03725	0.027	3.78E-07	6.46E-05	-9.64696
118	0.042	0.0365	0.02625	3.72E-07	6.46E-05	-9.64686
120	0.04275	0.0375	0.02725	3.72E-07	6.46E-05	-9.64686
122	0.044	0.0375	0.02725	3.72E-07	6.46E-05	-9.64686
124	0.04475	0.03875	0.0285	3.94E-07	6.46E-05	-9.6472
126	0.04475	0.03975	0.0295	4.03E-07	6.46E-05	-9.64734
128	0.04425	0.039	0.02875	3.94E-07	6.46E-05	-9.6472
130	0.0455	0.04	0.02975	4.09E-07	6.46E-05	-9.64744

132	0.04525	0.0405	0.03025	4.25E-07	6.46E-05	-9.64768
134	0.0455	0.04025	0.03	4.19E-07	6.46E-05	-9.64759
136	0.048	0.042	0.03175	4.44E-07	6.46E-05	-9.64797
138	0.0475	0.04175	0.0315	4.53E-07	6.45E-05	-9.64812
140	0.049	0.0435	0.03325	4.59E-07	6.45E-05	-9.64822
142	0.0485	0.043	0.03275	4.66E-07	6.45E-05	-9.64831
144	0.0505	0.04475	0.0345	4.81E-07	6.45E-05	-9.64855
146	0.05025	0.0445	0.03425	4.78E-07	6.45E-05	-9.64851
148	0.05075	0.0445	0.03425	5E-07	6.45E-05	-9.64885
150	0.05075	0.04525	0.035	4.94E-07	6.45E-05	-9.64875
152	0.05175	0.04575	0.0355	5.03E-07	6.45E-05	-9.64889
154	0.05325	0.04725	0.037	5.13E-07	6.45E-05	-9.64904
156	0.05375	0.04775	0.0375	5.22E-07	6.45E-05	-9.64918
158	0.05475	0.0485	0.03825	5.22E-07	6.45E-05	-9.64918
160	0.05475	0.048	0.03775	5.25E-07	6.45E-05	-9.64923
162	0.056	0.04925	0.039	5.34E-07	6.45E-05	-9.64938
164	0.05525	0.049	0.03875	5.44E-07	6.45E-05	-9.64952
166	0.0575	0.051	0.04075	5.53E-07	6.44E-05	-9.64967
168	0.0575	0.05025	0.04	5.63E-07	6.44E-05	-9.64981
170	0.058	0.051	0.04075	5.72E-07	6.44E-05	-9.64996
172	0.0585	0.052	0.04175	5.81E-07	6.44E-05	-9.65011
174	0.05925	0.0525	0.04225	5.88E-07	6.44E-05	-9.6502
176	0.0595	0.05275	0.0425	5.94E-07	6.44E-05	-9.6503
178	0.0605	0.05375	0.0435	6.09E-07	6.44E-05	-9.65054
180	0.0625	0.057	0.04675	6.38E-07	6.44E-05	-9.65098
182	0.06225	0.05575	0.0455	6.38E-07	6.44E-05	-9.65098
184	0.06175	0.0555	0.04525	6.28E-07	6.44E-05	-9.65083
186	0.064	0.05775	0.0475	6.56E-07	6.43E-05	-9.65127
188	0.063	0.0565	0.04625	6.47E-07	6.44E-05	-9.65113
190	0.0645	0.05825	0.048	6.72E-07	6.43E-05	-9.65151
192	0.06425	0.0585	0.04825	6.75E-07	6.43E-05	-9.65156
194	0.0655	0.059	0.04875	6.84E-07	6.43E-05	-9.65171
196	0.06625	0.0595	0.04925	6.88E-07	6.43E-05	-9.65176
198	0.071	0.06375	0.0535	6.91E-07	6.43E-05	-9.65181
200	0.0675	0.0605	0.05025	7.03E-07	6.43E-05	-9.652
202	0.06775	0.06125	0.051	7.22E-07	6.43E-05	-9.65229
204	0.06825	0.0615	0.05125	7.19E-07	6.43E-05	-9.65224
206	0.069	0.0625	0.05225	7.31E-07	6.43E-05	-9.65244
208	0.06975	0.063	0.05275	7.41E-07	6.43E-05	-9.65258
210	0.07	0.064	0.05375	7.44E-07	6.43E-05	-9.65263
212	0.07025	0.0635	0.05325	7.53E-07	6.42E-05	-9.65278
214	0.07025	0.06475	0.0545	7.63E-07	6.42E-05	-9.65292
216	0.07225	0.065	0.05475	7.75E-07	6.42E-05	-9.65312
218	0.0725	0.06575	0.0555	7.78E-07	6.42E-05	-9.65317
220	0.07225	0.06575	0.0555	7.94E-07	6.42E-05	-9.65341
222	0.0745	0.06775	0.0575	8E-07	6.42E-05	-9.65351

224	0.0745	0.06775	0.0575	8.09E-07	6.42E-05	-9.65365
226	0.07525	0.068	0.05775	8.13E-07	6.42E-05	-9.6537
228	0.07625	0.06925	0.059	8.28E-07	6.42E-05	-9.65395
230	0.07575	0.06875	0.0585	8.25E-07	6.42E-05	-9.6539
232	0.07575	0.06925	0.059	8.34E-07	6.42E-05	-9.65404
234	0.0775	0.0705	0.06025	8.44E-07	6.42E-05	-9.65419
236	0.07825	0.0705	0.06025	8.5E-07	6.42E-05	-9.65429
238	0.0785	0.0705	0.06025	8.5E-07	6.42E-05	-9.65429
240	0.07925	0.072	0.06175	8.66E-07	6.41E-05	-9.65453
242	0.0805	0.07275	0.0625	8.72E-07	6.41E-05	-9.65463
244	0.08075	0.07275	0.0625	8.94E-07	6.41E-05	-9.65497
246	0.0825	0.07425	0.064	8.91E-07	6.41E-05	-9.65492
248	0.0805	0.07375	0.0635	8.94E-07	6.41E-05	-9.65497
250	0.0835	0.07525	0.065	9.06E-07	6.41E-05	-9.65516
252	0.0835	0.07525	0.065	9.13E-07	6.41E-05	-9.65526
254	0.084	0.076	0.06575	9.19E-07	6.41E-05	-9.65536
256	0.085	0.07725	0.067	9.5E-07	6.41E-05	-9.65585
258	0.0855	0.07825	0.068	9.5E-07	6.41E-05	-9.65585
260	0.086	0.078	0.06775	9.5E-07	6.41E-05	-9.65585
262	0.08625	0.07825	0.068	9.53E-07	6.4E-05	-9.6559
264	0.0875	0.0795	0.06925	9.56E-07	6.4E-05	-9.65594
266	0.0875	0.07925	0.069	9.78E-07	6.4E-05	-9.65629
268	0.0885	0.08	0.06975	9.75E-07	6.4E-05	-9.65624
270	0.08975	0.08175	0.0715	9.91E-07	6.4E-05	-9.65648
272	0.0905	0.0825	0.07225	1.01E-06	6.4E-05	-9.65677
274	0.09125	0.0815	0.07125	9.97E-07	6.4E-05	-9.65658
276	0.092	0.0835	0.07325	1.03E-06	6.4E-05	-9.65702
278	0.09275	0.08425	0.074	1.02E-06	6.4E-05	-9.65697
280	0.09375	0.085	0.07475	1.03E-06	6.4E-05	-9.65712
282	0.09325	0.08475	0.0745	1.04E-06	6.4E-05	-9.65731
284	0.095	0.0865	0.07625	1.05E-06	6.39E-05	-9.65746
286	0.095	0.08675	0.0765	1.07E-06	6.39E-05	-9.65765
288	0.09625	0.0875	0.07725	1.06E-06	6.39E-05	-9.6576
290	0.0965	0.087	0.07675	1.06E-06	6.39E-05	-9.65756
292	0.0975	0.08925	0.079	1.1E-06	6.39E-05	-9.65814
294	0.09825	0.08975	0.0795	1.1E-06	6.39E-05	-9.65819
296	0.0985	0.08825	0.078	1.09E-06	6.39E-05	-9.658
298	0.0995	0.09075	0.0805	1.12E-06	6.39E-05	-9.65848

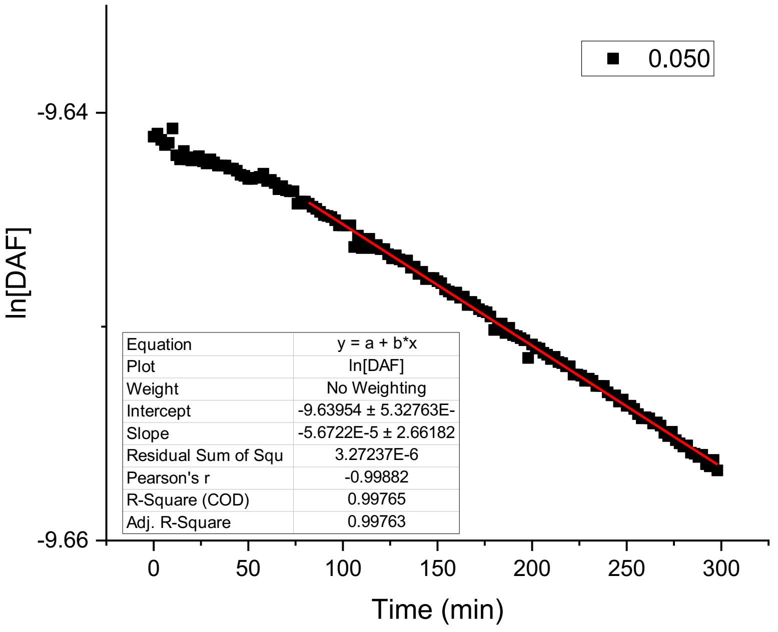

0.065 mM CoHwCl, 50 mM PBS pH 7, [DAF]=0.065 mM

Time (min)	A@490nm	A@490nm minus BK	set to zero	[FLU] (M)	[DAF] (M)	ln[DAF]
0	0.0225	0.01175	0	0	0.000065	-9.64112
2	0.0205	0.01125	-0.0005	-6.3E-09	6.5E-05	-9.64103
4	0.02075	0.01275	0.001	1.25E-08	6.5E-05	-9.64132
6	0.022	0.01425	0.0025	3.13E-08	6.5E-05	-9.6416
8	0.02225	0.0145	0.00275	3.44E-08	6.5E-05	-9.64165
10	0.0215	0.00975	-0.002	-2.5E-08	6.5E-05	-9.64074
12	0.02125	0.0165	0.00475	5.94E-08	6.49E-05	-9.64204
14	0.02225	0.017263	0.005513	6.89E-08	6.49E-05	-9.64218
16	0.021	0.0155	0.00375	4.69E-08	6.5E-05	-9.64184
18	0.02175	0.01725	0.0055	6.88E-08	6.49E-05	-9.64218
20	0.022	0.0185	0.00675	8.44E-08	6.49E-05	-9.64242
22	0.022	0.017	0.00525	6.56E-08	6.49E-05	-9.64213
24	0.022	0.017	0.00525	6.56E-08	6.49E-05	-9.64213
26	0.0225	0.0175	0.00575	7.19E-08	6.49E-05	-9.64223
28	0.02375	0.01825	0.0065	8.13E-08	6.49E-05	-9.64237
30	0.02375	0.01825	0.0065	8.13E-08	6.49E-05	-9.64237
32	0.024	0.01875	0.007	8.75E-08	6.49E-05	-9.64247
34	0.0245	0.0195	0.00775	9.69E-08	6.49E-05	-9.64261
36	0.0245	0.01875	0.007	8.75E-08	6.49E-05	-9.64247
38	0.02475	0.0195	0.00775	9.69E-08	6.49E-05	-9.64261
40	0.025	0.019	0.00725	9.06E-08	6.49E-05	-9.64252
42	0.02575	0.01975	0.008	1E-07	6.49E-05	-9.64266
44	0.02625	0.02075	0.009	1.13E-07	6.49E-05	-9.64286
46	0.02625	0.021	0.00925	1.16E-07	6.49E-05	-9.6429

48	0.02775	0.02175	0.01	1.25E-07	6.49E-05	-9.64305
50	0.02725	0.02225	0.0105	1.31E-07	6.49E-05	-9.64314
52	0.0285	0.0225	0.01075	1.34E-07	6.49E-05	-9.64319
54	0.02825	0.0225	0.01075	1.34E-07	6.49E-05	-9.64319
56	0.02875	0.02275	0.011	1.38E-07	6.49E-05	-9.64324
58	0.0295	0.022	0.01025	1.28E-07	6.49E-05	-9.6431
60	0.03	0.024	0.01225	1.53E-07	6.48E-05	-9.64348
62	0.02975	0.02375	0.012	1.5E-07	6.49E-05	-9.64343
64	0.0305	0.02475	0.013	1.63E-07	6.48E-05	-9.64363
66	0.0305	0.025	0.01325	1.66E-07	6.48E-05	-9.64367
68	0.0315	0.02575	0.014	1.75E-07	6.48E-05	-9.64382
70	0.03275	0.0265	0.01475	1.84E-07	6.48E-05	-9.64396
72	0.03275	0.02725	0.0155	1.94E-07	6.48E-05	-9.64411
74	0.034	0.0275	0.01575	1.97E-07	6.48E-05	-9.64416
76	0.03375	0.02775	0.016	2E-07	6.48E-05	-9.6442
78	0.03475	0.0285	0.01675	2.09E-07	6.48E-05	-9.64435
80	0.03425	0.0285	0.01675	2.09E-07	6.48E-05	-9.64435
82	0.03575	0.0295	0.01775	2.22E-07	6.48E-05	-9.64454
84	0.03625	0.03025	0.0185	2.31E-07	6.48E-05	-9.64469
86	0.03625	0.0305	0.01875	2.34E-07	6.48E-05	-9.64474
88	0.0365	0.03175	0.02	2.5E-07	6.48E-05	-9.64498
90	0.0375	0.032	0.02025	2.53E-07	6.47E-05	-9.64503
92	0.03825	0.03225	0.0205	2.56E-07	6.47E-05	-9.64507
94	0.0395	0.03375	0.022	2.75E-07	6.47E-05	-9.64536
96	0.0395	0.034	0.02225	2.78E-07	6.47E-05	-9.64541
98	0.0405	0.035	0.02325	2.91E-07	6.47E-05	-9.6456
100	0.04075	0.035	0.02325	2.91E-07	6.47E-05	-9.6456
102	0.0415	0.03575	0.024	3E-07	6.47E-05	-9.64575
104	0.04275	0.03625	0.0245	3.06E-07	6.47E-05	-9.64585
106	0.0425	0.03775	0.026	3.25E-07	6.47E-05	-9.64614
108	0.04375	0.03825	0.0265	3.31E-07	6.47E-05	-9.64623
110	0.044	0.03875	0.027	3.38E-07	6.47E-05	-9.64633
112	0.04525	0.039	0.02725	3.41E-07	6.47E-05	-9.64638
114	0.04475	0.039	0.02725	3.41E-07	6.47E-05	-9.64638
116	0.04625	0.042	0.03025	3.78E-07	6.46E-05	-9.64696
118	0.047	0.0415	0.02975	3.72E-07	6.46E-05	-9.64686
120	0.04675	0.0415	0.02975	3.72E-07	6.46E-05	-9.64686
122	0.048	0.0415	0.02975	3.72E-07	6.46E-05	-9.64686
124	0.04925	0.04325	0.0315	3.94E-07	6.46E-05	-9.6472
126	0.049	0.044	0.03225	4.03E-07	6.46E-05	-9.64734
128	0.0485	0.04325	0.0315	3.94E-07	6.46E-05	-9.6472
130	0.05	0.0445	0.03275	4.09E-07	6.46E-05	-9.64744
132	0.0505	0.04575	0.034	4.25E-07	6.46E-05	-9.64768
134	0.0505	0.04525	0.0335	4.19E-07	6.46E-05	-9.64759
136	0.05325	0.04725	0.0355	4.44E-07	6.46E-05	-9.64797
138	0.05375	0.048	0.03625	4.53E-07	6.45E-05	-9.64812

140	0.054	0.0485	0.03675	4.59E-07	6.45E-05	-9.64822
142	0.0545	0.049	0.03725	4.66E-07	6.45E-05	-9.64831
144	0.056	0.05025	0.0385	4.81E-07	6.45E-05	-9.64855
146	0.05575	0.05	0.03825	4.78E-07	6.45E-05	-9.64851
148	0.058	0.05175	0.04	5E-07	6.45E-05	-9.64885
150	0.05675	0.05125	0.0395	4.94E-07	6.45E-05	-9.64875
152	0.058	0.052	0.04025	5.03E-07	6.45E-05	-9.64889
154	0.05875	0.05275	0.041	5.13E-07	6.45E-05	-9.64904
156	0.0595	0.0535	0.04175	5.22E-07	6.45E-05	-9.64918
158	0.05975	0.0535	0.04175	5.22E-07	6.45E-05	-9.64918
160	0.0605	0.05375	0.042	5.25E-07	6.45E-05	-9.64923
162	0.06125	0.0545	0.04275	5.34E-07	6.45E-05	-9.64938
164	0.0615	0.05525	0.0435	5.44E-07	6.45E-05	-9.64952
166	0.0625	0.056	0.04425	5.53E-07	6.44E-05	-9.64967
168	0.064	0.05675	0.045	5.63E-07	6.44E-05	-9.64981
170	0.0645	0.0575	0.04575	5.72E-07	6.44E-05	-9.64996
172	0.06475	0.05825	0.0465	5.81E-07	6.44E-05	-9.65011
174	0.0655	0.05875	0.047	5.88E-07	6.44E-05	-9.6502
176	0.066	0.05925	0.0475	5.94E-07	6.44E-05	-9.6503
178	0.06725	0.0605	0.04875	6.09E-07	6.44E-05	-9.65054
180	0.06825	0.06275	0.051	6.38E-07	6.44E-05	-9.65098
182	0.06925	0.06275	0.051	6.38E-07	6.44E-05	-9.65098
184	0.06825	0.062	0.05025	6.28E-07	6.44E-05	-9.65083
186	0.0705	0.06425	0.0525	6.56E-07	6.43E-05	-9.65127
188	0.07	0.0635	0.05175	6.47E-07	6.44E-05	-9.65113
190	0.07175	0.0655	0.05375	6.72E-07	6.43E-05	-9.65151
192	0.0715	0.06575	0.054	6.75E-07	6.43E-05	-9.65156
194	0.073	0.0665	0.05475	6.84E-07	6.43E-05	-9.65171
196	0.0735	0.06675	0.055	6.88E-07	6.43E-05	-9.65176
198	0.07425	0.067	0.05525	6.91E-07	6.43E-05	-9.65181
200	0.075	0.068	0.05625	7.03E-07	6.43E-05	-9.652
202	0.076	0.0695	0.05775	7.22E-07	6.43E-05	-9.65229
204	0.076	0.06925	0.0575	7.19E-07	6.43E-05	-9.65224
206	0.07675	0.07025	0.0585	7.31E-07	6.43E-05	-9.65244
208	0.07775	0.071	0.05925	7.41E-07	6.43E-05	-9.65258
210	0.07725	0.07125	0.0595	7.44E-07	6.43E-05	-9.65263
212	0.07875	0.072	0.06025	7.53E-07	6.42E-05	-9.65278
214	0.07825	0.07275	0.061	7.63E-07	6.42E-05	-9.65292
216	0.081	0.07375	0.062	7.75E-07	6.42E-05	-9.65312
218	0.08075	0.074	0.06225	7.78E-07	6.42E-05	-9.65317
220	0.08175	0.07525	0.0635	7.94E-07	6.42E-05	-9.65341
222	0.0825	0.07575	0.064	8E-07	6.42E-05	-9.65351
224	0.08325	0.0765	0.06475	8.09E-07	6.42E-05	-9.65365
226	0.084	0.07675	0.065	8.13E-07	6.42E-05	-9.6537
228	0.085	0.078	0.06625	8.28E-07	6.42E-05	-9.65395
230	0.08475	0.07775	0.066	8.25E-07	6.42E-05	-9.6539

232	0.085	0.0785	0.06675	8.34E-07	6.42E-05	-9.65404
234	0.08625	0.07925	0.0675	8.44E-07	6.42E-05	-9.65419
236	0.0875	0.07975	0.068	8.5E-07	6.42E-05	-9.65429
238	0.08775	0.07975	0.068	8.5E-07	6.42E-05	-9.65429
240	0.08825	0.081	0.06925	8.66E-07	6.41E-05	-9.65453
242	0.08925	0.0815	0.06975	8.72E-07	6.41E-05	-9.65463
244	0.09125	0.08325	0.0715	8.94E-07	6.41E-05	-9.65497
246	0.09125	0.083	0.07125	8.91E-07	6.41E-05	-9.65492
248	0.09	0.08325	0.0715	8.94E-07	6.41E-05	-9.65497
250	0.0925	0.08425	0.0725	9.06E-07	6.41E-05	-9.65516
252	0.093	0.08475	0.073	9.13E-07	6.41E-05	-9.65526
254	0.09325	0.08525	0.0735	9.19E-07	6.41E-05	-9.65536
256	0.0955	0.08775	0.076	9.5E-07	6.41E-05	-9.65585
258	0.095	0.08775	0.076	9.5E-07	6.41E-05	-9.65585
260	0.09575	0.08775	0.076	9.5E-07	6.41E-05	-9.65585
262	0.096	0.088	0.07625	9.53E-07	6.4E-05	-9.6559
264	0.09625	0.08825	0.0765	9.56E-07	6.4E-05	-9.65594
266	0.09825	0.09	0.07825	9.78E-07	6.4E-05	-9.65629
268	0.09825	0.08975	0.078	9.75E-07	6.4E-05	-9.65624
270	0.099	0.091	0.07925	9.91E-07	6.4E-05	-9.65648
272	0.1005	0.0925	0.08075	1.01E-06	6.4E-05	-9.65677
274	0.10125	0.0915	0.07975	9.97E-07	6.4E-05	-9.65658
276	0.10225	0.09375	0.082	1.03E-06	6.4E-05	-9.65702
278	0.102	0.0935	0.08175	1.02E-06	6.4E-05	-9.65697
280	0.103	0.09425	0.0825	1.03E-06	6.4E-05	-9.65712
282	0.10375	0.09525	0.0835	1.04E-06	6.4E-05	-9.65731
284	0.1045	0.096	0.08425	1.05E-06	6.39E-05	-9.65746
286	0.10525	0.097	0.08525	1.07E-06	6.39E-05	-9.65765
288	0.1055	0.09675	0.085	1.06E-06	6.39E-05	-9.6576
290	0.106	0.0965	0.08475	1.06E-06	6.39E-05	-9.65756
292	0.10775	0.0995	0.08775	1.1E-06	6.39E-05	-9.65814
294	0.10825	0.09975	0.088	1.1E-06	6.39E-05	-9.65819
296	0.109	0.09875	0.087	1.09E-06	6.39E-05	-9.658
298	0.11	0.10125	0.0895	1.12E-06	6.39E-05	-9.65848

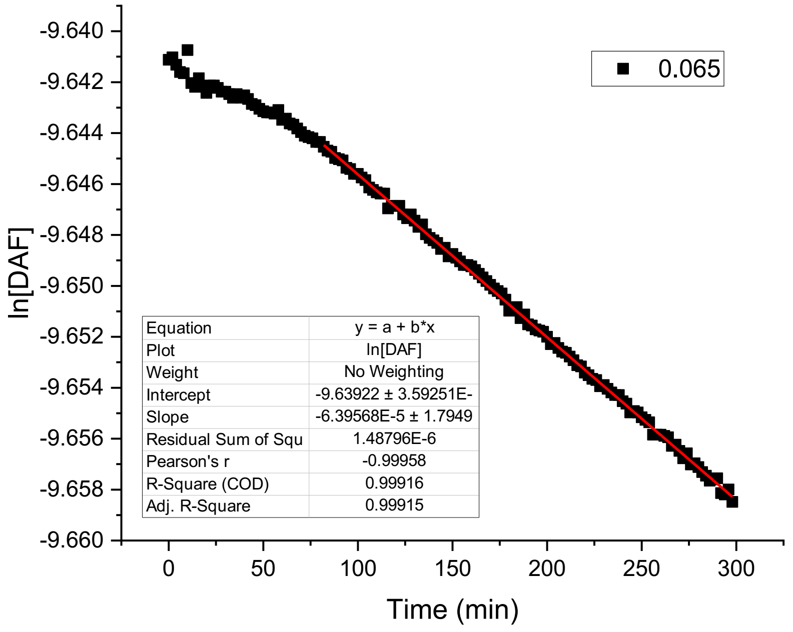

0.100 mM

CoHwCl, 50 mM PBS pH 7, [DAF]=0.065 mM

Time (min)	A@490nm	A@490nm minus BK	set to zero	[FLU] (M)	[DAF] (M)	ln[DAF]
0	0.02875	0.018	0	0	0.000065	-9.64112
2	0.029	0.01975	0.00175	2.19E-08	6.5E-05	-9.64146
4	0.029	0.021	0.003	3.75E-08	6.5E-05	-9.6417
6	0.029	0.02125	0.00325	4.06E-08	6.5E-05	-9.64175
8	0.02825	0.0205	0.0025	3.13E-08	6.5E-05	-9.6416
10	0.0285	0.01675	-0.00125	-1.6E-08	6.5E-05	-9.64088
12	0.02875	0.024	0.006	7.5E-08	6.49E-05	-9.64228
14	0.02925	0.024263	0.006263	7.83E-08	6.49E-05	-9.64233
16	0.0285	0.023	0.005	6.25E-08	6.49E-05	-9.64209
18	0.02875	0.02425	0.00625	7.81E-08	6.49E-05	-9.64233
20	0.03	0.0265	0.0085	1.06E-07	6.49E-05	-9.64276
22	0.0295	0.0245	0.0065	8.13E-08	6.49E-05	-9.64237
24	0.0305	0.0255	0.0075	9.38E-08	6.49E-05	-9.64257
26	0.0305	0.0255	0.0075	9.38E-08	6.49E-05	-9.64257
28	0.031	0.0255	0.0075	9.38E-08	6.49E-05	-9.64257
30	0.03125	0.02575	0.00775	9.69E-08	6.49E-05	-9.64261
32	0.032	0.02675	0.00875	1.09E-07	6.49E-05	-9.64281
34	0.033	0.028	0.01	1.25E-07	6.49E-05	-9.64305
36	0.034	0.02825	0.01025	1.28E-07	6.49E-05	-9.6431
38	0.03325	0.028	0.01	1.25E-07	6.49E-05	-9.64305
40	0.035	0.029	0.011	1.38E-07	6.49E-05	-9.64324
42	0.03525	0.02925	0.01125	1.41E-07	6.49E-05	-9.64329
44	0.03675	0.03125	0.01325	1.66E-07	6.48E-05	-9.64367
46	0.03775	0.0325	0.0145	1.81E-07	6.48E-05	-9.64392
48	0.03925	0.03325	0.01525	1.91E-07	6.48E-05	-9.64406

50	0.03875	0.03375	0.01575	1.97E-07	6.48E-05	-9.64416
52	0.04025	0.03425	0.01625	2.03E-07	6.48E-05	-9.64425
54	0.03975	0.034	0.016	2E-07	6.48E-05	-9.6442
56	0.03975	0.03375	0.01575	1.97E-07	6.48E-05	-9.64416
58	0.0415	0.034	0.016	2E-07	6.48E-05	-9.6442
60	0.0415	0.0355	0.0175	2.19E-07	6.48E-05	-9.64449
62	0.04175	0.03575	0.01775	2.22E-07	6.48E-05	-9.64454
64	0.04225	0.0365	0.0185	2.31E-07	6.48E-05	-9.64469
66	0.04325	0.03775	0.01975	2.47E-07	6.48E-05	-9.64493
68	0.0435	0.03775	0.01975	2.47E-07	6.48E-05	-9.64493
70	0.0455	0.03925	0.02125	2.66E-07	6.47E-05	-9.64522
72	0.04525	0.03975	0.02175	2.72E-07	6.47E-05	-9.64531
74	0.04675	0.04025	0.02225	2.78E-07	6.47E-05	-9.64541
76	0.04725	0.04125	0.02325	2.91E-07	6.47E-05	-9.6456
78	0.05	0.04375	0.02575	3.22E-07	6.47E-05	-9.64609
80	0.04825	0.0425	0.0245	3.06E-07	6.47E-05	-9.64585
82	0.04925	0.043	0.025	3.13E-07	6.47E-05	-9.64594
84	0.0505	0.0445	0.0265	3.31E-07	6.47E-05	-9.64623
86	0.0505	0.04475	0.02675	3.34E-07	6.47E-05	-9.64628
88	0.051	0.04625	0.02825	3.53E-07	6.46E-05	-9.64657
90	0.05275	0.04725	0.02925	3.66E-07	6.46E-05	-9.64676
92	0.0535	0.0475	0.0295	3.69E-07	6.46E-05	-9.64681
94	0.05425	0.0485	0.0305	3.81E-07	6.46E-05	-9.64701
96	0.0545	0.049	0.031	3.88E-07	6.46E-05	-9.6471
98	0.05475	0.04925	0.03125	3.91E-07	6.46E-05	-9.64715
100	0.056	0.05025	0.03225	4.03E-07	6.46E-05	-9.64734
102	0.057	0.05125	0.03325	4.16E-07	6.46E-05	-9.64754
104	0.058	0.0515	0.0335	4.19E-07	6.46E-05	-9.64759
106	0.05825	0.0535	0.0355	4.44E-07	6.46E-05	-9.64797
108	0.05875	0.05325	0.03525	4.41E-07	6.46E-05	-9.64793
110	0.0605	0.05525	0.03725	4.66E-07	6.45E-05	-9.64831
112	0.061	0.05475	0.03675	4.59E-07	6.45E-05	-9.64822
114	0.06125	0.0555	0.0375	4.69E-07	6.45E-05	-9.64836
116	0.0625	0.05825	0.04025	5.03E-07	6.45E-05	-9.64889
118	0.06325	0.05775	0.03975	4.97E-07	6.45E-05	-9.6488
120	0.0635	0.05825	0.04025	5.03E-07	6.45E-05	-9.64889
122	0.065	0.0585	0.0405	5.06E-07	6.45E-05	-9.64894
124	0.067	0.061	0.043	5.38E-07	6.45E-05	-9.64943
126	0.06675	0.06175	0.04375	5.47E-07	6.45E-05	-9.64957
128	0.0665	0.06125	0.04325	5.41E-07	6.45E-05	-9.64948
130	0.06725	0.06175	0.04375	5.47E-07	6.45E-05	-9.64957
132	0.068	0.06325	0.04525	5.66E-07	6.44E-05	-9.64986
134	0.069	0.06375	0.04575	5.72E-07	6.44E-05	-9.64996
136	0.0715	0.0655	0.0475	5.94E-07	6.44E-05	-9.6503
138	0.07175	0.066	0.048	6E-07	6.44E-05	-9.6504
140	0.0725	0.067	0.049	6.13E-07	6.44E-05	-9.65059

142	0.0725	0.067	0.049	6.13E-07	6.44E-05	-9.65059
144	0.075	0.06925	0.05125	6.41E-07	6.44E-05	-9.65103
146	0.07525	0.0695	0.0515	6.44E-07	6.44E-05	-9.65108
148	0.0765	0.07025	0.05225	6.53E-07	6.43E-05	-9.65122
150	0.07675	0.07125	0.05325	6.66E-07	6.43E-05	-9.65142
152	0.0775	0.0715	0.0535	6.69E-07	6.43E-05	-9.65147
154	0.079	0.073	0.055	6.88E-07	6.43E-05	-9.65176
156	0.07975	0.07375	0.05575	6.97E-07	6.43E-05	-9.6519
158	0.0805	0.07425	0.05625	7.03E-07	6.43E-05	-9.652
160	0.08125	0.0745	0.0565	7.06E-07	6.43E-05	-9.65205
162	0.08225	0.0755	0.0575	7.19E-07	6.43E-05	-9.65224
164	0.08275	0.0765	0.0585	7.31E-07	6.43E-05	-9.65244
166	0.0835	0.077	0.059	7.38E-07	6.43E-05	-9.65253
168	0.08525	0.078	0.06	7.5E-07	6.43E-05	-9.65273
170	0.08625	0.07925	0.06125	7.66E-07	6.42E-05	-9.65297
172	0.0865	0.08	0.062	7.75E-07	6.42E-05	-9.65312
174	0.088	0.08125	0.06325	7.91E-07	6.42E-05	-9.65336
176	0.08825	0.0815	0.0635	7.94E-07	6.42E-05	-9.65341
178	0.09	0.08325	0.06525	8.16E-07	6.42E-05	-9.65375
180	0.091	0.0855	0.0675	8.44E-07	6.42E-05	-9.65419
182	0.092	0.0855	0.0675	8.44E-07	6.42E-05	-9.65419
184	0.0915	0.08525	0.06725	8.41E-07	6.42E-05	-9.65414
186	0.094	0.08775	0.06975	8.72E-07	6.41E-05	-9.65463
188	0.09325	0.08675	0.06875	8.59E-07	6.41E-05	-9.65443
190	0.09475	0.0885	0.0705	8.81E-07	6.41E-05	-9.65477
192	0.096	0.09025	0.07225	9.03E-07	6.41E-05	-9.65511
194	0.09675	0.09025	0.07225	9.03E-07	6.41E-05	-9.65511
196	0.0975	0.09075	0.07275	9.09E-07	6.41E-05	-9.65521
198	0.09925	0.092	0.074	9.25E-07	6.41E-05	-9.65546
200	0.09975	0.09275	0.07475	9.34E-07	6.41E-05	-9.6556
202	0.1005	0.094	0.076	9.5E-07	6.41E-05	-9.65585
204	0.101	0.09425	0.07625	9.53E-07	6.4E-05	-9.6559
206	0.1015	0.095	0.077	9.63E-07	6.4E-05	-9.65604
208	0.10375	0.097	0.079	9.88E-07	6.4E-05	-9.65643
210	0.103	0.097	0.079	9.88E-07	6.4E-05	-9.65643
212	0.105	0.09825	0.08025	1E-06	6.4E-05	-9.65668
214	0.10425	0.09875	0.08075	1.01E-06	6.4E-05	-9.65677
216	0.106	0.09875	0.08075	1.01E-06	6.4E-05	-9.65677
218	0.1065	0.09975	0.08175	1.02E-06	6.4E-05	-9.65697
220	0.10725	0.10075	0.08275	1.03E-06	6.4E-05	-9.65716
222	0.10875	0.102	0.084	1.05E-06	6.4E-05	-9.65741
224	0.10975	0.103	0.085	1.06E-06	6.39E-05	-9.6576
226	0.1105	0.10325	0.08525	1.07E-06	6.39E-05	-9.65765
228	0.1115	0.1045	0.0865	1.08E-06	6.39E-05	-9.6579
230	0.1115	0.1045	0.0865	1.08E-06	6.39E-05	-9.6579
232	0.11125	0.10475	0.08675	1.08E-06	6.39E-05	-9.65795

234	0.11375	0.10675	0.08875	1.11E-06	6.39E-05	-9.65834
236	0.11425	0.1065	0.0885	1.11E-06	6.39E-05	-9.65829
238	0.11475	0.10675	0.08875	1.11E-06	6.39E-05	-9.65834
240	0.116	0.10875	0.09075	1.13E-06	6.39E-05	-9.65873
242	0.11675	0.109	0.091	1.14E-06	6.39E-05	-9.65878
244	0.11825	0.11025	0.09225	1.15E-06	6.38E-05	-9.65902
246	0.11875	0.1105	0.0925	1.16E-06	6.38E-05	-9.65907
248	0.118	0.11125	0.09325	1.17E-06	6.38E-05	-9.65922
250	0.12025	0.112	0.094	1.18E-06	6.38E-05	-9.65937
252	0.12125	0.113	0.095	1.19E-06	6.38E-05	-9.65956
254	0.12125	0.11325	0.09525	1.19E-06	6.38E-05	-9.65961
256	0.1235	0.11575	0.09775	1.22E-06	6.38E-05	-9.6601
258	0.12375	0.1165	0.0985	1.23E-06	6.38E-05	-9.66025
260	0.12475	0.11675	0.09875	1.23E-06	6.38E-05	-9.6603
262	0.1255	0.1175	0.0995	1.24E-06	6.38E-05	-9.66044
264	0.12625	0.11825	0.10025	1.25E-06	6.37E-05	-9.66059
266	0.127	0.11875	0.10075	1.26E-06	6.37E-05	-9.66069
268	0.12775	0.11925	0.10125	1.27E-06	6.37E-05	-9.66079
270	0.1295	0.1215	0.1035	1.29E-06	6.37E-05	-9.66123
272	0.12975	0.12175	0.10375	1.3E-06	6.37E-05	-9.66128
274	0.1305	0.12075	0.10275	1.28E-06	6.37E-05	-9.66108
276	0.132	0.1235	0.1055	1.32E-06	6.37E-05	-9.66162
278	0.13175	0.12325	0.10525	1.32E-06	6.37E-05	-9.66157
280	0.13375	0.125	0.107	1.34E-06	6.37E-05	-9.66191
282	0.1345	0.126	0.108	1.35E-06	6.37E-05	-9.66211
284	0.135	0.1265	0.1085	1.36E-06	6.36E-05	-9.66221
286	0.13625	0.128	0.11	1.38E-06	6.36E-05	-9.6625
288	0.1365	0.12775	0.10975	1.37E-06	6.36E-05	-9.66245
290	0.13725	0.12775	0.10975	1.37E-06	6.36E-05	-9.66245
292	0.139	0.13075	0.11275	1.41E-06	6.36E-05	-9.66304
294	0.139	0.1305	0.1125	1.41E-06	6.36E-05	-9.663
296	0.14025	0.13	0.112	1.4E-06	6.36E-05	-9.6629
298	0.1415	0.13275	0.11475	1.43E-06	6.36E-05	-9.66344

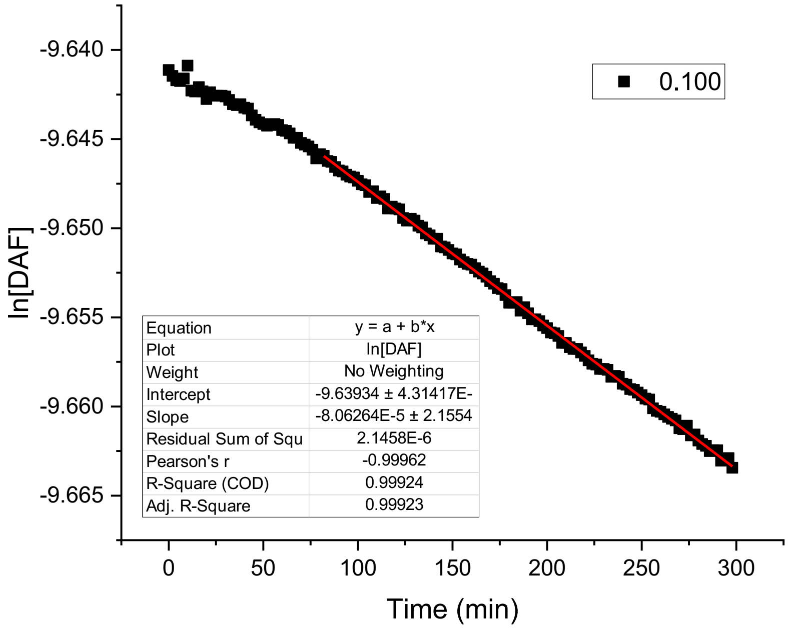

0.125 mM CoHwCl, 50 mM PBS pH 7, [DAF]=0.065 mM

Time (min)	A@490nm	A@490nm minus BK	set to zero	[FLU] (M)	[DAF] (M)	ln[DAF]
0 min	0.031	0.02025	0	0	0.000065	-9.64112
2 min	0.03025	0.021	0.00075	9.37E-09	6.5E-05	-9.64127
4 min	0.02975	0.02175	0.0015	1.88E-08	6.5E-05	-9.64141
6 min	0.0315	0.02375	0.0035	4.38E-08	6.5E-05	-9.6418
8 min	0.03125	0.0235	0.00325	4.06E-08	6.5E-05	-9.64175
10 min	0.03075	0.019	-0.00125	-1.6E-08	6.5E-05	-9.64088
12 min	0.0305	0.02575	0.0055	6.88E-08	6.49E-05	-9.64218
14 min	0.03175	0.026763	0.006513	8.14E-08	6.49E-05	-9.64238
16 min	0.03175	0.02625	0.006	7.5E-08	6.49E-05	-9.64228
18 min	0.03175	0.02725	0.007	8.75E-08	6.49E-05	-9.64247
20 min	0.033	0.0295	0.00925	1.16E-07	6.49E-05	-9.6429
22 min	0.03275	0.02775	0.0075	9.38E-08	6.49E-05	-9.64257
24 min	0.03375	0.02875	0.0085	1.06E-07	6.49E-05	-9.64276
26 min	0.03425	0.02925	0.009	1.13E-07	6.49E-05	-9.64286
28 min	0.03525	0.02975	0.0095	1.19E-07	6.49E-05	-9.64295
30 min	0.03525	0.02975	0.0095	1.19E-07	6.49E-05	-9.64295
32 min	0.0355	0.03025	0.01	1.25E-07	6.49E-05	-9.64305
34 min	0.0365	0.0315	0.01125	1.41E-07	6.49E-05	-9.64329
36 min	0.0375	0.03175	0.0115	1.44E-07	6.49E-05	-9.64334
38 min	0.0375	0.03225	0.012	1.5E-07	6.49E-05	-9.64343
40 min	0.0385	0.0325	0.01225	1.53E-07	6.48E-05	-9.64348
42 min	0.039	0.033	0.01275	1.59E-07	6.48E-05	-9.64358
44 min	0.04	0.0345	0.01425	1.78E-07	6.48E-05	-9.64387

46 min	0.04	0.03475	0.0145	1.81E-07	6.48E-05	-9.64392
48 min	0.04175	0.03575	0.0155	1.94E-07	6.48E-05	-9.64411
50 min	0.0415	0.0365	0.01625	2.03E-07	6.48E-05	-9.64425
52 min	0.0435	0.0375	0.01725	2.16E-07	6.48E-05	-9.64445
54 min	0.0435	0.03775	0.0175	2.19E-07	6.48E-05	-9.64449
56 min	0.04425	0.03825	0.018	2.25E-07	6.48E-05	-9.64459
58 min	0.04525	0.03775	0.0175	2.19E-07	6.48E-05	-9.64449
60 min	0.046	0.04	0.01975	2.47E-07	6.48E-05	-9.64493
62 min	0.04625	0.04025	0.02	2.5E-07	6.48E-05	-9.64498
64 min	0.04675	0.041	0.02075	2.59E-07	6.47E-05	-9.64512
66 min	0.04775	0.04225	0.022	2.75E-07	6.47E-05	-9.64536
68 min	0.0485	0.04275	0.0225	2.81E-07	6.47E-05	-9.64546
70 min	0.0505	0.04425	0.024	3E-07	6.47E-05	-9.64575
72 min	0.051	0.0455	0.02525	3.16E-07	6.47E-05	-9.64599
74 min	0.0525	0.046	0.02575	3.22E-07	6.47E-05	-9.64609
76 min	0.0525	0.0465	0.02625	3.28E-07	6.47E-05	-9.64618
78 min	0.05375	0.0475	0.02725	3.41E-07	6.47E-05	-9.64638
80 min	0.055	0.04925	0.029	3.63E-07	6.46E-05	-9.64672
82 min	0.056	0.04975	0.0295	3.69E-07	6.46E-05	-9.64681
84 min	0.057	0.051	0.03075	3.84E-07	6.46E-05	-9.64705
86 min	0.05775	0.052	0.03175	3.97E-07	6.46E-05	-9.64725
88 min	0.05825	0.0535	0.03325	4.16E-07	6.46E-05	-9.64754
90 min	0.06025	0.05475	0.0345	4.31E-07	6.46E-05	-9.64778
92 min	0.061	0.055	0.03475	4.34E-07	6.46E-05	-9.64783
94 min	0.06225	0.0565	0.03625	4.53E-07	6.45E-05	-9.64812
96 min	0.06325	0.05775	0.0375	4.69E-07	6.45E-05	-9.64836
98 min	0.0645	0.059	0.03875	4.84E-07	6.45E-05	-9.6486
100 min	0.0655	0.05975	0.0395	4.94E-07	6.45E-05	-9.64875
102 min	0.066	0.06025	0.04	5E-07	6.45E-05	-9.64885
104 min	0.068	0.0615	0.04125	5.16E-07	6.45E-05	-9.64909
106 min	0.06775	0.063	0.04275	5.34E-07	6.45E-05	-9.64938
108 min	0.07	0.0645	0.04425	5.53E-07	6.44E-05	-9.64967
110 min	0.07075	0.0655	0.04525	5.66E-07	6.44E-05	-9.64986
112 min	0.0725	0.06625	0.046	5.75E-07	6.44E-05	-9.65001
114 min	0.0725	0.06675	0.0465	5.81E-07	6.44E-05	-9.65011
116 min	0.0735	0.06925	0.049	6.13E-07	6.44E-05	-9.65059
118 min	0.07375	0.06825	0.048	6E-07	6.44E-05	-9.6504
120 min	0.07525	0.07	0.04975	6.22E-07	6.44E-05	-9.65074
122 min	0.077	0.0705	0.05025	6.28E-07	6.44E-05	-9.65083
124 min	0.0785	0.0725	0.05225	6.53E-07	6.43E-05	-9.65122
126 min	0.079	0.074	0.05375	6.72E-07	6.43E-05	-9.65151
128 min	0.07875	0.0735	0.05325	6.66E-07	6.43E-05	-9.65142
130 min	0.08075	0.07525	0.055	6.88E-07	6.43E-05	-9.65176
132 min	0.081	0.07625	0.056	7E-07	6.43E-05	-9.65195
134 min	0.08225	0.077	0.05675	7.09E-07	6.43E-05	-9.6521
136 min	0.08525	0.07925	0.059	7.38E-07	6.43E-05	-9.65253

138 min	0.0855	0.07975	0.0595	7.44E-07	6.43E-05	-9.65263
140 min	0.08675	0.08125	0.061	7.63E-07	6.42E-05	-9.65292
142 min	0.08675	0.08125	0.061	7.63E-07	6.42E-05	-9.65292
144 min	0.09	0.08425	0.064	8E-07	6.42E-05	-9.65351
146 min	0.08975	0.084	0.06375	7.97E-07	6.42E-05	-9.65346
148 min	0.091	0.08475	0.0645	8.06E-07	6.42E-05	-9.6536
150 min	0.0915	0.086	0.06575	8.22E-07	6.42E-05	-9.65385
152 min	0.09275	0.08675	0.0665	8.31E-07	6.42E-05	-9.65399
154 min	0.0945	0.0885	0.06825	8.53E-07	6.41E-05	-9.65434
156 min	0.096	0.09	0.06975	8.72E-07	6.41E-05	-9.65463
158 min	0.09625	0.09	0.06975	8.72E-07	6.41E-05	-9.65463
160 min	0.09725	0.0905	0.07025	8.78E-07	6.41E-05	-9.65472
162 min	0.0985	0.09175	0.0715	8.94E-07	6.41E-05	-9.65497
164 min	0.09875	0.0925	0.07225	9.03E-07	6.41E-05	-9.65511
166 min	0.10075	0.09425	0.074	9.25E-07	6.41E-05	-9.65546
168 min	0.1025	0.09525	0.075	9.38E-07	6.41E-05	-9.65565
170 min	0.10325	0.09625	0.076	9.5E-07	6.41E-05	-9.65585
172 min	0.1045	0.098	0.07775	9.72E-07	6.4E-05	-9.65619
174 min	0.1055	0.09875	0.0785	9.81E-07	6.4E-05	-9.65633
176 min	0.10625	0.0995	0.07925	9.91E-07	6.4E-05	-9.65648
178 min	0.10725	0.1005	0.08025	1E-06	6.4E-05	-9.65668
180 min	0.10925	0.10375	0.0835	1.04E-06	6.4E-05	-9.65731
182 min	0.11025	0.10375	0.0835	1.04E-06	6.4E-05	-9.65731
184 min	0.1105	0.10425	0.084	1.05E-06	6.4E-05	-9.65741
186 min	0.1125	0.10625	0.086	1.08E-06	6.39E-05	-9.6578
188 min	0.113	0.1065	0.08625	1.08E-06	6.39E-05	-9.65785
190 min	0.114	0.10775	0.0875	1.09E-06	6.39E-05	-9.65809
192 min	0.11575	0.11	0.08975	1.12E-06	6.39E-05	-9.65853
194 min	0.11675	0.11025	0.09	1.13E-06	6.39E-05	-9.65858
196 min	0.118	0.11125	0.091	1.14E-06	6.39E-05	-9.65878
198 min	0.119	0.11175	0.0915	1.14E-06	6.39E-05	-9.65888
200 min	0.12025	0.11325	0.093	1.16E-06	6.38E-05	-9.65917
202 min	0.12075	0.11425	0.094	1.18E-06	6.38E-05	-9.65937
204 min	0.1215	0.11475	0.0945	1.18E-06	6.38E-05	-9.65946
206 min	0.123	0.1165	0.09625	1.2E-06	6.38E-05	-9.65981
208 min	0.125	0.11825	0.098	1.23E-06	6.38E-05	-9.66015
210 min	0.125	0.119	0.09875	1.23E-06	6.38E-05	-9.6603
212 min	0.12675	0.12	0.09975	1.25E-06	6.38E-05	-9.66049
214 min	0.12675	0.12125	0.101	1.26E-06	6.37E-05	-9.66074
216 min	0.129	0.12175	0.1015	1.27E-06	6.37E-05	-9.66084
218 min	0.129	0.12225	0.102	1.28E-06	6.37E-05	-9.66093
220 min	0.1305	0.124	0.10375	1.3E-06	6.37E-05	-9.66128
222 min	0.13175	0.125	0.10475	1.31E-06	6.37E-05	-9.66147
224 min	0.133	0.12625	0.106	1.33E-06	6.37E-05	-9.66172
226 min	0.13475	0.1275	0.10725	1.34E-06	6.37E-05	-9.66196
228 min	0.13525	0.12825	0.108	1.35E-06	6.37E-05	-9.66211

230 min	0.135	0.128	0.10775	1.35E-06	6.37E-05	-9.66206
232 min	0.13575	0.12925	0.109	1.36E-06	6.36E-05	-9.66231
234 min	0.13725	0.13025	0.11	1.38E-06	6.36E-05	-9.6625
236 min	0.139	0.13125	0.111	1.39E-06	6.36E-05	-9.6627
238 min	0.14	0.132	0.11175	1.4E-06	6.36E-05	-9.66285
240 min	0.141	0.13375	0.1135	1.42E-06	6.36E-05	-9.66319
242 min	0.14225	0.1345	0.11425	1.43E-06	6.36E-05	-9.66334
244 min	0.14375	0.13575	0.1155	1.44E-06	6.36E-05	-9.66359
246 min	0.14525	0.137	0.11675	1.46E-06	6.35E-05	-9.66383
248 min	0.144	0.13725	0.117	1.46E-06	6.35E-05	-9.66388
250 min	0.14675	0.1385	0.11825	1.48E-06	6.35E-05	-9.66413
252 min	0.1475	0.13925	0.119	1.49E-06	6.35E-05	-9.66427
254 min	0.148	0.14	0.11975	1.5E-06	6.35E-05	-9.66442
256 min	0.149	0.14125	0.121	1.51E-06	6.35E-05	-9.66467
258 min	0.151	0.14375	0.1235	1.54E-06	6.35E-05	-9.66516
260 min	0.15125	0.14325	0.123	1.54E-06	6.35E-05	-9.66506
262 min	0.15175	0.14375	0.1235	1.54E-06	6.35E-05	-9.66516
264 min	0.15325	0.14525	0.125	1.56E-06	6.34E-05	-9.66546
266 min	0.1545	0.14625	0.126	1.58E-06	6.34E-05	-9.66565
268 min	0.15475	0.14625	0.126	1.58E-06	6.34E-05	-9.66565
270 min	0.15675	0.14875	0.1285	1.61E-06	6.34E-05	-9.66615
272 min	0.158	0.15	0.12975	1.62E-06	6.34E-05	-9.66639
274 min	0.15875	0.149	0.12875	1.61E-06	6.34E-05	-9.66619
276 min	0.16075	0.15225	0.132	1.65E-06	6.34E-05	-9.66684
278 min	0.1605	0.152	0.13175	1.65E-06	6.34E-05	-9.66679
280 min	0.16225	0.1535	0.13325	1.67E-06	6.33E-05	-9.66708
282 min	0.1635	0.155	0.13475	1.68E-06	6.33E-05	-9.66738
284 min	0.165	0.1565	0.13625	1.7E-06	6.33E-05	-9.66767
286 min	0.166	0.15775	0.1375	1.72E-06	6.33E-05	-9.66792
288 min	0.1665	0.15775	0.1375	1.72E-06	6.33E-05	-9.66792
290 min	0.167	0.1575	0.13725	1.72E-06	6.33E-05	-9.66787
292 min	0.168	0.15975	0.1395	1.74E-06	6.33E-05	-9.66832
294 min	0.169	0.1605	0.14025	1.75E-06	6.32E-05	-9.66846
296 min	0.1695	0.15925	0.139	1.74E-06	6.33E-05	-9.66822
298 min	0.17075	0.162	0.14175	1.77E-06	6.32E-05	-9.66876

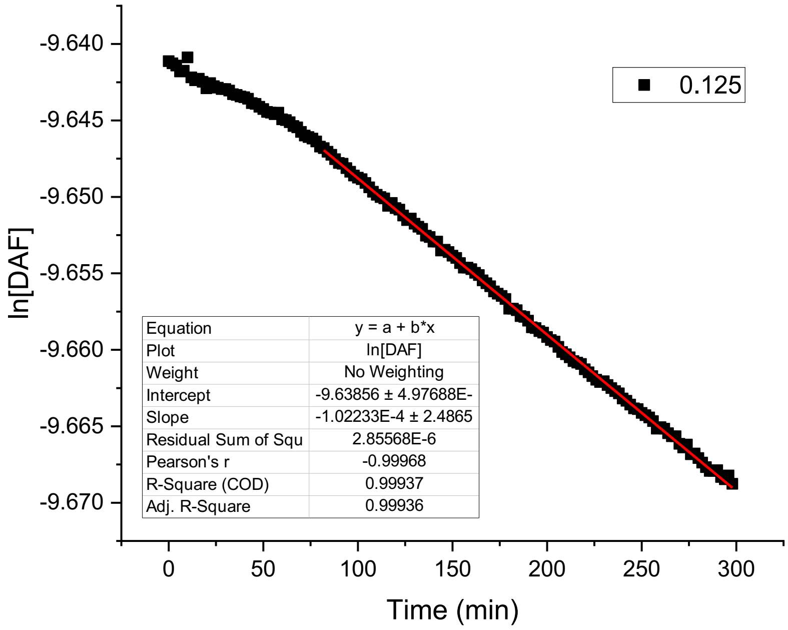

0.150 mM CoHwCl, 50 mM PBS pH 7, [DAF]=0.065 mM

Time (min)	A@490nm	A@490nm minus BK	set to zero	[FLU] (M)	[DAF] (M)	ln[DAF]
0 min	0.0315	0.02075	0	0	0.000065	-9.64112
2 min	0.03075	0.0215	0.00075	9.37E-09	6.5E-05	-9.64127
4 min	0.02975	0.02175	0.001	1.25E-08	6.5E-05	-9.64132
6 min	0.0325	0.02475	0.004	5E-08	6.5E-05	-9.64189
8 min	0.0315	0.02375	0.003	3.75E-08	6.5E-05	-9.6417
10 min	0.032	0.02025	-0.0005	-6.3E-09	6.5E-05	-9.64103
12 min	0.03225	0.0275	0.00675	8.44E-08	6.49E-05	-9.64242
14 min	0.03275	0.0277625	0.007013	8.77E-08	6.49E-05	-9.64247
16 min	0.032	0.0265	0.00575	7.19E-08	6.49E-05	-9.64223
18 min	0.03275	0.02825	0.0075	9.38E-08	6.49E-05	-9.64257
20 min	0.0335	0.03	0.00925	1.16E-07	6.49E-05	-9.6429
22 min	0.03425	0.02925	0.0085	1.06E-07	6.49E-05	-9.64276
24 min	0.03475	0.02975	0.009	1.13E-07	6.49E-05	-9.64286
26 min	0.035	0.03	0.00925	1.16E-07	6.49E-05	-9.6429
28 min	0.0365	0.031	0.01025	1.28E-07	6.49E-05	-9.6431
30 min	0.03775	0.03225	0.0115	1.44E-07	6.49E-05	-9.64334
32 min	0.03775	0.0325	0.01175	1.47E-07	6.49E-05	-9.64339
34 min	0.0385	0.0335	0.01275	1.59E-07	6.48E-05	-9.64358
36 min	0.04	0.03425	0.0135	1.69E-07	6.48E-05	-9.64372
38 min	0.03925	0.034	0.01325	1.66E-07	6.48E-05	-9.64367
40 min	0.041	0.035	0.01425	1.78E-07	6.48E-05	-9.64387
42 min	0.04225	0.03625	0.0155	1.94E-07	6.48E-05	-9.64411
44 min	0.0425	0.037	0.01625	2.03E-07	6.48E-05	-9.64425
46 min	0.04325	0.038	0.01725	2.16E-07	6.48E-05	-9.64445

48 min	0.04475	0.03875	0.018	2.25E-07	6.48E-05	-9.64459
50 min	0.04525	0.04025	0.0195	2.44E-07	6.48E-05	-9.64488
52 min	0.047	0.041	0.02025	2.53E-07	6.47E-05	-9.64503
54 min	0.0465	0.04075	0.02	2.5E-07	6.48E-05	-9.64498
56 min	0.048	0.042	0.02125	2.66E-07	6.47E-05	-9.64522
58 min	0.0495	0.042	0.02125	2.66E-07	6.47E-05	-9.64522
60 min	0.05	0.044	0.02325	2.91E-07	6.47E-05	-9.6456
62 min	0.0505	0.0445	0.02375	2.97E-07	6.47E-05	-9.6457
64 min	0.05125	0.0455	0.02475	3.09E-07	6.47E-05	-9.64589
66 min	0.05275	0.04725	0.0265	3.31E-07	6.47E-05	-9.64623
68 min	0.05275	0.047	0.02625	3.28E-07	6.47E-05	-9.64618
70 min	0.05525	0.049	0.02825	3.53E-07	6.46E-05	-9.64657
72 min	0.0555	0.05	0.02925	3.66E-07	6.46E-05	-9.64676
74 min	0.058	0.0515	0.03075	3.84E-07	6.46E-05	-9.64705
76 min	0.058	0.052	0.03125	3.91E-07	6.46E-05	-9.64715
78 min	0.0595	0.05325	0.0325	4.06E-07	6.46E-05	-9.64739
80 min	0.06025	0.0545	0.03375	4.22E-07	6.46E-05	-9.64763
82 min	0.06175	0.0555	0.03475	4.34E-07	6.46E-05	-9.64783
84 min	0.06275	0.05675	0.036	4.5E-07	6.46E-05	-9.64807
86 min	0.06375	0.058	0.03725	4.66E-07	6.45E-05	-9.64831
88 min	0.06475	0.06	0.03925	4.91E-07	6.45E-05	-9.6487
90 min	0.06675	0.06125	0.0405	5.06E-07	6.45E-05	-9.64894
92 min	0.06725	0.06125	0.0405	5.06E-07	6.45E-05	-9.64894
94 min	0.068	0.06225	0.0415	5.19E-07	6.45E-05	-9.64914
96 min	0.06925	0.06375	0.043	5.38E-07	6.45E-05	-9.64943
98 min	0.0705	0.065	0.04425	5.53E-07	6.44E-05	-9.64967
100 min	0.07125	0.0655	0.04475	5.59E-07	6.44E-05	-9.64977
102 min	0.07275	0.067	0.04625	5.78E-07	6.44E-05	-9.65006
104 min	0.0745	0.068	0.04725	5.91E-07	6.44E-05	-9.65025
106 min	0.07575	0.071	0.05025	6.28E-07	6.44E-05	-9.65083
108 min	0.077	0.0715	0.05075	6.34E-07	6.44E-05	-9.65093
110 min	0.07775	0.0725	0.05175	6.47E-07	6.44E-05	-9.65113
112 min	0.08	0.07375	0.053	6.63E-07	6.43E-05	-9.65137
114 min	0.08025	0.0745	0.05375	6.72E-07	6.43E-05	-9.65151
116 min	0.0815	0.07725	0.0565	7.06E-07	6.43E-05	-9.65205
118 min	0.083	0.0775	0.05675	7.09E-07	6.43E-05	-9.6521
120 min	0.0835	0.07825	0.0575	7.19E-07	6.43E-05	-9.65224
122 min	0.08575	0.07925	0.0585	7.31E-07	6.43E-05	-9.65244
124 min	0.08725	0.08125	0.0605	7.56E-07	6.42E-05	-9.65283
126 min	0.08775	0.08275	0.062	7.75E-07	6.42E-05	-9.65312
128 min	0.088	0.08275	0.062	7.75E-07	6.42E-05	-9.65312
130 min	0.09	0.0845	0.06375	7.97E-07	6.42E-05	-9.65346
132 min	0.09025	0.0855	0.06475	8.09E-07	6.42E-05	-9.65365
134 min	0.09125	0.086	0.06525	8.16E-07	6.42E-05	-9.65375
136 min	0.0945	0.0885	0.06775	8.47E-07	6.42E-05	-9.65424
138 min	0.09525	0.0895	0.06875	8.59E-07	6.41E-05	-9.65443

140 min	0.09675	0.09125	0.0705	8.81E-07	6.41E-05	-9.65477
142 min	0.0975	0.092	0.07125	8.91E-07	6.41E-05	-9.65492
144 min	0.10025	0.0945	0.07375	9.22E-07	6.41E-05	-9.65541
146 min	0.10075	0.095	0.07425	9.28E-07	6.41E-05	-9.65551
148 min	0.10175	0.0955	0.07475	9.34E-07	6.41E-05	-9.6556
150 min	0.1025	0.097	0.07625	9.53E-07	6.4E-05	-9.6559
152 min	0.10425	0.09825	0.0775	9.69E-07	6.4E-05	-9.65614
154 min	0.10575	0.09975	0.079	9.88E-07	6.4E-05	-9.65643
156 min	0.107	0.101	0.08025	1E-06	6.4E-05	-9.65668
158 min	0.10875	0.1025	0.08175	1.02E-06	6.4E-05	-9.65697
160 min	0.10925	0.1025	0.08175	1.02E-06	6.4E-05	-9.65697
162 min	0.11125	0.1045	0.08375	1.05E-06	6.4E-05	-9.65736
164 min	0.111	0.10475	0.084	1.05E-06	6.4E-05	-9.65741
166 min	0.11375	0.10725	0.0865	1.08E-06	6.39E-05	-9.6579
168 min	0.11475	0.1075	0.08675	1.08E-06	6.39E-05	-9.65795
170 min	0.11625	0.10925	0.0885	1.11E-06	6.39E-05	-9.65829
172 min	0.118	0.1115	0.09075	1.13E-06	6.39E-05	-9.65873
174 min	0.1185	0.11175	0.091	1.14E-06	6.39E-05	-9.65878
176 min	0.11925	0.1125	0.09175	1.15E-06	6.39E-05	-9.65893
178 min	0.12075	0.114	0.09325	1.17E-06	6.38E-05	-9.65922
180 min	0.123	0.1175	0.09675	1.21E-06	6.38E-05	-9.6599
182 min	0.12375	0.11725	0.0965	1.21E-06	6.38E-05	-9.65986
184 min	0.1245	0.11825	0.0975	1.22E-06	6.38E-05	-9.66005
186 min	0.12725	0.121	0.10025	1.25E-06	6.37E-05	-9.66059
188 min	0.1275	0.121	0.10025	1.25E-06	6.37E-05	-9.66059
190 min	0.129	0.12275	0.102	1.28E-06	6.37E-05	-9.66093
192 min	0.13	0.12425	0.1035	1.29E-06	6.37E-05	-9.66123
194 min	0.1315	0.125	0.10425	1.3E-06	6.37E-05	-9.66138
196 min	0.133	0.12625	0.1055	1.32E-06	6.37E-05	-9.66162
198 min	0.1345	0.12725	0.1065	1.33E-06	6.37E-05	-9.66182
200 min	0.1355	0.1285	0.10775	1.35E-06	6.37E-05	-9.66206
202 min	0.1365	0.13	0.10925	1.37E-06	6.36E-05	-9.66236
204 min	0.13775	0.131	0.11025	1.38E-06	6.36E-05	-9.66255
206 min	0.13875	0.13225	0.1115	1.39E-06	6.36E-05	-9.6628
208 min	0.1405	0.13375	0.113	1.41E-06	6.36E-05	-9.66309
210 min	0.141	0.135	0.11425	1.43E-06	6.36E-05	-9.66334
212 min	0.14325	0.1365	0.11575	1.45E-06	6.36E-05	-9.66363
214 min	0.14275	0.13725	0.1165	1.46E-06	6.35E-05	-9.66378
216 min	0.145	0.13775	0.117	1.46E-06	6.35E-05	-9.66388
218 min	0.14625	0.1395	0.11875	1.48E-06	6.35E-05	-9.66422
220 min	0.147	0.1405	0.11975	1.5E-06	6.35E-05	-9.66442
222 min	0.1485	0.14175	0.121	1.51E-06	6.35E-05	-9.66467
224 min	0.1495	0.14275	0.122	1.53E-06	6.35E-05	-9.66486
226 min	0.151	0.14375	0.123	1.54E-06	6.35E-05	-9.66506
228 min	0.153	0.146	0.12525	1.57E-06	6.34E-05	-9.6655
230 min	0.15275	0.14575	0.125	1.56E-06	6.34E-05	-9.66546

232 min	0.153	0.1465	0.12575	1.57E-06	6.34E-05	-9.6656
234 min	0.15525	0.14825	0.1275	1.59E-06	6.34E-05	-9.66595
236 min	0.157	0.14925	0.1285	1.61E-06	6.34E-05	-9.66615
238 min	0.1575	0.1495	0.12875	1.61E-06	6.34E-05	-9.66619
240 min	0.1585	0.15125	0.1305	1.63E-06	6.34E-05	-9.66654
242 min	0.1605	0.15275	0.132	1.65E-06	6.34E-05	-9.66684
244 min	0.162	0.154	0.13325	1.67E-06	6.33E-05	-9.66708
246 min	0.16325	0.155	0.13425	1.68E-06	6.33E-05	-9.66728
248 min	0.16175	0.155	0.13425	1.68E-06	6.33E-05	-9.66728
250 min	0.16475	0.1565	0.13575	1.7E-06	6.33E-05	-9.66758
252 min	0.16575	0.1575	0.13675	1.71E-06	6.33E-05	-9.66777
254 min	0.1665	0.1585	0.13775	1.72E-06	6.33E-05	-9.66797
256 min	0.1685	0.16075	0.14	1.75E-06	6.33E-05	-9.66842
258 min	0.169	0.16175	0.141	1.76E-06	6.32E-05	-9.66861
260 min	0.17025	0.16225	0.1415	1.77E-06	6.32E-05	-9.66871
262 min	0.17175	0.16375	0.143	1.79E-06	6.32E-05	-9.66901
264 min	0.1725	0.1645	0.14375	1.8E-06	6.32E-05	-9.66916
266 min	0.17425	0.166	0.14525	1.82E-06	6.32E-05	-9.66945
268 min	0.175	0.1665	0.14575	1.82E-06	6.32E-05	-9.66955
270 min	0.177	0.169	0.14825	1.85E-06	6.31E-05	-9.67005
272 min	0.17675	0.16875	0.148	1.85E-06	6.32E-05	-9.67
274 min	0.17875	0.169	0.14825	1.85E-06	6.31E-05	-9.67005
276 min	0.18075	0.17225	0.1515	1.89E-06	6.31E-05	-9.67069
278 min	0.181	0.1725	0.15175	1.9E-06	6.31E-05	-9.67074
280 min	0.1825	0.17375	0.153	1.91E-06	6.31E-05	-9.67099
282 min	0.18325	0.17475	0.154	1.93E-06	6.31E-05	-9.67119
284 min	0.184	0.1755	0.15475	1.93E-06	6.31E-05	-9.67133
286 min	0.18525	0.177	0.15625	1.95E-06	6.3E-05	-9.67163
288 min	0.18625	0.1775	0.15675	1.96E-06	6.3E-05	-9.67173
290 min	0.187	0.1775	0.15675	1.96E-06	6.3E-05	-9.67173
292 min	0.189	0.18075	0.16	0.000002	0.000063	-9.67238
294 min	0.189	0.1805	0.15975	2E-06	6.3E-05	-9.67233
296 min	0.19025	0.18	0.15925	1.99E-06	6.3E-05	-9.67223
298 min	0.1915	0.18275	0.162	2.03E-06	6.3E-05	-9.67277

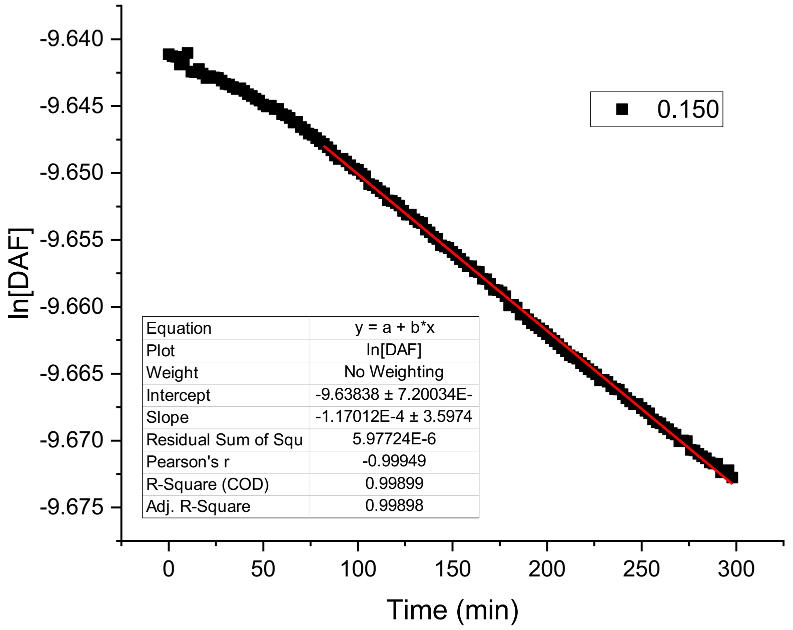

Catalysis of **DAF** to **FLU** in the presence of **CoHwBF$_4$** and various anions (0.25 mM **DAF**, 5 mM anion, 50 mM pH 7 **PBS** buffer, 0.1 mM **CoH^{w}BF$_4$**)

Catalysis of **DAF** to **FLU** in the presence of cage (0.25 mM **DAF**, 50 mM pH 7 **PBS** buffer, 0.1 mM **CoH^{wBF}$_4$**)

Time (min)	A@490nm	[FLU] (M)	[DAF] (M)	ln[DAF]
0	0	0	0.00025	-8.29405
2	0.00475	5.94E-08	0.0002499	-8.29429
4	0.0055	6.88E-08	0.0002499	-8.29432
6	0.005	6.25E-08	0.0002499	-8.2943
8	0.0055	6.88E-08	0.0002499	-8.29432
10	0.00475	5.94E-08	0.0002499	-8.29429
12	0.00575	7.19E-08	0.0002499	-8.29434
14	0.00175	2.19E-08	0.00025	-8.29414
16	0.00775	9.69E-08	0.0002499	-8.29444
18	0.00575	7.19E-08	0.0002499	-8.29434
20	0.00625	7.81E-08	0.0002499	-8.29436
22	0.01075	1.34E-07	0.0002499	-8.29459
24	0.0055	6.88E-08	0.0002499	-8.29432
26	0.0085	1.06E-07	0.0002499	-8.29447
28	0.01175	1.47E-07	0.0002499	-8.29464
30	0.00975	1.22E-07	0.0002499	-8.29454
32	0.0105	1.31E-07	0.0002499	-8.29457
34	0.01	1.25E-07	0.0002499	-8.29455
36	0.0115	1.44E-07	0.0002499	-8.29462

38	0.01175	1.47E-07	0.0002499	-8.29464
40	0.0125	1.56E-07	0.0002498	-8.29467
42	0.0115	1.44E-07	0.0002499	-8.29462
44	0.0135	1.69E-07	0.0002498	-8.29472
46	0.014	1.75E-07	0.0002498	-8.29475
48	0.01475	1.84E-07	0.0002498	-8.29479
50	0.01625	2.03E-07	0.0002498	-8.29486
52	0.01975	2.47E-07	0.0002498	-8.29504
54	0.02075	2.59E-07	0.0002497	-8.29509
56	0.01825	2.28E-07	0.0002498	-8.29496
58	0.01825	2.28E-07	0.0002498	-8.29496
60	0.02	2.5E-07	0.0002498	-8.29505
62	0.0235	2.94E-07	0.0002497	-8.29523
64	0.0215	2.69E-07	0.0002497	-8.29513
66	0.0225	2.81E-07	0.0002497	-8.29518
68	0.024	3E-07	0.0002497	-8.29525
70	0.02425	3.03E-07	0.0002497	-8.29526
72	0.02575	3.22E-07	0.0002497	-8.29534
74	0.02775	3.47E-07	0.0002497	-8.29544
76	0.02925	3.66E-07	0.0002496	-8.29551
78	0.027	3.38E-07	0.0002497	-8.2954
80	0.03	3.75E-07	0.0002496	-8.29555
82	0.03825	4.78E-07	0.0002495	-8.29596
84	0.03225	4.03E-07	0.0002496	-8.29566
86	0.0345	4.31E-07	0.0002496	-8.29578
88	0.034	4.25E-07	0.0002496	-8.29575
90	0.037	4.63E-07	0.0002495	-8.2959
92	0.04475	5.59E-07	0.0002494	-8.29629
94	0.037	4.63E-07	0.0002495	-8.2959
96	0.04025	5.03E-07	0.0002495	-8.29606
98	0.04025	5.03E-07	0.0002495	-8.29606
100	0.04325	5.41E-07	0.0002495	-8.29621
102	0.04275	5.34E-07	0.0002495	-8.29619
104	0.04525	5.66E-07	0.0002494	-8.29631
106	0.04625	5.78E-07	0.0002494	-8.29636
108	0.04575	5.72E-07	0.0002494	-8.29634
110	0.05	6.25E-07	0.0002494	-8.29655
112	0.04875	6.09E-07	0.0002494	-8.29649
114	0.05075	6.34E-07	0.0002494	-8.29659
116	0.053	6.63E-07	0.0002493	-8.2967
118	0.05225	6.53E-07	0.0002493	-8.29667
120	0.055	6.88E-07	0.0002493	-8.2968
122	0.057	7.13E-07	0.0002493	-8.2969
124	0.06125	7.66E-07	0.0002492	-8.29712
126	0.06	7.5E-07	0.0002493	-8.29705
128	0.0605	7.56E-07	0.0002492	-8.29708

130	0.06125	7.66E-07	0.0002492	-8.29712
132	0.0655	8.19E-07	0.0002492	-8.29733
134	0.07075	8.84E-07	0.0002491	-8.29759
136	0.06725	8.41E-07	0.0002492	-8.29742
138	0.0665	8.31E-07	0.0002492	-8.29738
140	0.06975	8.72E-07	0.0002491	-8.29754
142	0.07175	8.97E-07	0.0002491	-8.29764
144	0.07425	9.28E-07	0.0002491	-8.29777
146	0.0735	9.19E-07	0.0002491	-8.29773
148	0.0775	9.69E-07	0.000249	-8.29793
150	0.07625	9.53E-07	0.000249	-8.29787
152	0.07875	9.84E-07	0.000249	-8.29799
154	0.08025	1E-06	0.000249	-8.29807
156	0.0815	1.02E-06	0.000249	-8.29813
158	0.082	1.03E-06	0.000249	-8.29816
160	0.08325	1.04E-06	0.000249	-8.29822
162	0.08425	1.05E-06	0.0002489	-8.29827
164	0.0865	1.08E-06	0.0002489	-8.29838
166	0.0885	1.11E-06	0.0002489	-8.29848
168	0.0885	1.11E-06	0.0002489	-8.29848
170	0.0915	1.14E-06	0.0002489	-8.29864
172	0.09175	1.15E-06	0.0002489	-8.29865
174	0.094	1.18E-06	0.0002488	-8.29876
176	0.09525	1.19E-06	0.0002488	-8.29882
178	0.097	1.21E-06	0.0002488	-8.29891
180	0.09975	1.25E-06	0.0002488	-8.29905
182	0.1005	1.26E-06	0.0002487	-8.29909
184	0.102	1.28E-06	0.0002487	-8.29916
186	0.10325	1.29E-06	0.0002487	-8.29923
188	0.105	1.31E-06	0.0002487	-8.29931
190	0.1035	1.29E-06	0.0002487	-8.29924
192	0.10925	1.37E-06	0.0002486	-8.29953
194	0.10975	1.37E-06	0.0002486	-8.29955
196	0.11075	1.38E-06	0.0002486	-8.2996
198	0.11175	1.4E-06	0.0002486	-8.29965
200	0.113	1.41E-06	0.0002486	-8.29972
202	0.11625	1.45E-06	0.0002485	-8.29988
204	0.117	1.46E-06	0.0002485	-8.29992
206	0.118	1.48E-06	0.0002485	-8.29997
208	0.12	1.5E-06	0.0002485	-8.30007
210	0.12125	1.52E-06	0.0002485	-8.30013
212	0.1235	1.54E-06	0.0002485	-8.30024
214	0.12475	1.56E-06	0.0002484	-8.30031
216	0.1265	1.58E-06	0.0002484	-8.30039
218	0.128	1.6E-06	0.0002484	-8.30047
220	0.12875	1.61E-06	0.0002484	-8.30051

222	0.1305	1.63E-06	0.0002484	-8.3006
224	0.133	1.66E-06	0.0002483	-8.30072
226	0.13475	1.68E-06	0.0002483	-8.30081
228	0.13375	1.67E-06	0.0002483	-8.30076
230	0.138	1.73E-06	0.0002483	-8.30097
232	0.14	1.75E-06	0.0002483	-8.30107
234	0.1405	1.76E-06	0.0002482	-8.3011
236	0.14125	1.77E-06	0.0002482	-8.30114
238	0.1435	1.79E-06	0.0002482	-8.30125
240	0.145	1.81E-06	0.0002482	-8.30133
242	0.14625	1.83E-06	0.0002482	-8.30139
244	0.148	1.85E-06	0.0002482	-8.30148
246	0.14975	1.87E-06	0.0002481	-8.30157
248	0.151	1.89E-06	0.0002481	-8.30163
250	0.15225	1.9E-06	0.0002481	-8.30169
252	0.1535	1.92E-06	0.0002481	-8.30175
254	0.156	1.95E-06	0.0002481	-8.30188
256	0.156	1.95E-06	0.0002481	-8.30188
258	0.15875	1.98E-06	0.000248	-8.30202
260	0.1595	1.99E-06	0.000248	-8.30206
262	0.162	2.03E-06	0.000248	-8.30218
264	0.16325	2.04E-06	0.000248	-8.30225
266	0.1645	2.06E-06	0.0002479	-8.30231
268	0.1665	2.08E-06	0.0002479	-8.30241
270	0.169	2.11E-06	0.0002479	-8.30254
272	0.1685	2.11E-06	0.0002479	-8.30251
274	0.1715	2.14E-06	0.0002479	-8.30266
276	0.17	2.13E-06	0.0002479	-8.30259
278	0.1735	2.17E-06	0.0002478	-8.30276
280	0.1745	2.18E-06	0.0002478	-8.30281
282	0.1755	2.19E-06	0.0002478	-8.30286
284	0.17775	2.22E-06	0.0002478	-8.30298
286	0.17775	2.22E-06	0.0002478	-8.30298
288	0.18125	2.27E-06	0.0002477	-8.30315
290	0.18325	2.29E-06	0.0002477	-8.30325
292	0.18425	2.3E-06	0.0002477	-8.3033
294	0.188	2.35E-06	0.0002477	-8.30349
296	0.1875	2.34E-06	0.0002477	-8.30347
298	0.19	2.38E-06	0.0002476	-8.3036

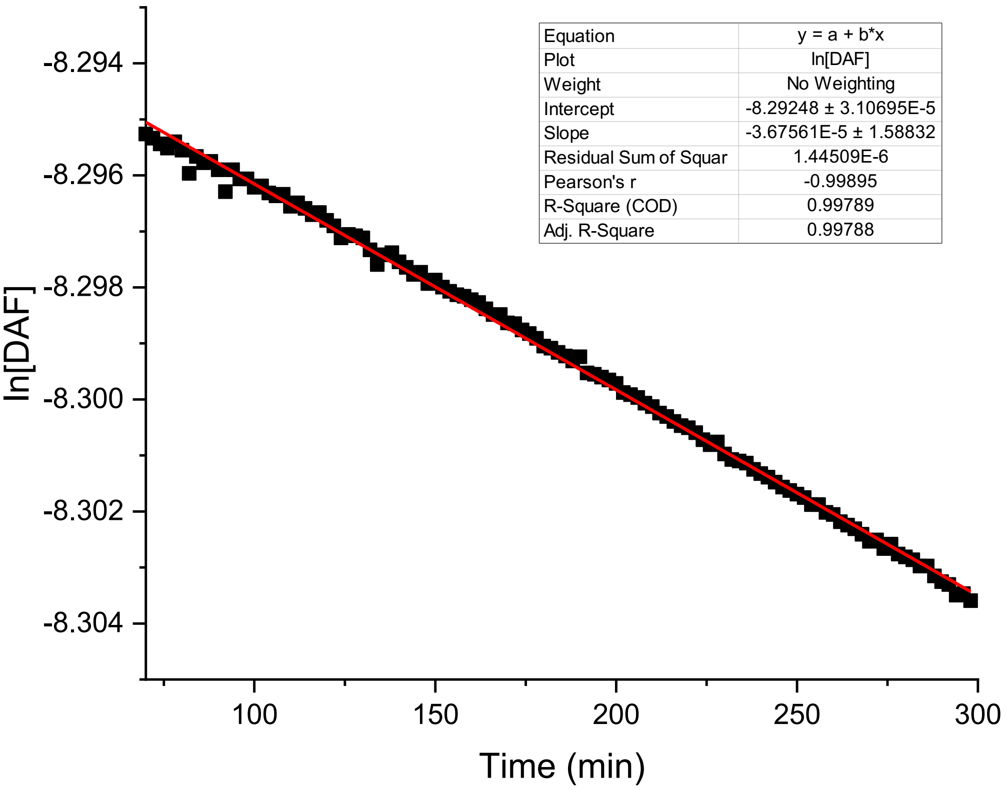
Equation
y = a + b*x
Plot
ln[DAF]
Weight
No Weighting
Intercept
-8.29248 ± 3.10695E-5
Slope
-3.67561E-5 ± 1.58832
Residual Sum of Squar
1.44509E-6
Pearson's r
-0.99895
R-Square (COD)
0.99789
Adj. R-Square
0.99788
-8.294
-8.296
-8.298
-8.300
-8.302
-8.304
ln[DAF]
100
150
200
250
300
Time (min)

Catalysis of **DAF** to **FLU** in the presence of cage and **5 mM NaF** (0.25 mM **DAF**, 50 mM pH 7 **PBS** buffer, 0.1 mM **CoH^{w}BF$_4$**)

Time (min)	A@490nm	set to 0	[FLU] (M)	[DAF] (M)	ln[DAF]
0	0.0225	0	0	0.00025	-8.29405
2	0.0315	0.009	1.13E-07	0.00025	-8.2945
4	0.03	0.0075	9.38E-08	0.00025	-8.29442
6	0.0355	0.013	1.63E-07	0.00025	-8.2947
8	0.0325	0.01	1.25E-07	0.00025	-8.29455
10	0.0345	0.012	1.5E-07	0.00025	-8.29465
12	0.037	0.0145	1.81E-07	0.00025	-8.29477
14	0.03175	0.00925	1.16E-07	0.00025	-8.29451
16	0.03625	0.01375	1.72E-07	0.00025	-8.29474
18	0.038	0.0155	1.94E-07	0.00025	-8.29482
20	0.0395	0.017	2.13E-07	0.00025	-8.2949
22	0.0425	0.02	2.5E-07	0.00025	-8.29505
24	0.0445	0.022	2.75E-07	0.00025	-8.29515
26	0.0435	0.021	2.63E-07	0.00025	-8.2951
28	0.04625	0.02375	2.97E-07	0.00025	-8.29524
30	0.0455	0.023	2.88E-07	0.00025	-8.2952
32	0.04675	0.02425	3.03E-07	0.00025	-8.29526
34	0.04825	0.02575	3.22E-07	0.00025	-8.29534
36	0.052	0.0295	3.69E-07	0.00025	-8.29553
38	0.05175	0.02925	3.66E-07	0.00025	-8.29551
40	0.05275	0.03025	3.78E-07	0.00025	-8.29556
42	0.05575	0.03325	4.16E-07	0.00025	-8.29571
44	0.058	0.0355	4.44E-07	0.00025	-8.29583
46	0.05875	0.03625	4.53E-07	0.00025	-8.29586
48	0.05975	0.03725	4.66E-07	0.00025	-8.29591
50	0.06525	0.04275	5.34E-07	0.000249	-8.29619
52	0.0655	0.043	5.38E-07	0.000249	-8.2962
54	0.06875	0.04625	5.78E-07	0.000249	-8.29636
56	0.067	0.0445	5.56E-07	0.000249	-8.29628
58	0.0705	0.048	6E-07	0.000249	-8.29645
60	0.071	0.0485	6.06E-07	0.000249	-8.29648
62	0.077	0.0545	6.81E-07	0.000249	-8.29678
64	0.07475	0.05225	6.53E-07	0.000249	-8.29667
66	0.07775	0.05525	6.91E-07	0.000249	-8.29682
68	0.08	0.0575	7.19E-07	0.000249	-8.29693
70	0.08425	0.06175	7.72E-07	0.000249	-8.29714
72	0.08375	0.06125	7.66E-07	0.000249	-8.29712
74	0.08675	0.06425	8.03E-07	0.000249	-8.29727
76	0.08975	0.06725	8.41E-07	0.000249	-8.29742
78	0.09025	0.06775	8.47E-07	0.000249	-8.29744

80	0.09325	0.07075	8.84E-07	0.000249	-8.29759
82	0.09975	0.07725	9.66E-07	0.000249	-8.29792
84	0.10075	0.07825	9.78E-07	0.000249	-8.29797
86	0.0995	0.077	9.63E-07	0.000249	-8.29791
88	0.102	0.0795	9.94E-07	0.000249	-8.29803
90	0.1035	0.081	1.01E-06	0.000249	-8.29811
92	0.10625	0.08375	1.05E-06	0.000249	-8.29825
94	0.10725	0.08475	1.06E-06	0.000249	-8.2983
96	0.111	0.0885	1.11E-06	0.000249	-8.29848
98	0.1125	0.09	1.13E-06	0.000249	-8.29856
100	0.11525	0.09275	1.16E-06	0.000249	-8.2987
102	0.11875	0.09625	1.2E-06	0.000249	-8.29887
104	0.12075	0.09825	1.23E-06	0.000249	-8.29897
106	0.124	0.1015	1.27E-06	0.000249	-8.29914
108	0.1235	0.101	1.26E-06	0.000249	-8.29911
110	0.12725	0.10475	1.31E-06	0.000249	-8.2993
112	0.12925	0.10675	1.33E-06	0.000249	-8.2994
114	0.13175	0.10925	1.37E-06	0.000249	-8.29953
116	0.1345	0.112	1.4E-06	0.000249	-8.29967
118	0.135	0.1125	1.41E-06	0.000249	-8.29969
120	0.13925	0.11675	1.46E-06	0.000249	-8.2999
122	0.141	0.1185	1.48E-06	0.000249	-8.29999
124	0.1435	0.121	1.51E-06	0.000248	-8.30012
126	0.147	0.1245	1.56E-06	0.000248	-8.30029
128	0.1475	0.125	1.56E-06	0.000248	-8.30032
130	0.1515	0.129	1.61E-06	0.000248	-8.30052
132	0.1535	0.131	1.64E-06	0.000248	-8.30062
134	0.1565	0.134	1.68E-06	0.000248	-8.30077
136	0.15975	0.13725	1.72E-06	0.000248	-8.30094
138	0.162	0.1395	1.74E-06	0.000248	-8.30105
140	0.16425	0.14175	1.77E-06	0.000248	-8.30116
142	0.16675	0.14425	1.8E-06	0.000248	-8.30129
144	0.1695	0.147	1.84E-06	0.000248	-8.30143
146	0.172	0.1495	1.87E-06	0.000248	-8.30155
148	0.1745	0.152	1.9E-06	0.000248	-8.30168
150	0.17775	0.15525	1.94E-06	0.000248	-8.30184
152	0.18	0.1575	1.97E-06	0.000248	-8.30196
154	0.185	0.1625	2.03E-06	0.000248	-8.30221
156	0.18425	0.16175	2.02E-06	0.000248	-8.30217
158	0.1865	0.164	2.05E-06	0.000248	-8.30228
160	0.189	0.1665	2.08E-06	0.000248	-8.30241
162	0.19075	0.16825	2.1E-06	0.000248	-8.3025
164	0.19425	0.17175	2.15E-06	0.000248	-8.30267
166	0.19575	0.17325	2.17E-06	0.000248	-8.30275
168	0.19875	0.17625	2.2E-06	0.000248	-8.3029
170	0.20225	0.17975	2.25E-06	0.000248	-8.30308

172	0.2055	0.183	2.29E-06	0.000248	-8.30324
174	0.2085	0.186	2.33E-06	0.000248	-8.30339
176	0.20975	0.18725	2.34E-06	0.000248	-8.30346
178	0.21275	0.19025	2.38E-06	0.000248	-8.30361
180	0.21475	0.19225	2.4E-06	0.000248	-8.30371
182	0.21775	0.19525	2.44E-06	0.000248	-8.30386
184	0.2205	0.198	2.48E-06	0.000248	-8.304
186	0.224	0.2015	2.52E-06	0.000247	-8.30418
188	0.2285	0.206	2.58E-06	0.000247	-8.3044
190	0.22775	0.20525	2.57E-06	0.000247	-8.30437
192	0.23275	0.21025	2.63E-06	0.000247	-8.30462
194	0.233	0.2105	2.63E-06	0.000247	-8.30463
196	0.235	0.2125	2.66E-06	0.000247	-8.30473
198	0.2415	0.219	2.74E-06	0.000247	-8.30506
200	0.2405	0.218	2.73E-06	0.000247	-8.30501
202	0.2445	0.222	2.78E-06	0.000247	-8.30521
204	0.24575	0.22325	2.79E-06	0.000247	-8.30527
206	0.24825	0.22575	2.82E-06	0.000247	-8.3054
208	0.252	0.2295	2.87E-06	0.000247	-8.30559
210	0.25175	0.22925	2.87E-06	0.000247	-8.30558
212	0.257	0.2345	2.93E-06	0.000247	-8.30584
214	0.25925	0.23675	2.96E-06	0.000247	-8.30596
216	0.262	0.2395	2.99E-06	0.000247	-8.3061
218	0.26375	0.24125	3.02E-06	0.000247	-8.30619
220	0.268	0.2455	3.07E-06	0.000247	-8.3064
222	0.26925	0.24675	3.08E-06	0.000247	-8.30646
224	0.2725	0.25	3.13E-06	0.000247	-8.30663
226	0.273	0.2505	3.13E-06	0.000247	-8.30665
228	0.276	0.2535	3.17E-06	0.000247	-8.30681
230	0.279	0.2565	3.21E-06	0.000247	-8.30696
232	0.28175	0.25925	3.24E-06	0.000247	-8.3071
234	0.28425	0.26175	3.27E-06	0.000247	-8.30722
236	0.28775	0.26525	3.32E-06	0.000247	-8.3074
238	0.29375	0.27125	3.39E-06	0.000247	-8.3077
240	0.29225	0.26975	3.37E-06	0.000247	-8.30763
242	0.29525	0.27275	3.41E-06	0.000247	-8.30778
244	0.2965	0.274	3.43E-06	0.000247	-8.30784
246	0.29925	0.27675	3.46E-06	0.000247	-8.30798
248	0.3005	0.278	3.48E-06	0.000247	-8.30805
250	0.3035	0.281	3.51E-06	0.000246	-8.3082
252	0.30625	0.28375	3.55E-06	0.000246	-8.30834
254	0.30875	0.28625	3.58E-06	0.000246	-8.30847
256	0.31175	0.28925	3.62E-06	0.000246	-8.30862
258	0.31425	0.29175	3.65E-06	0.000246	-8.30874
260	0.317	0.2945	3.68E-06	0.000246	-8.30888
262	0.32025	0.29775	3.72E-06	0.000246	-8.30905

264	0.3225	0.3	3.75E-06	0.000246	-8.30916
266	0.3245	0.302	3.78E-06	0.000246	-8.30926
268	0.329	0.3065	3.83E-06	0.000246	-8.30949
270	0.33125	0.30875	3.86E-06	0.000246	-8.30961
272	0.33175	0.30925	3.87E-06	0.000246	-8.30963
274	0.3355	0.313	3.91E-06	0.000246	-8.30982
276	0.337	0.3145	3.93E-06	0.000246	-8.3099
278	0.34125	0.31875	3.98E-06	0.000246	-8.31012
280	0.34425	0.32175	4.02E-06	0.000246	-8.31027
282	0.34675	0.32425	4.05E-06	0.000246	-8.3104
284	0.35025	0.32775	4.1E-06	0.000246	-8.31057
286	0.352	0.3295	4.12E-06	0.000246	-8.31066
288	0.35475	0.33225	4.15E-06	0.000246	-8.3108
290	0.35825	0.33575	4.2E-06	0.000246	-8.31098
292	0.359	0.3365	4.21E-06	0.000246	-8.31102
294	0.36075	0.33825	4.23E-06	0.000246	-8.31111
296	0.36275	0.34025	4.25E-06	0.000246	-8.31121
298	0.36575	0.34325	4.29E-06	0.000246	-8.31136

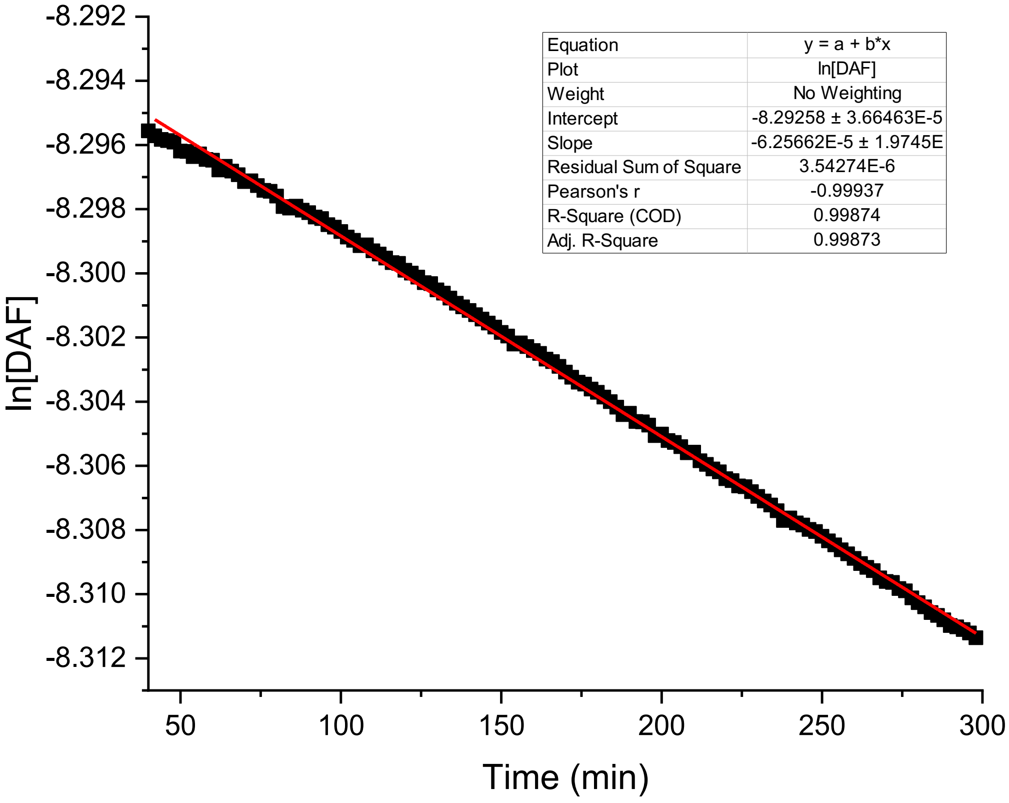

Catalysis of **DAF** to **FLU** in the presence of cage and **5 mM NaBr** (0.25 mM **DAF**, 50 mM pH 7 **PBS** buffer, 0.1 mM **$CoH^{w}BF_4$**)

Time (min)	A@490nm	set to 0	[FLU] (M)	[DAF] (M)	ln[DAF]
0	0.027	0	0	0.00025	-8.29405
2	0.03125	0.00425	5.31E-08	0.00025	-8.29426
4	0.03375	0.00675	8.44E-08	0.00025	-8.29439
6	0.03375	0.00675	8.44E-08	0.00025	-8.29439
8	0.0355	0.0085	1.06E-07	0.00025	-8.29447
10	0.03375	0.00675	8.44E-08	0.00025	-8.29439
12	0.036	0.009	1.13E-07	0.00025	-8.2945
14	0.0325	0.0055	6.88E-08	0.00025	-8.29432
16	0.03525	0.00825	1.03E-07	0.00025	-8.29446
18	0.03875	0.01175	1.47E-07	0.00025	-8.29464
20	0.037	0.01	1.25E-07	0.00025	-8.29455
22	0.036	0.009	1.13E-07	0.00025	-8.2945
24	0.037	0.01	1.25E-07	0.00025	-8.29455
26	0.03725	0.01025	1.28E-07	0.00025	-8.29456
28	0.03725	0.01025	1.28E-07	0.00025	-8.29456
30	0.037	0.01	1.25E-07	0.00025	-8.29455
32	0.038	0.011	1.38E-07	0.00025	-8.2946
34	0.04025	0.01325	1.66E-07	0.00025	-8.29471
36	0.0415	0.0145	1.81E-07	0.00025	-8.29477
38	0.03925	0.01225	1.53E-07	0.00025	-8.29466
40	0.0385	0.0115	1.44E-07	0.00025	-8.29462
42	0.03725	0.01025	1.28E-07	0.00025	-8.29456
44	0.04175	0.01475	1.84E-07	0.00025	-8.29479
46	0.0415	0.0145	1.81E-07	0.00025	-8.29477
48	0.04075	0.01375	1.72E-07	0.00025	-8.29474
50	0.03975	0.01275	1.59E-07	0.00025	-8.29469
52	0.04175	0.01475	1.84E-07	0.00025	-8.29479
54	0.0415	0.0145	1.81E-07	0.00025	-8.29477
56	0.04325	0.01625	2.03E-07	0.00025	-8.29486
58	0.045	0.018	2.25E-07	0.00025	-8.29495
60	0.043	0.016	2E-07	0.00025	-8.29485
62	0.04325	0.01625	2.03E-07	0.00025	-8.29486
64	0.04775	0.02075	2.59E-07	0.00025	-8.29509
66	0.0465	0.0195	2.44E-07	0.00025	-8.29503
68	0.04525	0.01825	2.28E-07	0.00025	-8.29496
70	0.04725	0.02025	2.53E-07	0.00025	-8.29506
72	0.04825	0.02125	2.66E-07	0.00025	-8.29511
74	0.0455	0.0185	2.31E-07	0.00025	-8.29498
76	0.046	0.019	2.38E-07	0.00025	-8.295
78	0.0475	0.0205	2.56E-07	0.00025	-8.29508
80	0.05	0.023	2.88E-07	0.00025	-8.2952
82	0.05	0.023	2.88E-07	0.00025	-8.2952
84	0.048	0.021	2.63E-07	0.00025	-8.2951

86	0.05025	0.02325	2.91E-07	0.00025	-8.29521
88	0.05325	0.02625	3.28E-07	0.00025	-8.29536
90	0.05175	0.02475	3.09E-07	0.00025	-8.29529
92	0.05075	0.02375	2.97E-07	0.00025	-8.29524
94	0.05	0.023	2.88E-07	0.00025	-8.2952
96	0.05325	0.02625	3.28E-07	0.00025	-8.29536
98	0.052	0.025	3.13E-07	0.00025	-8.2953
100	0.0535	0.0265	3.31E-07	0.00025	-8.29538
102	0.054	0.027	3.38E-07	0.00025	-8.2954
104	0.056	0.029	3.63E-07	0.00025	-8.2955
106	0.0555	0.0285	3.56E-07	0.00025	-8.29548
108	0.0545	0.0275	3.44E-07	0.00025	-8.29543
110	0.057	0.03	3.75E-07	0.00025	-8.29555
112	0.05625	0.02925	3.66E-07	0.00025	-8.29551
114	0.05725	0.03025	3.78E-07	0.00025	-8.29556
116	0.05875	0.03175	3.97E-07	0.00025	-8.29564
118	0.0575	0.0305	3.81E-07	0.00025	-8.29558
120	0.061	0.034	4.25E-07	0.00025	-8.29575
122	0.05925	0.03225	4.03E-07	0.00025	-8.29566
124	0.061	0.034	4.25E-07	0.00025	-8.29575
126	0.06175	0.03475	4.34E-07	0.00025	-8.29579
128	0.0605	0.0335	4.19E-07	0.00025	-8.29573
130	0.06275	0.03575	4.47E-07	0.00025	-8.29584
132	0.06225	0.03525	4.41E-07	0.00025	-8.29581
134	0.064	0.037	4.63E-07	0.00025	-8.2959
136	0.0655	0.0385	4.81E-07	0.00025	-8.29598
138	0.06525	0.03825	4.78E-07	0.00025	-8.29596
140	0.0655	0.0385	4.81E-07	0.00025	-8.29598
142	0.06775	0.04075	5.09E-07	0.000249	-8.29609
144	0.067	0.04	5E-07	0.00025	-8.29605
146	0.06925	0.04225	5.28E-07	0.000249	-8.29616
148	0.06975	0.04275	5.34E-07	0.000249	-8.29619
150	0.06825	0.04125	5.16E-07	0.000249	-8.29611
152	0.07025	0.04325	5.41E-07	0.000249	-8.29621
154	0.0705	0.0435	5.44E-07	0.000249	-8.29623
156	0.07175	0.04475	5.59E-07	0.000249	-8.29629
158	0.07175	0.04475	5.59E-07	0.000249	-8.29629
160	0.07175	0.04475	5.59E-07	0.000249	-8.29629
162	0.0725	0.0455	5.69E-07	0.000249	-8.29633
164	0.074	0.047	5.88E-07	0.000249	-8.2964
166	0.07375	0.04675	5.84E-07	0.000249	-8.29639
168	0.07425	0.04725	5.91E-07	0.000249	-8.29641
170	0.07525	0.04825	6.03E-07	0.000249	-8.29647
172	0.07625	0.04925	6.16E-07	0.000249	-8.29652
174	0.078	0.051	6.38E-07	0.000249	-8.2966
176	0.07875	0.05175	6.47E-07	0.000249	-8.29664

178	0.0785	0.0515	6.44E-07	0.000249	-8.29663
180	0.0795	0.0525	6.56E-07	0.000249	-8.29668
182	0.08025	0.05325	6.66E-07	0.000249	-8.29672
184	0.0805	0.0535	6.69E-07	0.000249	-8.29673
186	0.08075	0.05375	6.72E-07	0.000249	-8.29674
188	0.0825	0.0555	6.94E-07	0.000249	-8.29683
190	0.0815	0.0545	6.81E-07	0.000249	-8.29678
192	0.08475	0.05775	7.22E-07	0.000249	-8.29694
194	0.08475	0.05775	7.22E-07	0.000249	-8.29694
196	0.085	0.058	7.25E-07	0.000249	-8.29695
198	0.0865	0.0595	7.44E-07	0.000249	-8.29703
200	0.087	0.06	7.5E-07	0.000249	-8.29705
202	0.08775	0.06075	7.59E-07	0.000249	-8.29709
204	0.09	0.063	7.88E-07	0.000249	-8.2972
206	0.08875	0.06175	7.72E-07	0.000249	-8.29714
208	0.09025	0.06325	7.91E-07	0.000249	-8.29722
210	0.08975	0.06275	7.84E-07	0.000249	-8.29719
212	0.0925	0.0655	8.19E-07	0.000249	-8.29733
214	0.09175	0.06475	8.09E-07	0.000249	-8.29729
216	0.0935	0.0665	8.31E-07	0.000249	-8.29738
218	0.09275	0.06575	8.22E-07	0.000249	-8.29734
220	0.0955	0.0685	8.56E-07	0.000249	-8.29748
222	0.09575	0.06875	8.59E-07	0.000249	-8.29749
224	0.096	0.069	8.63E-07	0.000249	-8.29751
226	0.09675	0.06975	8.72E-07	0.000249	-8.29754
228	0.09675	0.06975	8.72E-07	0.000249	-8.29754
230	0.0985	0.0715	8.94E-07	0.000249	-8.29763
232	0.099	0.072	9E-07	0.000249	-8.29766
234	0.10025	0.07325	9.16E-07	0.000249	-8.29772
236	0.10075	0.07375	9.22E-07	0.000249	-8.29774
238	0.10225	0.07525	9.41E-07	0.000249	-8.29782
240	0.10175	0.07475	9.34E-07	0.000249	-8.29779
242	0.10475	0.07775	9.72E-07	0.000249	-8.29794
244	0.10475	0.07775	9.72E-07	0.000249	-8.29794
246	0.1055	0.0785	9.81E-07	0.000249	-8.29798
248	0.10475	0.07775	9.72E-07	0.000249	-8.29794
250	0.1065	0.0795	9.94E-07	0.000249	-8.29803
252	0.106	0.079	9.88E-07	0.000249	-8.29801
254	0.1065	0.0795	9.94E-07	0.000249	-8.29803
256	0.10875	0.08175	1.02E-06	0.000249	-8.29815
258	0.10925	0.08225	1.03E-06	0.000249	-8.29817
260	0.11	0.083	1.04E-06	0.000249	-8.29821
262	0.112	0.085	1.06E-06	0.000249	-8.29831
264	0.11125	0.08425	1.05E-06	0.000249	-8.29827
266	0.114	0.087	1.09E-06	0.000249	-8.29841
268	0.1155	0.0885	1.11E-06	0.000249	-8.29848

270	0.11625	0.08925	1.12E-06	0.000249	-8.29852
272	0.114	0.087	1.09E-06	0.000249	-8.29841
274	0.11675	0.08975	1.12E-06	0.000249	-8.29855
276	0.115	0.088	1.1E-06	0.000249	-8.29846
278	0.1175	0.0905	1.13E-06	0.000249	-8.29858
280	0.11875	0.09175	1.15E-06	0.000249	-8.29865
282	0.11825	0.09125	1.14E-06	0.000249	-8.29862
284	0.12	0.093	1.16E-06	0.000249	-8.29871
286	0.1245	0.0975	1.22E-06	0.000249	-8.29894
288	0.128	0.101	1.26E-06	0.000249	-8.29911
290	0.12625	0.09925	1.24E-06	0.000249	-8.29902
292	0.1235	0.0965	1.21E-06	0.000249	-8.29889
294	0.125	0.098	1.23E-06	0.000249	-8.29896
296	0.12575	0.09875	1.23E-06	0.000249	-8.299
298	0.12825	0.10125	1.27E-06	0.000249	-8.29912

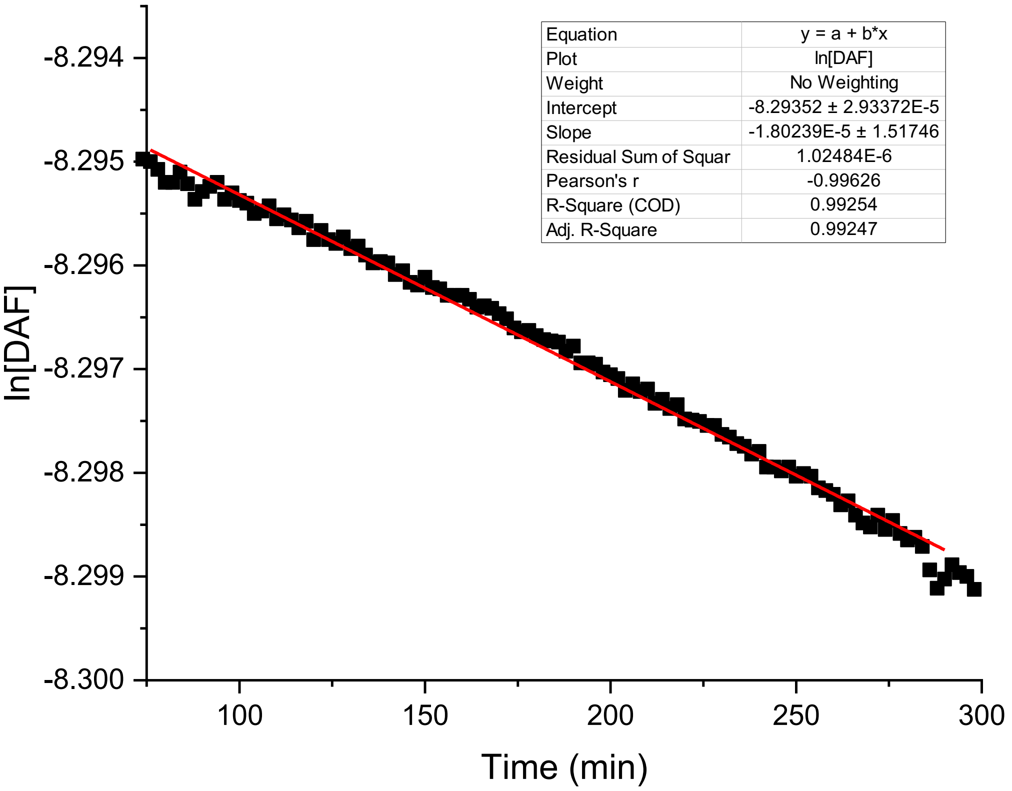

Catalysis of **DAF** to **FLU** in the presence of cage and **5 mM $NaNO_3$** (0.25 mM **DAF**, 50 mM pH 7 **PBS** buffer, 0.1 mM **$CoH^{w}BF_4$**)

Time (min)	A@490nm	set to 0	[FLU] (M)	[DAF] (M)	ln[DAF]
0	0.024	0	0	0.00025	-8.29405
2	0.03075	0.00675	8.44E-08	0.00025	-8.29439
4	0.034	0.01	1.25E-07	0.00025	-8.29455
6	0.03325	0.00925	1.16E-07	0.00025	-8.29451
8	0.03275	0.00875	1.09E-07	0.00025	-8.29449
10	0.0325	0.0085	1.06E-07	0.00025	-8.29447
12	0.03375	0.00975	1.22E-07	0.00025	-8.29454
14	0.02975	0.00575	7.19E-08	0.00025	-8.29434
16	0.0335	0.0095	1.19E-07	0.00025	-8.29452
18	0.035	0.011	1.38E-07	0.00025	-8.2946
20	0.03675	0.01275	1.59E-07	0.00025	-8.29469
22	0.03275	0.00875	1.09E-07	0.00025	-8.29449
24	0.03375	0.00975	1.22E-07	0.00025	-8.29454
26	0.03425	0.01025	1.28E-07	0.00025	-8.29456
28	0.034	0.01	1.25E-07	0.00025	-8.29455
30	0.03425	0.01025	1.28E-07	0.00025	-8.29456
32	0.03525	0.01125	1.41E-07	0.00025	-8.29461
34	0.035	0.011	1.38E-07	0.00025	-8.2946
36	0.03675	0.01275	1.59E-07	0.00025	-8.29469
38	0.03525	0.01125	1.41E-07	0.00025	-8.29461
40	0.035	0.011	1.38E-07	0.00025	-8.2946
42	0.035	0.011	1.38E-07	0.00025	-8.2946
44	0.037	0.013	1.63E-07	0.00025	-8.2947
46	0.03625	0.01225	1.53E-07	0.00025	-8.29466
48	0.03725	0.01325	1.66E-07	0.00025	-8.29471
50	0.03575	0.01175	1.47E-07	0.00025	-8.29464
52	0.03725	0.01325	1.66E-07	0.00025	-8.29471
54	0.03775	0.01375	1.72E-07	0.00025	-8.29474
56	0.03675	0.01275	1.59E-07	0.00025	-8.29469
58	0.03875	0.01475	1.84E-07	0.00025	-8.29479
60	0.03775	0.01375	1.72E-07	0.00025	-8.29474
62	0.04075	0.01675	2.09E-07	0.00025	-8.29489
64	0.0385	0.0145	1.81E-07	0.00025	-8.29477
66	0.03975	0.01575	1.97E-07	0.00025	-8.29484
68	0.04025	0.01625	2.03E-07	0.00025	-8.29486
70	0.04125	0.01725	2.16E-07	0.00025	-8.29491
72	0.04025	0.01625	2.03E-07	0.00025	-8.29486
74	0.0415	0.0175	2.19E-07	0.00025	-8.29493
76	0.041	0.017	2.13E-07	0.00025	-8.2949
78	0.0415	0.0175	2.19E-07	0.00025	-8.29493
80	0.04425	0.02025	2.53E-07	0.00025	-8.29506
82	0.046	0.022	2.75E-07	0.00025	-8.29515
84	0.04375	0.01975	2.47E-07	0.00025	-8.29504

86	0.04425	0.02025	2.53E-07	0.00025	-8.29506
88	0.04325	0.01925	2.41E-07	0.00025	-8.29501
90	0.0455	0.0215	2.69E-07	0.00025	-8.29513
92	0.04625	0.02225	2.78E-07	0.00025	-8.29516
94	0.04475	0.02075	2.59E-07	0.00025	-8.29509
96	0.046	0.022	2.75E-07	0.00025	-8.29515
98	0.04525	0.02125	2.66E-07	0.00025	-8.29511
100	0.04675	0.02275	2.84E-07	0.00025	-8.29519
102	0.04625	0.02225	2.78E-07	0.00025	-8.29516
104	0.0485	0.0245	3.06E-07	0.00025	-8.29528
106	0.04875	0.02475	3.09E-07	0.00025	-8.29529
108	0.04825	0.02425	3.03E-07	0.00025	-8.29526
110	0.04975	0.02575	3.22E-07	0.00025	-8.29534
112	0.05025	0.02625	3.28E-07	0.00025	-8.29536
114	0.05	0.026	3.25E-07	0.00025	-8.29535
116	0.05075	0.02675	3.34E-07	0.00025	-8.29539
118	0.04975	0.02575	3.22E-07	0.00025	-8.29534
120	0.051	0.027	3.38E-07	0.00025	-8.2954
122	0.05175	0.02775	3.47E-07	0.00025	-8.29544
124	0.0525	0.0285	3.56E-07	0.00025	-8.29548
126	0.05275	0.02875	3.59E-07	0.00025	-8.29549
128	0.05275	0.02875	3.59E-07	0.00025	-8.29549
130	0.05325	0.02925	3.66E-07	0.00025	-8.29551
132	0.0525	0.0285	3.56E-07	0.00025	-8.29548
134	0.05525	0.03125	3.91E-07	0.00025	-8.29561
136	0.05525	0.03125	3.91E-07	0.00025	-8.29561
138	0.05475	0.03075	3.84E-07	0.00025	-8.29559
140	0.056	0.032	4E-07	0.00025	-8.29565
142	0.0575	0.0335	4.19E-07	0.00025	-8.29573
144	0.057	0.033	4.13E-07	0.00025	-8.2957
146	0.05775	0.03375	4.22E-07	0.00025	-8.29574
148	0.058	0.034	4.25E-07	0.00025	-8.29575
150	0.0575	0.0335	4.19E-07	0.00025	-8.29573
152	0.05925	0.03525	4.41E-07	0.00025	-8.29581
154	0.059	0.035	4.38E-07	0.00025	-8.2958
156	0.06025	0.03625	4.53E-07	0.00025	-8.29586
158	0.06	0.036	4.5E-07	0.00025	-8.29585
160	0.06025	0.03625	4.53E-07	0.00025	-8.29586
162	0.06075	0.03675	4.59E-07	0.00025	-8.29589
164	0.062	0.038	4.75E-07	0.00025	-8.29595
166	0.06225	0.03825	4.78E-07	0.00025	-8.29596
168	0.06275	0.03875	4.84E-07	0.00025	-8.29599
170	0.063	0.039	4.88E-07	0.00025	-8.296
172	0.0645	0.0405	5.06E-07	0.000249	-8.29608
174	0.06525	0.04125	5.16E-07	0.000249	-8.29611
176	0.065	0.041	5.13E-07	0.000249	-8.2961

178	0.06625	0.04225	5.28E-07	0.000249	-8.29616
180	0.06625	0.04225	5.28E-07	0.000249	-8.29616
182	0.067	0.043	5.38E-07	0.000249	-8.2962
184	0.067	0.043	5.38E-07	0.000249	-8.2962
186	0.0665	0.0425	5.31E-07	0.000249	-8.29618
188	0.069	0.045	5.63E-07	0.000249	-8.2963
190	0.067	0.043	5.38E-07	0.000249	-8.2962
192	0.07025	0.04625	5.78E-07	0.000249	-8.29636
194	0.07025	0.04625	5.78E-07	0.000249	-8.29636
196	0.0695	0.0455	5.69E-07	0.000249	-8.29633
198	0.071	0.047	5.88E-07	0.000249	-8.2964
200	0.0715	0.0475	5.94E-07	0.000249	-8.29643
202	0.07325	0.04925	6.16E-07	0.000249	-8.29652
204	0.07225	0.04825	6.03E-07	0.000249	-8.29647
206	0.07325	0.04925	6.16E-07	0.000249	-8.29652
208	0.07525	0.05125	6.41E-07	0.000249	-8.29662
210	0.0745	0.0505	6.31E-07	0.000249	-8.29658
212	0.07475	0.05075	6.34E-07	0.000249	-8.29659
214	0.07475	0.05075	6.34E-07	0.000249	-8.29659
216	0.0765	0.0525	6.56E-07	0.000249	-8.29668
218	0.0755	0.0515	6.44E-07	0.000249	-8.29663
220	0.07725	0.05325	6.66E-07	0.000249	-8.29672
222	0.0775	0.0535	6.69E-07	0.000249	-8.29673
224	0.07975	0.05575	6.97E-07	0.000249	-8.29684
226	0.0775	0.0535	6.69E-07	0.000249	-8.29673
228	0.07825	0.05425	6.78E-07	0.000249	-8.29677
230	0.08025	0.05625	7.03E-07	0.000249	-8.29687
232	0.08075	0.05675	7.09E-07	0.000249	-8.29689
234	0.08025	0.05625	7.03E-07	0.000249	-8.29687
236	0.08125	0.05725	7.16E-07	0.000249	-8.29692
238	0.08275	0.05875	7.34E-07	0.000249	-8.29699
240	0.08275	0.05875	7.34E-07	0.000249	-8.29699
242	0.0845	0.0605	7.56E-07	0.000249	-8.29708
244	0.08325	0.05925	7.41E-07	0.000249	-8.29702
246	0.08475	0.06075	7.59E-07	0.000249	-8.29709
248	0.0835	0.0595	7.44E-07	0.000249	-8.29703
250	0.0855	0.0615	7.69E-07	0.000249	-8.29713
252	0.086	0.062	7.75E-07	0.000249	-8.29715
254	0.086	0.062	7.75E-07	0.000249	-8.29715
256	0.08825	0.06425	8.03E-07	0.000249	-8.29727
258	0.08875	0.06475	8.09E-07	0.000249	-8.29729
260	0.089	0.065	8.13E-07	0.000249	-8.2973
262	0.09025	0.06625	8.28E-07	0.000249	-8.29737
264	0.09025	0.06625	8.28E-07	0.000249	-8.29737
266	0.091	0.067	8.38E-07	0.000249	-8.29741
268	0.0925	0.0685	8.56E-07	0.000249	-8.29748

270	0.0935	0.0695	8.69E-07	0.000249	-8.29753
272	0.092	0.068	8.5E-07	0.000249	-8.29746
274	0.09425	0.07025	8.78E-07	0.000249	-8.29757
276	0.09325	0.06925	8.66E-07	0.000249	-8.29752
278	0.09425	0.07025	8.78E-07	0.000249	-8.29757
280	0.09475	0.07075	8.84E-07	0.000249	-8.29759
282	0.095	0.071	8.88E-07	0.000249	-8.29761
284	0.0965	0.0725	9.06E-07	0.000249	-8.29768
286	0.09725	0.07325	9.16E-07	0.000249	-8.29772
288	0.0985	0.0745	9.31E-07	0.000249	-8.29778
290	0.0995	0.0755	9.44E-07	0.000249	-8.29783
292	0.0995	0.0755	9.44E-07	0.000249	-8.29783
294	0.10025	0.07625	9.53E-07	0.000249	-8.29787
296	0.10075	0.07675	9.59E-07	0.000249	-8.29789
298	0.10275	0.07875	9.84E-07	0.000249	-8.29799

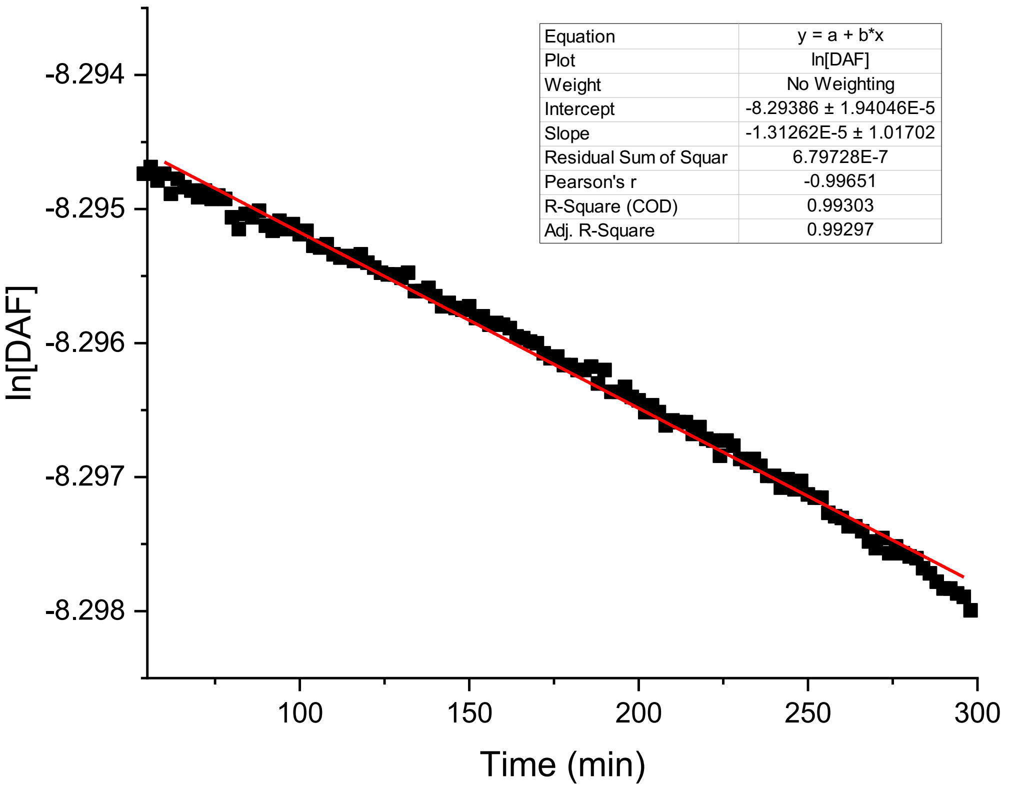

Catalysis of **DAF** to **FLU** in the presence of cage and **5 mM $NaHCO_3$** (0.25 mM **DAF**, 50 mM pH 7 **PBS** buffer, 0.1 mM **$CoH^{w}BF_4$**)

Time (min)	A@490nm	set to 0	[FLU] (M)	[DAF] (M)	ln[DAF]
0	0.0245	0	0	0.00025	-8.29405
2	0.03425	0.00975	1.22E-07	0.00025	-8.29454
4	0.03375	0.00925	1.16E-07	0.00025	-8.29451
6	0.03525	0.01075	1.34E-07	0.00025	-8.29459
8	0.0355	0.011	1.38E-07	0.00025	-8.2946
10	0.03625	0.01175	1.47E-07	0.00025	-8.29464
12	0.0365	0.012	1.5E-07	0.00025	-8.29465
14	0.0325	0.008	1E-07	0.00025	-8.29445
16	0.04075	0.01625	2.03E-07	0.00025	-8.29486
18	0.04075	0.01625	2.03E-07	0.00025	-8.29486
20	0.043	0.0185	2.31E-07	0.00025	-8.29498
22	0.04275	0.01825	2.28E-07	0.00025	-8.29496
24	0.0435	0.019	2.38E-07	0.00025	-8.295
26	0.04875	0.02425	3.03E-07	0.00025	-8.29526
28	0.04725	0.02275	2.84E-07	0.00025	-8.29519
30	0.04575	0.02125	2.66E-07	0.00025	-8.29511
32	0.05225	0.02775	3.47E-07	0.00025	-8.29544
34	0.05	0.0255	3.19E-07	0.00025	-8.29533
36	0.054	0.0295	3.69E-07	0.00025	-8.29553
38	0.0535	0.029	3.63E-07	0.00025	-8.2955
40	0.05575	0.03125	3.91E-07	0.00025	-8.29561
42	0.061	0.0365	4.56E-07	0.00025	-8.29588
44	0.06125	0.03675	4.59E-07	0.00025	-8.29589
46	0.0625	0.038	4.75E-07	0.00025	-8.29595
48	0.0635	0.039	4.88E-07	0.00025	-8.296
50	0.06625	0.04175	5.22E-07	0.000249	-8.29614
52	0.06825	0.04375	5.47E-07	0.000249	-8.29624
54	0.0725	0.048	6E-07	0.000249	-8.29645
56	0.074	0.0495	6.19E-07	0.000249	-8.29653
58	0.07575	0.05125	6.41E-07	0.000249	-8.29662
60	0.07625	0.05175	6.47E-07	0.000249	-8.29664
62	0.0805	0.056	7E-07	0.000249	-8.29685
64	0.08125	0.05675	7.09E-07	0.000249	-8.29689
66	0.08325	0.05875	7.34E-07	0.000249	-8.29699
68	0.08675	0.06225	7.78E-07	0.000249	-8.29717
70	0.09125	0.06675	8.34E-07	0.000249	-8.29739
72	0.09125	0.06675	8.34E-07	0.000249	-8.29739
74	0.09275	0.06825	8.53E-07	0.000249	-8.29747
76	0.09575	0.07125	8.91E-07	0.000249	-8.29762
78	0.10475	0.08025	1E-06	0.000249	-8.29807
80	0.10275	0.07825	9.78E-07	0.000249	-8.29797
82	0.1065	0.082	1.03E-06	0.000249	-8.29816
84	0.10875	0.08425	1.05E-06	0.000249	-8.29827

86	0.111	0.0865	1.08E-06	0.000249	-8.29838
88	0.114	0.0895	1.12E-06	0.000249	-8.29853
90	0.1175	0.093	1.16E-06	0.000249	-8.29871
92	0.1205	0.096	1.2E-06	0.000249	-8.29886
94	0.127	0.1025	1.28E-06	0.000249	-8.29919
96	0.1255	0.101	1.26E-06	0.000249	-8.29911
98	0.1285	0.104	1.3E-06	0.000249	-8.29926
100	0.1325	0.108	1.35E-06	0.000249	-8.29946
102	0.1335	0.109	1.36E-06	0.000249	-8.29951
104	0.13775	0.11325	1.42E-06	0.000249	-8.29973
106	0.1405	0.116	1.45E-06	0.000249	-8.29987
108	0.14225	0.11775	1.47E-06	0.000249	-8.29995
110	0.14625	0.12175	1.52E-06	0.000248	-8.30016
112	0.149	0.1245	1.56E-06	0.000248	-8.30029
114	0.153	0.1285	1.61E-06	0.000248	-8.3005
116	0.15475	0.13025	1.63E-06	0.000248	-8.30058
118	0.16075	0.13625	1.7E-06	0.000248	-8.30089
120	0.1595	0.135	1.69E-06	0.000248	-8.30082
122	0.1635	0.139	1.74E-06	0.000248	-8.30102
124	0.166	0.1415	1.77E-06	0.000248	-8.30115
126	0.16975	0.14525	1.82E-06	0.000248	-8.30134
128	0.171	0.1465	1.83E-06	0.000248	-8.3014
130	0.17675	0.15225	1.9E-06	0.000248	-8.30169
132	0.179	0.1545	1.93E-06	0.000248	-8.3018
134	0.1825	0.158	1.98E-06	0.000248	-8.30198
136	0.1865	0.162	2.03E-06	0.000248	-8.30218
138	0.18825	0.16375	2.05E-06	0.000248	-8.30227
140	0.19225	0.16775	2.1E-06	0.000248	-8.30247
142	0.19525	0.17075	2.13E-06	0.000248	-8.30262
144	0.1975	0.173	2.16E-06	0.000248	-8.30274
146	0.20175	0.17725	2.22E-06	0.000248	-8.30295
148	0.205	0.1805	2.26E-06	0.000248	-8.30312
150	0.208	0.1835	2.29E-06	0.000248	-8.30327
152	0.211	0.1865	2.33E-06	0.000248	-8.30342
154	0.2145	0.19	2.38E-06	0.000248	-8.3036
156	0.218	0.1935	2.42E-06	0.000248	-8.30377
158	0.2205	0.196	2.45E-06	0.000248	-8.3039
160	0.224	0.1995	2.49E-06	0.000248	-8.30407
162	0.227	0.2025	2.53E-06	0.000247	-8.30423
164	0.232	0.2075	2.59E-06	0.000247	-8.30448
166	0.2335	0.209	2.61E-06	0.000247	-8.30455
168	0.23725	0.21275	2.66E-06	0.000247	-8.30474
170	0.24025	0.21575	2.7E-06	0.000247	-8.3049
172	0.244	0.2195	2.74E-06	0.000247	-8.30509
174	0.24825	0.22375	2.8E-06	0.000247	-8.3053
176	0.25025	0.22575	2.82E-06	0.000247	-8.3054

178	0.25475	0.23025	2.88E-06	0.000247	-8.30563
180	0.25825	0.23375	2.92E-06	0.000247	-8.30581
182	0.261	0.2365	2.96E-06	0.000247	-8.30595
184	0.26325	0.23875	2.98E-06	0.000247	-8.30606
186	0.267	0.2425	3.03E-06	0.000247	-8.30625
188	0.2715	0.247	3.09E-06	0.000247	-8.30648
190	0.27325	0.24875	3.11E-06	0.000247	-8.30657
192	0.2785	0.254	3.18E-06	0.000247	-8.30683
194	0.2815	0.257	3.21E-06	0.000247	-8.30698
196	0.28425	0.25975	3.25E-06	0.000247	-8.30712
198	0.28775	0.26325	3.29E-06	0.000247	-8.3073
200	0.29075	0.26625	3.33E-06	0.000247	-8.30745
202	0.29525	0.27075	3.38E-06	0.000247	-8.30768
204	0.2975	0.273	3.41E-06	0.000247	-8.30779
206	0.3015	0.277	3.46E-06	0.000247	-8.308
208	0.30625	0.28175	3.52E-06	0.000246	-8.30824
210	0.307	0.2825	3.53E-06	0.000246	-8.30828
212	0.312	0.2875	3.59E-06	0.000246	-8.30853
214	0.31375	0.28925	3.62E-06	0.000246	-8.30862
216	0.31775	0.29325	3.67E-06	0.000246	-8.30882
218	0.32025	0.29575	3.7E-06	0.000246	-8.30895
220	0.325	0.3005	3.76E-06	0.000246	-8.30919
222	0.32775	0.30325	3.79E-06	0.000246	-8.30933
224	0.3315	0.307	3.84E-06	0.000246	-8.30952
226	0.3335	0.309	3.86E-06	0.000246	-8.30962
228	0.3365	0.312	3.9E-06	0.000246	-8.30977
230	0.3415	0.317	3.96E-06	0.000246	-8.31003
232	0.34375	0.31925	3.99E-06	0.000246	-8.31014
234	0.3485	0.324	4.05E-06	0.000246	-8.31038
236	0.35025	0.32575	4.07E-06	0.000246	-8.31047
238	0.35425	0.32975	4.12E-06	0.000246	-8.31067
240	0.35775	0.33325	4.17E-06	0.000246	-8.31085
242	0.3615	0.337	4.21E-06	0.000246	-8.31104
244	0.364	0.3395	4.24E-06	0.000246	-8.31117
246	0.36625	0.34175	4.27E-06	0.000246	-8.31128
248	0.369	0.3445	4.31E-06	0.000246	-8.31142
250	0.37275	0.34825	4.35E-06	0.000246	-8.31162
252	0.37775	0.35325	4.42E-06	0.000246	-8.31187
254	0.37925	0.35475	4.43E-06	0.000246	-8.31195
256	0.38275	0.35825	4.48E-06	0.000246	-8.31212
258	0.386	0.3615	4.52E-06	0.000245	-8.31229
260	0.3885	0.364	4.55E-06	0.000245	-8.31242
262	0.39325	0.36875	4.61E-06	0.000245	-8.31266
264	0.3945	0.37	4.63E-06	0.000245	-8.31272
266	0.3975	0.373	4.66E-06	0.000245	-8.31288
268	0.40275	0.37825	4.73E-06	0.000245	-8.31314

270	0.405	0.3805	4.76E-06	0.000245	-8.31326
272	0.408	0.3835	4.79E-06	0.000245	-8.31341
274	0.413	0.3885	4.86E-06	0.000245	-8.31367
276	0.41375	0.38925	4.87E-06	0.000245	-8.3137
278	0.417	0.3925	4.91E-06	0.000245	-8.31387
280	0.419	0.3945	4.93E-06	0.000245	-8.31397
282	0.42175	0.39725	4.97E-06	0.000245	-8.31411
284	0.426	0.4015	5.02E-06	0.000245	-8.31433
286	0.4295	0.405	5.06E-06	0.000245	-8.31451
288	0.43275	0.40825	5.1E-06	0.000245	-8.31467
290	0.43675	0.41225	5.15E-06	0.000245	-8.31488
292	0.43775	0.41325	5.17E-06	0.000245	-8.31493
294	0.44125	0.41675	5.21E-06	0.000245	-8.31511
296	0.4445	0.42	5.25E-06	0.000245	-8.31527
298	0.4495	0.425	5.31E-06	0.000245	-8.31553

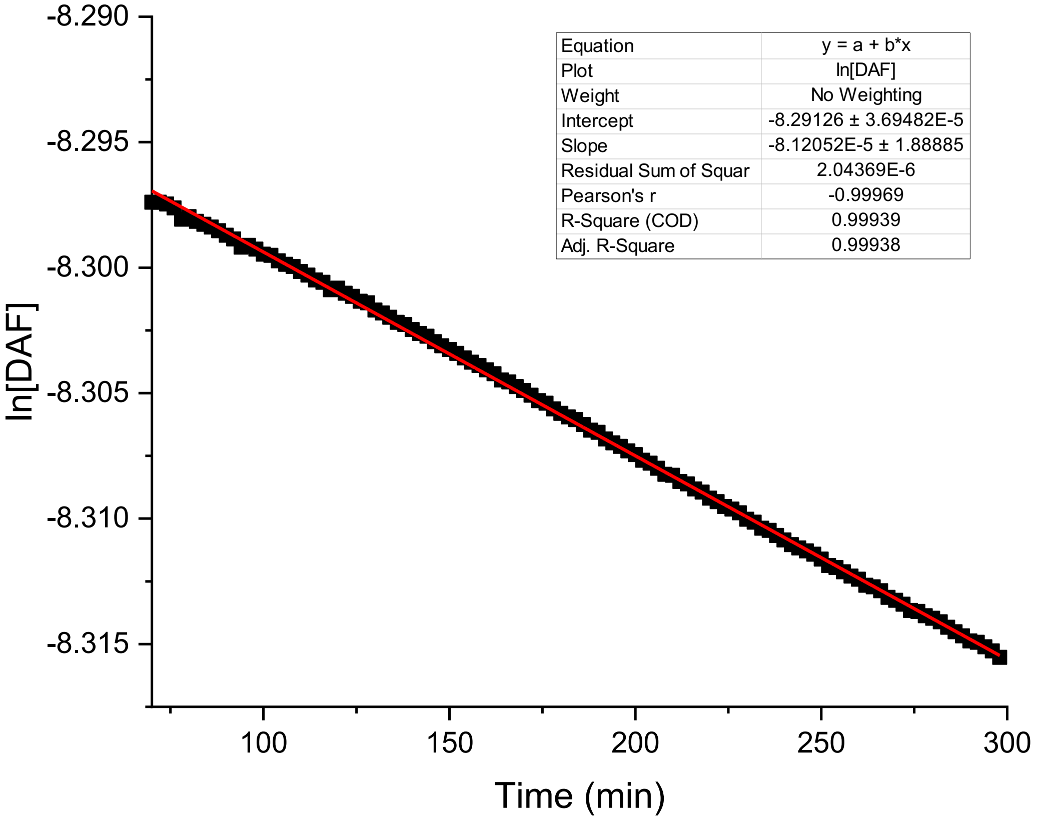

Catalysis of **DAF** to **FLU** in the presence of cage and **5 mM Na_2SO_4** (0.25 mM **DAF**, 50 mM pH 7 **PBS** buffer, 0.1 mM **$CoH^{w}BF_4$**)

Time (min)	A@490nm	set to 0	[FLU] (M)	[DAF] (M)	ln[DAF]
0	0.0185	0	0	0.00025	-8.29405
2	0.0265	0.008	1E-07	0.00025	-8.29445
4	0.031	0.0125	1.56E-07	0.00025	-8.29467
6	0.02975	0.01125	1.41E-07	0.00025	-8.29461
8	0.031	0.0125	1.56E-07	0.00025	-8.29467
10	0.03175	0.01325	1.66E-07	0.00025	-8.29471
12	0.0325	0.014	1.75E-07	0.00025	-8.29475
14	0.0315	0.013	1.63E-07	0.00025	-8.2947
16	0.036	0.0175	2.19E-07	0.00025	-8.29493
18	0.0365	0.018	2.25E-07	0.00025	-8.29495
20	0.038	0.0195	2.44E-07	0.00025	-8.29503
22	0.0385	0.02	2.5E-07	0.00025	-8.29505
24	0.041	0.0225	2.81E-07	0.00025	-8.29518
26	0.04175	0.02325	2.91E-07	0.00025	-8.29521
28	0.043	0.0245	3.06E-07	0.00025	-8.29528
30	0.04225	0.02375	2.97E-07	0.00025	-8.29524
32	0.0435	0.025	3.13E-07	0.00025	-8.2953
34	0.04475	0.02625	3.28E-07	0.00025	-8.29536
36	0.04775	0.02925	3.66E-07	0.00025	-8.29551
38	0.048	0.0295	3.69E-07	0.00025	-8.29553
40	0.04925	0.03075	3.84E-07	0.00025	-8.29559
42	0.0525	0.034	4.25E-07	0.00025	-8.29575
44	0.053	0.0345	4.31E-07	0.00025	-8.29578
46	0.0555	0.037	4.63E-07	0.00025	-8.2959
48	0.05725	0.03875	4.84E-07	0.00025	-8.29599
50	0.05725	0.03875	4.84E-07	0.00025	-8.29599
52	0.06825	0.04975	6.22E-07	0.000249	-8.29654
54	0.0625	0.044	5.5E-07	0.000249	-8.29625
56	0.0625	0.044	5.5E-07	0.000249	-8.29625
58	0.065	0.0465	5.81E-07	0.000249	-8.29638
60	0.0655	0.047	5.88E-07	0.000249	-8.2964
62	0.06825	0.04975	6.22E-07	0.000249	-8.29654
64	0.06925	0.05075	6.34E-07	0.000249	-8.29659
66	0.071	0.0525	6.56E-07	0.000249	-8.29668
68	0.075	0.0565	7.06E-07	0.000249	-8.29688
70	0.08125	0.06275	7.84E-07	0.000249	-8.29719
72	0.077	0.0585	7.31E-07	0.000249	-8.29698
74	0.0785	0.06	7.5E-07	0.000249	-8.29705
76	0.08075	0.06225	7.78E-07	0.000249	-8.29717
78	0.0825	0.064	8E-07	0.000249	-8.29725
80	0.0875	0.069	8.63E-07	0.000249	-8.29751
82	0.0895	0.071	8.88E-07	0.000249	-8.29761
84	0.08975	0.07125	8.91E-07	0.000249	-8.29762

86	0.092	0.0735	9.19E-07	0.000249	-8.29773
88	0.09425	0.07575	9.47E-07	0.000249	-8.29784
90	0.09625	0.07775	9.72E-07	0.000249	-8.29794
92	0.1	0.0815	1.02E-06	0.000249	-8.29813
94	0.09925	0.08075	1.01E-06	0.000249	-8.2981
96	0.10075	0.08225	1.03E-06	0.000249	-8.29817
98	0.10275	0.08425	1.05E-06	0.000249	-8.29827
100	0.10675	0.08825	1.1E-06	0.000249	-8.29847
102	0.107	0.0885	1.11E-06	0.000249	-8.29848
104	0.109	0.0905	1.13E-06	0.000249	-8.29858
106	0.112	0.0935	1.17E-06	0.000249	-8.29874
108	0.11275	0.09425	1.18E-06	0.000249	-8.29877
110	0.11775	0.09925	1.24E-06	0.000249	-8.29902
112	0.11775	0.09925	1.24E-06	0.000249	-8.29902
114	0.12125	0.10275	1.28E-06	0.000249	-8.2992
116	0.12275	0.10425	1.3E-06	0.000249	-8.29928
118	0.12375	0.10525	1.32E-06	0.000249	-8.29933
120	0.1275	0.109	1.36E-06	0.000249	-8.29951
122	0.12875	0.11025	1.38E-06	0.000249	-8.29958
124	0.13125	0.11275	1.41E-06	0.000249	-8.2997
126	0.133	0.1145	1.43E-06	0.000249	-8.29979
128	0.1345	0.116	1.45E-06	0.000249	-8.29987
130	0.13725	0.11875	1.48E-06	0.000249	-8.3
132	0.14025	0.12175	1.52E-06	0.000248	-8.30016
134	0.14325	0.12475	1.56E-06	0.000248	-8.30031
136	0.146	0.1275	1.59E-06	0.000248	-8.30045
138	0.14675	0.12825	1.6E-06	0.000248	-8.30048
140	0.1495	0.131	1.64E-06	0.000248	-8.30062
142	0.1525	0.134	1.68E-06	0.000248	-8.30077
144	0.15575	0.13725	1.72E-06	0.000248	-8.30094
146	0.15675	0.13825	1.73E-06	0.000248	-8.30099
148	0.15925	0.14075	1.76E-06	0.000248	-8.30111
150	0.162	0.1435	1.79E-06	0.000248	-8.30125
152	0.16575	0.14725	1.84E-06	0.000248	-8.30144
154	0.16875	0.15025	1.88E-06	0.000248	-8.30159
156	0.16975	0.15125	1.89E-06	0.000248	-8.30164
158	0.17	0.1515	1.89E-06	0.000248	-8.30165
160	0.17425	0.15575	1.95E-06	0.000248	-8.30187
162	0.1765	0.158	1.98E-06	0.000248	-8.30198
164	0.17875	0.16025	2E-06	0.000248	-8.30209
166	0.181	0.1625	2.03E-06	0.000248	-8.30221
168	0.18275	0.16425	2.05E-06	0.000248	-8.3023
170	0.186	0.1675	2.09E-06	0.000248	-8.30246
172	0.18925	0.17075	2.13E-06	0.000248	-8.30262
174	0.19325	0.17475	2.18E-06	0.000248	-8.30283
176	0.19375	0.17525	2.19E-06	0.000248	-8.30285

178	0.19525	0.17675	2.21E-06	0.000248	-8.30293
180	0.19825	0.17975	2.25E-06	0.000248	-8.30308
182	0.20025	0.18175	2.27E-06	0.000248	-8.30318
184	0.203	0.1845	2.31E-06	0.000248	-8.30332
186	0.20525	0.18675	2.33E-06	0.000248	-8.30343
188	0.208	0.1895	2.37E-06	0.000248	-8.30357
190	0.209	0.1905	2.38E-06	0.000248	-8.30362
192	0.2145	0.196	2.45E-06	0.000248	-8.3039
194	0.21475	0.19625	2.45E-06	0.000248	-8.30391
196	0.2165	0.198	2.48E-06	0.000248	-8.304
198	0.22	0.2015	2.52E-06	0.000247	-8.30418
200	0.222	0.2035	2.54E-06	0.000247	-8.30428
202	0.22575	0.20725	2.59E-06	0.000247	-8.30447
204	0.2275	0.209	2.61E-06	0.000247	-8.30455
206	0.229	0.2105	2.63E-06	0.000247	-8.30463
208	0.23275	0.21425	2.68E-06	0.000247	-8.30482
210	0.23475	0.21625	2.7E-06	0.000247	-8.30492
212	0.2395	0.221	2.76E-06	0.000247	-8.30516
214	0.2385	0.22	2.75E-06	0.000247	-8.30511
216	0.2425	0.224	2.8E-06	0.000247	-8.30531
218	0.244	0.2255	2.82E-06	0.000247	-8.30539
220	0.248	0.2295	2.87E-06	0.000247	-8.30559
222	0.2495	0.231	2.89E-06	0.000247	-8.30567
224	0.25325	0.23475	2.93E-06	0.000247	-8.30586
226	0.2535	0.235	2.94E-06	0.000247	-8.30587
228	0.25525	0.23675	2.96E-06	0.000247	-8.30596
230	0.2595	0.241	3.01E-06	0.000247	-8.30617
232	0.262	0.2435	3.04E-06	0.000247	-8.3063
234	0.26475	0.24625	3.08E-06	0.000247	-8.30644
236	0.26625	0.24775	3.1E-06	0.000247	-8.30651
238	0.26975	0.25125	3.14E-06	0.000247	-8.30669
240	0.27225	0.25375	3.17E-06	0.000247	-8.30682
242	0.27525	0.25675	3.21E-06	0.000247	-8.30697
244	0.27575	0.25725	3.22E-06	0.000247	-8.307
246	0.27825	0.25975	3.25E-06	0.000247	-8.30712
248	0.28025	0.26175	3.27E-06	0.000247	-8.30722
250	0.284	0.2655	3.32E-06	0.000247	-8.30741
252	0.2865	0.268	3.35E-06	0.000247	-8.30754
254	0.28775	0.26925	3.37E-06	0.000247	-8.3076
256	0.29	0.2715	3.39E-06	0.000247	-8.30772
258	0.29325	0.27475	3.43E-06	0.000247	-8.30788
260	0.29525	0.27675	3.46E-06	0.000247	-8.30798
262	0.3	0.2815	3.52E-06	0.000246	-8.30822
264	0.3	0.2815	3.52E-06	0.000246	-8.30822
266	0.30325	0.28475	3.56E-06	0.000246	-8.30839
268	0.30725	0.28875	3.61E-06	0.000246	-8.30859

270	0.309	0.2905	3.63E-06	0.000246	-8.30868
272	0.31025	0.29175	3.65E-06	0.000246	-8.30874
274	0.3145	0.296	3.7E-06	0.000246	-8.30896
276	0.3145	0.296	3.7E-06	0.000246	-8.30896
278	0.31725	0.29875	3.73E-06	0.000246	-8.3091
280	0.319	0.3005	3.76E-06	0.000246	-8.30919
282	0.3225	0.304	3.8E-06	0.000246	-8.30937
284	0.32525	0.30675	3.83E-06	0.000246	-8.30951
286	0.3275	0.309	3.86E-06	0.000246	-8.30962
288	0.33	0.3115	3.89E-06	0.000246	-8.30975
290	0.3335	0.315	3.94E-06	0.000246	-8.30992
292	0.33475	0.31625	3.95E-06	0.000246	-8.30999
294	0.3375	0.319	3.99E-06	0.000246	-8.31013
296	0.33925	0.32075	4.01E-06	0.000246	-8.31022
298	0.34325	0.32475	4.06E-06	0.000246	-8.31042

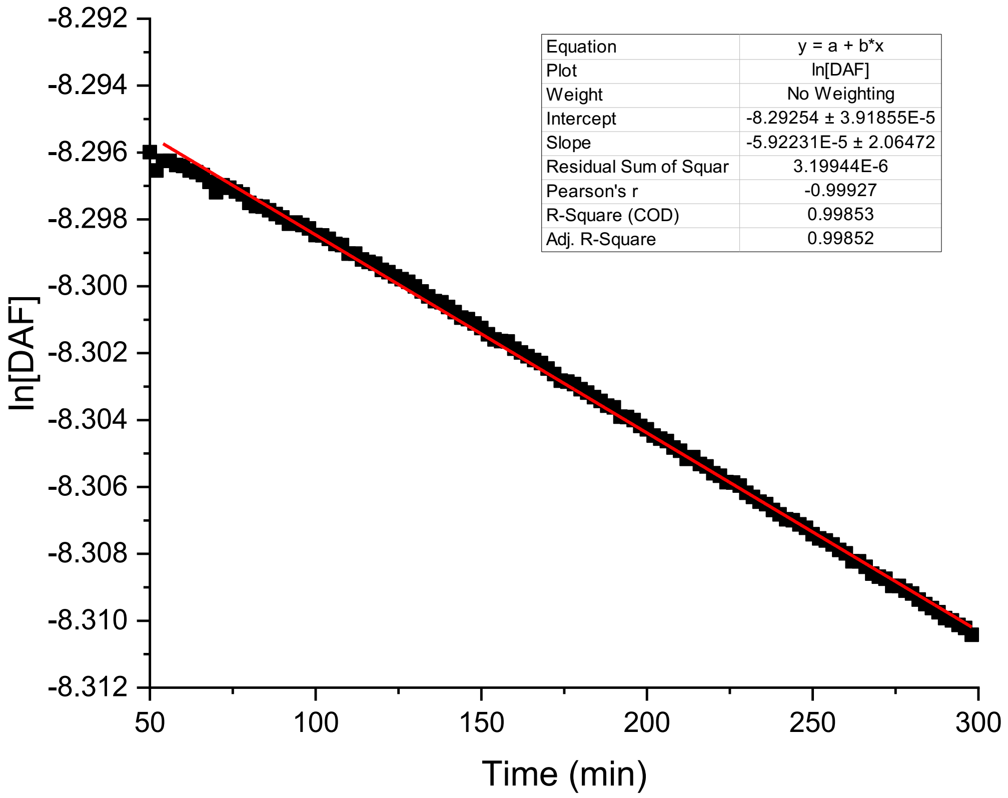

Catalysis of **DAF** to **FLU** in the presence of cage and **5 mM NaCl** (0.25 mM **DAF**, 50 mM pH 7 **PBS** buffer, 0.1 mM **CoH^w^BF$_4$**)

Time (min)	A@490nm	set to 0	[FLU] (M)	[DAF] (M)	ln[DAF]
0	0.02575	0	0	0.00025	-8.29405
2	0.03425	0.0085	1.06E-07	0.00025	-8.29447
4	0.0375	0.01175	1.47E-07	0.00025	-8.29464
6	0.0355	0.00975	1.22E-07	0.00025	-8.29454
8	0.036	0.01025	1.28E-07	0.00025	-8.29456
10	0.0365	0.01075	1.34E-07	0.00025	-8.29459
12	0.0375	0.01175	1.47E-07	0.00025	-8.29464
14	0.033	0.00725	9.06E-08	0.00025	-8.29441
16	0.03825	0.0125	1.56E-07	0.00025	-8.29467
18	0.03925	0.0135	1.69E-07	0.00025	-8.29472
20	0.043	0.01725	2.16E-07	0.00025	-8.29491
22	0.04425	0.0185	2.31E-07	0.00025	-8.29498
24	0.0415	0.01575	1.97E-07	0.00025	-8.29484
26	0.04375	0.018	2.25E-07	0.00025	-8.29495
28	0.0425	0.01675	2.09E-07	0.00025	-8.29489
30	0.042	0.01625	2.03E-07	0.00025	-8.29486
32	0.04625	0.0205	2.56E-07	0.00025	-8.29508
34	0.04375	0.018	2.25E-07	0.00025	-8.29495
36	0.0485	0.02275	2.84E-07	0.00025	-8.29519
38	0.049	0.02325	2.91E-07	0.00025	-8.29521
40	0.04875	0.023	2.88E-07	0.00025	-8.2952
42	0.04875	0.023	2.88E-07	0.00025	-8.2952
44	0.0515	0.02575	3.22E-07	0.00025	-8.29534
46	0.0535	0.02775	3.47E-07	0.00025	-8.29544
48	0.05425	0.0285	3.56E-07	0.00025	-8.29548
50	0.054	0.02825	3.53E-07	0.00025	-8.29546
52	0.056	0.03025	3.78E-07	0.00025	-8.29556
54	0.0575	0.03175	3.97E-07	0.00025	-8.29564
56	0.0595	0.03375	4.22E-07	0.00025	-8.29574
58	0.0615	0.03575	4.47E-07	0.00025	-8.29584
60	0.06025	0.0345	4.31E-07	0.00025	-8.29578
62	0.06125	0.0355	4.44E-07	0.00025	-8.29583
64	0.0625	0.03675	4.59E-07	0.00025	-8.29589
66	0.06425	0.0385	4.81E-07	0.00025	-8.29598
68	0.068	0.04225	5.28E-07	0.000249	-8.29616
70	0.0685	0.04275	5.34E-07	0.000249	-8.29619
72	0.06875	0.043	5.38E-07	0.000249	-8.2962
74	0.07025	0.0445	5.56E-07	0.000249	-8.29628
76	0.0715	0.04575	5.72E-07	0.000249	-8.29634
78	0.07275	0.047	5.88E-07	0.000249	-8.2964
80	0.0755	0.04975	6.22E-07	0.000249	-8.29654
82	0.07825	0.0525	6.56E-07	0.000249	-8.29668
84	0.07975	0.054	6.75E-07	0.000249	-8.29675

86	0.0805	0.05475	6.84E-07	0.000249	-8.29679
88	0.0815	0.05575	6.97E-07	0.000249	-8.29684
90	0.08325	0.0575	7.19E-07	0.000249	-8.29693
92	0.08475	0.059	7.38E-07	0.000249	-8.297
94	0.0845	0.05875	7.34E-07	0.000249	-8.29699
96	0.087	0.06125	7.66E-07	0.000249	-8.29712
98	0.08825	0.0625	7.81E-07	0.000249	-8.29718
100	0.09025	0.0645	8.06E-07	0.000249	-8.29728
102	0.09125	0.0655	8.19E-07	0.000249	-8.29733
104	0.0925	0.06675	8.34E-07	0.000249	-8.29739
106	0.09575	0.07	8.75E-07	0.000249	-8.29756
108	0.09575	0.07	8.75E-07	0.000249	-8.29756
110	0.1005	0.07475	9.34E-07	0.000249	-8.29779
112	0.09875	0.073	9.13E-07	0.000249	-8.29771
114	0.10125	0.0755	9.44E-07	0.000249	-8.29783
116	0.1035	0.07775	9.72E-07	0.000249	-8.29794
118	0.103	0.07725	9.66E-07	0.000249	-8.29792
120	0.10625	0.0805	1.01E-06	0.000249	-8.29808
122	0.10775	0.082	1.03E-06	0.000249	-8.29816
124	0.1105	0.08475	1.06E-06	0.000249	-8.2983
126	0.1115	0.08575	1.07E-06	0.000249	-8.29835
128	0.1135	0.08775	1.1E-06	0.000249	-8.29845
130	0.1145	0.08875	1.11E-06	0.000249	-8.2985
132	0.1165	0.09075	1.13E-06	0.000249	-8.2986
134	0.1195	0.09375	1.17E-06	0.000249	-8.29875
136	0.12075	0.095	1.19E-06	0.000249	-8.29881
138	0.1215	0.09575	1.2E-06	0.000249	-8.29885
140	0.1245	0.09875	1.23E-06	0.000249	-8.299
142	0.12725	0.1015	1.27E-06	0.000249	-8.29914
144	0.12775	0.102	1.28E-06	0.000249	-8.29916
146	0.1295	0.10375	1.3E-06	0.000249	-8.29925
148	0.1325	0.10675	1.33E-06	0.000249	-8.2994
150	0.132	0.10625	1.33E-06	0.000249	-8.29938
152	0.13525	0.1095	1.37E-06	0.000249	-8.29954
154	0.13625	0.1105	1.38E-06	0.000249	-8.29959
156	0.14075	0.115	1.44E-06	0.000249	-8.29982
158	0.1405	0.11475	1.43E-06	0.000249	-8.2998
160	0.1415	0.11575	1.45E-06	0.000249	-8.29985
162	0.14275	0.117	1.46E-06	0.000249	-8.29992
164	0.1465	0.12075	1.51E-06	0.000248	-8.30011
166	0.14925	0.1235	1.54E-06	0.000248	-8.30024
168	0.1495	0.12375	1.55E-06	0.000248	-8.30026
170	0.15175	0.126	1.58E-06	0.000248	-8.30037
172	0.155	0.12925	1.62E-06	0.000248	-8.30053
174	0.15725	0.1315	1.64E-06	0.000248	-8.30065
176	0.15775	0.132	1.65E-06	0.000248	-8.30067

178	0.159	0.13325	1.67E-06	0.000248	-8.30073
180	0.1615	0.13575	1.7E-06	0.000248	-8.30086
182	0.16325	0.1375	1.72E-06	0.000248	-8.30095
184	0.1655	0.13975	1.75E-06	0.000248	-8.30106
186	0.1665	0.14075	1.76E-06	0.000248	-8.30111
188	0.17025	0.1445	1.81E-06	0.000248	-8.3013
190	0.16975	0.144	1.8E-06	0.000248	-8.30128
192	0.1735	0.14775	1.85E-06	0.000248	-8.30146
194	0.1765	0.15075	1.88E-06	0.000248	-8.30162
196	0.176	0.15025	1.88E-06	0.000248	-8.30159
198	0.17825	0.1525	1.91E-06	0.000248	-8.3017
200	0.18025	0.1545	1.93E-06	0.000248	-8.3018
202	0.183	0.15725	1.97E-06	0.000248	-8.30194
204	0.184	0.15825	1.98E-06	0.000248	-8.30199
206	0.186	0.16025	2E-06	0.000248	-8.30209
208	0.18875	0.163	2.04E-06	0.000248	-8.30223
210	0.18925	0.1635	2.04E-06	0.000248	-8.30226
212	0.19275	0.167	2.09E-06	0.000248	-8.30243
214	0.1945	0.16875	2.11E-06	0.000248	-8.30252
216	0.19675	0.171	2.14E-06	0.000248	-8.30264
218	0.197	0.17125	2.14E-06	0.000248	-8.30265
220	0.1995	0.17375	2.17E-06	0.000248	-8.30278
222	0.20275	0.177	2.21E-06	0.000248	-8.30294
224	0.20475	0.179	2.24E-06	0.000248	-8.30304
226	0.20525	0.1795	2.24E-06	0.000248	-8.30307
228	0.20725	0.1815	2.27E-06	0.000248	-8.30317
230	0.21075	0.185	2.31E-06	0.000248	-8.30334
232	0.2125	0.18675	2.33E-06	0.000248	-8.30343
234	0.2145	0.18875	2.36E-06	0.000248	-8.30353
236	0.21625	0.1905	2.38E-06	0.000248	-8.30362
238	0.2195	0.19375	2.42E-06	0.000248	-8.30378
240	0.21925	0.1935	2.42E-06	0.000248	-8.30377
242	0.22175	0.196	2.45E-06	0.000248	-8.3039
244	0.22325	0.1975	2.47E-06	0.000248	-8.30397
246	0.22425	0.1985	2.48E-06	0.000248	-8.30402
248	0.2265	0.20075	2.51E-06	0.000247	-8.30414
250	0.229	0.20325	2.54E-06	0.000247	-8.30426
252	0.23075	0.205	2.56E-06	0.000247	-8.30435
254	0.23275	0.207	2.59E-06	0.000247	-8.30445
256	0.23575	0.21	2.63E-06	0.000247	-8.30461
258	0.23825	0.2125	2.66E-06	0.000247	-8.30473
260	0.2395	0.21375	2.67E-06	0.000247	-8.30479
262	0.24175	0.216	2.7E-06	0.000247	-8.30491
264	0.24325	0.2175	2.72E-06	0.000247	-8.30498
266	0.245	0.21925	2.74E-06	0.000247	-8.30507
268	0.2485	0.22275	2.78E-06	0.000247	-8.30525

270	0.2495	0.22375	2.8E-06	0.000247	-8.3053
272	0.25175	0.226	2.83E-06	0.000247	-8.30541
274	0.2545	0.22875	2.86E-06	0.000247	-8.30555
276	0.2545	0.22875	2.86E-06	0.000247	-8.30555
278	0.257	0.23125	2.89E-06	0.000247	-8.30568
280	0.25975	0.234	2.93E-06	0.000247	-8.30582
282	0.26225	0.2365	2.96E-06	0.000247	-8.30595
284	0.26275	0.237	2.96E-06	0.000247	-8.30597
286	0.265	0.23925	2.99E-06	0.000247	-8.30608
288	0.267	0.24125	3.02E-06	0.000247	-8.30619
290	0.27125	0.2455	3.07E-06	0.000247	-8.3064
292	0.272	0.24625	3.08E-06	0.000247	-8.30644
294	0.27325	0.2475	3.09E-06	0.000247	-8.3065
296	0.27625	0.2505	3.13E-06	0.000247	-8.30665
298	0.28	0.25425	3.18E-06	0.000247	-8.30684

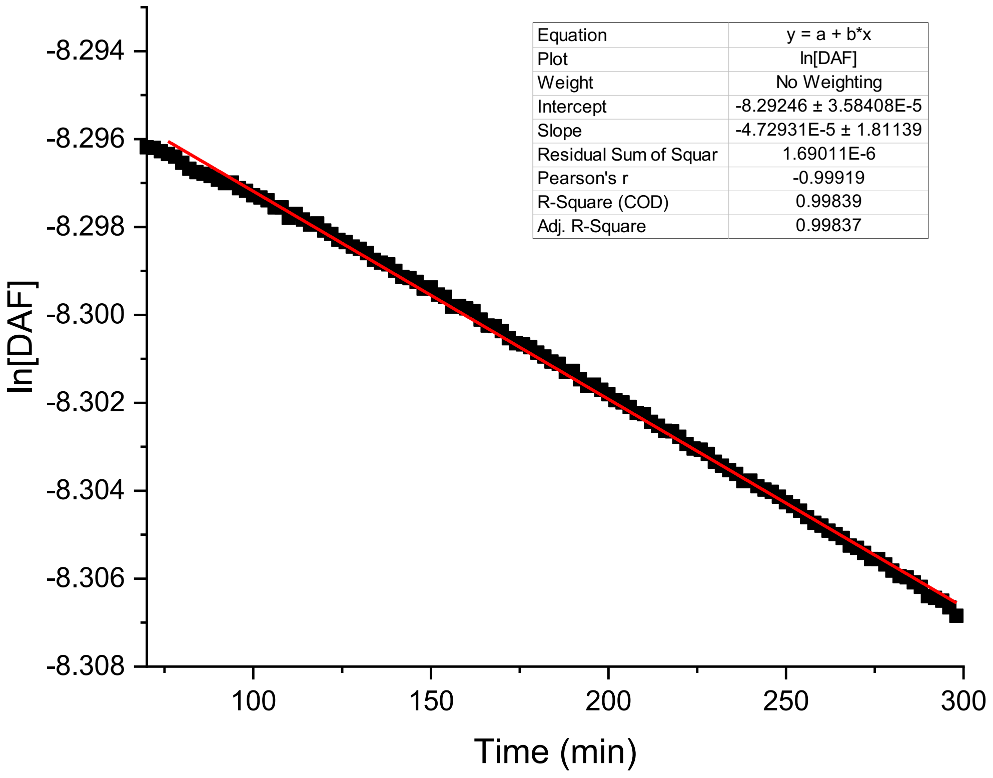

Catalysis of **DAF** to **FLU** in the presence of cage and **5 mM $NaIO_3$** (0.25 mM **DAF**, 50 mM pH 7 **PBS** buffer, 0.1 mM **$CoH^{w}BF_4$**)

Time (min)	A@490nm	set to 0	[FLU] (M)	[DAF] (M)	ln[DAF]
0	0.02125	0	0	0.00025	-8.29405
2	0.0265	0.00525	6.56E-08	0.00025	-8.29431
4	0.02825	0.007	8.75E-08	0.00025	-8.2944
6	0.02925	0.008	1E-07	0.00025	-8.29445
8	0.02775	0.0065	8.13E-08	0.00025	-8.29437
10	0.0315	0.01025	1.28E-07	0.00025	-8.29456
12	0.032	0.01075	1.34E-07	0.00025	-8.29459
14	0.029	0.00775	9.69E-08	0.00025	-8.29444
16	0.03475	0.0135	1.69E-07	0.00025	-8.29472
18	0.037	0.01575	1.97E-07	0.00025	-8.29484
20	0.03725	0.016	2E-07	0.00025	-8.29485
22	0.04	0.01875	2.34E-07	0.00025	-8.29499
24	0.03975	0.0185	2.31E-07	0.00025	-8.29498
26	0.041	0.01975	2.47E-07	0.00025	-8.29504
28	0.04275	0.0215	2.69E-07	0.00025	-8.29513
30	0.043	0.02175	2.72E-07	0.00025	-8.29514
32	0.04375	0.0225	2.81E-07	0.00025	-8.29518
34	0.04475	0.0235	2.94E-07	0.00025	-8.29523
36	0.0485	0.02725	3.41E-07	0.00025	-8.29541
38	0.05075	0.0295	3.69E-07	0.00025	-8.29553
40	0.0485	0.02725	3.41E-07	0.00025	-8.29541
42	0.05125	0.03	3.75E-07	0.00025	-8.29555
44	0.0585	0.03725	4.66E-07	0.00025	-8.29591
46	0.05775	0.0365	4.56E-07	0.00025	-8.29588
48	0.059	0.03775	4.72E-07	0.00025	-8.29594
50	0.0585	0.03725	4.66E-07	0.00025	-8.29591
52	0.063	0.04175	5.22E-07	0.000249	-8.29614
54	0.06275	0.0415	5.19E-07	0.000249	-8.29613
56	0.06325	0.042	5.25E-07	0.000249	-8.29615
58	0.07275	0.0515	6.44E-07	0.000249	-8.29663
60	0.068	0.04675	5.84E-07	0.000249	-8.29639
62	0.06975	0.0485	6.06E-07	0.000249	-8.29648
64	0.07275	0.0515	6.44E-07	0.000249	-8.29663
66	0.07575	0.0545	6.81E-07	0.000249	-8.29678
68	0.07725	0.056	7E-07	0.000249	-8.29685
70	0.08275	0.0615	7.69E-07	0.000249	-8.29713
72	0.08175	0.0605	7.56E-07	0.000249	-8.29708
74	0.08275	0.0615	7.69E-07	0.000249	-8.29713
76	0.08475	0.0635	7.94E-07	0.000249	-8.29723
78	0.08775	0.0665	8.31E-07	0.000249	-8.29738
80	0.09	0.06875	8.59E-07	0.000249	-8.29749
82	0.09475	0.0735	9.19E-07	0.000249	-8.29773
84	0.09425	0.073	9.13E-07	0.000249	-8.29771

86	0.097	0.07575	9.47E-07	0.000249	-8.29784
88	0.0995	0.07825	9.78E-07	0.000249	-8.29797
90	0.10175	0.0805	1.01E-06	0.000249	-8.29808
92	0.10475	0.0835	1.04E-06	0.000249	-8.29823
94	0.106	0.08475	1.06E-06	0.000249	-8.2983
96	0.109	0.08775	1.1E-06	0.000249	-8.29845
98	0.1105	0.08925	1.12E-06	0.000249	-8.29852
100	0.11275	0.0915	1.14E-06	0.000249	-8.29864
102	0.1145	0.09325	1.17E-06	0.000249	-8.29872
104	0.11925	0.098	1.23E-06	0.000249	-8.29896
106	0.11975	0.0985	1.23E-06	0.000249	-8.29899
108	0.11975	0.0985	1.23E-06	0.000249	-8.29899
110	0.12425	0.103	1.29E-06	0.000249	-8.29921
112	0.126	0.10475	1.31E-06	0.000249	-8.2993
114	0.128	0.10675	1.33E-06	0.000249	-8.2994
116	0.13	0.10875	1.36E-06	0.000249	-8.2995
118	0.13325	0.112	1.4E-06	0.000249	-8.29967
120	0.135	0.11375	1.42E-06	0.000249	-8.29975
122	0.1375	0.11625	1.45E-06	0.000249	-8.29988
124	0.14025	0.119	1.49E-06	0.000249	-8.30002
126	0.14225	0.121	1.51E-06	0.000248	-8.30012
128	0.14425	0.123	1.54E-06	0.000248	-8.30022
130	0.1465	0.12525	1.57E-06	0.000248	-8.30033
132	0.14875	0.1275	1.59E-06	0.000248	-8.30045
134	0.15225	0.131	1.64E-06	0.000248	-8.30062
136	0.15525	0.134	1.68E-06	0.000248	-8.30077
138	0.15525	0.134	1.68E-06	0.000248	-8.30077
140	0.159	0.13775	1.72E-06	0.000248	-8.30096
142	0.163	0.14175	1.77E-06	0.000248	-8.30116
144	0.1665	0.14525	1.82E-06	0.000248	-8.30134
146	0.16525	0.144	1.8E-06	0.000248	-8.30128
148	0.17025	0.149	1.86E-06	0.000248	-8.30153
150	0.1705	0.14925	1.87E-06	0.000248	-8.30154
152	0.17425	0.153	1.91E-06	0.000248	-8.30173
154	0.176	0.15475	1.93E-06	0.000248	-8.30182
156	0.17975	0.1585	1.98E-06	0.000248	-8.30201
158	0.17975	0.1585	1.98E-06	0.000248	-8.30201
160	0.1845	0.16325	2.04E-06	0.000248	-8.30225
162	0.1845	0.16325	2.04E-06	0.000248	-8.30225
164	0.1885	0.16725	2.09E-06	0.000248	-8.30245
166	0.1895	0.16825	2.1E-06	0.000248	-8.3025
168	0.1925	0.17125	2.14E-06	0.000248	-8.30265
170	0.19625	0.175	2.19E-06	0.000248	-8.30284
172	0.2025	0.18125	2.27E-06	0.000248	-8.30315
174	0.204	0.18275	2.28E-06	0.000248	-8.30323
176	0.20525	0.184	2.3E-06	0.000248	-8.30329

178	0.20725	0.186	2.33E-06	0.000248	-8.30339
180	0.211	0.18975	2.37E-06	0.000248	-8.30358
182	0.2135	0.19225	2.4E-06	0.000248	-8.30371
184	0.21525	0.194	2.43E-06	0.000248	-8.3038
186	0.21775	0.1965	2.46E-06	0.000248	-8.30392
188	0.22075	0.1995	2.49E-06	0.000248	-8.30407
190	0.221	0.19975	2.5E-06	0.000248	-8.30409
192	0.226	0.20475	2.56E-06	0.000247	-8.30434
194	0.2275	0.20625	2.58E-06	0.000247	-8.30442
196	0.22875	0.2075	2.59E-06	0.000247	-8.30448
198	0.23175	0.2105	2.63E-06	0.000247	-8.30463
200	0.2345	0.21325	2.67E-06	0.000247	-8.30477
202	0.2375	0.21625	2.7E-06	0.000247	-8.30492
204	0.238	0.21675	2.71E-06	0.000247	-8.30495
206	0.24075	0.2195	2.74E-06	0.000247	-8.30509
208	0.24375	0.2225	2.78E-06	0.000247	-8.30524
210	0.24525	0.224	2.8E-06	0.000247	-8.30531
212	0.24775	0.2265	2.83E-06	0.000247	-8.30544
214	0.25	0.22875	2.86E-06	0.000247	-8.30555
216	0.2525	0.23125	2.89E-06	0.000247	-8.30568
218	0.2545	0.23325	2.92E-06	0.000247	-8.30578
220	0.258	0.23675	2.96E-06	0.000247	-8.30596
222	0.26075	0.2395	2.99E-06	0.000247	-8.3061
224	0.26325	0.242	3.03E-06	0.000247	-8.30622
226	0.2645	0.24325	3.04E-06	0.000247	-8.30629
228	0.26625	0.245	3.06E-06	0.000247	-8.30638
230	0.27025	0.249	3.11E-06	0.000247	-8.30658
232	0.27275	0.2515	3.14E-06	0.000247	-8.3067
234	0.2755	0.25425	3.18E-06	0.000247	-8.30684
236	0.27775	0.2565	3.21E-06	0.000247	-8.30696
238	0.28075	0.2595	3.24E-06	0.000247	-8.30711
240	0.282	0.26075	3.26E-06	0.000247	-8.30717
242	0.2855	0.26425	3.3E-06	0.000247	-8.30735
244	0.28675	0.2655	3.32E-06	0.000247	-8.30741
246	0.2885	0.26725	3.34E-06	0.000247	-8.3075
248	0.291	0.26975	3.37E-06	0.000247	-8.30763
250	0.29475	0.2735	3.42E-06	0.000247	-8.30782
252	0.29625	0.275	3.44E-06	0.000247	-8.3079
254	0.29875	0.2775	3.47E-06	0.000247	-8.30802
256	0.3015	0.28025	3.5E-06	0.000246	-8.30816
258	0.3045	0.28325	3.54E-06	0.000246	-8.30831
260	0.30675	0.2855	3.57E-06	0.000246	-8.30843
262	0.30925	0.288	3.6E-06	0.000246	-8.30855
264	0.31175	0.2905	3.63E-06	0.000246	-8.30868
266	0.313	0.29175	3.65E-06	0.000246	-8.30874
268	0.31675	0.2955	3.69E-06	0.000246	-8.30893

270	0.31925	0.298	3.73E-06	0.000246	-8.30906
272	0.32	0.29875	3.73E-06	0.000246	-8.3091
274	0.32575	0.3045	3.81E-06	0.000246	-8.30939
276	0.327	0.30575	3.82E-06	0.000246	-8.30946
278	0.3285	0.30725	3.84E-06	0.000246	-8.30953
280	0.33025	0.309	3.86E-06	0.000246	-8.30962
282	0.333	0.31175	3.9E-06	0.000246	-8.30976
284	0.33575	0.3145	3.93E-06	0.000246	-8.3099
286	0.33775	0.3165	3.96E-06	0.000246	-8.31
288	0.34075	0.3195	3.99E-06	0.000246	-8.31015
290	0.34475	0.3235	4.04E-06	0.000246	-8.31036
292	0.346	0.32475	4.06E-06	0.000246	-8.31042
294	0.348	0.32675	4.08E-06	0.000246	-8.31052
296	0.34975	0.3285	4.11E-06	0.000246	-8.31061
298	0.353	0.33175	4.15E-06	0.000246	-8.31078

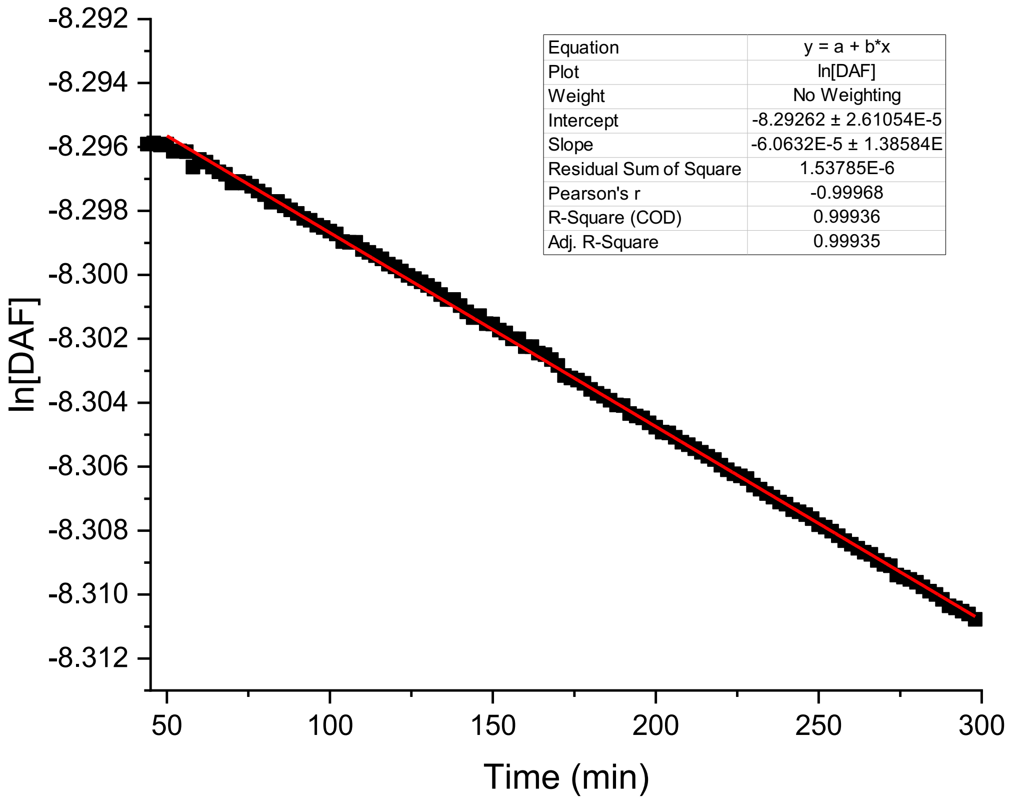

Supplement: DT-051-D2DT01713J-s001 [file DT-051-D2DT01713J-s001.pdf]
